# *De Novo* Access to BODIPY *C*-Glycosides as Linker-Free Nonsymmetrical BODIPY-Carbohydrate Conjugates

**DOI:** 10.1021/acs.joc.3c02907

**Published:** 2024-03-04

**Authors:** Clara Uriel, Dylan Grenier, Florian Herranz, Natalia Casado, Jorge Bañuelos, Esther Rebollar, Inmaculada Garcia-Moreno, Ana M. Gomez, J. Cristobal López

**Affiliations:** $ Instituto de Química Orgánica General, IQOG-CSIC, Juan de la Cierva 3, Madrid 28006, Spain; § Departamento de Química Física, Universidad del Pais Vasco, UPV-EHU, Apartado 644, Bilbao 48080, Spain; ¶ Instituto de Química y Física Blas Cabrera, CSIC, Serrano 119, Madrid 28006, Spain

## Abstract

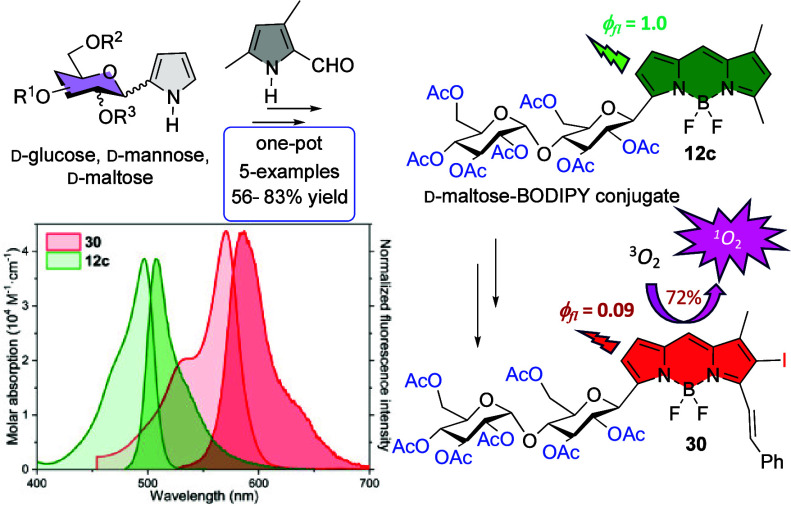

Recent years have witnessed an increasing interest in the synthesis and study of BODIPY-glycoconjugates. Most of the described synthetic methods toward these derivatives involve postfunctional modifications of the BODIPY core followed by the covalent attachment of the fluorophore and the carbohydrate through a “connector”. Conversely, few *de novo* synthetic approaches to linker-free carbohydrate-BODIPY hybrids have been described. We have developed a reliable modular, *de novo*, synthetic strategy to linker-free BODIPY-sugar derivatives using the condensation of pyrrole *C*-glycosides with a pyrrole-carbaldehyde derivative mediated by POCl_3_. This methodology allows labeling of carbohydrate biomolecules with fluorescent-enough BODIPYs within the biological window, stable in aqueous media, and able to display singlet oxygen generation.

## Introduction

**Figure 1:**
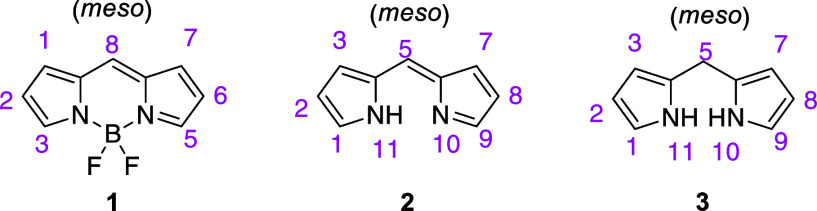
BODIPY (**1**), dipyrromethene (**2**), and dipyrromethane (**3**) (IUPAC numbering).

The development of small-molecule fluorophores is a fast-growing research area due to the increasing use of fluorescence imaging methods, in modern research fields, such as biochemistry, molecular biology, and materials science.^1^ Among these fluorophores, difluoroboron dipyrromethene (4,4-difluoro-4-bora-3a,4a-diaza-*s*-indacene) or BODIPY, i.e., **1** (Figure 1), fluorescent dyes have attracted considerable interest for biological and photophysical studies over the past two decades due to their excellent biocompatibility and tunable photophysical and chemical properties.^2^ Compared with commonly used fluorophores, the BODIPY dye family exhibits high molar absorption coefficients, insensitivity to the polarity and pH, sharp absorption and emission bands, and high fluorescence quantum yields.^3^ From a structural standpoint, the BODIPY scaffold consists of two planar pyrrole moieties connected by a methylene bridge and a boron difluoro moiety. From this structural arrangement, two key synthetic precursors to BODIPYs, dipyrromethene (i.e., **2**) and dipyrromethane (i.e., **3**) (Figure 1), can easily be envisioned.^4^ Remarkably, the BODIPY scaffold has been compared to a “rigidified” monomethine cyanine dye and a porphyrin’s sibling (“porphyrin’s little sister”).^5^

In this context, we have been interested in the emergent field of BODIPY-glycoconjugates,^6^ where carbohydrates, one of the four essential types of biomolecules, are covalently linked to BODIPY fluorophores.^7,8^ In these derivatives, either component may play a relevant role. For example, the carbohydrate moiety has been shown to internalize,^9^ solubilize in water,^10^ target,^11^ and reduce the cytotoxicity of the fluorophore.^12^ Conversely, the presence of the fluorophore in BODIPY-carbohydrate conjugates has shown its value in the investigation of carbohydrate-receptor interactions in biological systems with high sensitivity, by way of fluorescently labeled carbohydrates.^3,13,14^

In general, the synthesis of BODIPY-carbohydrate conjugates benefits from the variety of postfunctional modifications already known in borondipyrromethene derivatives.^15^ Therefore, the covalent linkage is often achieved by coupling conveniently functionalized BODIPYs with carbohydrates.^16^ Among these transformations, the copper-mediated azide–alkyne cycloaddition (CuAAC)^17^ “click-type”^18^ reactions of either alkyne- or azide-containing BODIPYs play a prominent role. Overall, these approaches lead to the formation of tethered BODIPY-glycoconjugates.

Conversely, to our knowledge, only a small number of approaches to linker-free BODIPY-carbohydrate conjugates have been reported in the literature. Some of these methods have been based on postfunctional BODIPY transformations to incorporate the sugar moiety. For instance, Brothers and co-workers reported on the preparation of sugar-*O*-BODIPY conjugates in which the fluorophore and the carbohydrate are directly linked through B–O–C bonds.^19^ We recently described a *C*-glycosylation approach leading to 2,6-disubstituted BODIPY-carbohydrate derivatives.^10b^

**Scheme 1:**
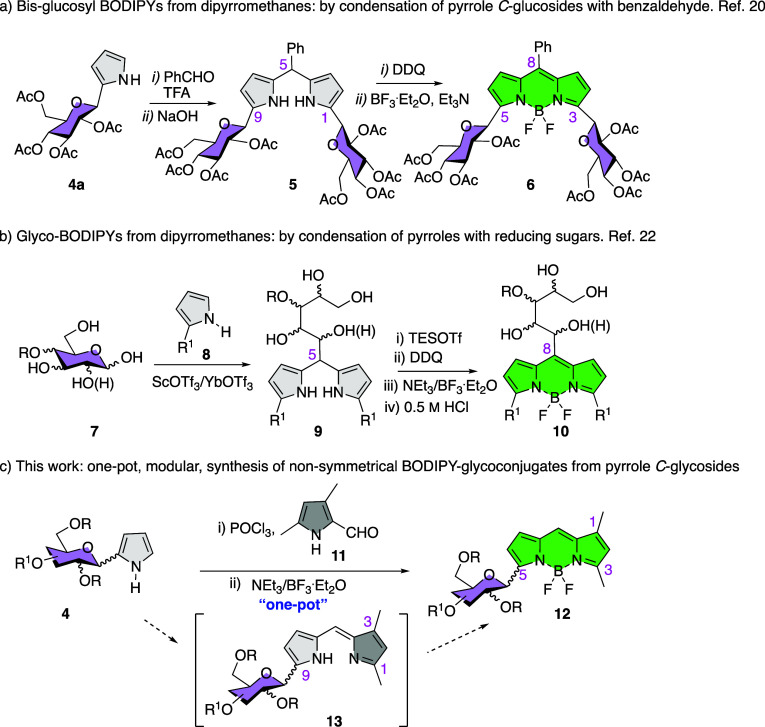
(a–c) *De Novo* Synthesis of Carbohydrate-BODIPY Hybrids

In addition, protocols based on the *de novo* synthesis of BODIPY-carbohydrate hybrids have been reported in the literature, albeit scarcely. Recently, two such methods have appeared. First, bis-glucosylated BODIPY dye **6** (Scheme 1a) was obtained by an oxidation/chelation protocol from 5-phenyl dipyrromethane **5**, which is readily obtained by condensation of β-*C*-glucosyl pyrrole **4a** with benzaldehyde (Scheme 1a).^20^ A second method, applied to a wide variety of saccharides, involves the condensation of unprotected reducing sugars (e.g., **7**, Scheme 1b) with a pyrrole unit (**8**) leading to C5-substituted dipyrromethane intermediates **9**,^21^ which can be processed by oxidation/chelation steps, to C8 sugar-substituted BODIPYs, e.g., **10** (Scheme 1b).^22^ Both methods are based on the well-precedented dipyrromethane → BODIPY transformation, leading to symmetrical BODIPYs **6** and **10** (Scheme 1a,b). In this manuscript, we report a reliable one-pot route to nonsymmetrical BODIPY *C*-glycosides, i.e., **12** (Scheme 1c). The overall transformation (**4** → **12**) includes the condensation of glycosyl pyrroles **4** with formyl pyrrole **11**, in the presence of POCl_3_ (Scheme 1c), to afford an intermediate *C*-glycosyl dipyrromethene **13**, which is then complexed *in situ* by adding triethyl amine and borontrifluoride diethyl etherate to yield BODIPY dyes **12**. The process has been performed on a gram scale in one case. Furthermore, postfunctional modifications on carbohydrate-BODIPY hybrids **12** have been implemented to improve the photophysical properties of these BODIPYs.

## Results and Discussion

We were interested in investigating *de novo* approaches to linker-free BODIPY-carbohydrate derivatives involving dipyrromethene-based, e.g., **2** (Figure 1), instead of dipyrromethane-based glycosyl intermediates, e.g., **3** (Figure 1). The use of the former would circumvent the need for the oxidation step and allow the BODIPY synthesis to be carried out as a one-pot operation. At the onset of this work, the interest in glycosyl dipyrrane derivatives had been mainly triggered by their use as building blocks in the synthesis of glycoporphyrins and derivatives.^23^ In this context, 5-glycosyl dipyrromethanes, e.g., **9** (Scheme 1b),^24^ had been employed as convenient synthetic precursors for *meso*-glycoporphyrins.^25^ On the other hand, *C*-glycosylation^26^ at C-1 of an unsubstituted dipyrromethane with gluco-, galacto-, and mannopyranosyl trichloroacetimidate glycosyl donors,^27^ leading to 1-glycosyl dipyrromethanes, has also been reported.^28^ However, the subsequent boron chelation leading to a glyconjugated BODIPY was not explored.

**Scheme 2:**
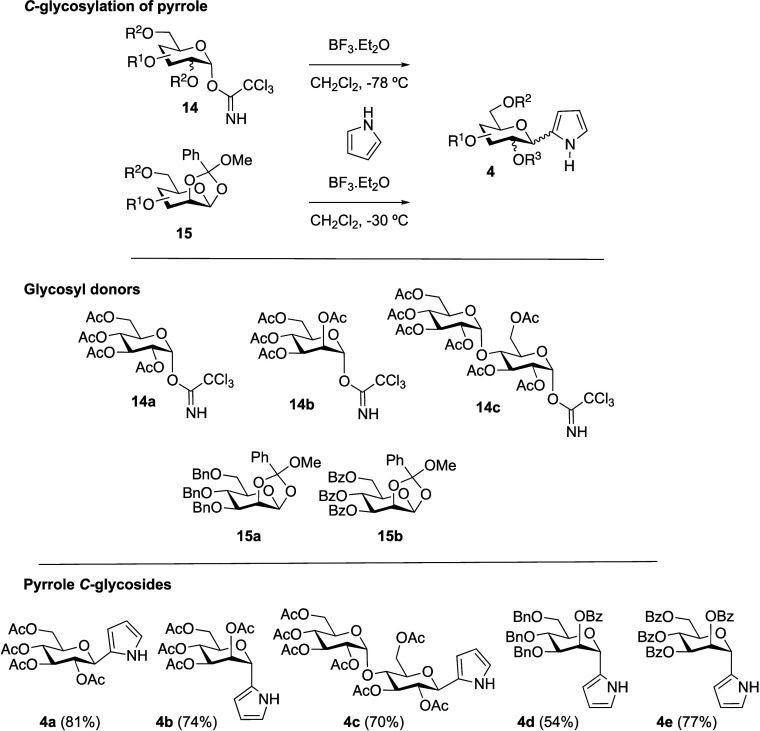
Synthesis of *C*-Glycosyl Pyrroles

According to plan, we aimed at exploring well-established synthetic routes to BODIPYs involving glycosyl dipyrromethene intermediates. As starting materials, we selected pyrrole *C*-glycosides **4** (Scheme 2), readily accessible by *C*-glycosylation of pyrrole. Accordingly, as glycosyl donors, we selected trichloroacetimidates **14a**, **14b**, and **14c**,^27^ derived from d-glucose, d-mannose, and d-maltose, respectively, as well as differently protected d-mannose-derived 1,2-methyl orthoesters (MeOEs)^29,30^**15a,b** (Scheme 2). The glycosylation of pyrrole with glycosyl trichloroacetimidate donors **14a**–**c**, mediated by BF_3_·Et_2_O at −78 °C in CH_2_Cl_2_, produced good yields of pyrrole *C*-glycosides **4a**, **4b**, and **4c**, respectively. On the other hand, the reaction of MeOEs **15a,b** with pyrrole, promoted by BF_3_·Et_2_O at −30 °C in CH_2_Cl_2_, provided good yields of pyrrole *C*-mannopyranosides **4d** and **4e**, respectively. All of these glycosylations took place with complete stereoselectivity providing exclusively one of the two possible anomeric isomers.

**Scheme 3:**
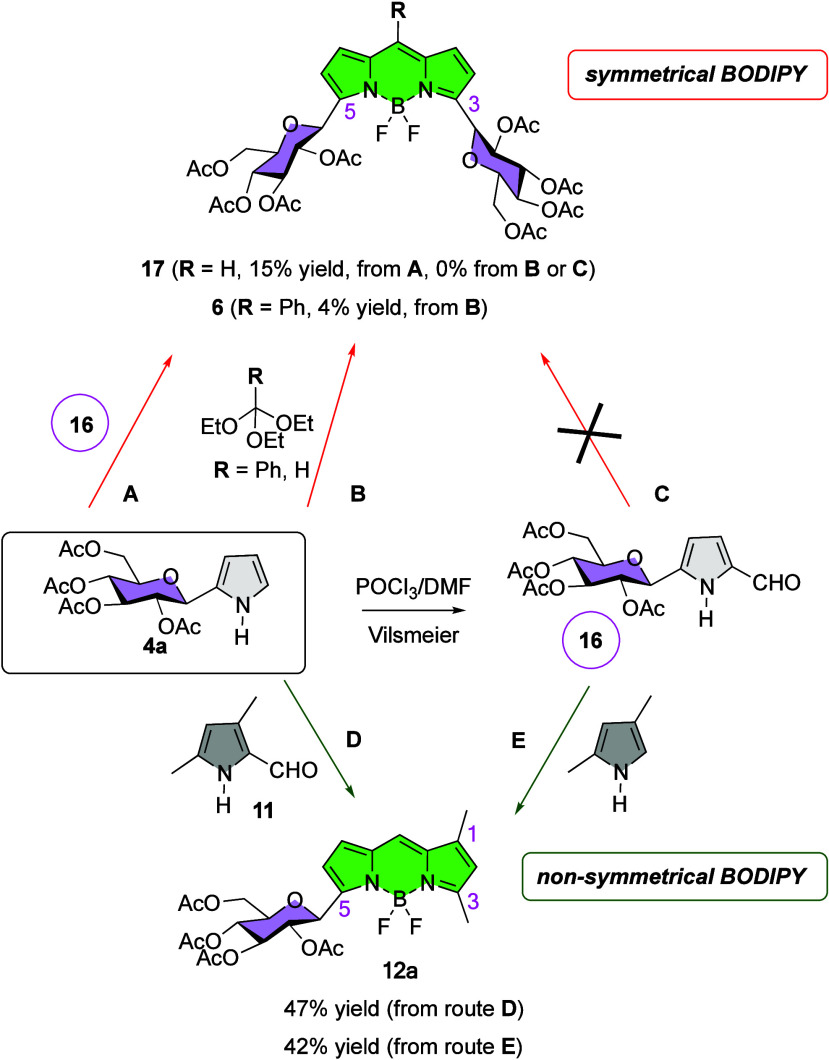
Synthetic Routes to BODIPY-Carbohydrate Conjugates

With glycopyrroles **4** in hand, we set out to explore several synthetic routes to BODIPYs (routes **A** to **E**, Scheme 3). All these routes involved the additional *in situ* chelation step (not shown in the scheme) of the dipyrromethene intermediates to yield the desired borondipyrromethene derivatives. In our hands, implementation of route **A** (Scheme 3) consisting of the condensation between pyrrole *C*-glucoside **4a** and its corresponding pyrrole-carbaldehyde **16**, easily prepared by the Vilsmeier–Haack formylation of **4a**,^31^ afforded low yield (15%) of bis-glucosyl BODIPY **17**, after chelation (Scheme 3 and Scheme S2 in the SI). Alternatively, according to route **B** (Scheme 3), the reaction of **4a** with ethyl orthoformate or ethyl orthobenzoate either gave no BODIPY (**17**) formation or trace yields of BODIPY **6** (4%), respectively (Scheme S1 in the SI). Along these lines, the application of route **C** (Scheme 3), involving the one-pot condensation-decarbonylation of pyrrole 2-carbaldehyde **16**,^32^ yielded none of the desired BODIPY **17**.

Conversely, better results were obtained in the preparation of nonsymmetrical BODIPY-carbohydrate conjugate **12a** according to routes **D** and **E** (Scheme 3). Such routes involved the condensation of either glycosyl pyrrole **4a** with commercially available 2-formyl-3,4-dimethyl pyrrole (**11**) (route **D**) or the condensation of **16** with 2,4-dimethyl pyrrole (route **E**).

Therefore, condensation of **4a** with formyl pyrrole **11**, mediated by POCl_3_,^33−35^ (route **D**, Scheme 3), followed by chelation with boron trifluoride etherate (Et_3_N, then BF_3_·Et_2_O) at room temperature (r.t.), gave BODIPY **12a** in 47% yield (Scheme 3). Likewise, POCl_3_-mediated condensation of d-glucose-pyrrole-carbaldehyde **16**, with 2,4-dimethyl pyrrole, yielded BODIPY **12a**, although in a slightly lower yield of 42% (Scheme 3). Based on these results and considering that route **D** is more convergent than route **E** since the latter required an additional Vilsmeier reaction on glucoside **4a**, we decided to pursue our investigations by optimizing route **D**.

**Scheme 4:**
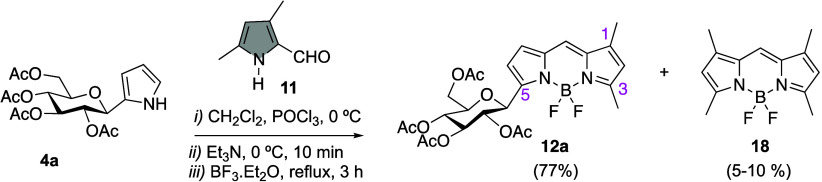
Access to BODIPY **12a** by an Optimized Route **D**

Interestingly, during the course of these investigations, we found that when the chelation step was carried out at reflux (CH_2_Cl_2_), the yield of BODIPY **12a** could rise up to 77% (Scheme 4). It is important to note that in this transformation, the formation of variable amounts of symmetrical BODIPY **18** (5–15%) was always observed.^36,37^

**Scheme 5:**
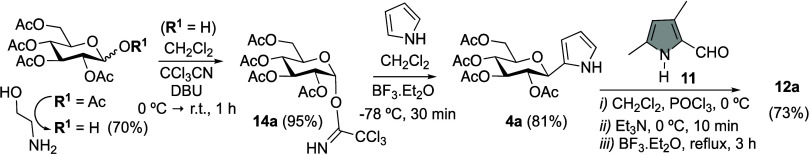
Gram-Scale Synthesis of **12a** from 1,2,3,4,6-Penta-*O*-acetyl-d-glucose

To validate the route, we next carried out the gram-scale synthesis of **12a** (Scheme 5). Thus, chemoselective anomeric deacetylation^38^ of commercially available 1,2,3,4,6-penta-*O*-acetyl-d-glucose provided access to 2,3,4,6-tetra-*O*-acetyl-d-glucopyranose, which upon the reaction with trichloroacetonitrile in the presence of 1,8-diazabicyclo(5.4.0)undec-7-ene (DBU) provided glucosyl donor **14a**.^27,39^*C*-Glycosylation of pyrrole with glucosyl trichloroacetimidates **14a** took place smoothly at −78 °C in the presence of BF_3_·Et_2_O, leading to pyrrole *C*-glucoside **4a**. Finally, condensation of **4a** with pyrrole-carbaldehyde **11** provided BODIPY **12a** in a respectable 73% yield. Formyl pyrrole **11**, which is commercially available, could also be uneventfully prepared by Vilsmeier–Haack formylation of 2,4-dimethyl pyrrole.^32a^

**Scheme 6:**
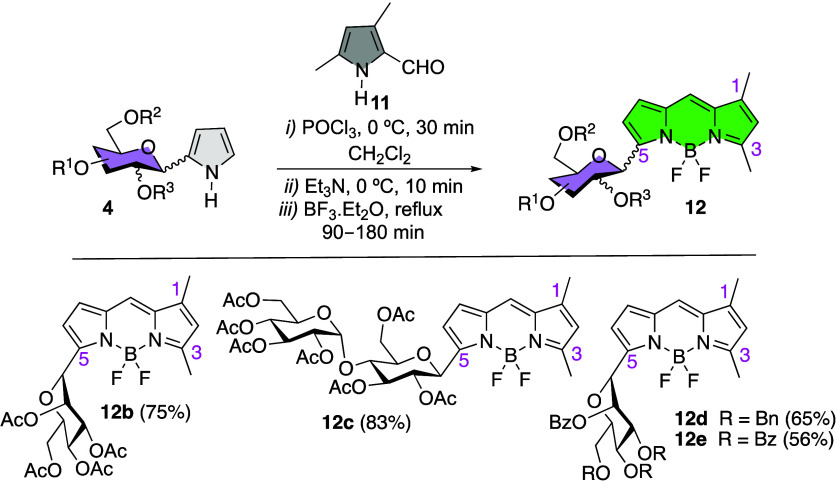
Synthesis of Linker-Free Carbohydrate-BODIPY Hybrids **12b**–**e**

The scope of this approach was next investigated with the synthesis of additional carbohydrate-BODIPY hybrids **12b**–**e**, from pyrrole *C*-glycosides **4b**–**e**, respectively (Scheme 6). Thus, the protocol was successfully applied to *C*-glycosyl derivatives **4b**–**e**, providing good yields of d-mannose- and d-maltose-derived BODIPYs **12b**–**e**.

Finally, since the chemical and photophysical properties of the BODIPY fluorophores can be modulated by postfunctional modifications on their skeleton, we decided to evaluate a series of transformations on d-glucosyl and d-maltosyl BODIPY derivatives, **12a** and **12c**, respectively. Thus, we aimed at obtaining water-soluble, red-edge fluorescent derivatives,^40^ as well as dyes for efficient singlet oxygen production.^41^

**Scheme 7:**
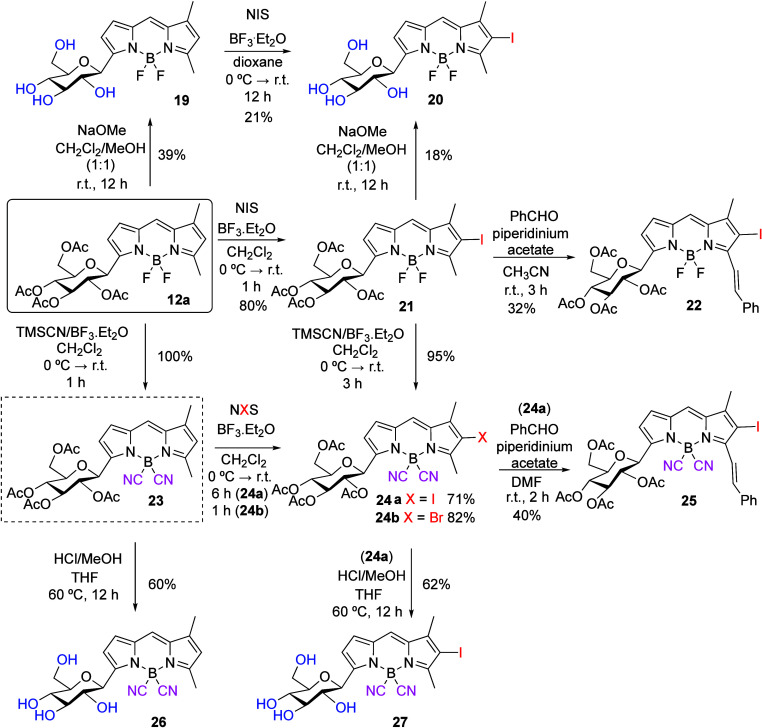
Postfunctional Modifications on BODIPY **12a**

Along these lines, BODIPY **12a** was transformed into water-soluble BODIPY **19**, by saponification of the sugar acetyl groups under controlled alkaline conditions (Scheme 7).^42^ On the other hand, iodination at C2 (NIS/BF_3_·Et_2_O) was carried out in compounds **19** and **12a** (Scheme 7) leading to iodo-BODIPYs **20** and **21**, respectively. The latter was transformed into C3-styryl BODIPY **22** by Knoevenagel condensation with benzaldehyde, mediated by piperidinium acetate. Owing to the special properties associated with B(CN)_2_-BODIPYs,^43^ we prepared BODIPY **23** from **12a**. Thus, starting from compound **23**, by way of related synthetic transformations, we were able to access iodinated (NIS) and brominated (NBS), B(CN)_2_-BODIPYs **24a,b**, respectively, and iodo-styryl B(CN)_2_-BODIPY **25** (from **24a**). The corresponding unprotected *C*-glucosyl BODIPYs **26** and **27** were obtained by removal of the acetyl protecting groups under acidic (HCl/MeOH) rather than alkaline conditions owing to the higher stability of B(CN)_2_-BODIPYs compared to BF_2_-BODIPYs,^44^ under acidic media from derivatives **23** and **24a**, respectively.

**Scheme 8:**
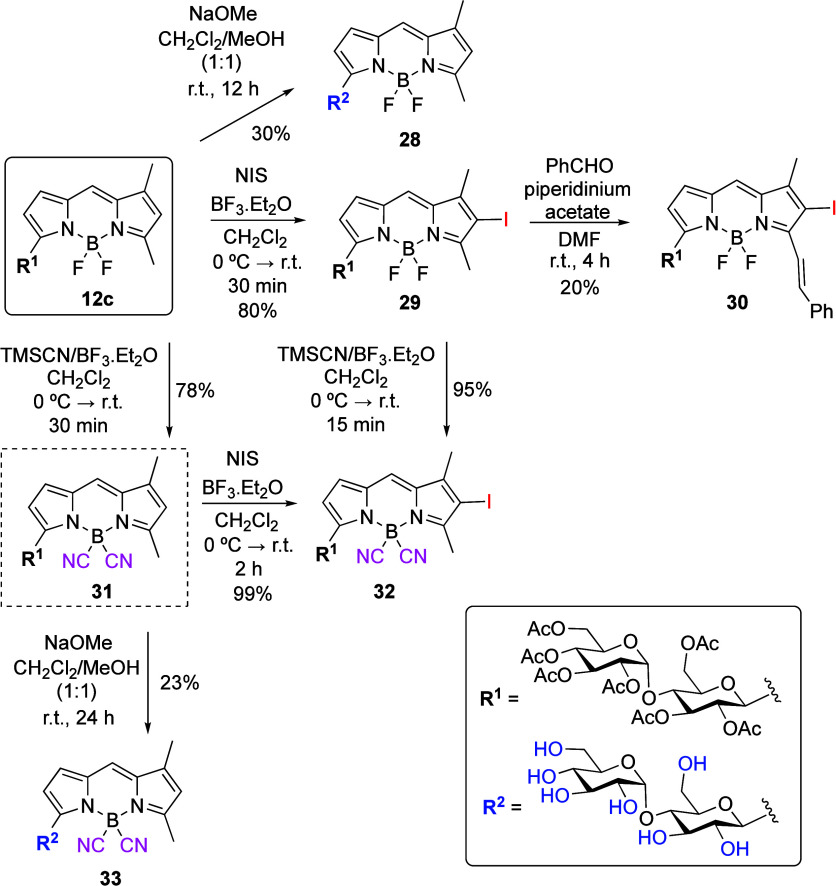
Postfunctional Modifications on BODIPY **12c**

On the other hand, a related set of reactions carried out on BODIPY-labeled disaccharides **12c** (Scheme 8) allowed the preparation of analogous linker-free BODIPY *C*-maltosides. For instance, saponification of **12c** gave water-soluble BODIPY disaccharide **28**, whereas iodination produced iodo-BODIPY **29**, which could undergo a Knoevenagel condensation resulting in the formation of styryl-iodo-BODIPY disaccharide **30**. Again, the transformation of BODIPY **12c** into B(CN)_2_-BODIPY **31** took place efficiently. The latter was then transformed into the iodinated derivative **32**. Compound **31** could be saponified under alkaline conditions to protecting-group-free maltoside **33** (Scheme 8). Conversely, attempted acetyl hydrolysis on **31** to **33**, under acidic conditions, gave compound **26**, where cleavage of the anomeric bond of the terminal glucosyl residue had taken place along with the desired de-*O*-acetylation. On the other hand, compound **32** could also be efficiently accessed from the corresponding BF_2_-BODIPY **29** by fluoride to cyanide exchange (TMSCN/BF_3_·Et_2_O) (Scheme 8).

The photophysical behavior of the heavy-atom-free glyco-BODIPYs (**12a,c** and **23**, as well as the corresponding unprotected derivatives **19**, **28**, and **26**, respectively) resembled that reported for conventional BODIPYs (such as **1** or **18**), supporting the C5 position as suitable for the direct attachment of carbohydrates (Table 1 and Table S1 in the SI). Indeed, albeit the absorption capacity of these glycosyl derivatives decreased (mainly in the unprotected derivatives), all of them displayed strong and sharp emission bands in organic solvents (see methanol in Table 1), with fluorescence efficiencies surpassing 80% and even reaching the 100% for **12c** (Table 1). Moreover, the unprotected glycosyl-BODIPYs were also soluble and fluorescent in water (Figure S1 and Table S1 in the SI), especially BODIPY **28** bearing a disaccharidic unit, which also ensured a higher solubility limit (around 0.1 to 1 mM without signs of aggregation-induced quenching, consistent with our previously reported results for related carbohydrate-BODIPY hybrids).^10b,42^ In this regard, note that the at-boron replacement of the fluorine atoms by cyano moieties is a successful strategy to further enhance the fluorescence efficiency (Table 1).^43^ In all cases, the B(CN)_2_-BODIPYs (e.g., **23** and **26**) displayed improved fluorescence efficiency with respect to the corresponding BF_2_-BODIPY (**12a** and **19**). This enhancement of the emission efficiency, attributed to a decrease in nonradiative probability, is strongly correlated with an increased photostability during prolonged irradiation of dyes based on B(CN)_2_ pattern substitution.^43^ Photostability in water of B(CN)_2_-BODIPY **26** and its corresponding BF_2_-BODIPY **19** was assessed by monitoring the evolution of laser-induced fluorescence under repeated pumping pulses (see the Experimental Section). Consistently, dye **26**, which exhibited higher efficiency, also demonstrated greater photostability; its laser-induced emission remained unchanged after 50,000 pump pulses, while its fluorinated counterpart **19** experienced a 25% decrease in emission under identical experimental conditions.

**Table 1:**
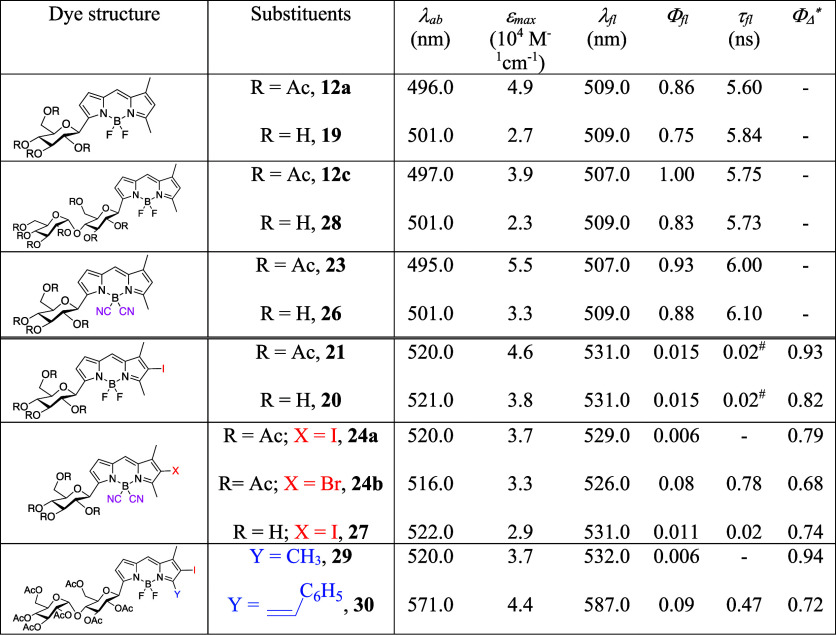
Main Photophysical Properties of the Glyco-BODIPYs in Diluted (2 μM) Solutions of Methanol^a^

a Absorption (λ_ab_) and fluorescence (λ_fl_) wavelength, molar absorption coefficient at the maximum (ε_max_), fluorescence quantum yield (Φ), lifetime (τ), and singlet oxygen generation quantum yield (Φ_Δ_). Full photophysical data in other solvents are listed in Tables S1 and S2 in the SI. The model molecular structures in each case have been added for the sake of clarity.

b Data in chloroform.

c Main lifetime (contribution of >90%) of the multiexponential fit (see Table S1 in the SI).

The C2 monobromination of the glycosyl-BODIPY **24b** promoted a slight bathochromic shift of the spectral absorption and fluorescence bands but modified strongly the fluorescence signatures (Table 1 and Table S2 in the SI). As it was expected, the bromine heavy atom enhanced the intersystem crossing (ISC), reducing the fluorescence efficiency to less than 10%. Alternatively, the ISC-mediated triplet-state population enabled an effective generation of singlet oxygen^45^ (approaching the 70%, Table 1). In this regard, the dual response of this monobrominated glycosyl-BODIPY could be suitable for phototheragnostic applications^46^ since it is able to generate a notable amount of singlet oxygen upon irradiation while retaining enough fluorescence output to visualize the process.

By going from the brominated BODIPY **24b** to the monoiodinated glycosyl-BODIPYs **21**, **24a**, and **29** (as well as the unprotected derivatives **20** and **27**), the heavy-atom effect increased the spin–orbit coupling, consequently reducing even more the fluorescence emission that became almost negligible (Table 1 and Table S2 in the SI). Note that in this case, the replacement of fluorine atoms by cyano moieties was not enough to improve the fluorescence response since the iodinated B(CN)_2_-BODIPYs **24a** and **27** exhibited also a faint emission (Table 1). Consequently, these dyes enabled an efficient generation of singlet oxygen, reaching values that surpassed 75% and even 90% (see iodinated BF_2_-BODIPYs **21** and **29**, Table 1). Therefore, these iodinated BODIPYs could act as efficient photosensitizers for photodynamic therapy (PDT). Among the tested compounds, **20** and **27** show promise for their potential use in photodynamic therapy (PDT). While they generate slightly less singlet oxygen than their protected counterparts, compounds **21** and **24a** (see Table 1), they have the advantage of being fully soluble in water and remaining stable over time. These qualities make them promising candidates for further preclinical testing as PDT photosensitizers.

**Figure 2:**
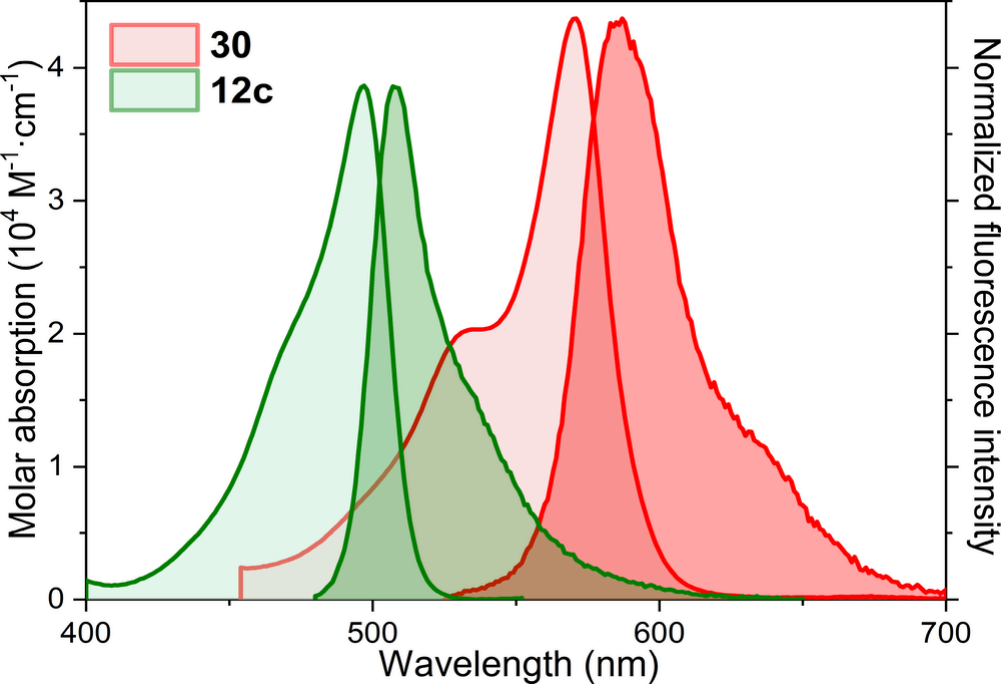
Absorption and normalized fluorescence (darker shading) spectra of glycosyl-BODIPYs **12c** and **30**, bearing disaccharidic maltosyl residues, in diluted (2 μM) solutions of methanol.

Further functionalization of the monoiodinated glycosyl-BODIPY **29**, by grafting one styryl group at C3, led to BODIPY **30**, which contains a π-extended chromophoric framework. This caused a bathochromic shift of the spectral bands toward the red edge of the visible region (Figure 2). Indeed, the long-wavelength tail of the fluorescence spectrum fell within the biological window (λ > 650 nm). Thus, although the iodine substitution enhanced the triplet-state population, this π-extended derivative retained a remarkable fluorescence efficiency (around 10% at 590 nm, Table 1), together with a singlet oxygen generation of 72%, which was lower than the yield recorded from its iodinated glycosyl-BODIPY analog **29** without a styryl moiety that reached up to 94% (Table 1). Therefore, as detailed for **24b**, BODIPY **30** could be a suitable fluorescent photosensitizer for bioimaging-guided PDT, especially once the maltosyl residue is unprotected to ensure solubility in the physiological media.

In a first approach, the population of long-lived triplet excited states in these new derivatives was proven by nanosecond-resolved transient absorption (ns-TA) spectroscopy (see the Experimental Section for details). The ns-TA spectra recorded from **21** and **30**, as representative examples of the behavior followed by these dyes, showed the characteristic profile previously recorded for iodinated BODIPYs, with two broad absorption bands flanking the ground-state bleaching band centered at 500 nm (Figure S2 in the SI).^47^ The decay of the positive signals had monoexponential character within the microsecond scale, suggesting that just one long-lived transient triplet state was populated.

**Figure 3:**
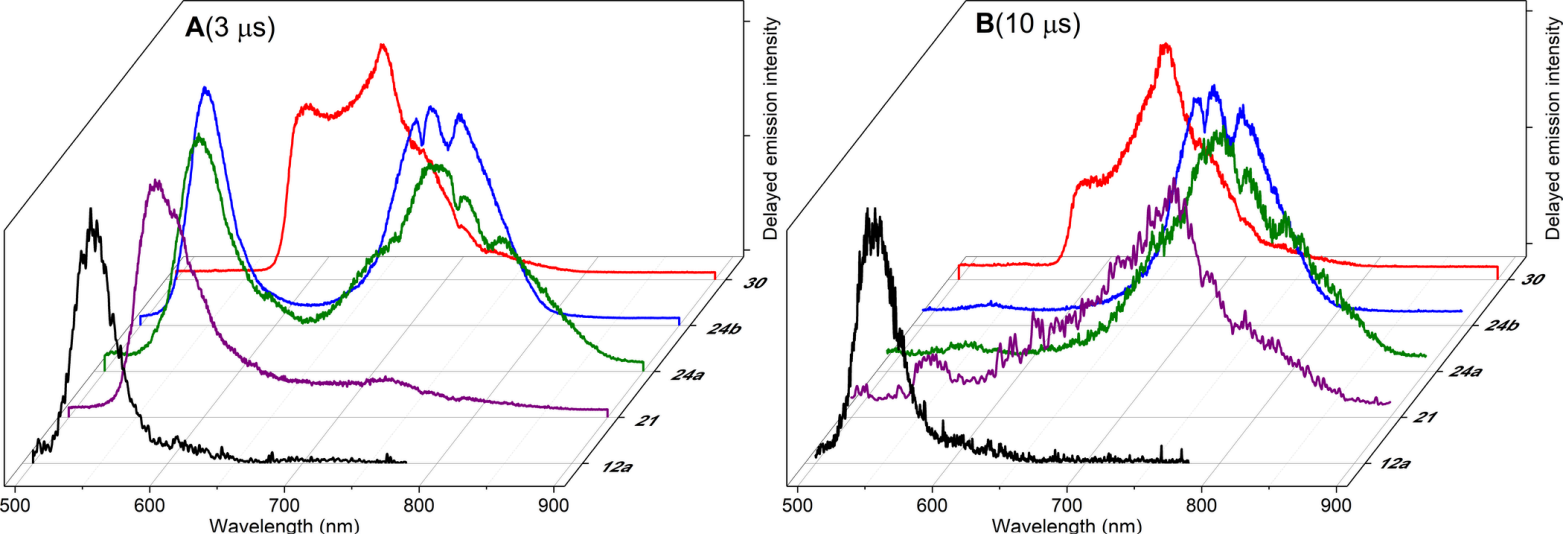
Delayed emission spectra of BODIPYs **12a**, **21**, **24a**, **24b**, and **30** in methanol, recorded at (A) 3 and (B) 10 μs time delay after photoexcitation at 532 nm under ambient conditions. Optically matched solutions were used.

To get deeper insights into the long-lived emissions of the new dyes, time-gated spectral analysis induced upon laser excitation at 532 nm in aerated methanolic solutions was performed at room temperature (see the Experimental Section). As it has been reported for other heavy-atom-free single BODIPY fluorophores,^48^ dye **12a** exhibited delayed emission in the 500–600 nm spectral region with a profile similar to its prompt fluorescence (Figure 3 and Figure S3 in the SI). Considering that this emission can be recorded at delay times up to 20 μs, it must unequivocally imply involvement of long-lived triplet excited states harvested through reverse ISC (rISC), giving rise to the detected thermally activated delayed fluorescence (TADF).

Heavy-atom functionalization of these BODIPYs led to the growing of a dual delayed emission since a new and broad long-wavelength band raised in the 650–900 nm spectral region. When increasing the time delay up to 10 μs, the spectral profile evolved toward a single band since the delayed fluorescence around 560 nm virtually disappeared, while the longer-lasting emission placed at the red edge of the spectrum could be recorded up to 50 μs (Figure 4 and Figure S3 in the SI). On the basis of the T_1_ energy level detected and computed for other *F*-BODIPYs as well as boron-substituted related dyes,^48^ this long-wavelength band can be reliably assigned to the phosphorescence emission.

With respect to the B(CN)_2_-substituted BODIPY **24a**, its corresponding BF_2_-BODIPY **21** exhibited a faint phosphorescence emission that became undetectable after 10 μs upon photoexcitation (Figure S3 in the SI). In this regard, this effectively quenched phosphorescence emission was in agreement with the highly efficient singlet oxygen generation recorded from **21** that reached a quantum yield up to 93%. An exception to the behavior described above was the delayed emission recorded from the red-emitting dye **30**. In this case, the spectral profile, resulting from the delayed fluorescence that peaked at 600 nm and strongly overlapped with its phosphorescence, remained unaltered on increasing the time delay from 3 to 50 μs (Figure 4 and Figure S3 in the SI). The simultaneous detection of delayed fluorescence and phosphorescence emission sustains the balanced fluorescence and singlet oxygen generation recorded from the herein developed dyes advancing their potential as valuable theragnostic agents.^49^

## Conclusions

We have developed a modular, *de novo*, one-pot, synthetic strategy to linker-free BODIPY-carbohydrate derivatives based on the condensation of readily accessible pyrrole *C*-glycosides with a pyrrole-carbaldehyde derivative mediated by POCl_3_ followed by chelation with boron trifluoride etherate (Et_3_N, then BF_3_·Et_2_O). Furthermore, fine adjustment by postfunctional modifications allows ready access to water-soluble, linker-free BODIPY-carbohydrate conjugates, with tailored photophysical properties, which sustain a balanced fluorescence and singlet oxygen generation advancing their potential as valuable phototheragnostic agents. Thus, the overall approach allows the preparation of multifunctional BODIPYs with diverse substitution at C2, C3, C5, and at-boron of the chromophoric core. Such derivatives permit the labeling of carbohydrates with fluorescent-enough BODIPYs amenable to be tracked by bioimaging within the biological window, stable in the physiological media, and displaying singlet oxygen generation, which might prove useful in photodynamic therapy.

## Experimental Section

### General Information

All solvents and reagents were obtained commercially and used as received unless stated otherwise. Residual water was removed from starting compounds by repeated coevaporation with toluene. All moisture-sensitive reactions were performed in dry flasks fitted with glass stoppers or rubber septa under a positive pressure of argon. In general, reactions were carried out at room temperature (r.t.) unless indicated otherwise. Heating blocks were utilized as heat sources for all reactions requiring elevated temperatures. Anhydrous MgSO_4_ or Na_2_SO_4_ was used to dry organic solutions during workup. Evaporation of the solvents was performed under reduced pressure using a rotary evaporator. Flash column chromatography was performed using 230–400 mesh silica gel. Thin-layer chromatography was conducted on Kieselgel 60 F254. Spots were observed first under UV irradiation (254 nm) then by charring with a solution of 20% aqueous H_2_SO_4_ (200 mL) in AcOH (800 mL). All melting points were determined with a Stuart SMP-20 apparatus. Optical rotations were measured on a Jasco P2000 polarimeter with [α]_D_^25^ values reported in degrees with concentrations expressed in g/100 mL. ^1^H, ^13^C, ^19^F, and ^11^B NMR spectra were recorded in CDCl_3_ or CD_3_OD at 300, 400, or 500 MHz (^1^H NMR), 75, 101, or 126 MHz (^13^C NMR), 376 MHz (^19^F NMR), and 128 MHz (^11^B NMR), respectively. Chemical shifts are expressed in parts per million (δ scale) downfield from tetramethylsilane and are referenced to residual protium in the NMR solvent (CHCl_3_: δ 7.25 ppm, CH_3_OH: δ 4.87 ppm). Coupling constants (*J*) are given in Hz. All presented ^13^C NMR spectra are proton-decoupled. Mass spectra were recorded by direct injection with an accurate mass Q-TOF LC/MS spectrometer equipped with an electrospray ion source in the positive mode. 3,5-Dimethylpyrrole-2-carbaldehyde **11** was prepared according to previously reported Vilsmeier formylation.^32a^ The synthesis of trichloroacetimidate glycosyl donors **14b** and **14c** was carried out in two steps starting from peracetylated mannose and maltose, respectively, in the same way as described for its d-glucose analog **14a** (Gram-Scale Synthesis of BODIPY **12a**, Scheme 5); the data of the products thus prepared are in accordance with those described in the bibliography.^50^ Orthoester **15a**(51) was synthesized according to literature procedures, and methyl orthobenzoate **15b** was prepared from tetra-*O*-benzoyl-α-d-mannopyranosyl bromide^52^ following a published method.^53^ 1,2,3,4,6-Penta-*O*-acetyl-d-mannopyranose, 1,2,3,6,2′,3′,4′,6′-octa-*O*-acetyl-d-maltopyranose, and 1,2,3,4,6-penta-*O*-acetyl-d-glucopyranose were obtained from commercial sources.

### General Procedures

#### General Procedure A: Preparation of C2-Glycosylated Pyrroles

The corresponding glycosyl donor (1 equiv) and pyrrole (5 equiv) were dissolved in anhydrous CH_2_Cl_2_ and cooled to an indicated temperature. Then, BF_3_·Et_2_O (0.5 equiv) was added. The reaction mixture was stirred until TLC showed a complete disappearance of the glycosyl donor; then, Et_3_N was added (3.5 equiv); after stirring for 5 min, the crude material was concentrated *in vacuo*. The residue was purified by silica column chromatography.

#### General Procedure B: Preparation of *C*-Glycosyl-BODIPYs

A solution of the corresponding glycosyl pyrrole (1 equiv) and 3,5-dimethylpyrrole-2-carbaldehyde **11** (1.2 equiv) in anhydrous CH_2_Cl_2_ (5–20 mL/mmol) was cooled at 0 °C. Then, POCl_3_ (3 equiv) was added dropwise. The solution was stirred at 0 °C for 15 min and then at room temperature (r.t.) overnight. The reaction mixture was recooled at 0 °C, and triethylamine (10 equiv) and BF_3_·Et_2_O (6 equiv) were added dropwise and heated to 40 °C and stirred at that temperature for 3 h. The solution was then diluted with CH_2_Cl_2_ (50 mL/mmol), washed with water (2 × 50 mL/mmol) and NaHCO_3_ (50 mL/mmol), dried over Na_2_SO_4_, and concentrated under reduced pressure. The residue was purified by silica column chromatography.

#### Reactions and Compounds’ Characterization

##### 2-(2′,3′,4′,6′-Tetra-*O*-acetyl-β-d-glucopyranosyl)-pyrrole (**4a**)

The compound **4a** was prepared according to the general procedure A starting from 2,3,4,6-tetra-*O*-acetyl-β-d-glucopyranosyl-trichloroacetimidate (2.3 g, 4.7 mmol), pyrrole (1.4 mL, 20.3 mmol), and BF_3_·Et_2_O (260 μL, 2 mmol) at −78 °C (30 min). After workup (Et_3_N, 2 mL), the residue was purified by silica gel column chromatography (hexane/ethyl acetate, 8:2 to 6:4) to afford **4a**(54,20) (1.5 g, 81%). ^1^H NMR (CDCl_3_, 400 MHz): δ 8.42 (s, 1H), 6.77 (td, *J* = 2.6, 1.7 Hz, 1H), 6.14–6.10 (m, 2H), 5.31 (t, *J* = 9.35 Hz, 1H), 5.19 (dd, *J* = 9.2, 5.0 Hz, 1H), 5.15 (dd, *J* = 9.4, 5.1 Hz, 1H), 4.52 (d, *J* = 9.9 Hz, 1H), 4.28 (dd, *J* = 12.4, 4.9 Hz, 1H), 4.17–4.07 (m, 1H), 3.82 (ddd, *J* = 9.9, 4.9, 2.3 Hz, 1H), 2.07 (s, 3H), 2.04 (s, 3H), 2.01 (s, 3H), 1.90 (s, 3H). HRMS (ESI/Q-TOF) *m*/*z*: [M + Na]^+^ calcd for C_18_H_23_NNaO_9_, 420.1271; found, 420.1276.

##### 2-(2′,3′,4′,6′-Tetra-*O*-acetyl-α-d-mannopyranosyl)-pyrrole (**4b**)

Compound **4b** was prepared according to the general procedure A starting from 2,3,4,6-tetra-*O*-acetyl-β-d-mannopyranosyl-trichloroacetimidate (2 g, 4.1 mmol), pyrrole (1.4 mL, 20.3 mmmol), and BF_3_·Et_2_O (260 μL, 2 mmol) at −78 °C (30 min). After workup (Et_3_N, 2 mL), the residue was purified by silica gel column chromatography (hexane/ethyl acetate, 8:2 to 6:4) to afford **4b**(47) (1.20 g, 74%). ^1^H NMR (CDCl_3_, 400 MHz): δ 8.78 (s, 1H), 6.26 (d, *J* = 1.9 Hz, 1H), 5.47–5.43 (m, 1H), 5.40–5.34 (m, 2H), 4.30–4.06 (m, 4H), 2.17 (s, 3H), 2.06 (s, 3H), 2.04 (s, 3H), 1.98 (s, 3H). HRMS (ESI/Q-TOF) *m*/*z*: [M + Na]^+^ calcd for C_18_H_23_NNaO_9_, 420.1271; found, 420.1266.

##### 2-(2′,3′,6′,2″,3″,4”6″-Hepta-*O*-acetyl-β-d-maltopyranosyl)-pyrrole (**4c**)

This compound was prepared according to the general procedure A starting from 2,3,6,2′,3′,4′,6′-hepta-*O*-acetyl-β-d-maltopyranosyl-trichloroacetimidate (600 mg, 0.77 mmol), pyrrole (0.25 mL, 3.84 mmol), and BF_3_·Et_2_O (60 μL, 0.46 mmol) at −78 °C during 2h. After workup (Et_3_N, 360 μL), the residue was purified by silica gel column chromatography (hexane/ethyl acetate, 8:2 to 6:4) to afford **4c**(55) (368.6 mg, 70%). ^1^H NMR (CDCl_3_, 400 MHz): δ 8.33 (s, 1H), 6.76 (d, *J* = 1.7 Hz, 1H), 6.10 (m, 2H), 5.46 (d, *J* = 4.1 Hz, 1H), 5.42–5.33 (m, 2H), 5.07 (t, *J* = 9.9 Hz, 1H), 5.02 (t, *J* = 9.6 Hz, 1H), 4.89 (dd, *J* = 10.5, 4.0 Hz, 1H), 4.56 (d, *J* = 9.9 Hz, 1H), 4.48 (dd, *J* = 12.2, 2.5 Hz, 1H), 4.28–4.23 (m, 2H), 4.11–4.02 (m, 2H), 4.02–3.94 (m, 1H), 3.81 (ddd, *J* = 9.7, 4.4, 2.4 Hz, 1H), 2.13 (s, 3H), 2.11 (s, 3H), 2.06 (s, 3H), 2.03 (s, 3H), 2.01 (s, 6H), 1.90 (s, 3H).). ^13^C {^1^H} NMR (CDCl_3_, 125 MHz): δ 170.8, 170.7, 170.4, 170.1, 169.8, 169.6, 125.9, 118.8, 108.7, 107.9, 95.8, 77.4, 76.3, 73.9, 73.0, 72.2, 70.1, 69.5, 68.7, 68.1, 63.3, 61.6, 21.1, 21.0, 20.85, 20.7, 20.6. HRMS (ESI/Q-TOF) *m*/*z*: [M + Na]^+^ calcd for C_30_H_39_NNaO_17_, 708.2116; found, 708.2112.

##### 2-(2′-*O*-Benzoyl-3′,4′,6′-tri-*O*-benzyl-α-d-mannopyranosyl)-pyrrole (**4d**)

The compound **4d** was prepared according to the general procedure A starting from 3,4,6-tri-*O*-benzyl mannopyranosyl 1,2-orthobenzoate **15a** (250 mg, 0.44 mmol), pyrrole (150 μL, 2.2 mmol), and BF_3_·Et_2_O (28 μL, 0.22 mmol) at −30 °C (1 h). After workup (Et_3_N, 2 mL), the residue was purified by silica gel column chromatography (hexane/ethyl acetate, 8:2) to afford **4d** (143 mg, 54%). ^1^H NMR (CDCl_3_, 400 MHz): δ 8.47 (bs, 1H), 8.19–8.10 (m, 2H), 7.63–7.54 (m, 1H), 7.49–7.24 (m, 15H), 7.21–7.13 (m, 2H), 6.76–6.72 (m, 1H), 6.12–6.08 (m, 2H), 5.95 (m, 1H), 5.23 (bs, 1H), 4.86 (d, *J* = 10.8 Hz, 1H), 4.85 (d, *J* = 11.5 Hz, 1H), 4.69 (d, *J* = 12.0 Hz, 1H), 4.66 (d, *J* = 11.4 Hz, 1H), 4.58 (d, *J* = 12.1 Hz, 1H), 4.53 (d, *J* = 10.9 Hz, 1H), 4.13 (dd, *J* = 9.1, 3.2 Hz, 1H), 4.01 (t, *J* = 9.3 Hz, 1H), 3.84–3.76 (m, 2H), 3.63 (ddd, *J* = 9.5, 4.6, 2.8 Hz, 1H). ^13^C {^1^H} NMR (CDCl_3_, 125 MHz): δ 165.9, 138.4, 138.3, 138.0, 133.2, 130.2, 130.1, 128.5, 128.4, 128.1, 127.9, 127.8, 127.74, 126.2, 118.7, 108.6, 107.1, 78.6, 75.2, 75.0, 73.9, 73.5, 72.0, 69.8, 69.7. HRMS (ESI/Q-TOF) *m*/*z*: [M + Na]^+^ calcd for C_38_H_37_NNaO_6_, 626.2519; found, 626.2533.

##### 2-(2′-3′,4′,6′-Tetra-*O*-benzoyl-α-d-mannopyranosyl)-pyrrole (**4e**)

The compound **4e** was prepared according to the general procedure A starting from methyl 3,4,6-tri-*O*-benzoyl mannopyranosyl 1,2-orthobenzoate **15b** (250 mg, 0.4 mmol), pyrrole (60 μL, 0.82 mmol), and BF_3_·Et_2_O (30 μL, 0.2 mmol) at −30 °C (2 h). After workup (Et_3_N, 2 mL), the residue was purified by silica gel column chromatography (hexane/ethyl acetate, 8:2) to afford **4e** (200 mg, 77%). ^1^H NMR (CDCl_3_, 400 MHz): δ 8.61 (s, 1H), 8.14–8.12 (m, 2H), 8.08–8.03 (m, 2H), 7.97–7.83 (m, 4H), 7.65–7.53 (m, 2H), 7.52–7.24 (m, 10H), 6.93–6.81 (m, 1H), 6.74–6.61 (m, 1H), 6.36 (dd, *J* = 3.2, 1.8 Hz, 1H), 6.34–6.29 (m, 1H), 6.21 (t, *J* = 10.0 Hz, 1H), 5.94 (dd, *J* = 10.0, 3.2 Hz, 1H), 5.40 (bs, 1H), 4.72 (dd, *J* = 12.2, 2.6 Hz, 1H), 4.49 (dd, *J* = 12.2, 4.1 Hz, 1H), 4.10 (ddd, *J* = 10.0, 4.1, 2.6 Hz, 1H). ^13^C {^1^H} NMR (CDCl_3_, 125 MHz): δ 166.4, 166.1, 165.6, 165.5, 133.6, 133.5, 133.4, 133.3, 130.0, 129.9, 129.8, 129.6, 129.1, 129.0, 128.7, 128.6, 128.5, 125.1, 119.5, 109.1, 109.0, 73.2, 71.7, 71.5, 70.1, 67.9, 63.0. HRMS (ESI/Q-TOF) *m*/*z*: [M + H]^+^ calcd for C_38_H_32_NO_9_, 646.2071; found, 646.2067.

##### 1,3-Dimethyl-5-(2′,3′,4′,6′-tetra-*O*-acetyl-β-d-glucopyranosyl)-4,4-difluoro-4-bora-3a,4a-diaza-*s*-indacene (**12a**)

According to general procedure B, a solution of C2-glucosylpyrrole **4a** (100 mg, 0.25 mmol) and 3,5-dimethylpyrrole-2-carbaldehyde **11** (147 mg, 0.3 mmol) in anhydrous CH_2_Cl_2_ (5 mL) was reacted with POCl_3_ (70 mL, 0.75 mmol). The crude was treated with triethylamine (0.35 mL, 2.5 mmol) and BF_3_·Et_2_O (0.19 mL, 1.5 mmol). The residue was purified by flash chromatography (hexane/ethyl acetate 6:4) to give derivative **12a** as a red solid (106 mg, 77%) along with 1,3,5,7-tetramethyl-4,4-difluoro-4-bora-3a,4a-diaza-*s*-indacene **18** (1.9 mg, 5%). Data for **12a**: [α]_D_^25^, +545.8 (*c* 0.36, CHCl_3_); Mp 114–116 °C; ^1^H NMR (CDCl_3_, 400 MHz): δ 7.11 (s, 1H), 6.84 (d, *J* = 4.1 Hz, 1H), 6.48 (d, *J* = 4.1 Hz, 1H), 6.15 (s, 1H), 5.37 (m, 2H), 5.19 (t, *J* = 9.4 Hz, 1H), 5.01 (d, *J* = 9.3 Hz, 1H), 4.24 (dd, *J* = 12.4, 4.7 Hz, 1H), 4.11 (dd, *J* = 12.3, 2.3 Hz), 3.90 (ddd, *J* = 10.0, 4.7, 2.2 Hz, 1H), 2.56 (s, 3H), 2.23 (s, 3H), 2.03 (s, 3H), 2.01 (s, 3H), 1.98 (s, 3H), 1.83 (s, 3H). ^13^C {^1^H} NMR (CDCl_3_, 125 MHz): δ 170.8, 170.2, 169.7, 169.6, 164.1, 148.3, 146.3, 136.8, 133.0, 126.6, 125.0, 121.9, 115.4, 74.6, 73.0, 71.0, 68.6, 62.2, 20.8, 20.7, 20.6, 15.3, 11.4. ^19^F NMR (CDCl_3_, 376 MHz): δ −142.03 (dq, *J* = 103.5, 33.4 Hz), −145.48 (dq, *J* = 103.7, 32.3 Hz). ^11^B NMR (CDCl_3_, 128 MHz): δ 0.73 (t, *J* = 32.7 Hz). HRMS (ESI/Q-TOF) *m*/*z*: [M + NH_4_]^+^ calcd for C_25_H_33_BF_2_N_3_O_9_, 568.2277; found, 568.2293; [M + Na]^+^ calcd for C_25_H_29_BF_2_N_2_NaO_9_, 573.1831; found, 573.1841. Data for **18**(56): ^1^H NMR (CDCl_3_, 400 MHz): δ 7.01 (s, 1H), 6.02 (s, 2H), 2.51 (s, 6H), 2.22 (s, 6H).

##### 1,3-Dimethyl-5-(2′,3′,4′,6′-tetra-*O*-acetyl-α-d-mannopyranosyl)-4,4-difluoro-4-bora-3a,4a-diaza-*s*-indacene (**12b**)

Compound **12b** was prepared according to general procedure B: compound **4b** (55.4 mg, 0.14 mmol) and 3,5-dimethylpyrrole-2-carbaldehyde **11** (20.6 mg, 0.17 mmol) in anhydrous CH_2_Cl_2_ (3 mL) were reacted with POCl_3_ (39 μL, 0.42 mmol) during 15 min. The crude was treated with triethylamine (77 μL, 0.56 mmol) and BF_3_·Et_2_O (106 μL, 0.84 mmol). The residue was purified by flash chromatography (toluene/ethyl acetate 8:2) to give derivative **12b** as a red solid (57.7 mg, 75%) along with 1,3,5,7-tetramethyl-4,4-difluoro-4-bora-3a,4a-diaza-*s-*indacene (**18**). Data for **12b**: [α]_D_^25^, +434.2 (*c* 0.1, CHCl_3_); Mp 118–120 °C; ^1^H NMR (CDCl_3_, 400 MHz): δ 7.16 (s, 1H), 6.89 (d, *J* = 4.0 Hz, 1H), 6.71 (d, *J* = 4.0 Hz, 1H), 6.20 (s, 1H), 5.80 (t, *J* = 3.3 Hz, 1H), 5.46 (dd, *J* = 8.5, 3.0 Hz, 2H), 5.41 (dd, *J* = 8.7, 7.6 Hz, 1H), 4.46 (dd, *J* = 12.2, 4.5 Hz, 1H), 4.25 (dd, *J* = 12.2, 3.3 Hz, 1H), 3.84 (ddd, *J* = 7.8, 4.5, 3.3 Hz, 1H), 2.60 (s, 3H), 2.28 (s, 3H), 2.13 (s, 3H), 2.09 (s, 3H), 2.08 (s, 3H), 2.04 (s, 3H). ^13^C {^1^H} NMR (CDCl_3_, 125 MHz): δ 171.2, 170.6, 170.4, 169.7, 165.7, 146.6, 137.4, 133.8, 129.2, 128.4, 125.8, 124.6, 122.6, 116.5, 72.3, 70.8, 70.4, 69.1, 66.9, 62.1, 62.0, 21.1, 21.0, 20.90, 15.6, 11.6. ^19^F NMR (CDCl_3_, 376 MHz): δ −140.52 (dq, *J* = 99.0, 32.0 Hz), −149.48 (dq, *J* = 96.7, 31.1 Hz). ^11^B NMR (CDCl_3_, 128 MHz): δ −0.13 (t, *J* = 31.7 Hz). HRMS (ESI/Q-TOF) *m*/*z*: [M + NH_4_]^+^ calcd for C_25_H_33_BF_2_N_3_O_9_, 568.2277; found, 568.2290.

##### 1,3-Dimethyl-5-(2′,3′,6′,2″,3″,4″6″-Hepta-*O*-acetyl-β-d-maltopyranosyl)-4,4-difluoro-4-bora-3a,4a-diaza-*s*-indacene (**12c**)

According to general procedure B, a solution of C2-maltosylpyrrole **4c** (200 mg, 0.15 mmol) and 3,5-dimethylpyrrole-2-carbaldehyde **11** (22 mg, 0.175 mmol) in anhydrous CH_2_Cl_2_ (5 mL) was reacted with POCl_3_ (40 μL, 0.44 mmol). The crude was treated with triethylamine (80 μL, 0.58 mmol) and BF_3_·Et_2_O (110 μL, 0.87 mmol). The residue was purified by flash chromatography (hexane/ethyl acetate 3:7) to give derivative **12c** as an orange solid (97 mg, 83%). [α]_D_^25^, +166.2 (*c* 0.1, CHCl_3_); Mp 139–141 °C; ^1^H NMR (CDCl_3_, 400 MHz): δ 7.12 (s, 1H), 6.84 (d, *J* = 4.1 Hz, 1H), 6.43 (d, *J* = 4.1 Hz, 1H), 6.18 (s, 1H), 5.43–5.39 (m, 2H), 5.37 (dd, *J* = 10.5, 9.4 Hz, 1H), 5.28 (t, *J* = 9.4 Hz, 1H), 5.13–5.00 (m, 2H), 4.88 (dd, *J* = 10.5, 4.1 Hz, 1H), 4.46 (dd, *J* = 12.3, 2.4 Hz, 1H), 4.27–4.21 (m, 2H), 4.10 (dd, *J* = 9.8, 8.5 Hz, 1H), 4.04 (dd, *J* = 12.5, 2.3 Hz, 1H), 3.98 (ddd, *J* = 10.3, 3.7, 2.3 Hz, 1H), 3.92 (ddd, *J* = 9.7, 4.5, 2.4 Hz, 1H), 2.59 (s, 3H), 2.26 (s, 3H), 2.10 (s, 3H), 2.09 (s, 3H), 2.07 (s, 3H), 2.02 (s, 3H), 2.01 (s, 3H), 2.00 (s, 3H), 1.84 (s, 3H). ^13^C {^1^H} NMR (CDCl_3_, 125 MHz): δ 170.8, 170.8, 170.7, 170.2, 169.9, 169.6, 164.4, 148.5, 146.3, 137.0, 133.1, 126.5, 124.9, 122.0, 122.0, 115.0, 95.8, 76.4, 73.4, 72.7, 71.7, 70.2, 69.6, 68.6, 68.2, 63.4, 61.6, 21.1, 21.0, 20.8, 20.7, 20.6, 15.4, 11.5. ^19^F NMR (CDCl_3_, 376 MHz): δ −142.24 (dq, *J* = 103.9, 33.3 Hz), −145.48 (dq, *J* = 103.7, 32.0 Hz). ^11^B NMR (CDCl_3_, 128 MHz): δ 0.71 (t, *J* = 32.7 Hz). HRMS (ESI/Q-TOF) *m*/*z*: [M + NH_4_]^+^ calcd for C_37_H_49_BF_2_N_3_O_17_, 856.3123; found, 856.3116.

##### 1,3-Dimethyl-5-(2′-benzoyl-3′,4′,6′-tri-*O*-benzyl-α-d-mannopyranosyl)-4,4-difluoro-4-bora-3a,4a-diaza-*s*-indacene (**12d**)

Following the general procedure B, a solution of compound **4d** (161 mg, 0.27 mmol) and 3,5-dimethylpyrrole-2-carbaldehyde **11** (28 mg, 0.225 mmol) in anhydrous CH_2_Cl_2_ (7 mL) was treated with POCl_3_ (63 μL, 0.67 mmol). After stirring for 12 h, triethylamine (0.31 mL, 2.25 mmol) and BF_3_·Et_2_O (0.29 mL, 2.25 mmol) were added. The residue was purified by flash chromatography (hexane/ethyl acetate 9:1) to give BODIPY **12d** as an orange solid (133 mg, 65%). [α]_D_^25^, +1854.2 (*c* 0.05, CHCl_3_); Mp 98–100 °C; ^1^H NMR (CDCl_3_, 400 MHz): δ 8.01 (m, 2H), 7.53–7.21 (m, 18H), 7.08 (s, 1H), 6.71 (d, *J* = 4.1 Hz, 1H), 6.45 (d, *J* = 4.0 Hz, 1H), 6.20 (d, *J* = 2.9 Hz, 1H), 6.14 (s, 1H), 5.25 (s, 1H), 4.94–4.85 (m, 3H), 4.65–4.58 (m, 3H), 4.16 (t, *J* = 9.4 Hz, 1H), 4.04 (dd, *J* = 9.3, 3.0 Hz, 1H), 3.98 (dd, *J* = 11.4, 3.9 Hz, 1H), 3.87 (dd, *J* = 11.3, 1.7 Hz, 1H), 3.75 (ddd, *J* = 9.6, 3.9, 1.7 Hz, 1H), 2.60 (s, 3H), 2.24 (s, 3H). ^13^C {^1^H} NMR (CDCl_3_, 125 MHz): δ 165.2, 162.3, 152.1, 145.1, 138.99, 138.8, 138.0, 136.0, 132.9, 130.3, 130.0, 128.5, 128.5, 128.4, 128.0, 127.7, 127.5, 127.2, 124.9, 121.2, 116.7, 81.7, 79.9, 75.3, 74.2, 74.0, 73.6, 71.3, 69.9, 69.8, 69.5, 15.3, 11.5. ^19^F NMR (CDCl_3_, 376 MHz): δ −144.08 (dq, *J* = 105.0, 32.3 Hz), −146.48 (dq, *J* = 103.2, 33.7 Hz). ^11^B NMR (CDCl_3_, 128 MHz): δ 0.77 (t, *J* = 33.4 Hz). HRMS (ESI/Q-TOF) *m*/*z*: [M + NH_4_]^+^ calcd for C_45_H_47_BF_2_N_3_O_6_, 774.3528; found, 774.3481. [M + Na]^+^ calcd for C_45_H_43_BF_2_N_2_NaO_6_, 779.3082; found, 779.3075.

##### 1,3-Dimethyl-5-(2′,3′,4′,6′-tetra-*O*-benzoyl-α-d-mannopyranosyl)-4,4-difluoro-4-bora-3a,4a-diaza-*s*-indacene (**12e**)

Following the general procedure B, a solution of compound **4e** (210 mg, 0.32 mmol) and 3,5-dimethylpyrrole-2-carbaldehyde **11** (48 mg, 0.39 mmol) in anhydrous CH_2_Cl_2_ (15 mL) was treated with POCl_3_ (100 μL, 0.96 mmol). After stirring for 12 h, triethylamine (0.3 mL, 2 mmol) and BF_3_·Et_2_O (0.4 mL, 2.93 mmol) were added. The residue was purified by flash chromatography (hexane/ethyl acetate 8:2) to give BODIPY **12e** as a red solid (143 mg, 56%). [α]_D_^25^, +1153.4 (*c* 0.06, CHCl_3_); Mp 110–112 °C; ^1^H NMR (CDCl_3_, 400 MHz): δ 8.11 (m, 4H), 7.92 (m, 4H), 7.61–7.30 (m, 12H), 7.21 (s, 1H), 7.05 (s, *J* = 4.0 Hz, 1H), 7.00 (d, *J* = 4.0 Hz, 1H), 6.36 (t, *J* = 2.9 Hz, 1H), 6.28 (t, *J* = 9.1 Hz, 1H), 6.21 (s, 1H), 5.99 (dd, *J* = 9.3, 3.1 Hz, 1H), 5.76 (d, *J* = 2.8 Hz, 1H), 4.79 (dd, *J* = 12.1, 3.3 Hz, 1H), 4.63 (dd, *J* = 12.1, 3.8 Hz, 1H), 4.26 (dt, *J* = 8.9, 3.5 Hz, 1H), 2.60 (s, 3H), 2.30 (s, 3H). ^13^C {^1^H} NMR (CDCl_3_, 125 MHz): δ 166.5, 166.4, 166.0, 165.6, 165.3, 146.6, 146.4, 137.5, 134.1, 133.5, 133.4, 132.9, 130.4, 130.2, 130.0, 129.9, 129.6, 129.3, 129.1, 128.6, 128.5, 128.3, 125.8, 124.6, 122.8, 117.1, 117.0, 72.2, 72.0, 71.9, 70.2, 67.5, 63.3, 15.6, 11.6. ^19^F NMR (CDCl_3_, 376 MHz): δ −141.17 (dq, *J* = 101.5, 32.2 Hz), −150.98 (dq, *J* = 101.1, 30.6 Hz). ^11^B NMR (CDCl_3_, 128 MHz): δ 0.89 (t, *J* = 31.5 Hz). HRMS (ESI/Q-TOF) *m*/*z*: [M + NH_4_]^+^ calcd for C_45_H_41_BF_2_N_3_O_9_, 816.2906; found, 816.2885. [M + Na]^+^ calcd for C_45_H_37_BF_2_N_2_NaO_9_, 821.2460; found, 821.2453.

### Gram-Scale Synthesis of BODIPY **12a**

For 2,3,4,6-tetra-*O*-acetyl-α,β-d-glucopyranose, 1,2,3,4,6-penta-*O*-acetyl-α,β*-*d-glucopyranose (4g, 10.2 mmol) was dissolved in ethyl acetate (150 mL) and DMSO (15 mL); then, aminoethanol (0.62 mL, 10.2 mmol) was added, and the mixture was stirred at r.t. until the reaction was complete (1 h). The crude was diluted with ethyl acetate and washed several times with brine. The organic layer was dried over MgSO_4_, filtered, and concentrated *in vacuo*. The residue was purified by column chromatography on silica gel (hexane/ethyl acetate, 1:1) to afford 2,3,4,6-tetra-*O*-acetyl-α,β-d-glucopyranose^47^ (2.5 g, 70%).

#### 2,3,4,6-Tetra-*O*-acetyl-α-d-glucopyranosyl-trichloroacetimidate (**14a**)

To a stirred solution of 2,3,4,6-tetra-*O*-acetyl-α,β-d*-*glucopyranose (2.42 g, 6.95 mmol) in dry CH_2_Cl_2_ (25 mL) cooled at 0 °C were added dropwise 1,8-diazabicyclo[5.4.0]undec-7-ene (0.84 mL, 5.6 mmol) and trichloroacetonitrile (2.78 mL, 27.8 mmol). The reaction mixture was stirred at r.t. for 2 h, the solvent was evaporated under reduced pressure, and the crude was subjected to column chromatography (hexane/ethyl acetate, 85:15) to obtain **14a**(47) (3.24 g, 95%).

#### 2-(2′,3′,4′,6′-Tetra-*O*-acetyl-β-d-glucopyranosyl)-pyrrole (**4a**)

Compound **4a** (1.5 g, 81%) was prepared following general procedure A, starting from 2,3,4,6-tetra-*O*-acetyl-α-d-glucopyranosyl-trichloroacetimidate **14a** (2.3 g, 4.7 mmol), pyrrole (1.4 mL, 20.3 mmol), and BF_3_·Et_2_O (260 μL, 2 mmol) at −78 °C (30 min).

#### 1,3-Dimethyl-5-(2′,3′,4′,6′-tetra-*O*-acetyl-β-d-glucopyranosyl)-4,4-difluoro-4-bora-3a,4a-diaza-*s*-indacene (**12a**)

According to general procedure B, a solution of C2-glucosylpyrrole **4a** (1.18 g, 2.96 mmol) and 3,5-dimethylpyrrole-2-carbaldehyde (437 mg, 3.55 mmol) in anhydrous CH_2_Cl_2_ (30 mL) was reacted with POCl_3_ (0.83 mL, 8.88 mmol). The reaction crude was treated with triethylamine (4.1 mL, 29.6 mmol) and BF_3_·Et_2_O (2.25 mL, 17.8 mmol). The residue was purified by flash chromatography (hexane/ethyl acetate 6:4) to give derivative **12a** as a red solid (1.19 g, 73%) along with 1,3,5,7-tetramethyl-4,4-difluoro-4-bora-3a,4a-diaza-*s*-indacene **18** (31 mg, 7%). The spectroscopic and analytical data of **12a** from the gram-scale reaction were consistent with those above for this derivative, obtained in the 0.25 mmol-scale experiment.

### Photophysical Properties

The photophysical properties were registered from diluted solutions (around 2 × 10^–6^ M), prepared by adding the corresponding solvent to the residue from the adequate amount of a concentrated stock solution in methanol, after vacuum evaporation of this solvent. All organic solvents were of spectroscopic grade, and water was of Milli-Q grade. UV–vis absorption and fluorescence spectra were recorded on a Varian model CARY 4E spectrophotometer and an Edinburgh Instruments spectrofluorometer (model FLSP 920), respectively. Fluorescence quantum yields (Φ_fl_) were obtained using commercial BODIPYs (PM546, Φ_fl_ = 0.85 in ethanol, for the nonhalogenated dyes, and PM567, Φ_fl_ = 0.84 in ethanol, for the halogenated dyes) and cresyl violet (Φ_fl_ = 0.54 in methanol, for the π-extended dye **30**), as references, from corrected spectra (detector sensibility to the wavelength). The values were corrected by the refractive index of the solvent. Radiative decay curves were registered with the time-correlated single-photon counting technique as implemented in the aforementioned spectrofluorometer. Fluorescence emission was monitored at the maximum emission wavelengths after excitation by means of a Fianium pulsed laser (time resolution of picoseconds) with a tunable wavelength. The fluorescence lifetime (τ_fl_) was obtained after the deconvolution of the instrumental response signal from the recorded decay curves by means of an iterative method. The goodness of the exponential fit was controlled by statistical parameters (chi-square, Durbin–Watson, and the analysis of the residuals).

Nanosecond transient absorption spectra (ns-TAS) were recorded on an LP 980 laser flash photolysis spectrometer (Edinburgh Instruments). Samples were excited by a nanosecond pulsed laser (Nd:YAG laser, Lotis TII 2134) operating at 1 Hz and a pulse width of ≥7 ns, coupled to an OPO, which allows the selection of the excitation wavelength. The transient signals were recorded on a single detector (PMT R928P) oscilloscope for kinetic traces and an ICCD detector DH320T TE cooled (Andor Technology) for time-resolved spectra. Samples were measured aerated and deaerated with nitrogen or oxygen for ca. 15 min before each measurement.

The photoinduced production of singlet oxygen (^1^O_2_) was determined by direct measurement of the luminescence at 1276 nm with an NIR detector integrated in the aforementioned spectrofluorometer (InGaAs detector, Hamamatsu G8605-23). The ^1^O_2_ signal was registered in a front configuration (front face), 40 and 50° to the excitation and emission beams, respectively, and leaned 30° to the plane formed by the direction of incidence and registration in cells of 1 cm. The signal was filtered by a low cutoff of 850 nm. The ^1^O_2_ generation quantum yield (Φ_Δ_) was determined using the following equation:
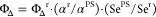
where Φ_Δ_^r^ is the quantum of ^1^O_2_ generation for the used reference (2,6-diiodo-3,5-dimethyl-8-methylthioBODIPY, MeSBDP) being 0.91 in chloroform. Factor α = 1 – 10^–Abs^ corrects the different amounts of photons absorbed by the sample (α^PS^) and reference (α^R^). Factor Se is the intensity of the ^1^O_2_ phosphorescence signal of the sample (Se^PS^) and the reference (Se^r^) at 1276 nm. ^1^O_2_ quantum yields were averaged from at least five concentrations between 10^–6^ and 10^–5^ M.

### Photostability

The photostability of the dyes was evaluated from concentrated water solutions (millimolar) contained in 0.1 cm optical-path quartz cells to allow for the minimum solution volume (0.3 mL) to be excited. The liquid solutions were pumped transversely with 5 mJ, 8 ns full width at half-maximum (fwhm) pulses from the third harmonic (355 nm) of a Q-switched Nd:YAG laser (Lotis TII2134) at a 10 Hz repetition rate. The exciting pulses were line-focused onto the cell using a combination of positive and negative cylindrical lenses (*f* = 15 cm and *f* = −15 cm, respectively) perpendicularly arranged. The lateral faces of cells were grounded, whereupon no laser oscillation was obtained. Photostability was determined by monitoring the decrease in laser-induced fluorescence intensity after 50,000 pump pulses. The emitted light was collected in a front-face configuration and integrated by a boxcar averager (Stanford Research, model 250) before digitization and computer analysis. The estimated error in photostability measurement was 10%.

### Delayed Spectroscopy

Aerated solutions at room temperature of the new dyes contained in 1 cm optical-path rectangular quartz cells were transversally pumped with intense laser pulses from the second harmonic (532 nm) of a Nd:YAG laser (Lotis TII, LS-2147) at a 10 Hz repetition rate. The time-gated emission upon laser photoexcitation, analyzed perpendicularly to the input radiation, was focused onto a spectrograph (Kymera 193i-A, Andor Technologies) coupled to an intensified CCD camera (iStar, Andor Technologies). This camera enables gate widths ranging from nanoseconds up to seconds, and its opening can be delayed in a controlled way with respect to the incoming pump laser pulse. Neither long-pass filters nor band-pass filters were used to remove the excitation laser since we have verified that these filters, especially long-pass ones, under drastic pump conditions, exhibited their own fluorescence and/or phosphorescence emission, which could lead to misunderstanding the experimental results. Each spectrum is the average of at least 200 scans recorded with a gate time of 50 μs. The experiments were usually carried out at an excitation energy fluence of 5 mJ/cm^2^, which was varied from 1 up to 25 mJ/cm^2^ to determine the dependence of the delayed fluorescence on the laser fluence. A solution volume of 3 cm^3^ was used in order to avoid (or at least, to reduce) the risk of photobleaching the sample during the experiments. This experimental setup allowed to carry out the projected measurements even under adverse conditions but avoided to determine properly the efficiency of the delayed emission.

## Data Availability

The data underlying this study are available in the published article and its Supporting Information

## Supporting Information Available

Experimental procedures; copies of ^1^H, ^13^C NMR, and photophysical data of all compounds; absorption and steady-state fluorescence spectra, transient absorption spectra, and time-dependent emission spectra of selected representative BODIPY-carbohydrate conjugates (PDF)
