# Supplementary material for: De Novo Access to BODIPY C-Glycosides as Linker-Free Nonsymmetrical BODIPY-Carbohydrate Conjugates
Source: J Org Chem. 2024 Mar 4;89(6):4042–55. doi: 10.1021/acs.joc.3c02907 (PMC10949249; doi:10.1021/acs.joc.3c02907)
Supplement: Supplementary file 1 — jo3c02907_si_001.pdf [file jo3c02907_si_001.pdf]

Supporting Information

De novo access to BODIPY *C*-glycosides as linker-free non-symmetrical BODIPY carbohydrate conjugates

Clara Uriel,[$]* Dylan Grenier,[$] Florian Herranz,[$] Natalia Casado,[§] Jorge Bañuelos,[§]* Esther Rebollar,[¶] Inmaculada Garcia-Moreno,[¶] Ana M. Gomez,[$] and J. Cristobal López[$]*

[$] *Instituto de Química Orgánica General,* IQOG-CSIC*, Juan de la Cierva 3, 28006, Madrid, Spain.*

[§] *Departamento de Química Física. Universidad del Pais Vasco,* UPV-EHU*, Apartado 644, 48080, Bilbao, Spain.*

[¶] *Instituto de Química y Física Blas Cabrera, CSIC, Serrano 119, 28006, Madrid, Spain.*

Table of Contents

1. General procedures……S2
2. Synthetic procedures and compound characterization……S3
3. **Table S1**. Photophysical properties of the non-halogenated glyco-BODIPYs……S14
4. **Table S2**. Photophysical properties of the halogenated glyco-BODIPYs……S14
5. **Figure S1**. Absorption and normalized fluorescence spectra of non-halogenated and unprotected glycol BODIPYs……S15
6. **Figure S2**. Nanosecond-resolved transient absorption spectra……S15
7. **Figure S3**. Time-dependent emission spectra……S16
8. Copies of 1H, 13C {1H}, 19F, 11B NMR Spectra……S16

1. General procedures

General procedure A. Preparation of C2-glycosylated pyrroles. The corresponding glycosyl donor (1 equiv.) and pyrrole (5 equiv.) were dissolved in anhydrous CH_2Cl_2 and cooled to indicated temperature. Then $BF_3{\cdot}Et_2O$ (0.5 equiv.) was added. The reaction mixture was stirred until TLC showed complete disparition of the glycosyl donor, then Et_3N was added (3.5 equiv.), after stirring 5 min. the crude material was concentrated *in vacuo*. The residue was purified by silica column chromatography.

General procedure B. Preparation of C-glycosyl BODIPYs. A solution of the corresponding C2-glycosylpyrrole (1 equiv.) and 3,5-dimethylpyrrole-2-carbaldehyde **11** (1.2 equiv.) in anhydrous CH_2Cl_2 (5–20 mL/mmol) was cooled at 0 °C. Then $POCl_3$ (3 equiv.) was added dropwise. The solution was stirred at 0 °C for 15 min and then at room temperature (r.t.) overnight. The reaction mixture was recooled at 0 °C, triethylamine (10 equiv.) and $BF_3{\cdot}Et_2O$ (6 equiv.) were added dropwise and stirred at 50 °C for another 3 h. The solution was diluted with CH_2Cl_2 (50 mL/mmol), washed with water (2x50 mL/mmol), $NaHCO_3$ (50 mL/mmol), dried over Na_2SO_4, and concentrated under reduced presure. The residue was purified by silica column chromatography.

General procedure C. Preparation of B(CN)$_2$-BODIPYs from BF$_2$-BODIPYs. A solution of the corresponding BF_2-BODIPY (1 equiv.) in dry CH_2Cl_2 was cooled to 0 °C and treated with $BF_3{\cdot}Et_2O$ (0.4 equiv.). The mixture was stirred at r.t. for 5-10 min, then TMSCN (5-10 equiv.) was added and the reaction kept under these conditions for 30-120 min, then neutralized with saturated aqueous $NaHCO_3$ solution, diluted with CH_2Cl_2 and washed twice with water. The organic phase was dried over $MgSO_4$, concentrated and purified on a silica gel column.

General procedure D. NaOMe mediated deacetylation. A solution of the corresponding BODIPY (1 equiv.) in a CH_2Cl_2:MeOH (1:1) mixture (35 mL/mmol), was treated with NaOMe (2 equiv./Ac). After stirring at r.t. (1–12 h) the solution was neutralized with dry ice (carbon dioxide), then filtered and concentrated. The residue was purified by column chromatography on silica gel.

General procedure E. Acid mediated deacetylation. A solution of the corresponding BODIPY (1 equiv.) in anhydrous THF was treated with 1.25 M HCl/MeOH solution (10 equiv./Ac). After stirring at the indicated temperature (3–12 h) the solution was quenched with Et_3N until neutral pH and concentrated. The residue was purified using column chromatography.

General procedure F. Preparation of halogenated BODIPYs. To a stirred solution of the appropriate BODIPY (1 equiv.) in CH_2Cl_2 at 0 °C was added *N*-iodosuccinimide or *N*-bromosuccinimide (1.2 equiv.) and $BF_3{\cdot}Et_2O$ (0.5 equiv.). The mixture was left to stand at rt. After completion of the reaction monitored by TLC the crude was diluted with CH_2Cl_2, quenched with saturated sodium bicarbonate and 10% sodium thiosulfate solutions. The organic layer was extracted with CH_2Cl_2, washed with water, and dried. The solvents were removed, and the residue was purified by column chromatography on a silica gel column.

General procedure G. Knoevenagel reaction. To a solution of the corresponding BODIPY (1 equiv.) and benzaldehyde (2–4 equiv) in dry DMF or CH_3CN were added piperidinium acetate (2 equiv.) at r.t. (2-24 h). The resulting crude mixture was then partitioned between AcOEt and water, and the aqueous layer was reextracted. The organic phase was dried, concentrated and then chromatographed over silica gel flash column**.**

2. Synthetic procedures and compound characterization

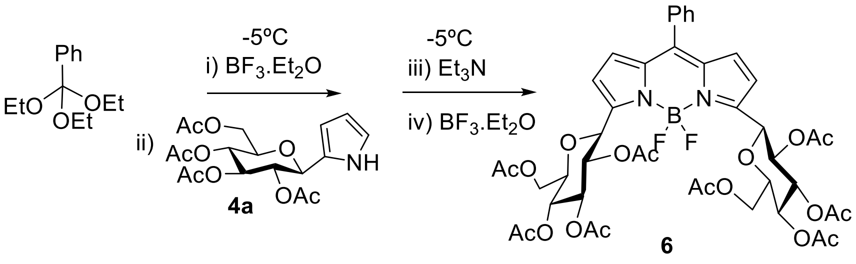

Scheme S1. Synthesis of BODIPY **6**

BODIPY **(6).** A solution of triethylorthobenzoate (38 μL, 0.17 mmol) in anhydrous CH_2Cl_2 (1 mL) was treated with $BF_3{\cdot}Et_2O$ (25 μL, 0.2 mmol) at -5 °C. The resulting solution was stirred for 30 min and then C-2 glucosylpyrrol **4a** (146 mg, 0.37 mmol) was added, the mixture was stirred at -5 °C for additional 3 h, then Et_3N (0.2 mL, 1.44 mmol) and $BF_3{\cdot}Et_2O$ (0.21 mL, 1.67 mmol) were added dropwise and stirred at room temperature (r.t.) for another 12 h. The crude was neutralized with saturated aqueous $NaHCO_3$ solution, diluted with CH_2Cl_2 and washed twice with water. The organic phase was dried over $MgSO_4$, concentrated and purified on a silica gel column (hexane/ethyl acetate 1:1) to give Bodipy **6** as a red solid (6.4 mg, 4%) along with unreacted glycosylpyrrole **4a** (45.6 mg, 31%). Data for BODIPY **6**[1]: ^1H NMR ($CDCl_3$, 500 MHz) δ 7.62 – 7.45 (m,

[1] Sollert, C.; Kocsi, D.; Jane, R. T.; Orthaber, A.; Borbas, K. E. *C*-glycosylated pyrroles and their application in dipyrromethane and porphyrin synthesis. *J. Porphyrins Phthalocyanines* **2021**, *25*, 741–755

5H), 6.89 (d, *J* = 4.5 Hz, 2H), 6.67 (d, *J* = 4.5 Hz, 2H), 5.49 – 5.39 (m, 4H), 5.28 – 5.14 (m, 4H), 4.29 (dd, *J* = 12.5, 4.7 Hz, 2H), 4.19 (dd, *J* = 12.5, 2.2 Hz, 2H), 4.02 (ddd, *J* = 10.1, 4.7, 2.2 Hz, 2H), 2.09 (s, 6H), 2.08 (s, 6H), 2.03 (s, 6H), 1.88 (s, 6H). $^{13}C\{^1H\}$ NMR ($CDCl_3$, 125 MHz) δ: 170.8, 170.1, 169.6, 154.1, 148.1, 135.2, 133.2, 132.1, 131.0, 130.5, 128.5, 118.0, 76.4, 74.3, 72.7, 70.6, 68.4, 62.1, 20.8, 20.7, 20.6, 20.5.

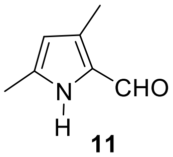

11

3,5-dimethylpyrrole-2-carbaldehyde **(11)**. A mixture of DMF (0.57 mL, 7.26 mmol) and $POCl_3$ (0.68 mL, 7.26 mmol) was stirred in an ice bath for 5 min. After being warmed to r. t., the mixture was stirred for 30 min. Then, a solution of 2,4-dimethylpyrrole (0.63 mL, 6.05 mmol) in anhydrous CH_2Cl_2 (20 mL) was added, the temperature was raised to 50 °C, and then stirred at that temperature for 1 h. The reaction mixture was cooled to r. t. and slowly neutralized with a 1M NaOH solution (40 mL) under ice-cold conditions. After being warmed to 50 °C, the reaction mixture was further stirred for 30 min and washed with water (2 × 50 mL). The organic layers were combined, dried, and evaporated under vacuum. The resulting crude product was further purified using column chromatography (hexane/ethyl acetate 9:1) to give 3,5-dimethylpyrrole-2-carbaldehyde **11**[2] as a solid (744 mg, 96%). ^1H NMR ($CDCl_3$, 400 MHz): δ 9.48 (s, 1H), 5.86 (bs, 1H), 2.32 (s, 3H), 2.29 (s, 3H).

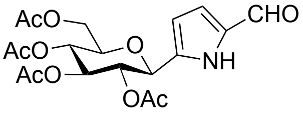

16

5-(2′,3′,4′,6′ Tetra-O-acetyl-β-D-glucopyranosyl)-pyrrole-2-carbaldehyde **(16)**. A dried flask under argon atmosphere was charged with anhydrous DMF (0.195 mL, 2.52 mmol), then $POCl_3$ (0.235 mL, 2.52 mmol) was added dropwise at 0 °C and the resulting solution was stirred for 15 min. Anhydrous 1,2-dichloroethane (10 mL) was then added. The Vilsmeier intermediate was transferred to a solution of 4-(2′,3′,4′,6′ tetra-*O*-acetyl-β-D-glucopyranosyl)-pyrrole (500 mg, 1.26 mmol) in dry 1,2-dichloroethane (10 mL). The mixture was heated to reflux for 15 min before being cooled to r.t.. Aqueous sodium acetate solution (45 g in 100 mL water) was then added. The mixture was refluxed overnight. The layers were separated, and the aqueous layer was washed three times with CH_2Cl_2. The combined organic layer was washed with saturated brine, dried over

---

[2] Wu, L.; Burgess, K. A new synthesis of symmetric boraindacene (BODIPY) dyes. *Chem. Commun.* **2008**, 4933–4935.

$MgSO_4$, and evaporated. The residue was purified by flash chromatography (hexane/ethyl acetate 75:25) to obtain **16**[1] 505 mg (94%). ^1H NMR ($CDCl_3$, 400 MHz): δ 10.10 (s, 1H), 9.48 (s, 1H), 6.87 (dd, *J* = 3.9, 2.5 Hz, 1H), 6.21 (dd, *J* = 3.9, 2.4 Hz, 1H), 5.35 – 5.29 (m, 1H), 5.19 (t, *J* = 9.6 Hz, 1H), 5.18 (t, *J* = 9.7 Hz, 1H), 4.61 (d, *J* = 9.9 Hz, 1H), 4.28 (dd, *J* = 12.5, 5.0 Hz, 2H), 4.14 (dd, *J* = 12.1, 2.6 Hz, 1H), 3.85 (ddd, *J* = 9.9, 5.0, 2.2 Hz, 2H), 2.07 (s, 3H), 2.04 (s, 3H), 2.00 (s, 3H), 1.90 (s, 4H). ^{13}C {^1H} NMR ($CDCl_3$, 125 MHz): δ: 179.5, 170.9, 170.5, 169.6, 169.1, 134.8, 133.1, 121.2, 109.8, 76.5, 73.9, 73.8, 71.3, 68.2, 62.2, 20.9, 20.8, 20.7, 20.6.

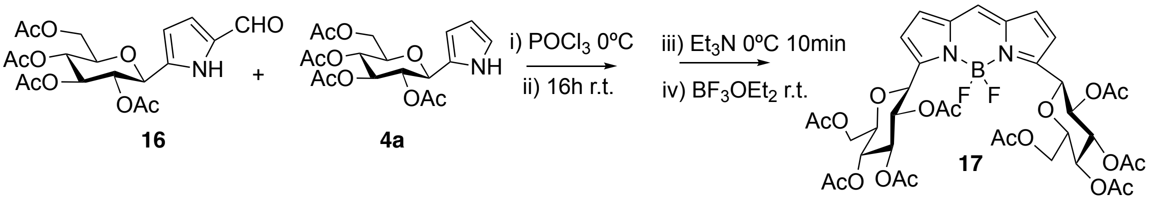

Scheme S2. Synthesis of BODIPY **17**

BODIPY **17**. A stirred solution of **16** (57 mg, 0.13 mmol) and **4a** (77 mg, 0.195 mmol) in anhydrous CH_2Cl_2 (5 mL) was cooled to 0 ˚C, then $POCl_3$ (39 µl, 0.42 mmol) was added dropwise. The resulting solution was stirred for 12 h at r.t., triethylamine (0.2 mL, 1.4 mmol) and BF_3·Et_2O (0.2 mL, 1.54 mmol) were added. After 12 h the solution was diluted with CH_2Cl_2 (40mL), washed with water (20 mL), $NaHCO_3$ (20 mL), dried over Na_2SO_4, and concentrated under reduced presure. The residue was purified by silica column chromatography (hexane/ethyl acetate 1:1) to give BODIPY **17** (17 mg, 15%) as an amorfous red solid. ^1H NMR ($CDCl_3$, 500 MHz) δ: 7.33 (s, 1H), 7.12 (d, *J* = 4.3 Hz, 2H), 6.68 (d, *J* = 4.4 Hz, 2H), 5.47 – 5.37 (m, 4H), 5.23 (t, *J* = 9.3 Hz, 2H), 5.08 (d, *J* = 9.3 Hz, 2H), 4.29 (dd, *J* = 12.6, 4.5 Hz, 2H), 4.16 (dd, *J* = 12.6, 2.1 Hz, 2H), 3.98 (ddd, *J* = 10.1, 4.6, 2.1 Hz, 2H), 2.09 – 2.08 (s, 6H), 2.07 – 2.07 (s, 6H), 2.03 (d, *J* = 1.0 Hz, 6H), 1.86 (d, *J* = 1.0 Hz, 6H). ^{13}C {^1H} NMR ($CDCl_3$, 125 MHz) δ: 170.9, 170.2, 169.8, 169.8, 155.5, 135.4, 132.0, 118.6, 74.3, 72.8, 70.9, 68.4, 62.2, 20.9, 20.8, 20.6. ^{19}F NMR ($CDCl_3$, 376 MHz) δ -137.85 (q, *J* = 32.5 Hz). ^{11}B NMR ($CDCl_3$, 128 MHz) δ: -0.25 (t, *J* = 31.9 Hz). HRMS (ESI/Q-TOF) m/z: $[M+NH_4]^+$ calcd for C_{37} H_{47} B F_2 N_3 O_{18} 870.2916; found 870.2948. $[M+Na]^+$ calcd for C_{37} H_{43} B F_2 N_2 Na O_{18} 875.2470; found 875.2505.

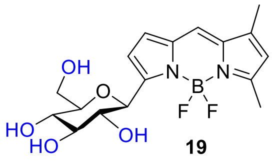

1,3-Dimethyl-5- (β-D-glucopyranosyl)- 4,4-difluoro-4-bora-3a,4a-diaza-s-indacene **19**. Compound **19** was prepared according to general procedure **D** from **12a** (200 mg, 0.36 mmol) and NaOMe

(78 mg, 1.45 mmol) in 12 mL CH_2Cl_2:MeOH (1:1) during 12 h. The residue was purified by flash chromatography (CH_2Cl_2:MeOH, 95:5) to give **19** as a brown solid (54 mg, 39%). $[\alpha]_D^{25}$ +289.8 (*c* 0.78, CH_3OH); Mp 144 – 146 °C; ^1H NMR (CD_3OD, 400 MHz): δ 7.54 (s, 1H), 7.05 (d, *J* = 4.2 Hz, 1H), 6.60 (d, *J* = 4.0 Hz, 1H), 6.30 (s, 1H), 4.74 (d, *J* = 9.8 Hz, 1H), 3.90 – 3.83 (m, 1H), 3.70 – 3.64 (m, 2H), 3.53 (t, *J* = 8.7 Hz, 1H), 3.49 – 3.38 (m, 2H), 2.56 (s, 3H), 2.32 (s, 3H). ^{13}C {^1H} NMR (CD_3OD, 125 MHz): δ 164.0, 153.7, 147.5, 137.6, 134.7, 128.3, 126.7, 122.4, 116.5, 82.3, 79.2, 76.0, 74.8, 71.8, 63.1, 15.1, 11.2.^{19}F NMR (CD_3OD, 376 MHz) δ -144.08 (dq, *J* =104.2, 32.3 Hz), -144.16 (dq, *J* = 104.2, 31.8 Hz). ^{11}B NMR (CD_3OD, 128 MHz) δ 0.83 (t, *J* = 32.5 Hz). HRMS (ESI/Q-TOF) m/z: $[M+NH_4]^+$ calcd for $C_{17}H_{25}BF_2N_3O_5$: 400.1853; found 400.1839; $[M+Na]^+$ calcd for $C_{17}H_{21}BF_2N_2NaO_5$: 405.1406; found 405.1388.

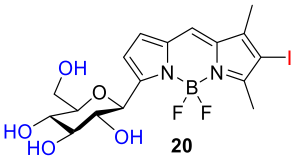

20

BODIPY **20**. Compound **20** was prepared according to general procedure **D** from **21** (95 mg, 0.15 mmol) and NaOMe (32 mg, 0.6 mmol) in 12 mL CH_2Cl_2:MeOH (1:1) during 12 h. The residue was purified by flash chromatography (CH_2Cl_2:MeOH 95:5) to give **20** as a brown solid (13 mg, 18%). This compound was also prepared started from **19** (65 mg, 0.17 mmol) in dioxane (5 mL) treated with *N*-iodosuccinimide (46 mg, 0.2 mmol) and $BF_3{\cdot}Et_2O$ (15 μL, 0.12 mmol) according to general procedure **F** (18 mg, 21%) during 12 h. Compound **20**: $[\alpha]_D^{25}$ +338.5 (*c* 0.1, $CHCl_3$); Mp 169 – 170 °C; ^1H NMR (CD_3OD, 400 MHz): δ 7.64 (s, 1H), 7.16 (d, *J* = 4.2 Hz, 1H), 6.67 (d, *J* = 4.2 Hz, 1H), 4.73 (d, *J* = 9.8 Hz, 1H), 3.87 (dd, *J* = 12.0, 1.7 Hz, 1H), 3.75 – 3.63 (m, 2H), 3.53 (t, *J* = 8.8 Hz, 1H), 3.48 – 3.42 (m, 2H), 2.60 (s, 3H), 2.28 (s, 3H). ^{13}C {^1H} NMR (CD_3OD, 125 MHz): δ 161.6, 156.3, 148.5, 136.0, 135.1, 130.7, 127.5, 117.8, 82.4, 79.8, 76.0, 74.8, 71.8, 63.0, 16.1, 13.8. ^{19}F NMR (CD_3OD, 376 MHz) δ −143.29 (dq, *J* = 102.6, 32.0 Hz, 1F), −144.15 (dq, *J* = 102.4, 32.5 Hz, 1F) ; ^{11}B NMR (CD_3OD, 128 MHz) δ 0.72 (t, *J* = 32.1 Hz). HRMS (ESI/Q-TOF) m/z: $[M+Na]^+$ calcd for $C_{17}H_{20}BF_2IN_2NaO_5$: 531.0373; found 531.0385.

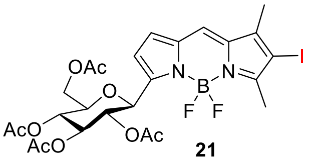

21

BODIPY **21**. Compound **21** was obtained from **12a** (416 mg, 0.76 mmol) in CH_2Cl_2 (7 mL) treated with *N*-iodosuccinimide (204 mg, 0.91 mmol) and $BF_3{\cdot}Et_2O$ (48 μL, 0.38 mmol) according to procedure **F**. Reaction time: 1 h. The residue was purified by flash chromatography (hexane/ethyl

acetate 7:3) to give **21** as a red solid (411 mg, 80%). $[\alpha]_D^{25}$ +309.1 (*c* 0.1, $CHCl_3$); Mp > 300 °C; ^1H NMR ($CDCl_3$, 400 MHz): δ 7.21 (s, 1H), 6.97 (d, *J* = 4.2 Hz, 1H), 5.43-5.35 (m, 2H), 5.21 (t, *J* = 9.5 Hz, 1H), 5.02 (d, *J* = 9.4 Hz, 1H), 4.27 (dd, *J* = 12.5, 4.7 Hz, 1H), 4.15 (dd, *J* = 12.5, 2.2 Hz, 1H), 3.92 (ddd, *J* = 10.1, 4.7, 2.2 Hz, 1H), 2.66 (s, 3H), 2.25 (s, 3H), 2.06 (s, 3H), 2.05 (s, 3H), 2.01 (s, 3H), 1.86 (s, 3H). ^{13}C {^1H} NMR ($CDCl_3$, 125 MHz): δ 170.9, 170.2, 169.8, 169.8, 162.9, 150.6, 147.9, 135.7, 133.3, 128.7, 125.4, 116.6, 76.4, 74.5, 73.0, 71.1, 68.6, 62.3, 20.9, 20.8, 20.7, 20.7, 16.3, 14.0.^{19}F NMR ($CDCl_3$, 376 MHz) δ −141.51 (dq, *J* = 99.6, 32.9 Hz, 1F), −145.22 (dq, *J* = 101.5, 31.9 Hz, 1F); ^{11}B NMR ($CDCl_3$, 128 MHz) δ 0.64 (t, *J* = 32.4 Hz). HRMS (ESI/Q-TOF) m/z: $[M+NH_4]^+$ calcd for $C_{25}H_{32}BF_2IN_3O_9$: 694.1243; found 694.1228.

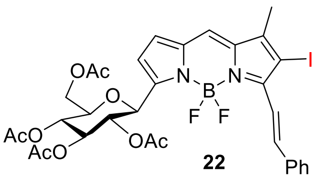

22

BODIPY **22**. Obtained from iodo derivative **21** (100 mg, 0.15 mmol) in CH_3CN (12 mL) treated with benzaldehyde (66 μL, 0.6 mmol) and piperidinium acetate (43 mg, 0.3 mmol) according to procedure **G** during 3 h. Purification: flash chromatography (hexane/ethyl acetate 7:3) to give **22** as a blue solid (34 mg, 32%). $[\alpha]_D^{25}$ +1421.4 (*c* 0.2, $CHCl_3$); Mp 55-60 °C; ^1H NMR ($CDCl_3$, 400 MHz): δ 8.23 (d, *J* = 16.7 Hz, 1H), 7.65 – 7.61 (m, 2H), 7.57 (d, *J* = 16.7 Hz, 1H), 7.39 (t, *J* = 7.3 Hz, 2H), 7.35 – 7.31 (m, 1H), 7.19 (s, 1H), 7.14 (s, 1H), 6.92 (d, *J* = 4.2 Hz, 1H), 6.55 (d, *J* = 4.3 Hz, 1H), 5.41 – 5.28 (m, 2H), 5.15 (t, *J* = 9.6 Hz, 1H), 5.04 (d, *J* = 9.5 Hz, 1H), 4.20 (dd, *J* = 12.5, 4.8 Hz, 1H), 4.09 (dd, *J* = 12.5, 2.2 Hz, 1H), 3.91 (ddd, *J* = 10.1, 4.9, 2.2 Hz, 1H), 2.22 (s, 3H), 1.99 (s, 3H), 1.97 (s, 2H), 1.95 (s, 3H), 1.80 (s, 3H). ^{13}C {^1H} NMR ($CDCl_3$, 125 MHz): δ 170.9, 170.3, 169.8, 154.6, 151.2, 148.4, 141.9, 136.4, 136.3, 134.0, 130.2, 129.1, 128.6, 128.1, 124.4, 118.3, 117.2, 76.4, 74.5, 73.0, 71.1, 68.7, 62.3, 20.9, 20.8, 20.7, 14.5. ^{19}F NMR ($CDCl_3$, 376 MHz) δ −137.57 (dq, *J* = 100.4, 33.7 Hz, 1F), −141.87 (dq, *J* = 98.8, 32.7 Hz, 1F); ^{11}B NMR ($CDCl_3$, 128 MHz) δ 0.92 (t, *J* = 33.2 Hz). HRMS (ESI/Q-TOF) m/z: $[M+NH_4]^+$ calcd for $C_{32}H_{36}BF_2IN_3O_9$: 782.1558; found 782.1531.

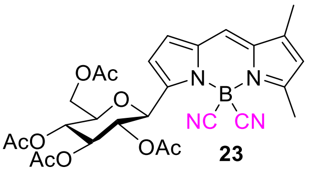

23

BODIPY **23**. Compound **23** was prepared according to general procedure **C** from **12a** (200 mg, 0.36 mmol) in CH_2Cl_2 (5 mL) treated with $BF_3·Et_2O$ (18 μL, 0.15mmol) and TMSCN (0.45 mL, 3.6 mmol). The reaction was kept under these conditions for 1 h. The residue was purified by flash chromatography (hexane/ethyl acetate 6:4 to 4:6) to give **23** as a red solid (201 mg, 99%). $[\alpha]_D^{21}$

+1095.4 (*c* 0.1, $CHCl_3$); Mp 109 – 110 °C; ^{1}H NMR ($CDCl_3$, 400 MHz): δ 7.29 (s, 1H), 7.02 (d, *J* = 4.2 Hz, 1H), 6.66 (d, *J* = 4.2 Hz, 1H), 6.39 (d, *J* = 1.2 Hz, 1H), 5.59 (dd, *J* = 10.1, 9.0 Hz, 1H), 5.41 (t, *J* = 9.2 Hz, 1H), 5.27 (t, *J* = 9.2 Hz, 1H), 5.10 (d, *J* = 10.1 Hz, 1H), 4.37 (dd, *J* = 12.6, 4.1 Hz, 1H), 4.24 (dd, *J* = 12.7, 2.1 Hz, 1H), 4.05 (ddd, *J* = 10.1, 4.1, 2.1 Hz, 1H), 2.78 (s, 3H), 2.33 (d, *J* = 1.0 Hz, 3H), 2.05 (s, 3H), 2.04 (s, 3H), 2.02 (s, 3H), 1.90 (s, 3H). ^{13}C {^{1}H} NMR ($CDCl_3$, 125 MHz): δ 170.9, 170.2, 169.8, 169.8, 165.4, 148.4, 147.8, 135.5, 131.7, 127.9, 125.7, 123.7, 117.3, 76.6, 74.9, 72.2, 70.3, 68.1, 61.7, 53.6, 20.9, 20.8, 20.7, 16.5, 11.7. ^{19}F NMR ($CDCl_3$, 376 MHz) δ no signals observed ; ^{11}B NMR ($CDCl_3$, 128 MHz) δ -16.89 (s). HRMS (ESI/Q-TOF) m/z: $[M+NH_4]^+$ calcd for C_{27} H_{33} B N_5 O_9: 582.2371; found 582.2350.

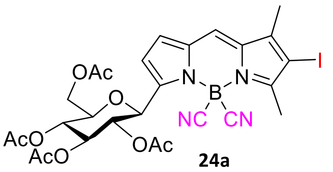

24a

BODIPY **24a**. Compound **24a** was obtained from **23** (125 mg, 0.22 mmol) in CH_2Cl_2 (8 mL) treated with *N*-iodosuccinimide (118 mg, 0.53 mmol) and $BF_3{\cdot}Et_2O$ complex (39 μL, 0.31 mmol) according to procedure **F**. Reaction time: 6 h. The residue was purified by flash chromatography (hexane/ethyl acetate 6:4) to give **24a** as a red solid (108 mg, 71%). This compound was also prepared starting from **21** (484 mg, 0.71 mmol) in CH_2Cl_2 (10 mL) treated with $BF_3{\cdot}Et_2O$ (36 μL, 0.28 mmol) and TMSCN (0.89 mL, 7.1 mmol) during 3 h following the general procedure **C** to give **24a** (467 mg, 95%). $[\alpha]_D^{21}$ -135.6 (*c* 0.04, $CHCl_3$); Mp 110 – 115 °C; ^{1}H NMR ($CDCl_3$, 400 MHz): δ 7.36 (s, 1H), 7.12 (d, *J* = 4.3 Hz, 1H), 6.72 (d, *J* = 4.2 Hz, 1H), 5.59 (dd, *J* = 10.0, 9.0 Hz, 1H), 5.42 (t, *J* = 9.2 Hz, 1H), 5.28 (dd, *J* = 10.1, 9.3 Hz, 1H), 5.10 (d, *J* = 10.0 Hz, 1H), 4.38 (dd, *J* = 12.6, 4.1 Hz, 1H), 4.25 (dd, *J* = 12.7, 2.1 Hz, 1H), 4.06 (ddd, *J* = 10.0, 4.1, 2.1 Hz, 1H), 2.87 (s, 3H), 2.32 (s, 3H), 2.06 (s, 3H), 2.05 (s, 3H), 2.03 (s, 3H), 1.92 (s, 3H).^{13}C {^{1}H} NMR ($CDCl_3$, 125 MHz): δ 170.9, 170.2, 169.8, 169.8, 164.3, 150.1, 149.7, 134.4, 132.0, 129.6, 126.0, 118.3, 76.7, 74.8, 72.2, 70.3, 68.0, 61.6, 20.9, 20.8, 20.7, 17.6, 14.4. ^{11}B NMR ($CDCl_3$, 128 MHz) δ -16.54 (s). HRMS (ESI/Q-TOF) m/z: $[M+NH_4]^+$ calcd for C_{27} H_{32} B I N_5 O_9: 708.1337; found 708.1313.

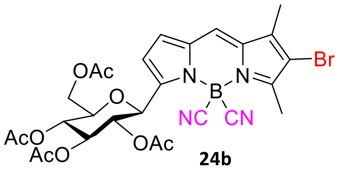

24b

BODIPY **24b**. Compound **24b** was obtained from **23** (50 mg, 0.088 mmol) in CH_2Cl_2 (4 mL) treated with *N*-bromosuccinimide (16.7 mg, 0.106 mmol) and $BF_3{\cdot}Et_2O$ complex (18 μL, 0.15 mmol) according to procedure **F**. Reaction time: 1 h. The residue was purified by flash chromatography

(hexane/ethyl acetate 6:4) to give **24b** as a red solid (46 mg, 82%). $[\alpha]_D^{25}$ +75.8 (*c* 0.06, $CHCl_3$); Mp 125 –127 °C; ^{1}H NMR ($CDCl_3$, 400 MHz): δ 7.35 (s, 1H), 7.11 (d, *J* = 4.3 Hz, 1H), 6.71 (d, *J* = 4.3 Hz, 1H), 5.59 (dd, *J* = 10.1, 9.1 Hz, 1H), 5.42 (t, *J* = 9.2 Hz, 1H), 5.27 (t, *J* = 9.7 Hz, 1H), 5.10 (d, *J* = 10.1 Hz, 1H), 4.37 (dd, *J* = 12.7, 4.2 Hz, 1H), 4.25 (dd, *J* = 12.6, 2.1 Hz, 1H), 4.06 (ddd, *J* = 10.2, 4.1, 2.1 Hz, 1H), 2.83 (s, 3H), 2.31 (s, 3H), 2.05 (s, 3H), 2.04 (s, 3H), 2.03 (s, 3H), 1.91 (s, 3H). ^{13}C {^{1}H} NMR ($CDCl_3$, 125 MHz): δ ^{13}C NMR (101 MHz, $CDCl_3$) δ 170.9, 170.2, 169.8, 161.6, 150.3, 144.3, 133.3, 132.1, 129.6, 126.4, 118.2, 113.9, 76.7, 74.8, 72.1, 70.3, 68.0, 61.6, 20.8, 15.3, 11.6. ^{11}B NMR ($CDCl_3$, 128 MHz) δ -16.79 (s). HRMS (ESI/Q-TOF) m/z: $[M+NH_4]^+$ calcd for C_{27} H_{28} B Br Na N_4 O_9: 667.1014; found 667.1003.

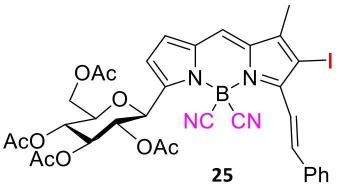

25

BODIPY **25**. Compound **25** was obtained from iodo derivative **24a** (69 mg, 0.1 mmol) in dry DMF (5 mL) treated with benzaldehyde (44 μL, 0.4 mmol) and piperidinium acetate (29 mg, 0.2 mmol) according to procedure **G** during 2 h. Purification: flash chromatography (hexane/ethyl acetate 6:4) to give BODIPY **25** as a dark blue solid (31 mg, 40%). $[\alpha]_D^{21}$ +10528.3 (*c* 0.36, $CHCl_3$); Mp 85 – 87 °C; ^{1}H NMR ($CDCl_3$, 400 MHz): δ 8.39 (d, *J* = 16.4 Hz, 1H), 7.78 – 7.73 (m, 2H), 7.68 (d, *J* = 16.3 Hz, 1H), 7.53 – 7.44 (m, 3H), 7.34 (s, 1H), 7.12 (d, *J* = 3.8 Hz, 1H), 6.76 (d, *J* = 4.3 Hz, 1H), 5.60 (t, *J* = 9.5 Hz, 1H), 5.45 (t, *J* = 9.2 Hz, 1H), 5.28 (t, *J* = 9.7 Hz, 1H), 5.19 (d, *J* = 10.0 Hz, 1H), 4.37 (dd, *J* = 12.4, 4.0 Hz, 1H), 4.22 (dd, *J* = 13.3, 2.4 Hz, 1H), 4.14 – 4.03 (m, 1H), 2.36 (s, 3H), 2.06 (s, 3H), 2.03 (s, 6H), 1.92 (s, 3H). ^{13}C {^{1}H} NMR ($CDCl_3$, 125 MHz): δ 170.9, 170.1, 169.9, 169.8, 155.9, 150.3, 150.0, 145.3, 135.4, 134.9, 132.6, 131.3, 129.4, 129.1, 128.6, 124.6, 118.6, 116.7, 76.7, 74.9, 72.1, 70.4, 68.1, 61.7, 20.9, 20.8, 20.7, 14.8. ^{11}B NMR ($CDCl_3$, 128 MHz) δ -17.47 (s). HRMS (ESI/Q-TOF) m/z: $[M+NH_4]^+$ calcd for C_{34} H_{36} B I N_5 O_9 796.1651; found 796.1624.

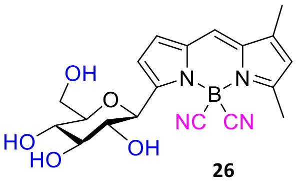

26

BODIPY **26.** According to general procedure **E**, a solution of **23** (141 mg, 0.25 mmol) in THF (5 mL) was treated with HCl/MeOH (2 mL, 2.5 mmol) at 60°C during 12 h. The residue was purified by flash chromatography (CH_2Cl_2: MeOH) to give **26** as a brown solid (59.4 mg, 60%). $[\alpha]_D^{25}$ -435.3 (*c* 0.15, CH_3OH); Mp 135–140 °C; ^{1}H NMR (CD_3OD, 400 MHz): δ 7.81 (s, 1H), 7.26 (d, *J* = 4.2 Hz, 1H),

6.82 (d, *J* = 4.2 Hz, 1H), 6.55 (s, 1H), 4.81 (d, *J* = 10.2 Hz, 1H), 4.04 – 3.94 (m, 1H), 3.90 – 3.76 (m, 2H), 3.63 – 3.49 (m, 4H), 2.75 (s, 3H), 2.40 (s, 3H). ^{13}C {^{1}H} NMR (CD_3OD, 125 MHz): δ 163.1, 152.7, 147.9, 134.7, 132.1, 128.5, 126.6, 122. 8, 117. 5, 80.8, 78.7, 74.1, 72.5, 69.6, 60.8, 14.8, 10.0; ^{19}F NMR ($CDCl_3$, 376 MHz) δ no signals observed; ^{11}B NMR ($CDCl_3$, 128 MHz) δ -17.57 (s). HRMS (ESI/Q-TOF) m/z: $[M+NH_4]^+$ calcd for C_{19} H_{25} B N_5 O_5: 414.1947; found 414.1940.

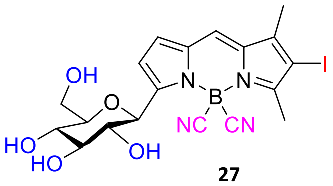

27

BODIPY **27.** According to general procedure **E**, a solution of **24a** (156 mg, 0.22 mmol) in THF (5 mL) was treated with HCl/MeOH (10 mL, 12.5 mmol) during 12 h at 60 °C. The residue was purified by flash chromatography (CH_2Cl_2: MeOH, 9:1) to give **27** as a red solid (71 mg, 62%). $[\alpha]_D^{25}$ +203.4 (*c* 1.5, CH_3OH); Mp > 300 °C; ^{1}H NMR (CD_3OD, 400 MHz): δ 7.94 (s, 1H), 7.38 (d, *J* = 4.3 Hz, 1H), 6.90 (d, *J* = 4.3 Hz, 1H), 4.81 (d, *J* = 9.8 Hz, 1H), 4.00 (dd, *J* = 12.2, 2.1 Hz, 1H), 3.90 – 3.79 (m, 2H), 3.68 – 3.49 (m, 3H), 2.83 (s, 3H), 2.38 (s, 3H). ^{13}C NMR (126 MHz, CD_3OD) δ 162.3, 156.3, 150.5, 134.7, 133.9, 131.9, 128.6, 120.1, 82.3, 80.0, 75.6, 73.8, 70.9, 62.2, 17.4, 14.2. ^{11}B NMR (CD_3OD, 128 MHz) δ -17.38 (s). HRMS (ESI/Q-TOF) m/z: $[M+NH_4]^+$ calcd for C_{19} H_{24} B I N_5 O_5 540.0913; found 540.0893.

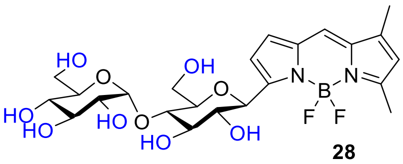

28

BODIPY **28.** A stirred solution of **12c** (100 mg, 0.11mmol) in a CH_2Cl_2:MeOH (1 :1) mixture (4 mL) was treated with NaOMe (51 mg, 1.47 mmol) for 12 h according to procedure **D**. The residue was purified by column chromatography on silica gel (AcOEt/MeOH/H_2O 20:2:1) to give compound **28** (20 mg, 30%) as a brown solid. $[\alpha]_D^{25}$ +434.2 (*c* 0.1, $CHCl_3$); Mp 183 – 185 °C; ^{1}H NMR NMR (CD_3OD, 400 MHz): δ 7.55 (s, 1H), 7.06 (d, *J* = 4.1 Hz, 1H), 6.61 (d, *J* = 4.1 Hz, 1H), 6.31 (s, 1H), 5.24 (d, *J* = 3.8 Hz, 1H), 4.74 (d, *J* = 9.2 Hz, 1H), 4.59 (s, 1H), 3.93 – 3.62 (m, 8H), 3.53 (dd, *J* = 9.6, 2.6 Hz, 1H), 3.48 (dd, *J* = 9.7, 3.7 Hz, 1H), 3.28 (m, 1H), 2.56 (s, 3H), 2.33 (s, 3H). ^{13}C {^{1}H} NMR (CD_3OD, 125 MHz): δ 153.5, 147.6, 137.6, 134.7, 128.3, 126.7, 122.4, 116.6, 102.9, 81.4, 80.9, 79.7, 76.1, 75.1, 74.8, 74.5, 74.3, 71.6, 62.7, 62.5, 15.1, 11.2. ^{19}F NMR (CD_3OD, 376 MHz) δ -142.74 (dq, *J* = 104.2, 32.5 Hz), -143.48 (dq, *J* = 104.5, 32.7 Hz); ^{11}B NMR (CD_3OD, 128 MHz) δ 0.90 (t, *J* = 32.4 Hz). HRMS (ESI/Q-TOF) m/z: calcd for C_{23} H_{31} B F_2 N_2 O_{10} $[M+NH_4]^+$: 562.2382; found 562.2346.

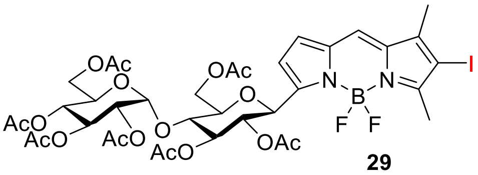

29

BODIPY **29**. A stirred solution of BODIPY **12c** (39 mg, 0.046 mmol) in CH_2Cl_2 (5 mL) was treated with *N*-iodosuccinimide (16 mg, 0.07 mmol) and $BF_3{\cdot}Et_2O$ (3 μL, 0.02 mmol) according to the general procedure **F** during 30 min. Purification by column chromatography (hexane/ethyl acetate 6:4) gave compound **29** as a red solid (44.8 mg, 80%). $[\alpha]_D^{25}$ +888.0 (*c* 0.1, $CHCl_3$); Mp 110 – 112 °C; ^{1}H NMR ($CDCl_3$, 400 MHz): δ 7.19 (s, 1H), 6.94 (d, *J* = 4.2 Hz, 1H), 6.49 (d, *J* = 4.2 Hz, 1H), 5.43 (d, *J* = 4.3 Hz, 1H), 5.43– 5.34 (m, 2H), 5.26 (t, *J* = 9.4 Hz, 1H), 5.09– 5.03 (m, 2H), 4.89 (dd, *J* = 10.5, 4.0 Hz, 1H), 4.47 (dd, *J* = 12.3, 2.4 Hz, 1H), 4.24 (ddd, *J* = 11.7, 7.3, 4.1 Hz, 2H), 4.14 – 4.03 (m, 2H), 3.99 (dt, *J* = 10.2, 3.1 Hz, 1H), 3.92 (ddd, *J* = 9.8, 4.6, 2.4 Hz, 1H), 2.65 (s, 3H), 2.24 (s, 3H), 2.10 (s, 3H), 2.10 (s, 3H), 2.08 (s, 3H), 2.03 (s, 3H), 2.01 (s, 3H), 2.00 (s, 6H), 1.85 (s, 3H). ^{13}C {^{1}H} NMR ($CDCl_3$, 125 MHz) δ: 170.8, 170.7, 170.7, 170.2, 170.0, 169.7, 163.0, 150.6, 147.9, 135.7, 133.3, 128.5, 125.3, 116.1, 95.9, 77.0, 76.5, 73.4, 72.7, 71.7, 70.2, 69.6, 68.6, 68.2, 63.4, 61.7, 21.1, 21.0, 20.8, 20.7, 20.6, 16.3, 14.0.^{19}F NMR ($CDCl_3$, 376 MHz) δ -141.74 (dq, *J* = 99.6, 32.8 Hz), -145.24 (dq, *J* = 101.1, 31.7 Hz); ^{11}B NMR (CD_3OD, 128 MHz) δ 0.62 (t, *J* = 32.2 Hz). HRMS (ESI/Q-TOF) m/z: $[M+NH_4]^+$ calcd for C_{37} H_{48} B F_2 I N_3 O_{17}: 982.2091 found: 982.2080.

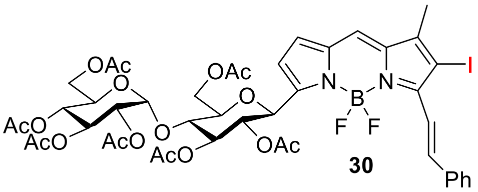

30

BODIPY **30**. According to general procedure **G**, a solution of maltosyl-BODIPY **29** (33 mg, 0.031 mmol) and benzaldehyde (14 μL, 0.124 mmol) in dry DMF (2mL) was added piperidinium acetate (9 mg, 0.062 mmol) at r.t. (4 h). Purification by column chromatography (hexane/ethyl acetate 6:4) gave compound **30** as a blue solid (6 mg, 20%). $[\alpha]_D^{25}$+13842 (*c* 0.03, $CHCl_3$); Mp 90 – 95 °C; ^{1}H NMR ($CDCl_3$, 400 MHz): δ 8.31 (d, *J* = 16.7 Hz, 1H), 7.73 – 7.69 (m, 2H), 7.65 (d, *J* = 16.7 Hz, 1H), 7.47 (t, *J* = 7.4 Hz, 2H), 7.41 (d, *J* = 7.3 Hz, 1H), 7.21 (s, 1H), 6.97 (d, *J* = 4.2 Hz, 1H), 6.54 (d, *J* = 3.8 Hz, 1H), 5.47 (t, *J* = 8.7 Hz, 1H), 5.44 (d, *J* = 4.0 Hz, 1H), 5.39 (dd, *J* = 10.6, 9.5 Hz, 1H), 5.31 – 5.25 (m, 1H), 5.13 (d, *J* = 9.9 Hz, 1H), 5.08 (dd, *J* = 10.2, 9.5 Hz, 1H), 4.90 (dd, *J* = 10.5, 4.0 Hz, 1H), 4.49 (dd, *J* = 12.3, 2.4 Hz, 1H), 4.31 – 4.20 (m, 2H), 4.10 (dd, *J* = 9.7, 8.5 Hz, 1H), 4.06 (dd, *J* = 12.5, 2.3 Hz, 1H), 3.99 (ddd, *J* = 9.7, 7.1, 2.9 Hz, 2H), 2.29 (s, 3H), 2.10 (s, 3H), 2.09 (s, 3H), 2.08 (s, 3H), 2.03 (s, 3H), 2.02 (s, 3H), 2.01 (s, 3H), 1.85 (s, 3H). ^{13}C {^{1}H} NMR ($CDCl_3$, 125 MHz): δ 170.7, 170.6, 170.6, 170.1, 169.8, 169.5, 154.6, 151.0, 148.3, 141.8, 136.3, 136.1, 133.9, 130.0, 129.0, 128.3, 128.0,

124.2, 118.2, 116.5, 95.8, 76.4, 73.3, 72.5, 71.6, 70.0, 69.4, 68.5, 68.0, 63.3, 61.5, 21.0, 20.8, 20.7, 20.7, 20.6, 20.5. ^{19}F NMR (CD_3OD, 376 MHz) δ -138.13 (dq, *J* = 100.1, 33.6 Hz), -145.24 (dq, *J* = 98.3, 32.5 Hz); ^{11}B NMR (CD_3OD, 128 MHz) δ 0.92 (t, *J* = 33.2 Hz). HRMS (ESI/Q-TOF) m/z: $[M+NH_4]^+$ calcd for $C_{44}H_{52}BF_2IN_3O_{17}$: 1070.2405 found: 1070.2395.

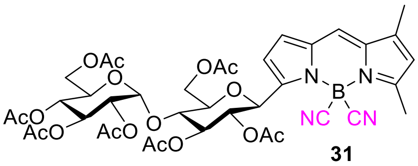

31

BODIPY **31**. A solution of the BF_2-BODIPY **12c** (250 mg, 0.3 mmol) in anhydrous CH_2Cl_2 (32 mL) was cooled to 0 °C and treated with BF_3·Et_2O (19 μL, 0.15 mmol) and TMSCN (0.37 mL, 3 mmol) according to procedure **C** during 30 min. Purification by column chromatography (hexane/ethyl acetate 6:4) gave compound **31** as a red solid (199 mg, 78%). $[\alpha]_D^{25}$ +127.2 (*c* 0.5, $CHCl_3$); Mp 108 – 109 °C; ^{1}H NMR ($CDCl_3$, 400 MHz): δ 7.28 (s, 1H), 7.01 (d, *J* = 4.2 Hz, 1H), 6.57 (d, *J* = 4.2 Hz, 1H), 6.40 (d, *J* = 1.1 Hz, 1H), 5.50 – 5.42 (m, 3H), 5.39 (dd, *J* = 10.5, 9.5 Hz, 1H), 5.13 – 5.05 (m, 2H), 4.90 (dd, *J* = 10.5, 4.0 Hz, 1H), 4.58 (dd, *J* = 12.4, 2.3 Hz, 1H), 4.32 (dd, *J* = 12.5, 3.7 Hz, 1H), 4.25 (dd, *J* = 12.4, 3.2 Hz, 1H), 4.22 – 4.18 (m, 1H), 4.09 – 3.94 (m, 3H), 2.80 (s, 3H), 2.34 (s, 3H), 2.10 (s, 9H), 2.03 (s, 6H), 2.01 (s, 3H), 1.89 (s, 3H). ^{13}C {^{1}H} NMR ($CDCl_3$, 125 MHz): δ 170.9, 170.8, 170.7, 170.3, 170.2, 169.9, 169.7, 165.5, 148.6, 147.7, 135.5, 131.8, 127.8, 125.7, 123.7, 117.0, 96.0, 77.4, 76.7, 72.9, 72.0, 71.1, 70.2, 69.6, 68.6, 68.1, 63.0, 61.6, 21.1, 21.0, 20.9, 20.8, 16.6, 11.7. ^{11}B NMR ($CDCl_3$, 128 MHz) δ -17.74 (s). HRMS (ESI/Q-TOF) m/z: $[M+NH_4]^+$calcd for $C_{39}H_{49}BN_5O_{17}$ 870.3218; found 870.3194.

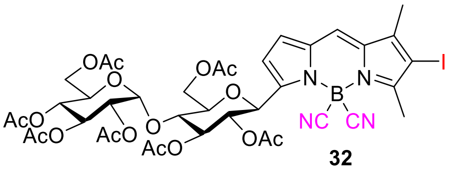

32

BODIPY **32**. A stirred solution of BODIPY **31** (140 mg, 0.16 mmol) in CH_2Cl_2 (10 mL) was treated with *N*-iodosuccinimide (73.6 mg, 0.32 mmol) and BF_3·Et_2O (14 μL, 0.11 mmol) for 2 h following experimental procedure **F**. Purification by column chromatography (CH_2Cl_2/MeOH 98:2) gave **32** as an orange solid (180 mg, 99%). Iodo-BODIPY **32** could be efficiently prepared by F →CN exchange from iodo-BODIPY **29** following the general procedure **C**. Accordingly, a solution of BODIPY **29** (25 mg, 0.025 mmol) in dry CH_2Cl_2 (4 mL) was treated with BF_3·Et_2O (1.3 μL, 0.01 mmol) and TMSCN (32 μL, 0.25 mmol). After 15 min. of reaction and purification by flash chromatography

on silica gel, compound **32** (20 mg, 95%) was obtained. $[\alpha]_D^{25}$ +2491 (*c* 0.1, $CHCl_3$); Mp 87 – 91 °C; ^{1}H NMR ($CDCl_3$, 400 MHz): δ 7.35 (s, 1H), 7.10 (d, *J* = 4.3 Hz, 1H), 6.62 (d, *J* = 4.3 Hz, 1H), 5.48 – 5.43 (m, 3H), 5.39 (dd, *J* = 10.5, 9.5 Hz, 1H), 5.08 (t, *J* = 9.6 Hz, 2H), 4.90 (dd, *J* = 10.5, 4.0 Hz, 1H), 4.58 (dd, *J* = 12.5, 2.3 Hz, 1H), 4.32 (dd, *J* = 12.5, 3.8 Hz, 1H), 4.25 (dd, *J* = 12.4, 3.4 Hz, 1H), 4.20 (dd, *J* = 8.4, 1.4 Hz, 1H), 4.07 – 3.91 (m, 3H), 2.87 (s, 3H), 2.31 (s, 3H), 2.10 (s, 9H), 2.03 (s, 6H), 2.01 (s, 3H), 1.90 (s, 3H). ^{13}C {^{1}H} NMR ($CDCl_3$, 125 MHz): δ 170.7, 170.6, 170.6, 170.0, 170.0, 169.7, 169.6, 163.9, 150.0, 149.6, 134.2, 131.9, 129.5, 126.0, 117.7, 95.9, 86.7, 77.0, 76.7, 72.7, 71.9, 70.9, 70.1, 69.4, 68.5, 67.9, 62.8, 61.4, 20.9, 20.9, 20.7, 20.6, 20.5, 17.4, 14.2. HRMS (ESI/Q-TOF) m/z: $[M+NH_4]^+$ calcd for C_{39} H_{48} B I N_5 O_{17} 996.2184; found 996.2178.

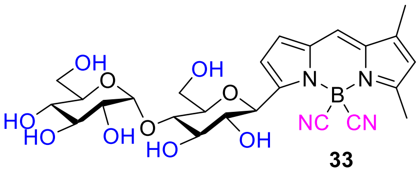

33

BODIPY **33**. Compound **33** was prepared according to general procedure **D** from **31** (250 mg, 0.29 mmol) and NaOMe (221.4 mg, 4.1 mmol) in 12 mL CH_2Cl_2:MeOH (1:1) during 24 h. The residue was purified by flash chromatography (AcOEt/MeOH/H_2O 15:2:1) to give **33** as a brown solid (37.2 mg, 23%). $[\alpha]_D^{25}$ +1202.4 (*c* 0.02, CH_3OH); Mp 126 –128 °C; ^{1}H NMR (CD_3OD, 400 MHz) δ: 7.80 (s, 1H), 7.24 (d, *J* = 4.1 Hz, 1H), 6.82 (d, *J* = 4.2 Hz, 1H), 6.54 (s, 1H), 5.24 (d, *J* = 3.7 Hz, 1H), 4.79 (d, *J* = 9.3 Hz, 1H), 4.03 (m, 1H), 3.92 (m, 1H), 3.88 – 3.39 (m, 10H), 2.74 (s, 3H), 2.39 (s, 3H). ^{13}C {^{1}H} NMR (CD_3OD, 125 MHz) δ: 164.6, 153.7, 149.3, 136.1, 133.4, 129.8, 127.9, 124.2, 118.8, 103.0, 80.9, 80.5, 79.8, 75.4, 75.1, 74.9, 74.4, 73.5, 71.6, 62.8, 61.50, 16.1, 11.4. ^{11}B NMR (CD_3OD, 128 MHz) δ -17.63 (s). HRMS (ESI/Q-TOF) m/z: $[M+Na]^+$ calcd for C_{25} H_{31} B N_4 Na O_{10} 581.2031; found 581.2034.

3. Table S1. Photophysical properties of the non-halogenated glyco-BODIPYs

Table S1. Photophysical properties of the non-halogenated glyco-BODIPYs in diluted (2 μM) water solutions.

	λ_{ab} (nm)	ε_{max} (10^4 $M^{-1}cm^{-1}$)	λ_{fl} (nm)	Φ_{fl}	τ_{fl} (ns)
**19**	495.0	2.4	506.0	0.71	5.81
**26**	494.0	2.4	506.0	0.79	6.06
**28**	495.0	1.6	506.0	0.75	5.72

Absorption (λ_{ab}) and fluorescence (λ_{fl}) wavelength, molar absorption coefficient at the maximum (ε_{max}), fluorescence quantum yield (Φ_{fl}) and lifetime (τ_{fl})

4. Table S2. Photophysical properties of the halogenated glyco-BODIPYs

Table S2. Photophysical properties of the halogenated glyco-BODIPYs in diluted (2 μM) solutions.

		λ_{ab} (nm)	ε_{max} (10^4 $M^{-1}cm^{-1}$)	λ_{fl} (nm)	Φ_{fl}	τ_{fl} (ns)
**20**	MeOH	521.0	3.8	531.0	0.015	0.02(98%)-4.80(2%)
	$CHCl_3$	529.0	3.3	537.0	0.016	0.02(98%)-4.49(2%)
	H_2O	519.0	2.8	530.0	0.005	-
**21**	MeOH	520.0	4.6	531.0	0.015	0.02(98%)-4.79(2%)
	$CHCl_3$	528.0	5.3	536.0	0.016	0.02(98%)-4.49(2%)
**24a**	MeOH	520.0	3.7	529.0	0.006	-
	$CHCl_3$	529.0	3.8	539.0	0.007	-
**24b**	MeOH	516.0	3.3	526.0	0.08	0.78(99%)-3.19(1%)
	$CHCl_3$	524.0	4.4	531.0	0.10	0.55(99%)-1.87(1%)
**27**	MeOH	522.0	2.9	531.0	0.011	0.02(99%)-4.85(1%)
	$CHCl_3$	530.0	2.4	534.0	0.014	0.02(99%)-4.40(1%)
	H_2O	519.0	2.0	523.0	0.012	0.02(98%)-4.30(2%)
**29**	MeOH	520.0	3.7	532.0	0.006	-
	$CHCl_3$	528.0	3.8	537.0	0.010	0.02(98%)-4.52(2%)
**30**	MeOH	571.0	4.4	587.0	0.09	0.47(91%)-3.47(9%)
	$CHCl_3$	579.0	4.4	588.0	0.14	0.80(90%)-3.48(10%)

Absorption (λ_{ab}) and fluorescence (λ_{fl}) wavelength, molar absorption coefficient at the maximum (ε_{max}), fluorescence quantum yield (Φ_{fl}) and lifetime (τ_{fl})
MeOH: methanol; $CHCl_3$: chloroform; H_2O: water

5. Figure S1. Absorption and normalized fluorescence spectra of non-halogenated and unprotected glycol-BODIPYs

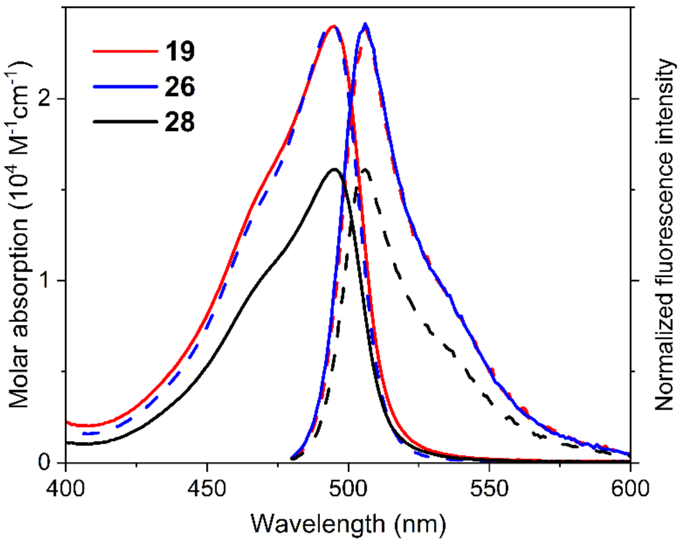

Figure S1. Absorption and normalized fluorescence spectra of non-halogenated and unprotected glycol-BODIPYs **19**, **26** and **28** in diluted (2 μM) water solutions.

6. Figure S2. Nanosecond-resolved transient absorption spectra

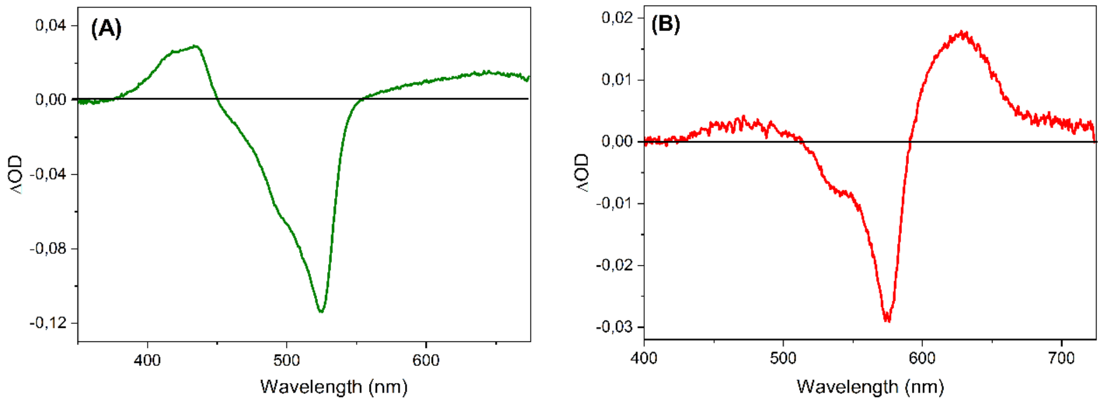

Figure S2. Nanosecond-resolved transient absorption spectra recorded by flash photolysis for representative monoiodinated glyco-BODIPYs **21** (A) and **30** (B) in chloroform

7. Figure S3. Time-dependent emission spectra

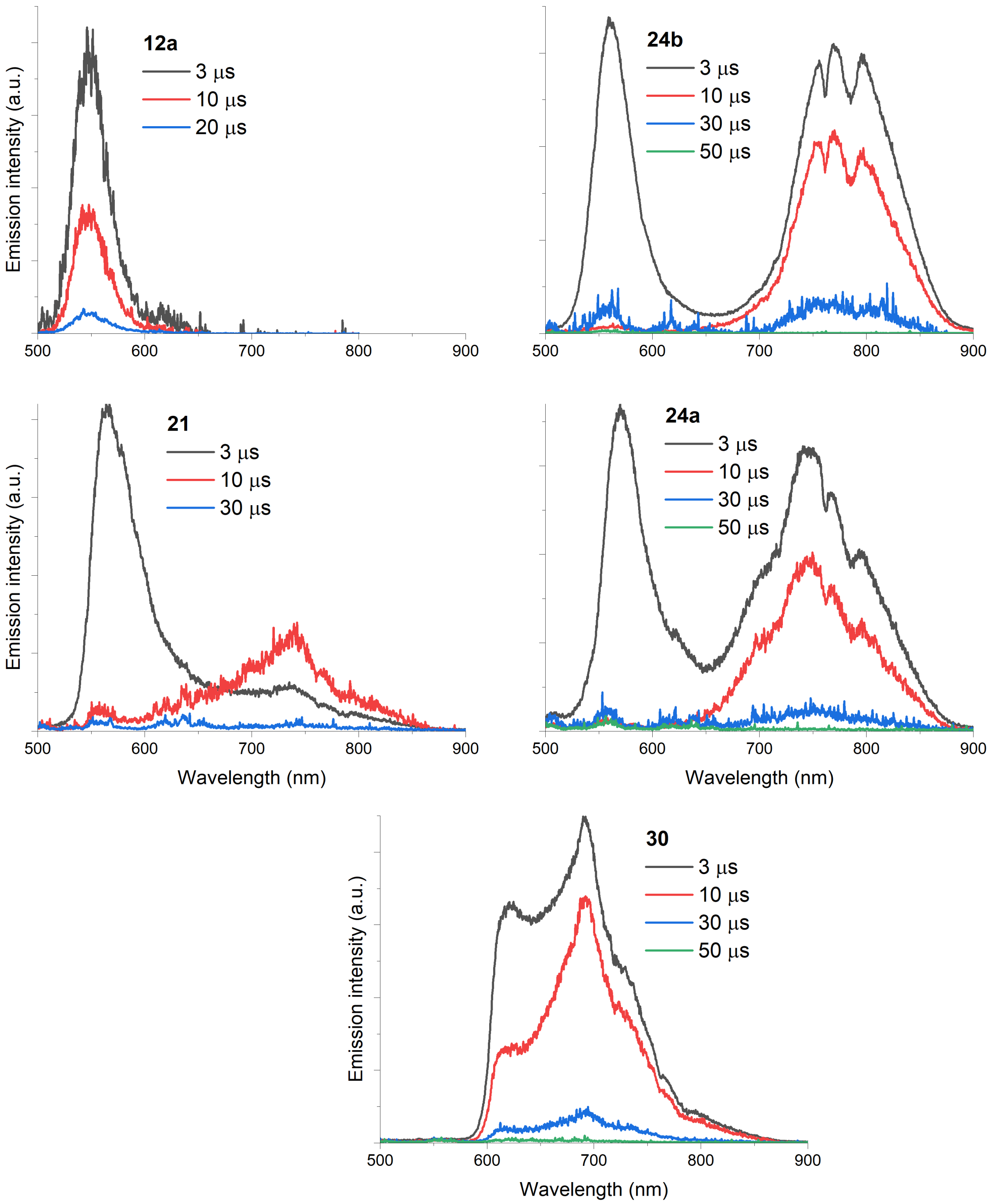

Figure S3. Time-dependent emission spectra of BODIPYs **12a, 21**, **24a**, **24b**, and **30** in methanol recorded under ambient conditions after laser photoexcitation at 532 nm. Optically matched solutions were used.

8. Copies of ^{1}H, ^{13}C {^{1}H}, ^{19}F, ^{11}B NMR Spectra

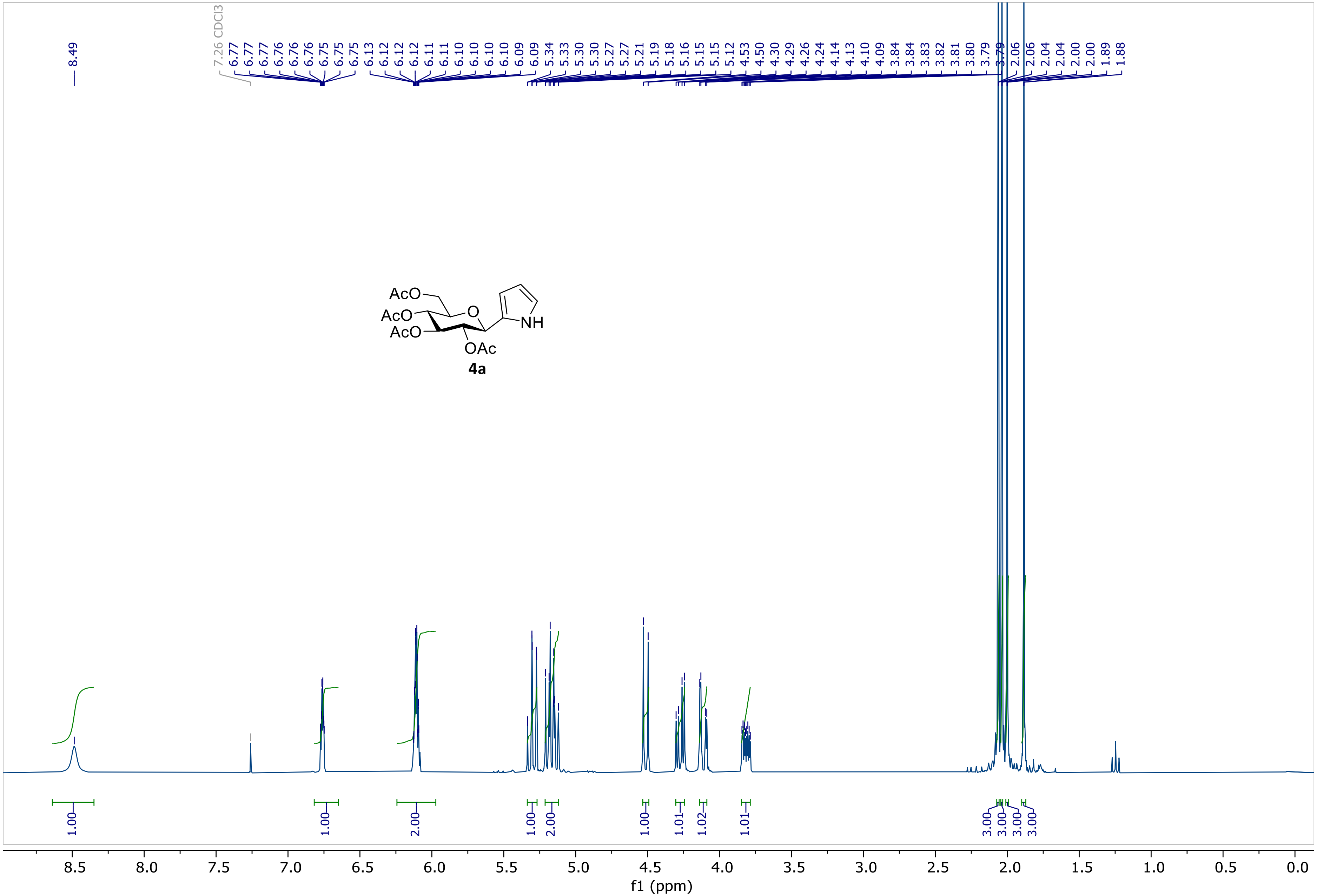

1H NMR CDCl3 400 MHz

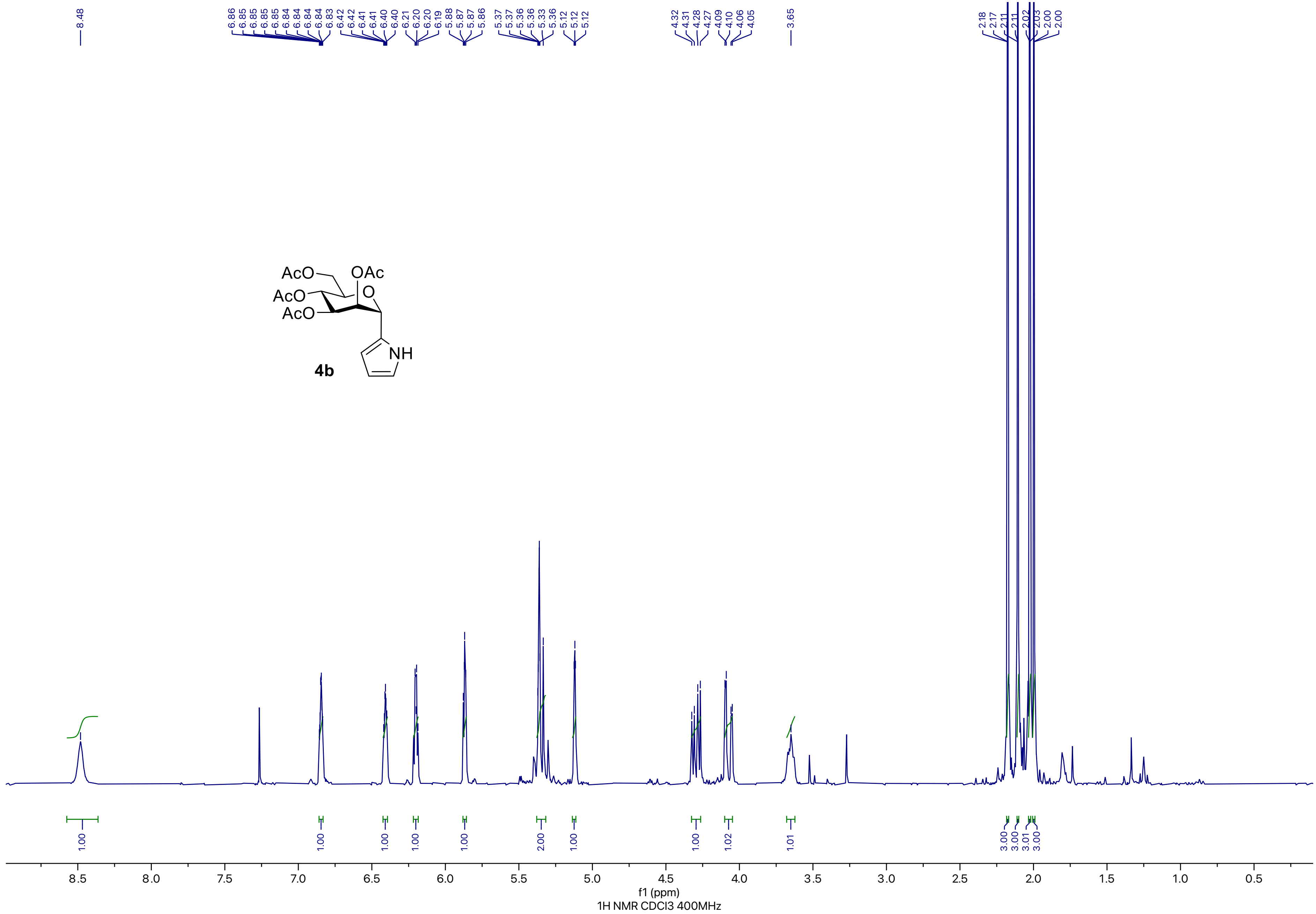
8.48
6.86 6.85 6.85 6.85 6.85 6.84 6.84 6.84 6.84 6.83 6.42 6.42 6.41 6.41 6.40 6.40 6.21 6.20 6.20 6.19 5.88 5.87 5.87 5.86 5.37 5.37 5.36 5.36 5.33 5.36 5.12 5.12 5.12
4.32 4.31 4.28 4.27 4.09 4.10 4.06 4.05
3.65
2.18 2.17 2.11 2.11 2.02 2.03 2.00 2.00
AcO
OAc
AcO
AcO
O
NH
4b
1.00 1.00 1.00 1.00 1.00 2.00 1.00 1.00 1.02 1.01 3.00 3.00 3.01 3.00
8.5 8.0 7.5 7.0 6.5 6.0 5.5 5.0 4.5 4.0 3.5 3.0 2.5 2.0 1.5 1.0 0.5
f1 (ppm)
1H NMR CDCl3 400MHz

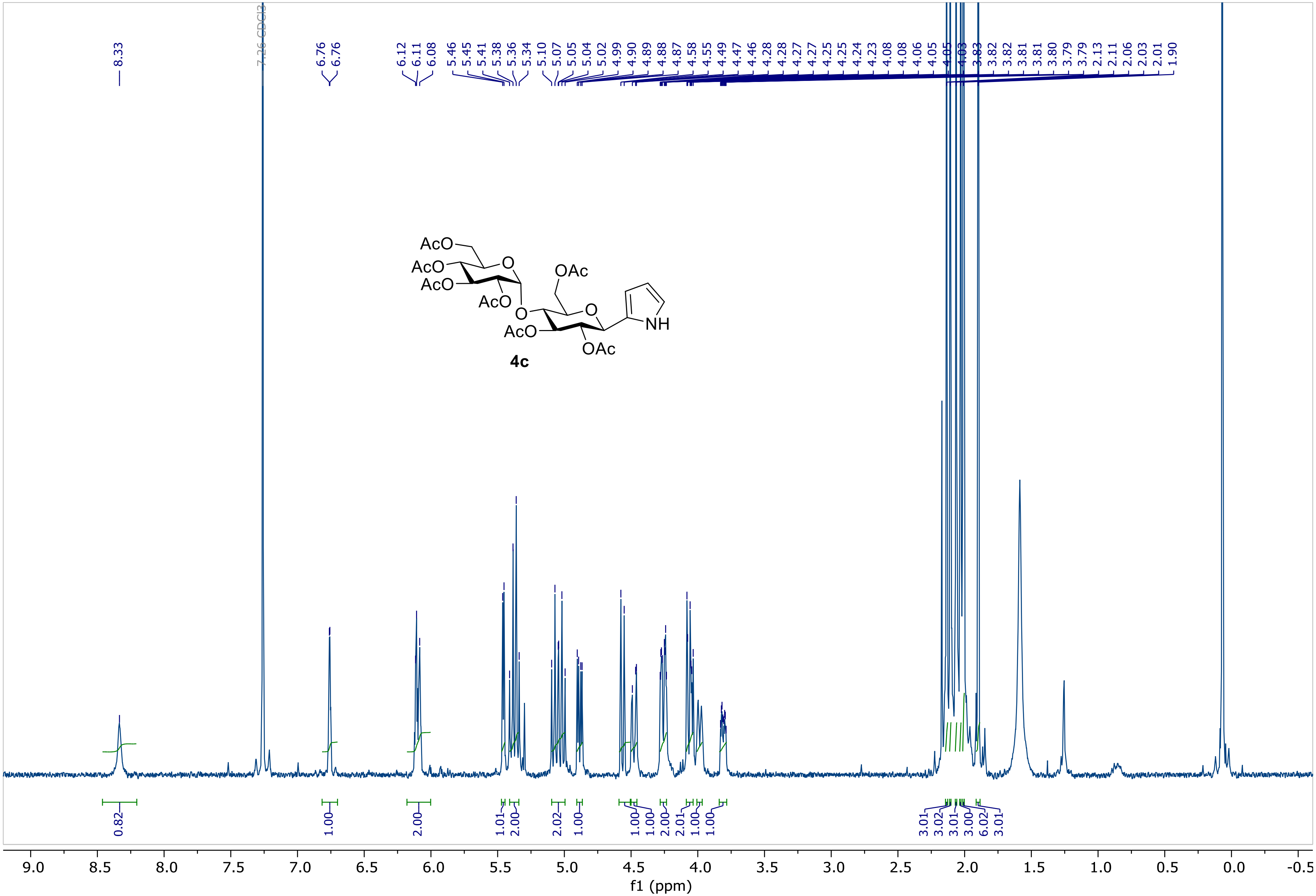

1H NMR CDCl3 400MHz

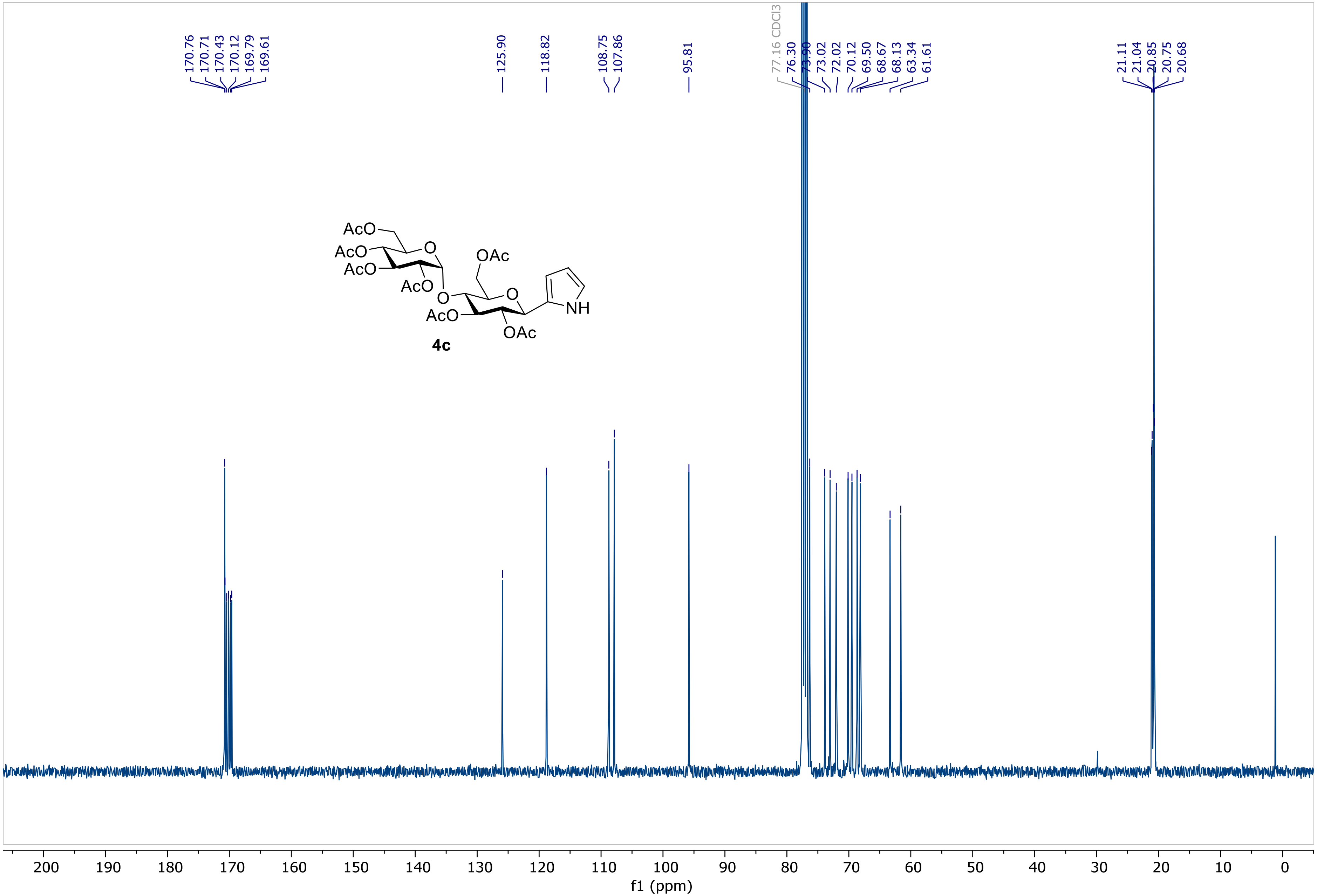

13C{1H} NMR CDCl3 125 MHz

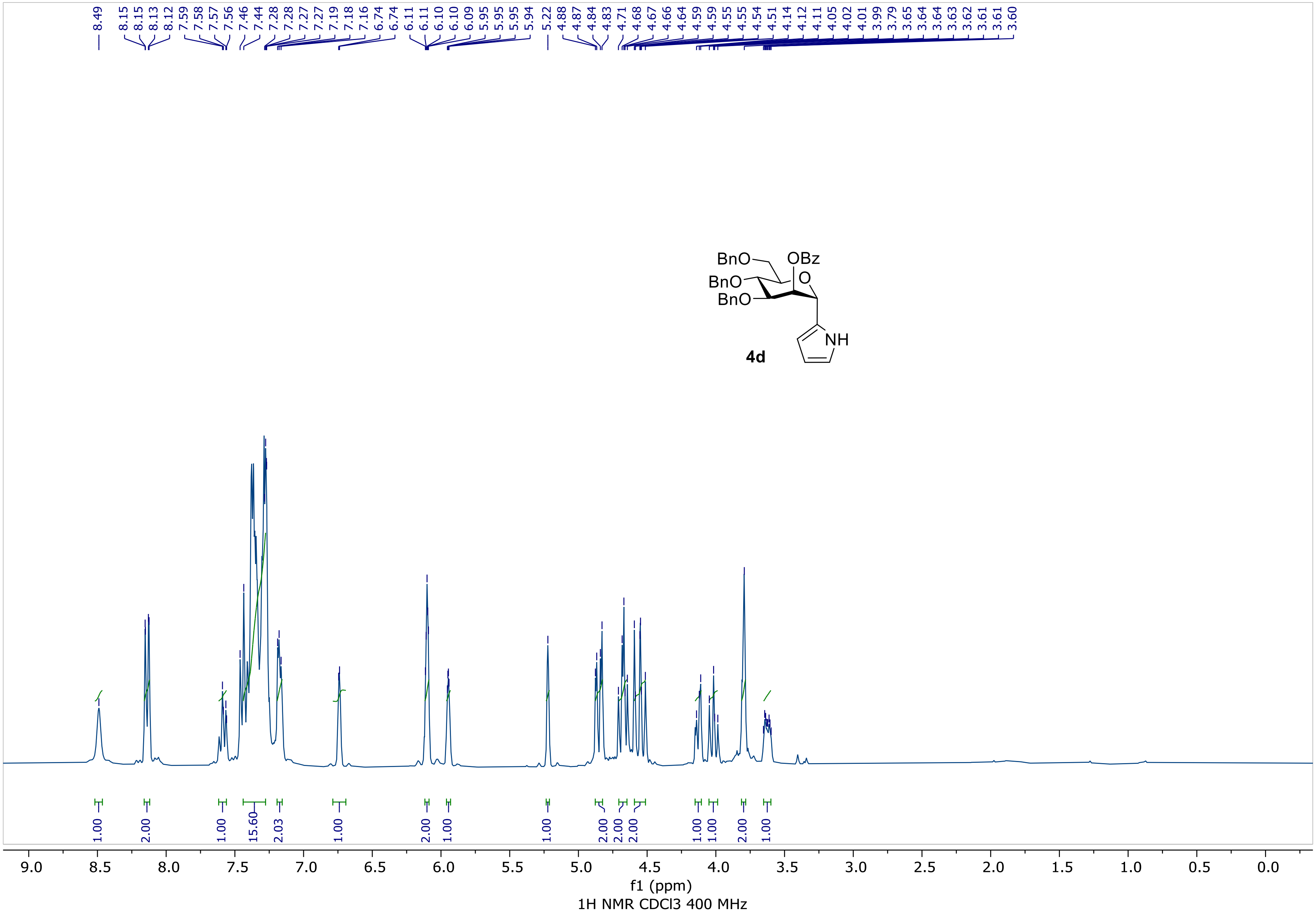
8.49
8.15
8.15
8.13
8.12
7.59
7.58
7.57
7.56
7.46
7.44
7.28
7.28
7.27
7.27
7.19
7.18
7.16
6.74
6.74
6.11
6.11
6.10
6.10
6.09
5.95
5.95
5.95
5.94
5.22
4.88
4.87
4.84
4.83
4.71
4.68
4.67
4.66
4.64
4.59
4.59
4.55
4.55
4.54
4.51
4.14
4.12
4.11
4.05
4.02
4.01
3.99
3.79
3.65
3.64
3.64
3.63
3.62
3.61
3.61
3.60
BnO
BnO
BnO
OBz
O
NH
4d
1.00
2.00
1.00
15.60
2.03
1.00
2.00
1.00
1.00
2.00
2.00
2.00
1.00
1.00
2.00
1.00
9.0
8.5
8.0
7.5
7.0
6.5
6.0
5.5
5.0
4.5
4.0
3.5
3.0
2.5
2.0
1.5
1.0
0.5
0.0
f1 (ppm)
1H NMR CDCl3 400 MHz

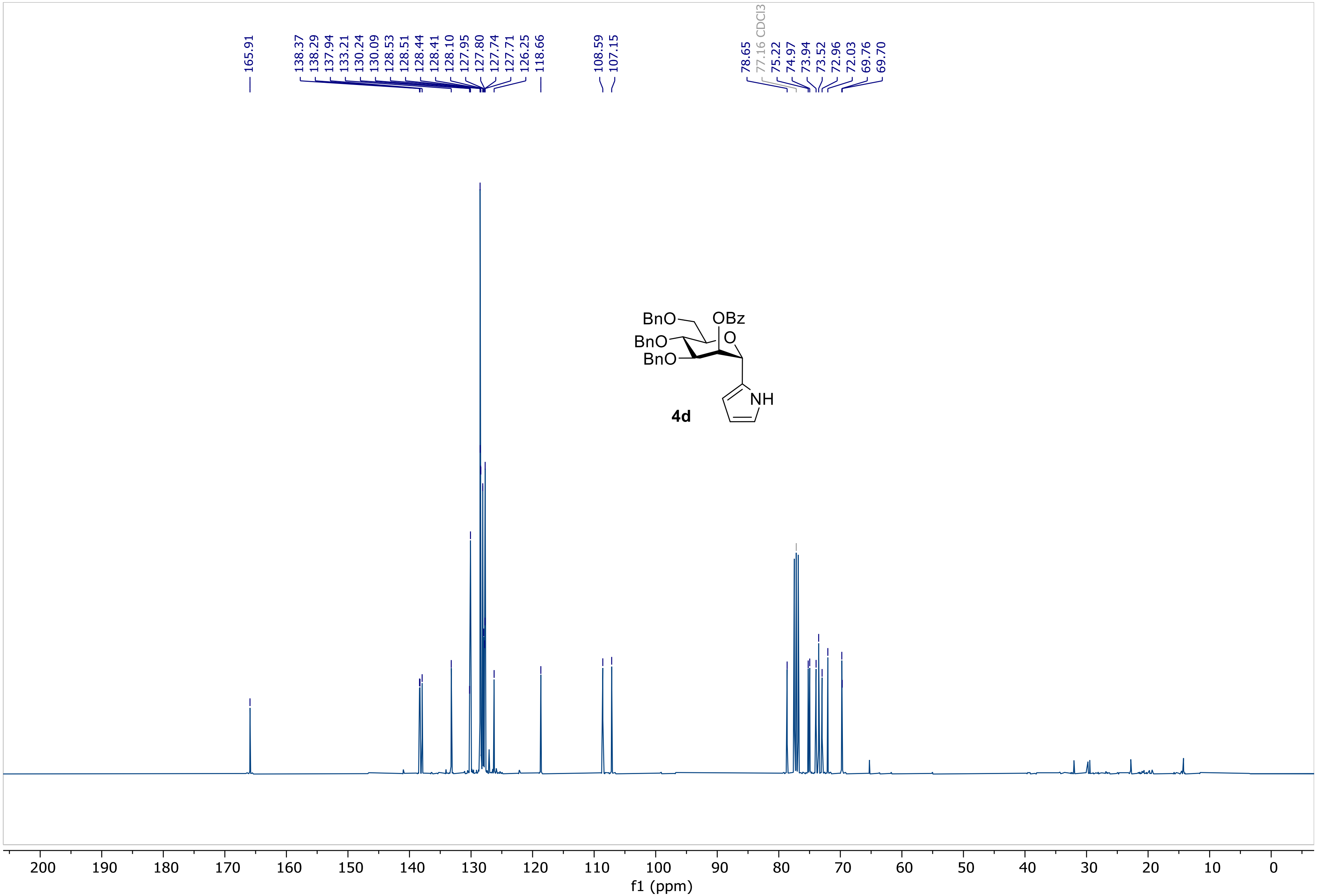

13C{1H} NMR CDCl3 125 MHz

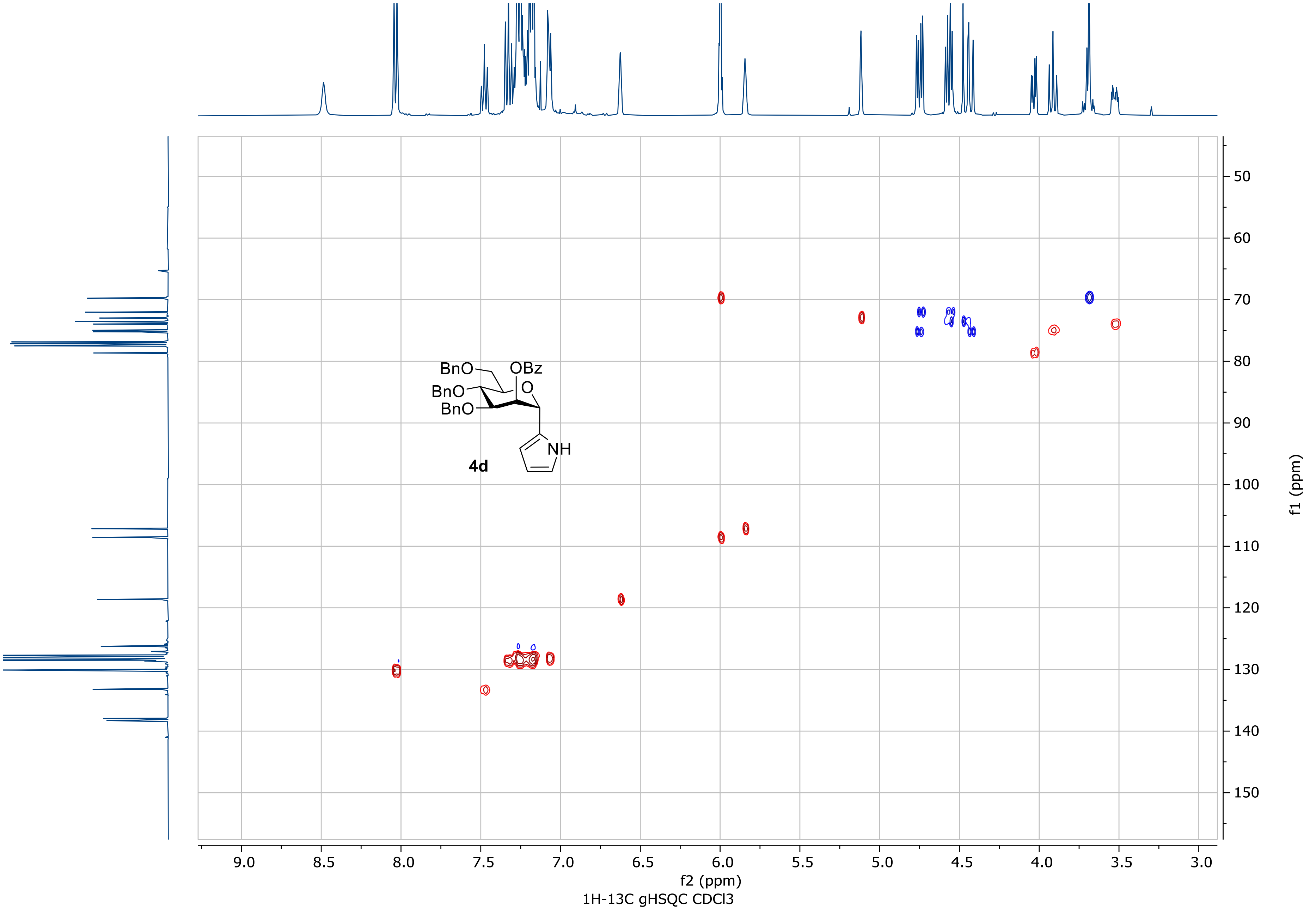
BnO
OBz
BnO
O
BnO
NH
4d
9.0 8.5 8.0 7.5 7.0 6.5 6.0 5.5 5.0 4.5 4.0 3.5 3.0
f2 (ppm)
50 60 70 80 90 100 110 120 130 140 150
f1 (ppm)
1H-13C gHSQC CDCl3

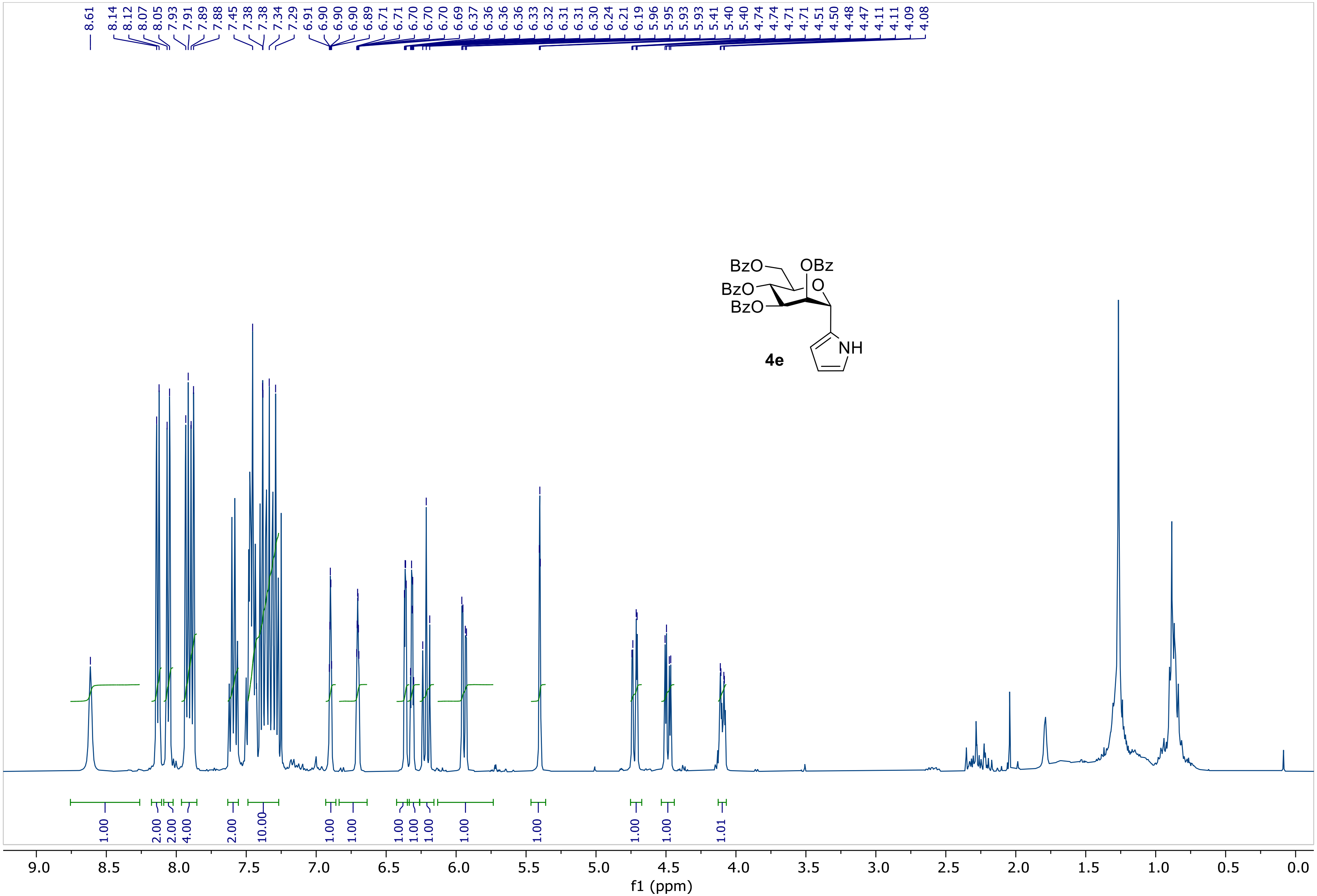

1H NMR 400 MHz CDCl3

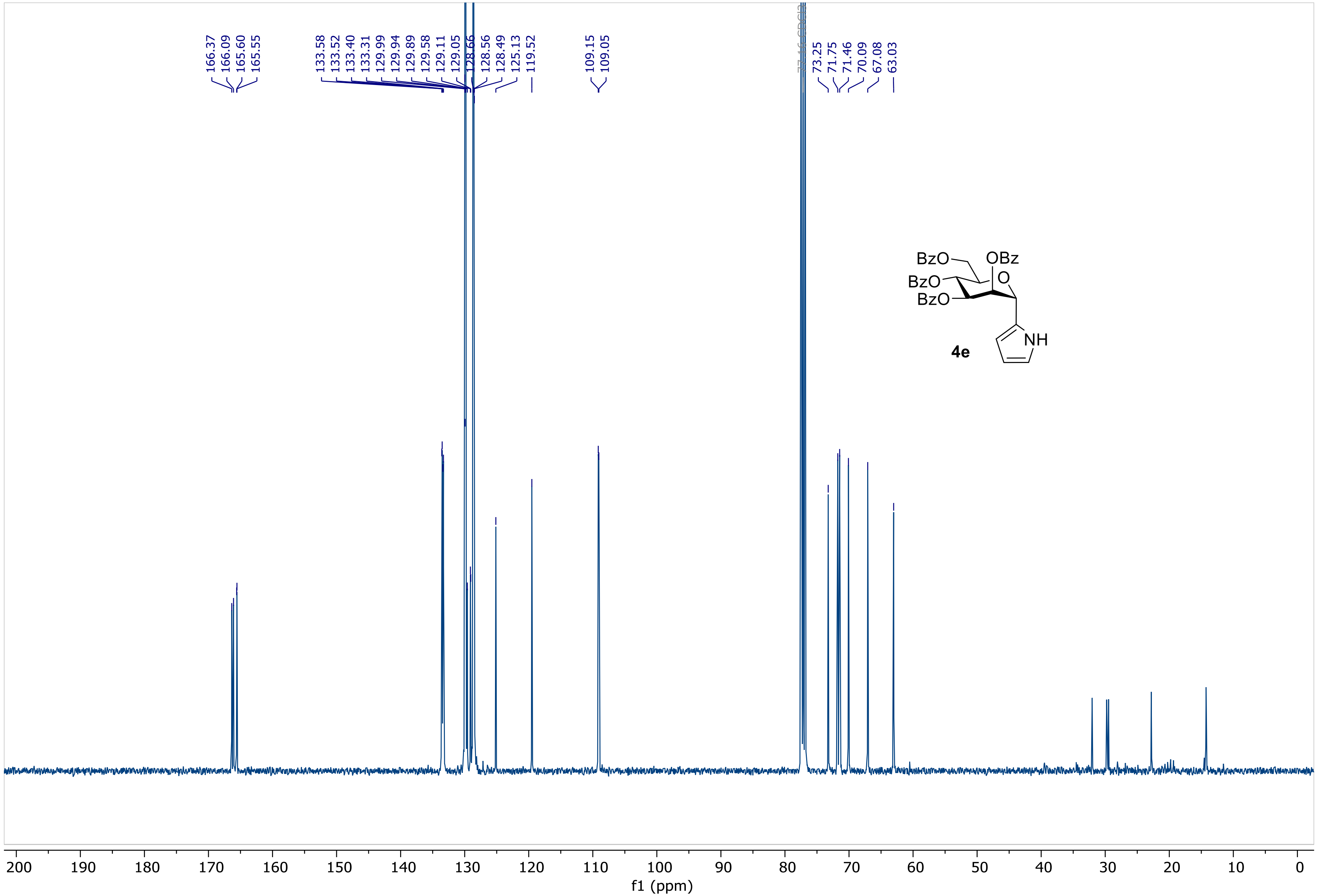

13C{1H} NMR CDCl3 125 MHz

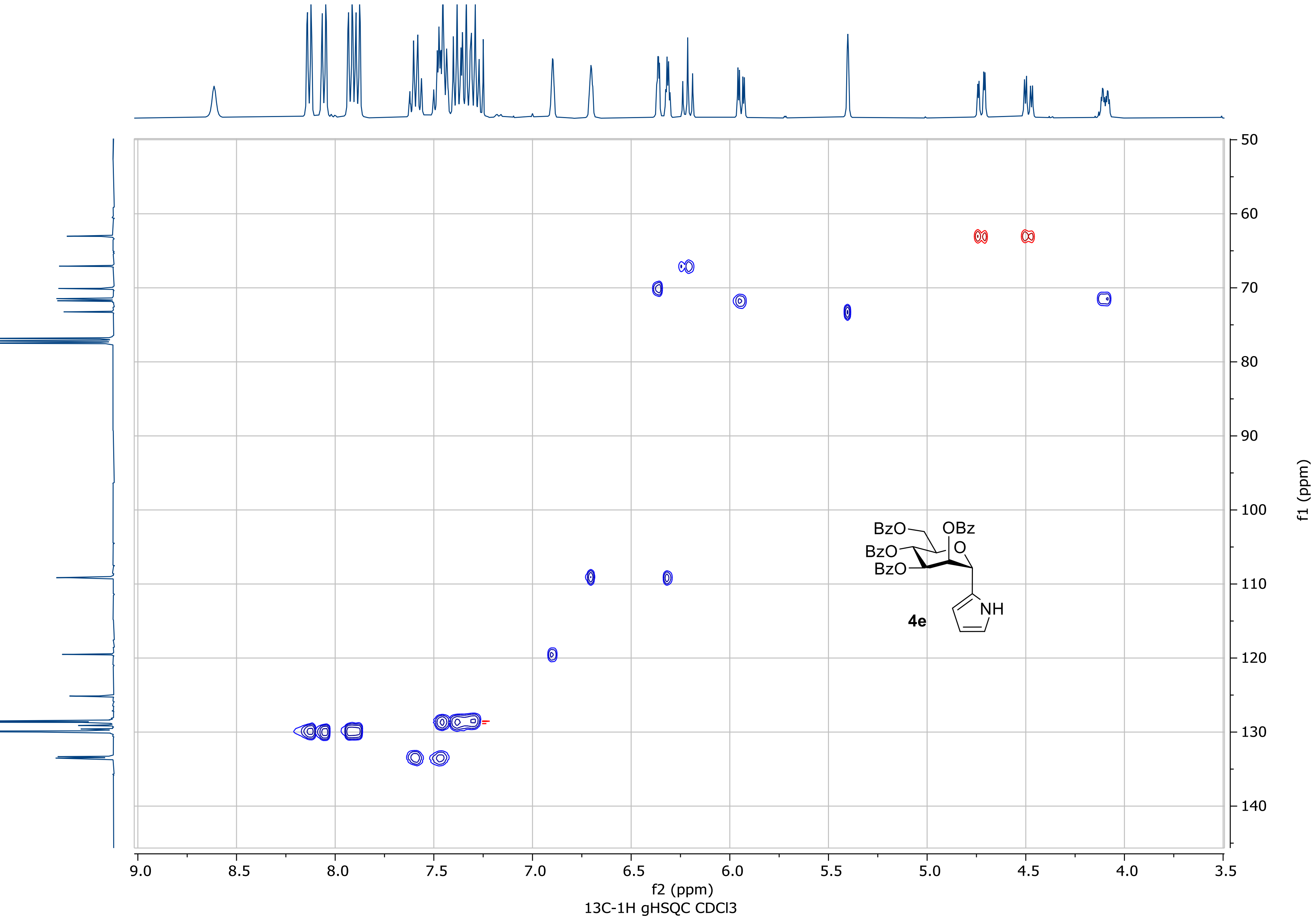

50
60
70
80
90
100
110
120
130
140
f1 (ppm)
BzO
OBz
O
BzO
BzO
NH
4e
9.0
8.5
8.0
7.5
7.0
6.5
6.0
5.5
5.0
4.5
4.0
3.5
f2 (ppm)
13C-1H gHSQC CDCl3

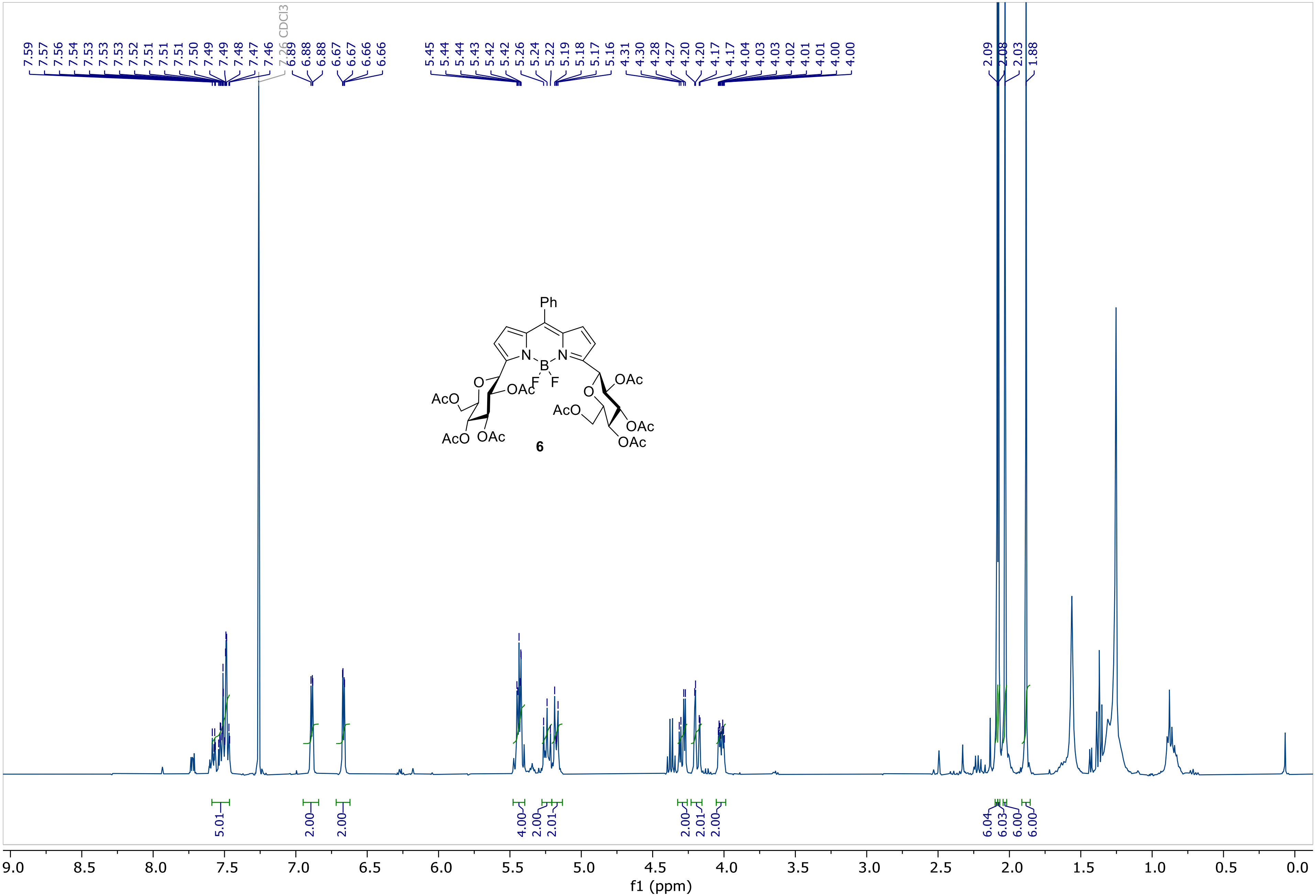

1H NMR (CDCl3, 400 MHz)

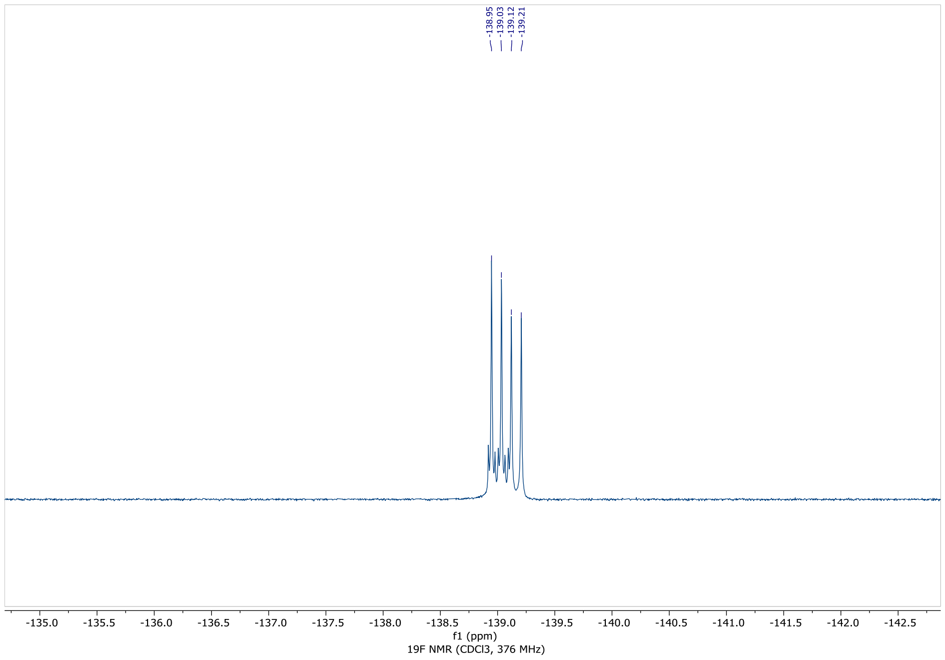
-138.95
-139.03
-139.12
-139.21
-135.0 -135.5 -136.0 -136.5 -137.0 -137.5 -138.0 -138.5 -139.0 -139.5 -140.0 -140.5 -141.0 -141.5 -142.0 -142.5
f1 (ppm)
19F NMR (CDCl3, 376 MHz)

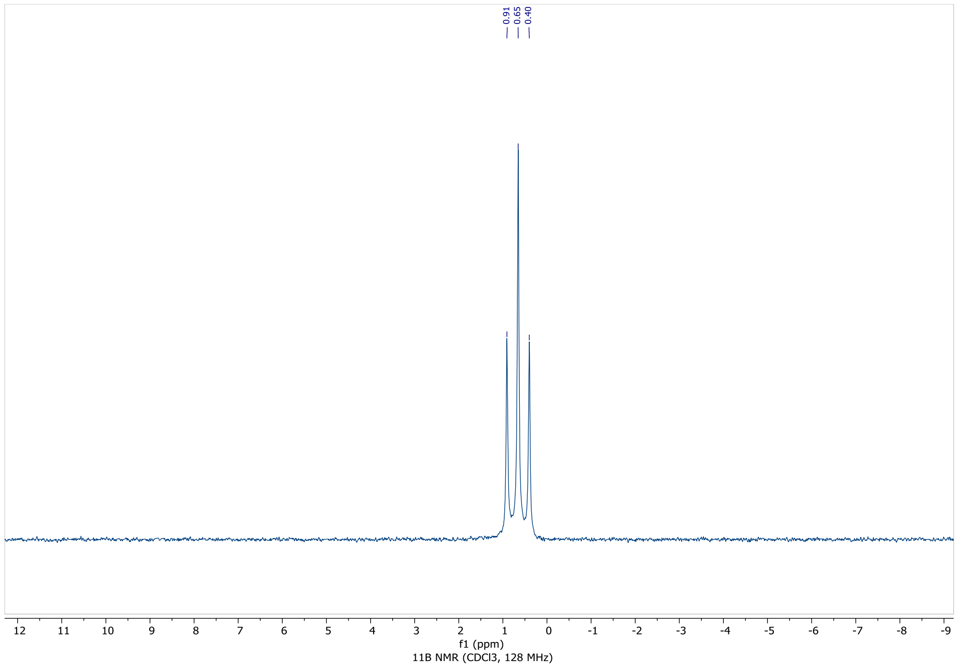
0.91
0.65
0.40
12 11 10 9 8 7 6 5 4 3 2 1 0 -1 -2 -3 -4 -5 -6 -7 -8 -9
f1 (ppm)
11B NMR (CDCl3, 128 MHz)

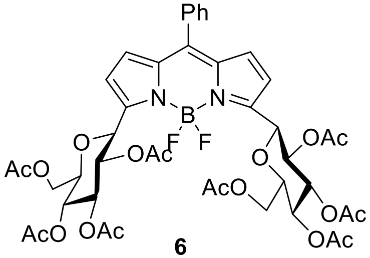
Ph
N
B
N
F
F
O
OAc
AcO
AcO
OAc
OAc
O
AcO
OAc
OAc

6

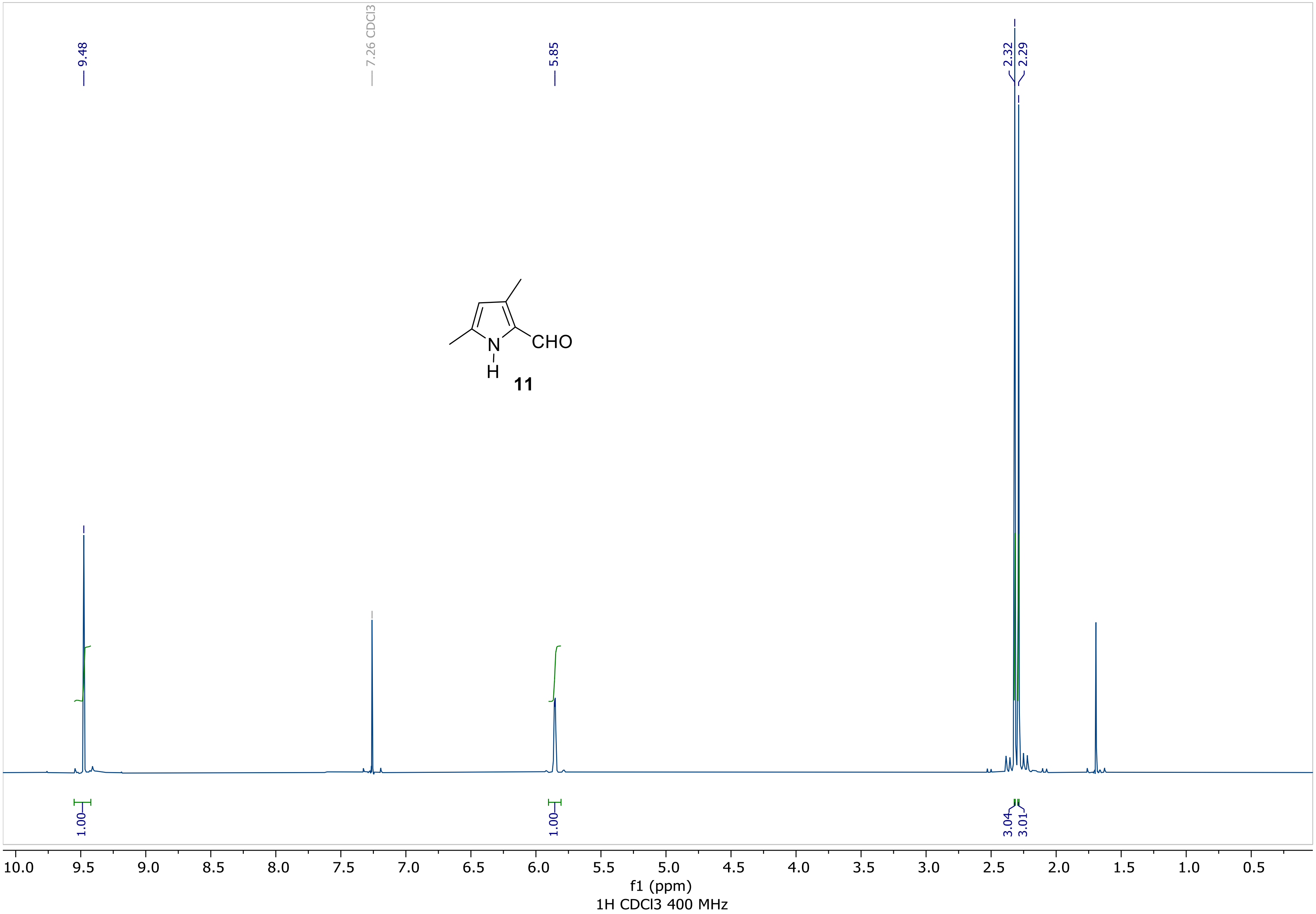
9.48
7.26 CDCl3
5.85
2.32
2.29
CHO
N
H
11
1.00
1.00
3.04
3.01
10.0
9.5
9.0
8.5
8.0
7.5
7.0
6.5
6.0
5.5
5.0
4.5
4.0
3.5
3.0
2.5
2.0
1.5
1.0
0.5
f1 (ppm)
1H CDCl3 400 MHz

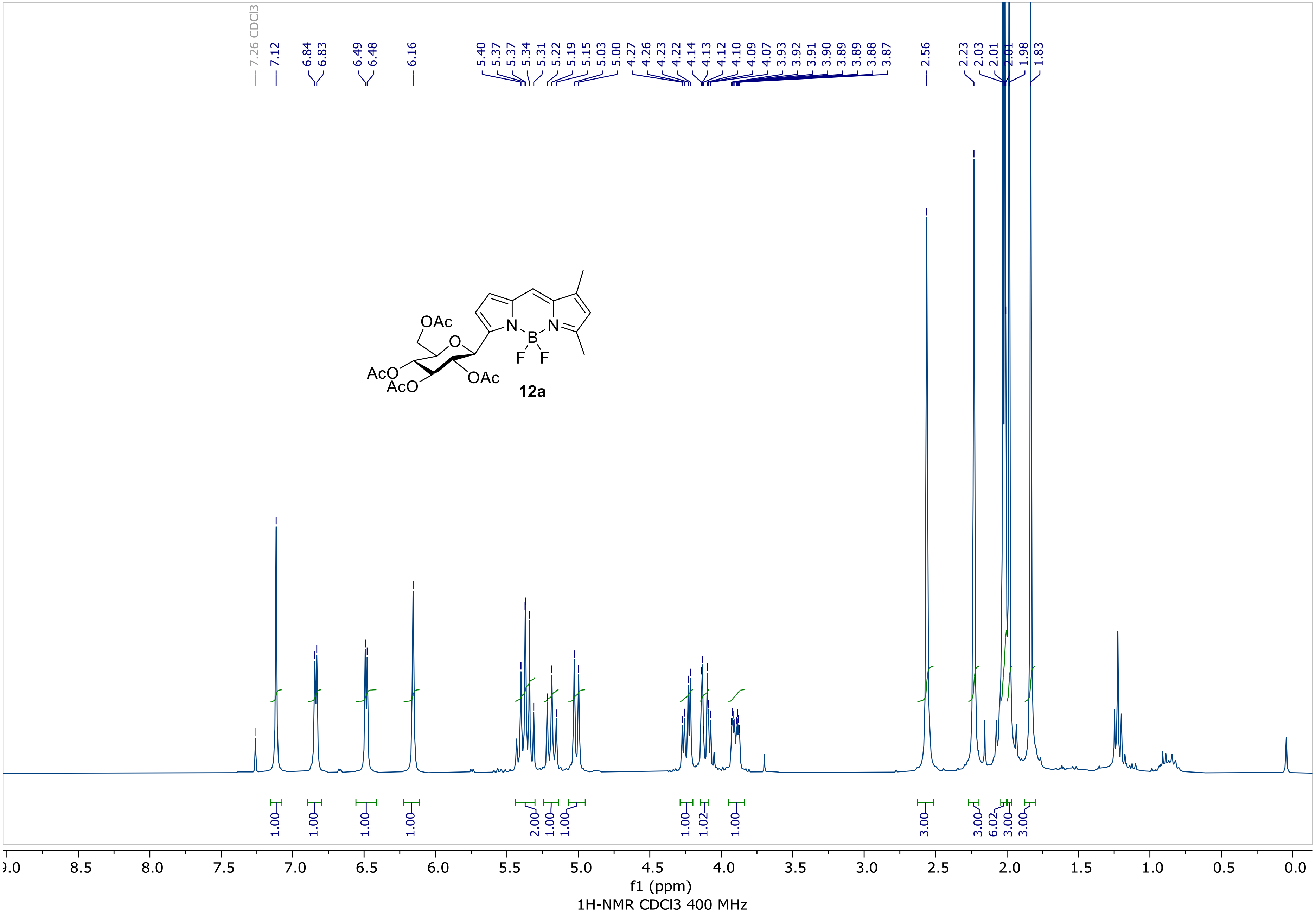
7.26 CDCl3
7.12
6.84
6.83
6.49
6.48
6.16
5.40
5.37
5.37
5.34
5.31
5.22
5.19
5.15
5.03
5.00
4.27
4.26
4.23
4.22
4.14
4.13
4.12
4.10
4.09
4.07
3.93
3.92
3.91
3.90
3.89
3.89
3.88
3.87
2.56
2.23
2.03
2.01
2.01
1.98
1.83
OAc
O
N
B
N
F
F
AcO
AcO
OAc
12a
1.00
1.00
1.00
1.00
2.00
1.00
1.00
1.00
1.02
1.00
3.00
3.00
6.02
3.00
3.00
f1 (ppm)
1H-NMR CDCl3 400 MHz

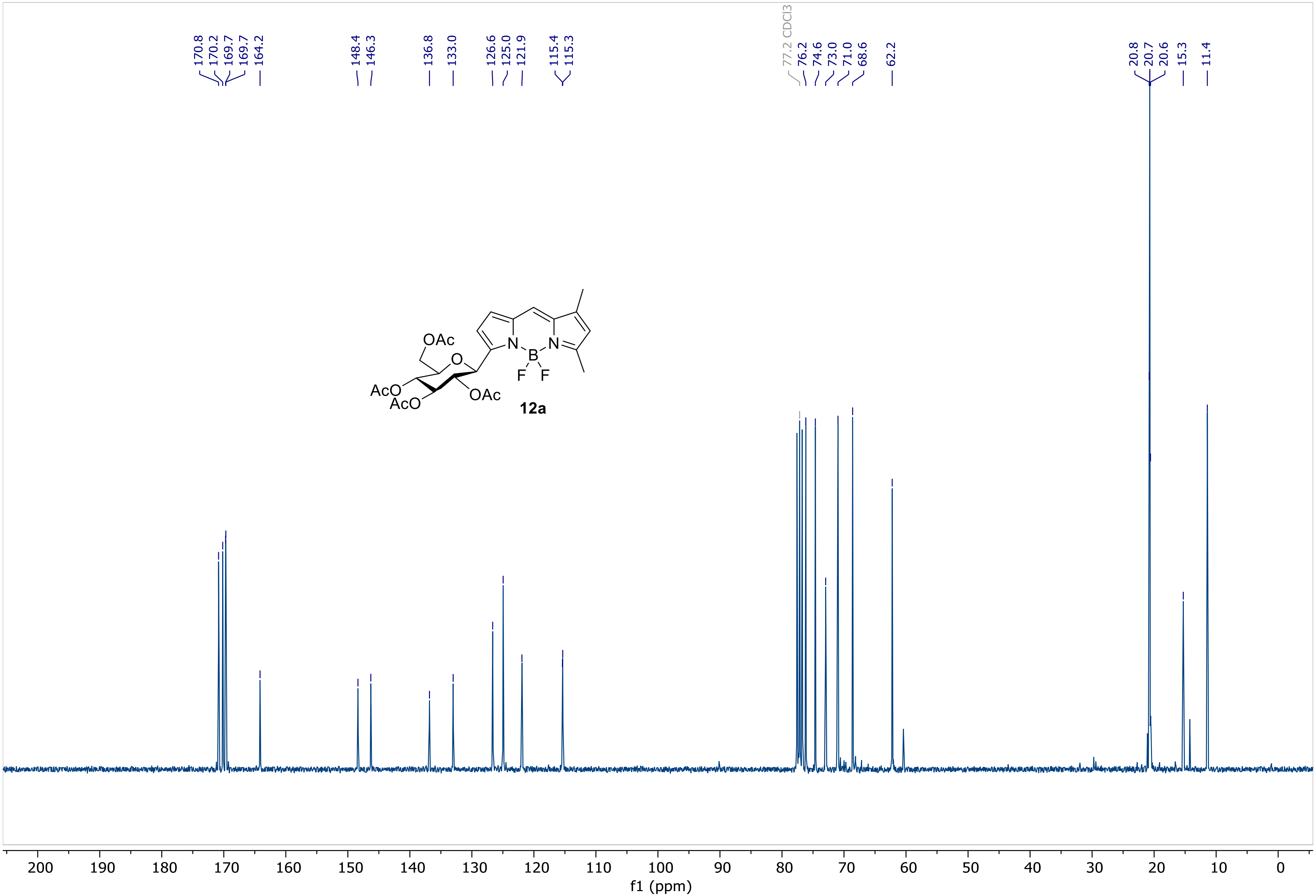

13C {1H}-NMR CDCl3 400 MHz

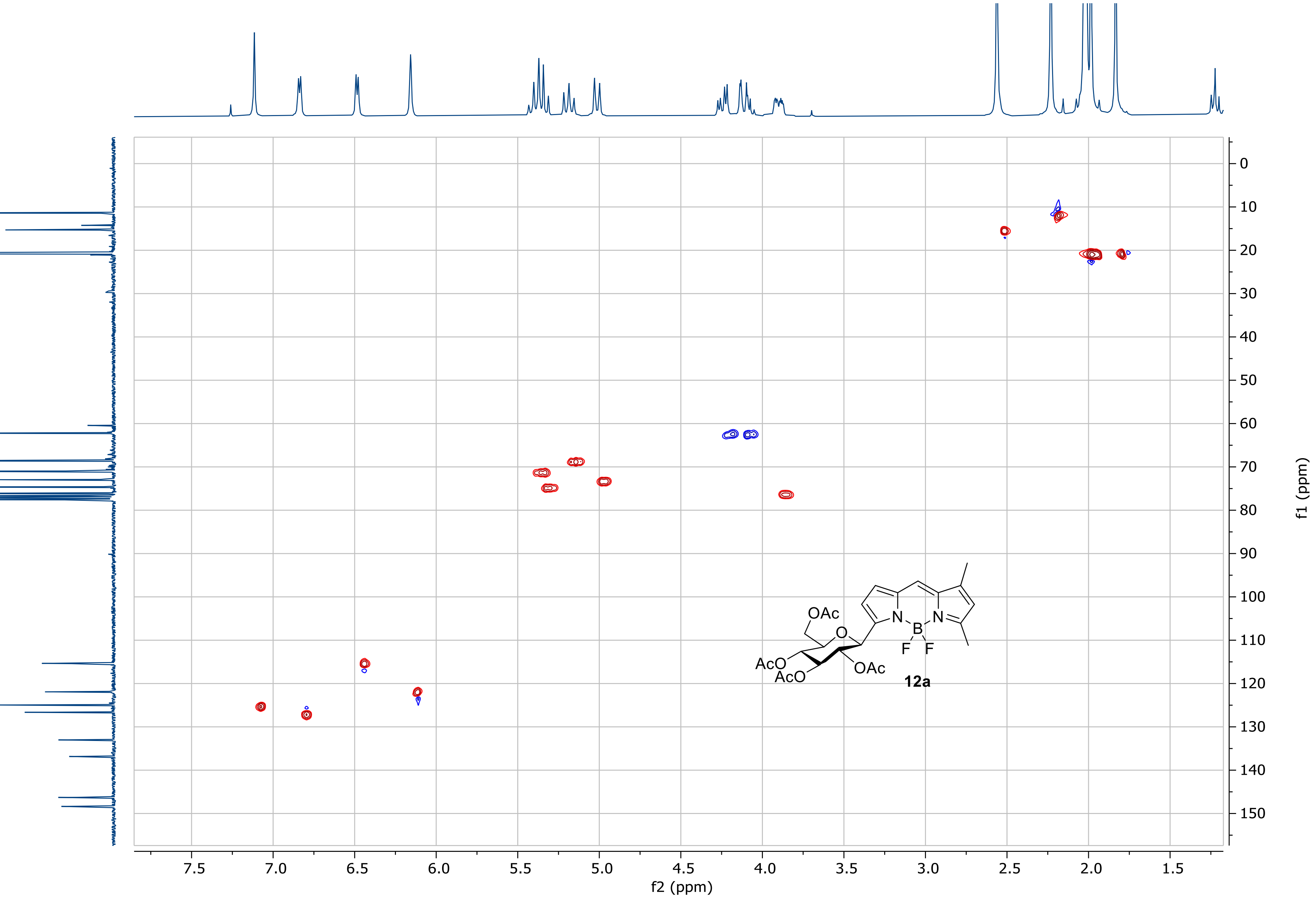

1H-13C gHSQC CDCl3

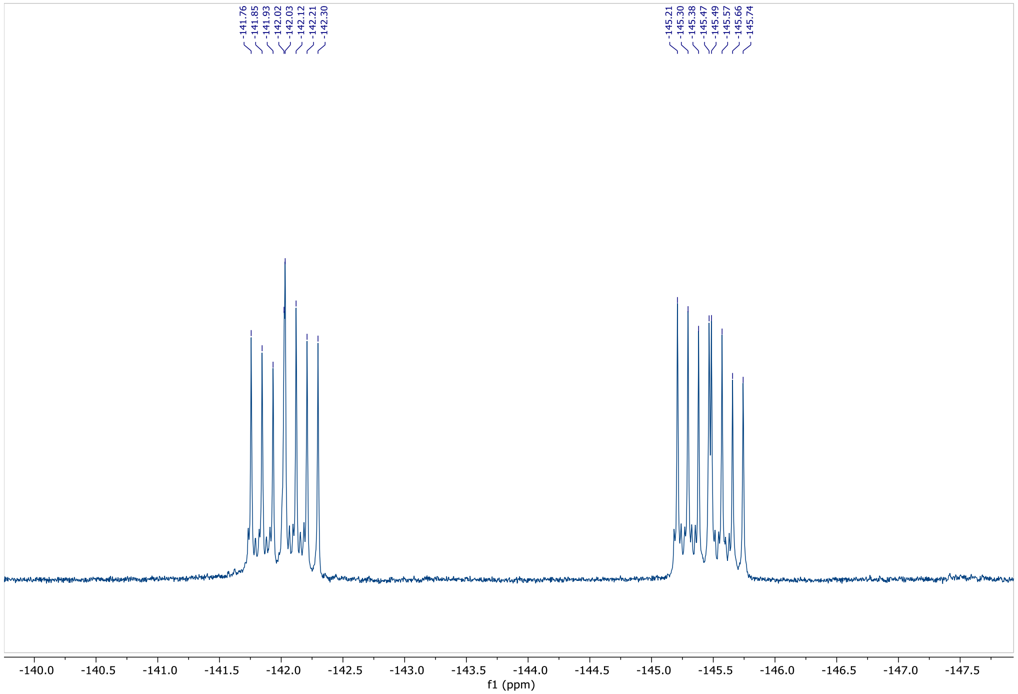

19F NMR (CDCl3, 376 MHz)

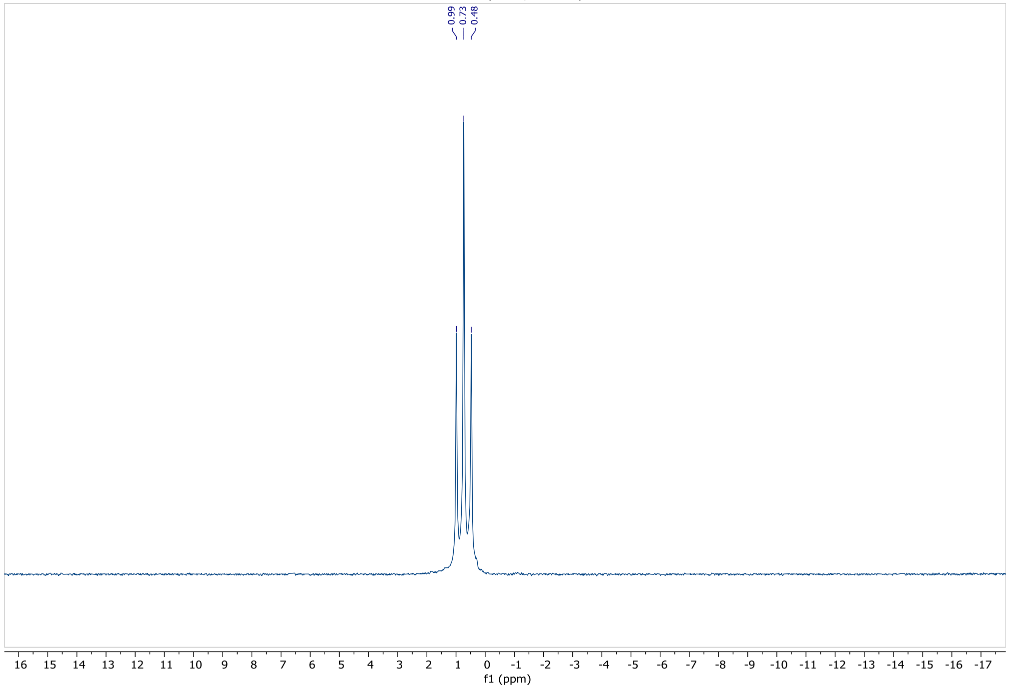

11B NMR (CDCl3, 128 MHz)

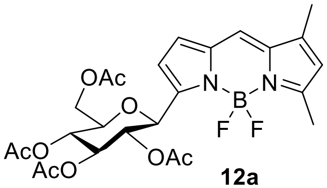

12a

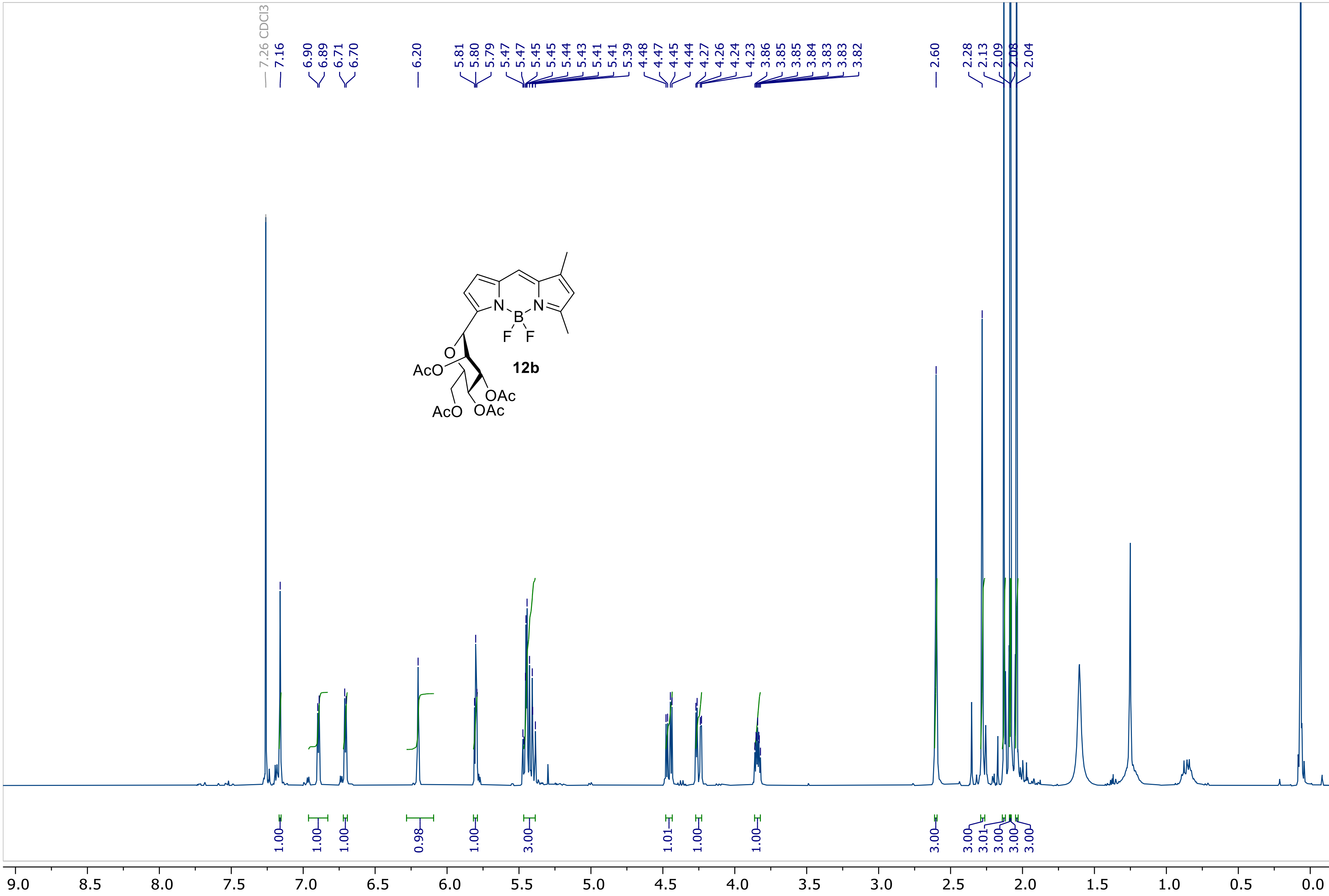

1H-NMR CDCl3 400MHz

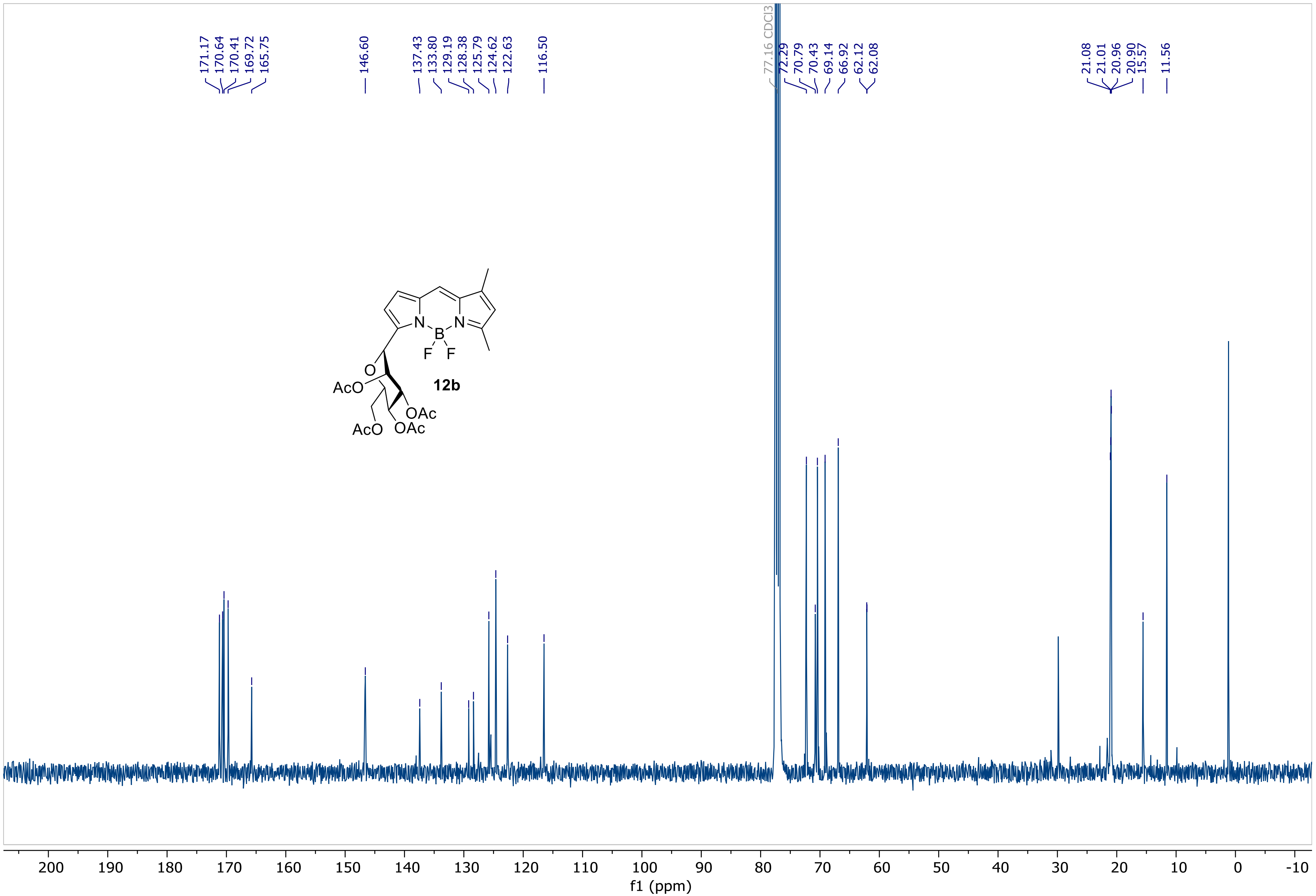

13C{1H} NMR CDCl3 125 MHz

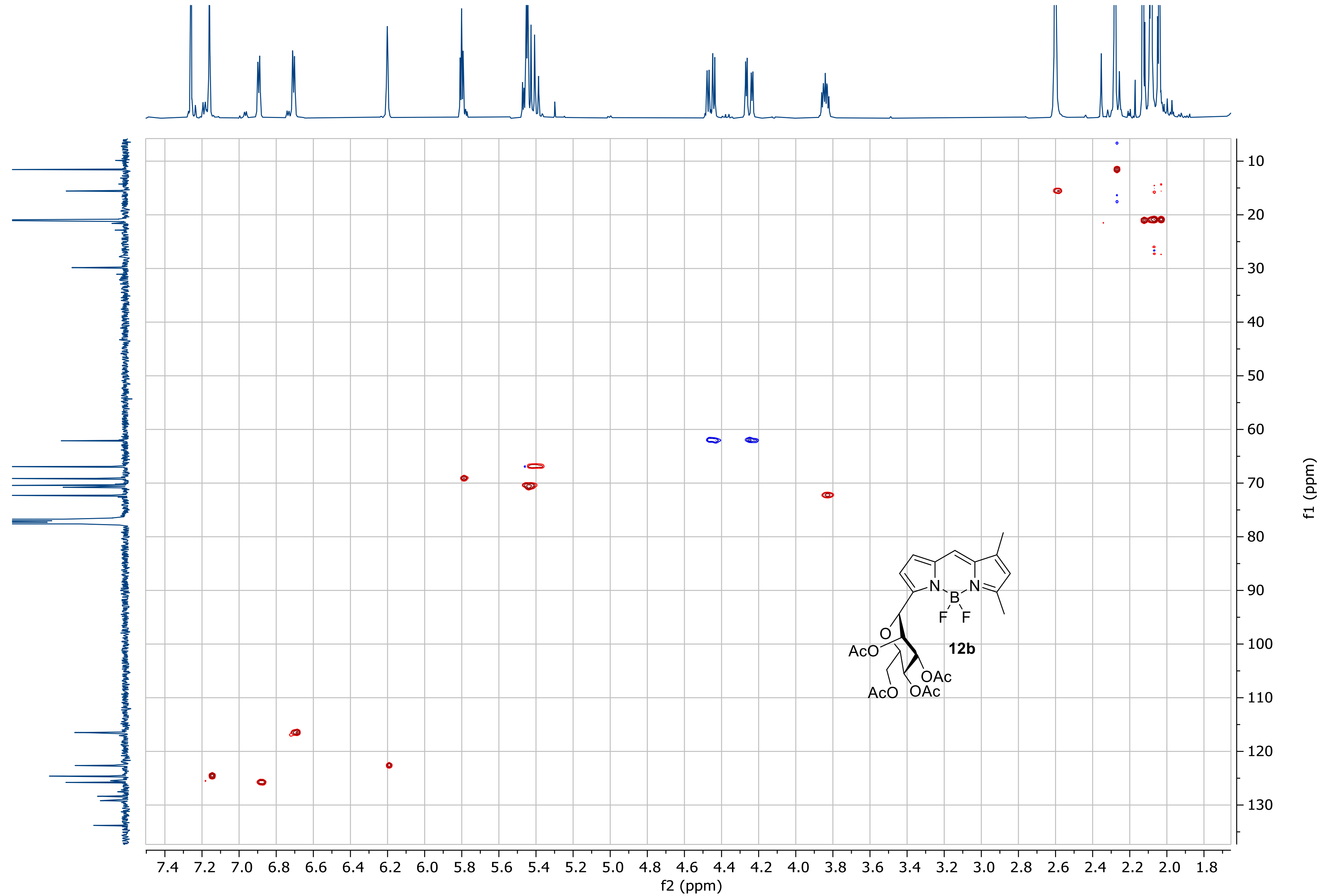

1H-13C gHSQC

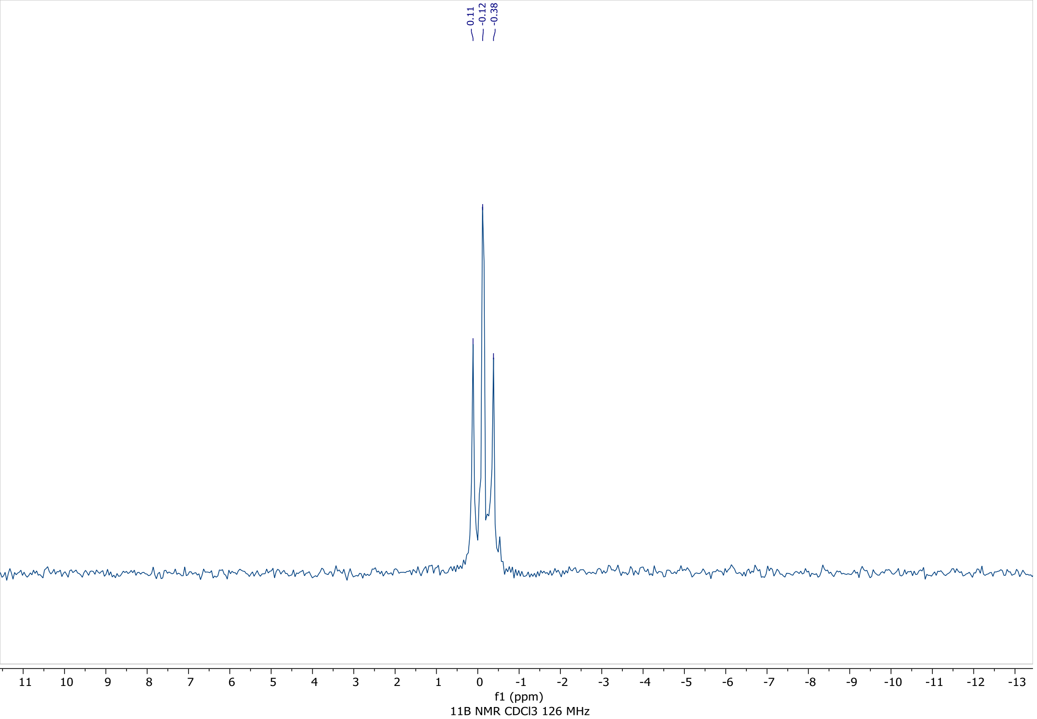
0.11
-0.12
-0.38
11 10 9 8 7 6 5 4 3 2 1 0 -1 -2 -3 -4 -5 -6 -7 -8 -9 -10 -11 -12 -13
f1 (ppm)
11B NMR CDCl3 126 MHz

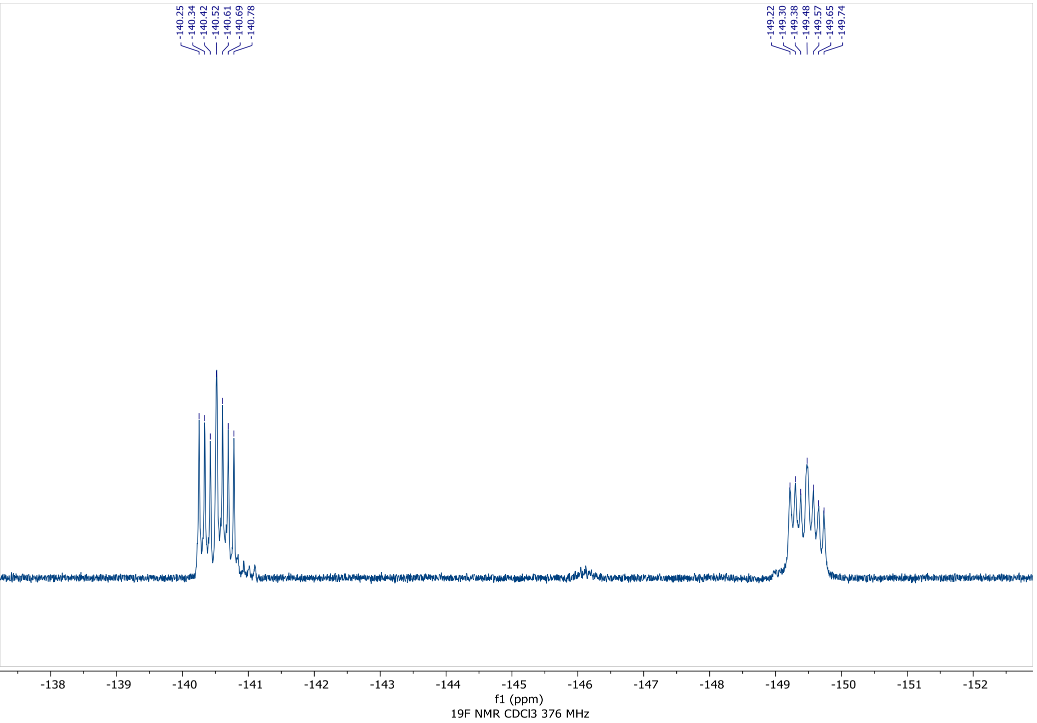
-140.25
-140.34
-140.42
-140.52
-140.61
-140.69
-140.78
-149.22
-149.30
-149.38
-149.48
-149.57
-149.65
-149.74
-138 -139 -140 -141 -142 -143 -144 -145 -146 -147 -148 -149 -150 -151 -152
f1 (ppm)
19F NMR CDCl3 376 MHz

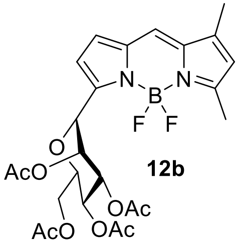
N
B
N
F
F
O
AcO
OAc
AcO
OAc

12b

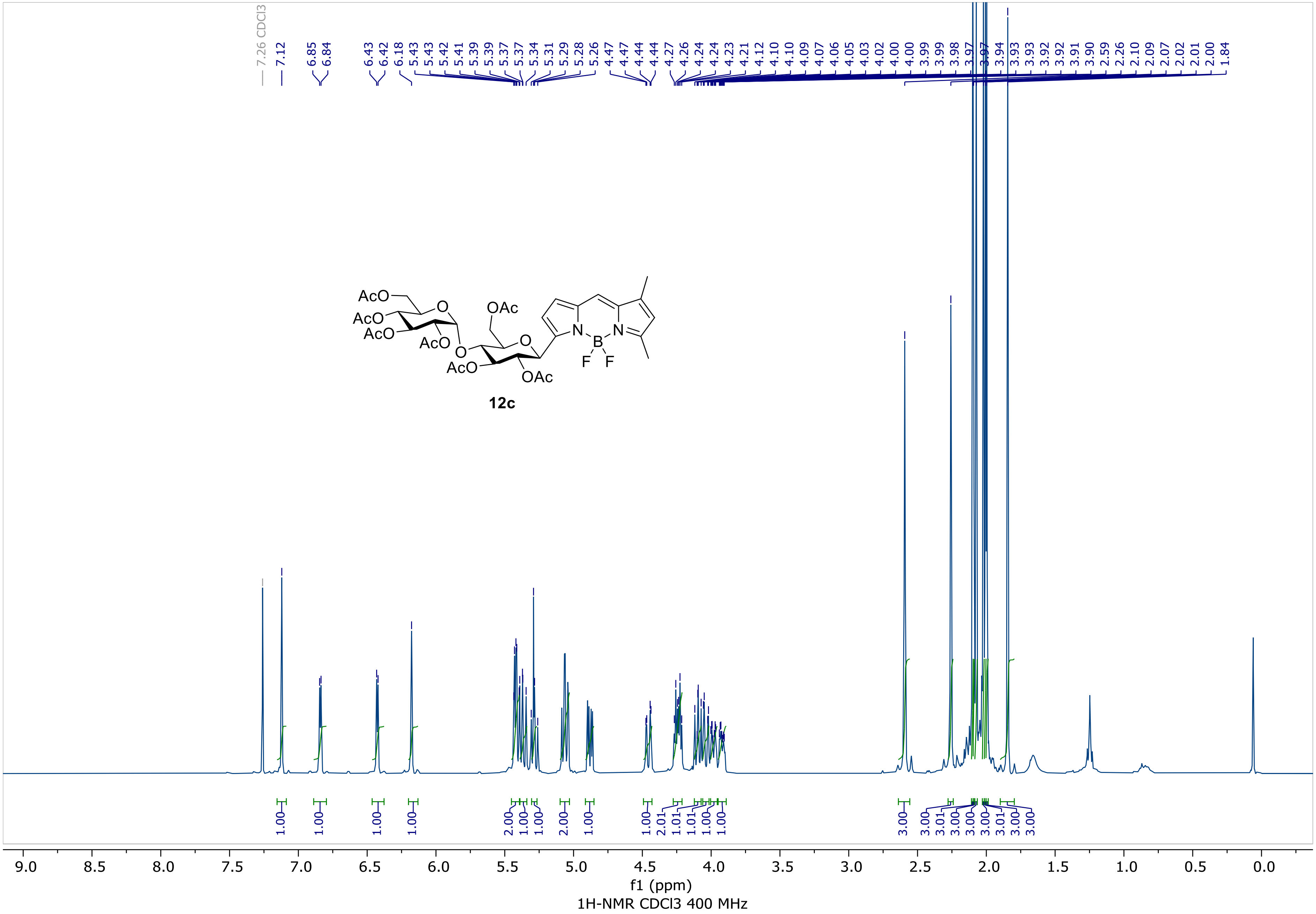

12c
f1 (ppm)
1H-NMR CDCl3 400 MHz

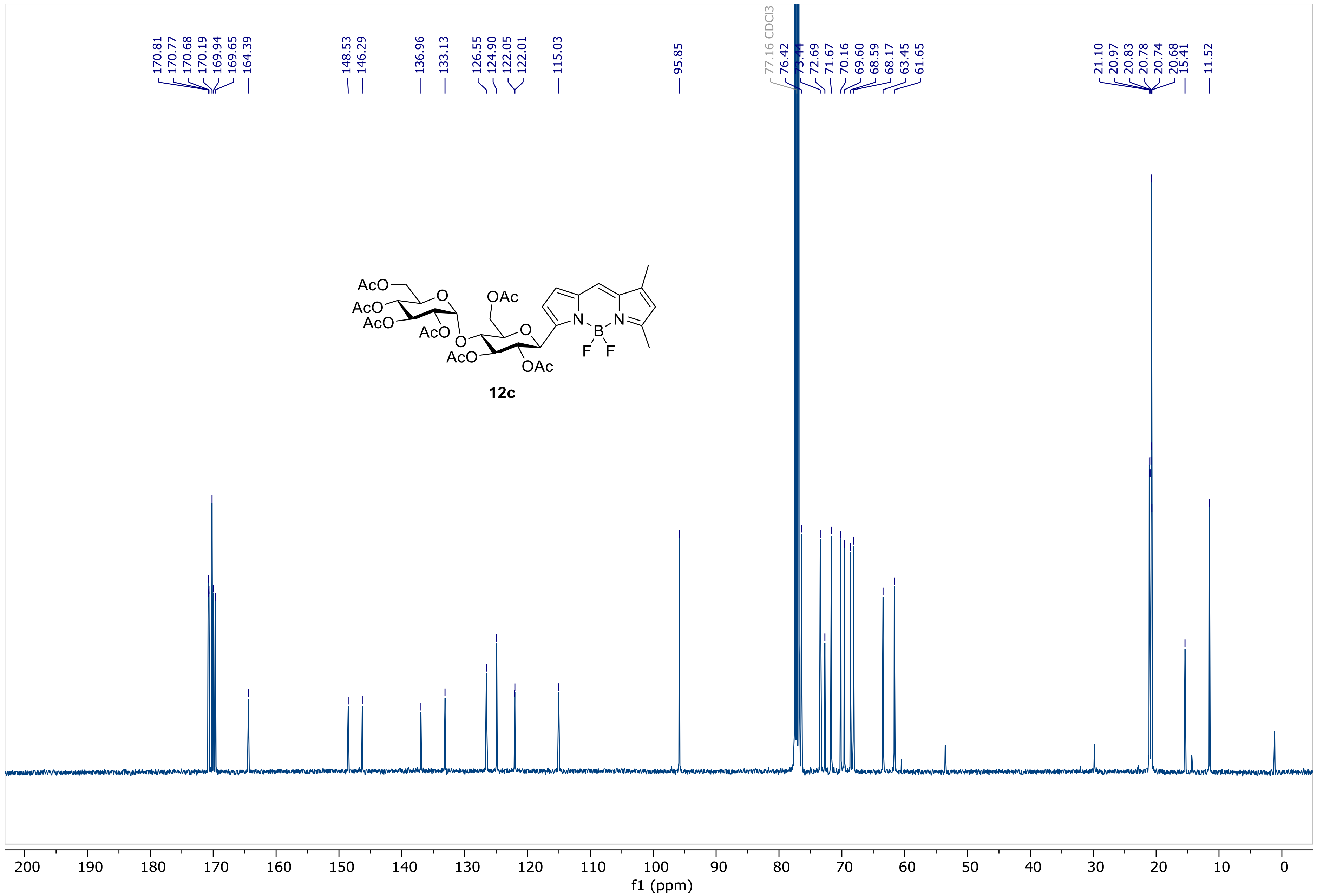

13C{1H} NMR CDCl3 125 MHz

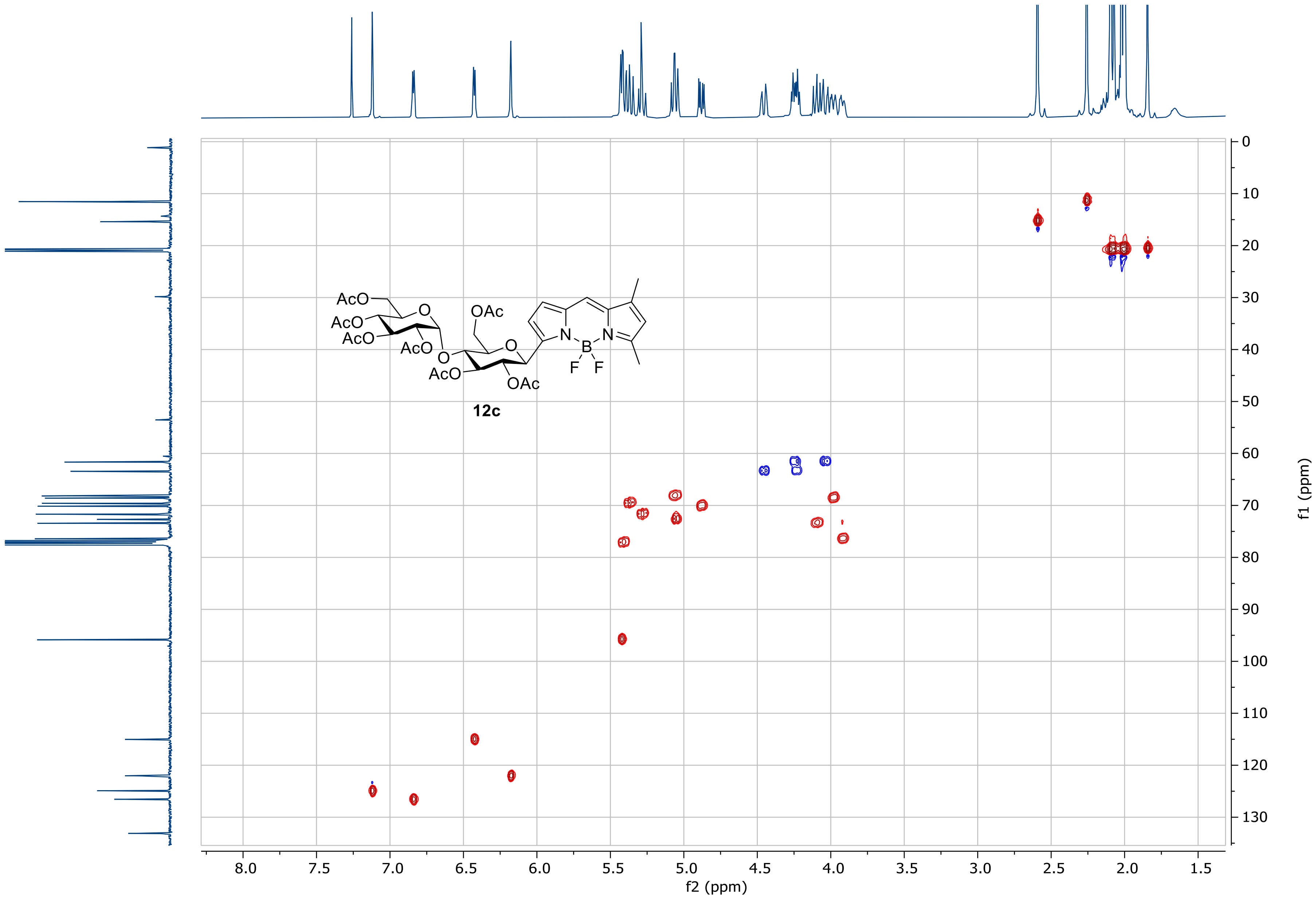

1H-13C gHSQC CCDCl3

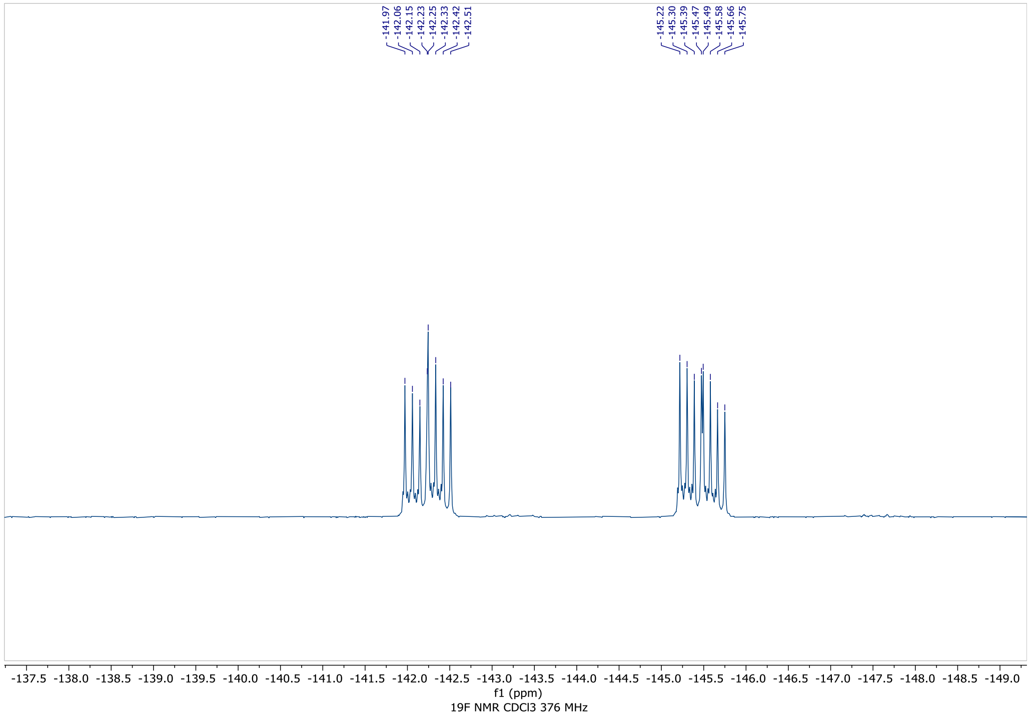
-141.97
-142.06
-142.15
-142.23
-142.25
-142.33
-142.42
-142.51
-145.22
-145.30
-145.39
-145.47
-145.49
-145.58
-145.66
-145.75
-137.5 -138.0 -138.5 -139.0 -139.5 -140.0 -140.5 -141.0 -141.5 -142.0 -142.5 -143.0 -143.5 -144.0 -144.5 -145.0 -145.5 -146.0 -146.5 -147.0 -147.5 -148.0 -148.5 -149.0
f1 (ppm)
19F NMR CDCl3 376 MHz

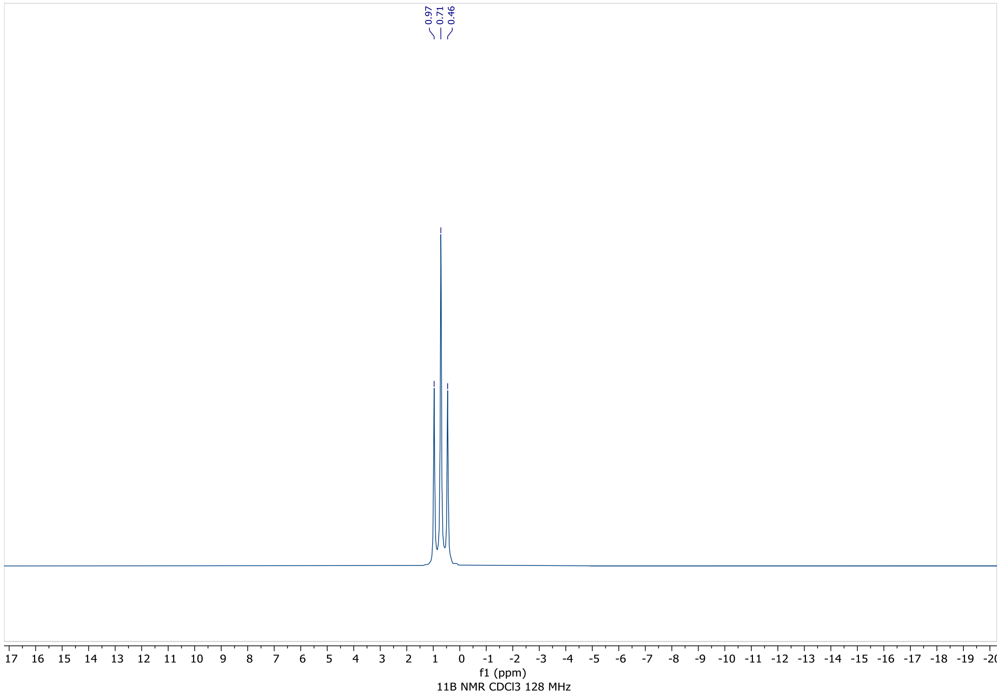
0.97
0.71
0.46
17 16 15 14 13 12 11 10 9 8 7 6 5 4 3 2 1 0 -1 -2 -3 -4 -5 -6 -7 -8 -9 -10 -11 -12 -13 -14 -15 -16 -17 -18 -19
f1 (ppm)
11B NMR CDCl3 128 MHz

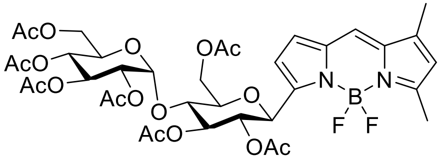
AcO
AcO
AcO
O
AcO
O
OAc
O
AcO
OAc
N
B
N
F
F

12c

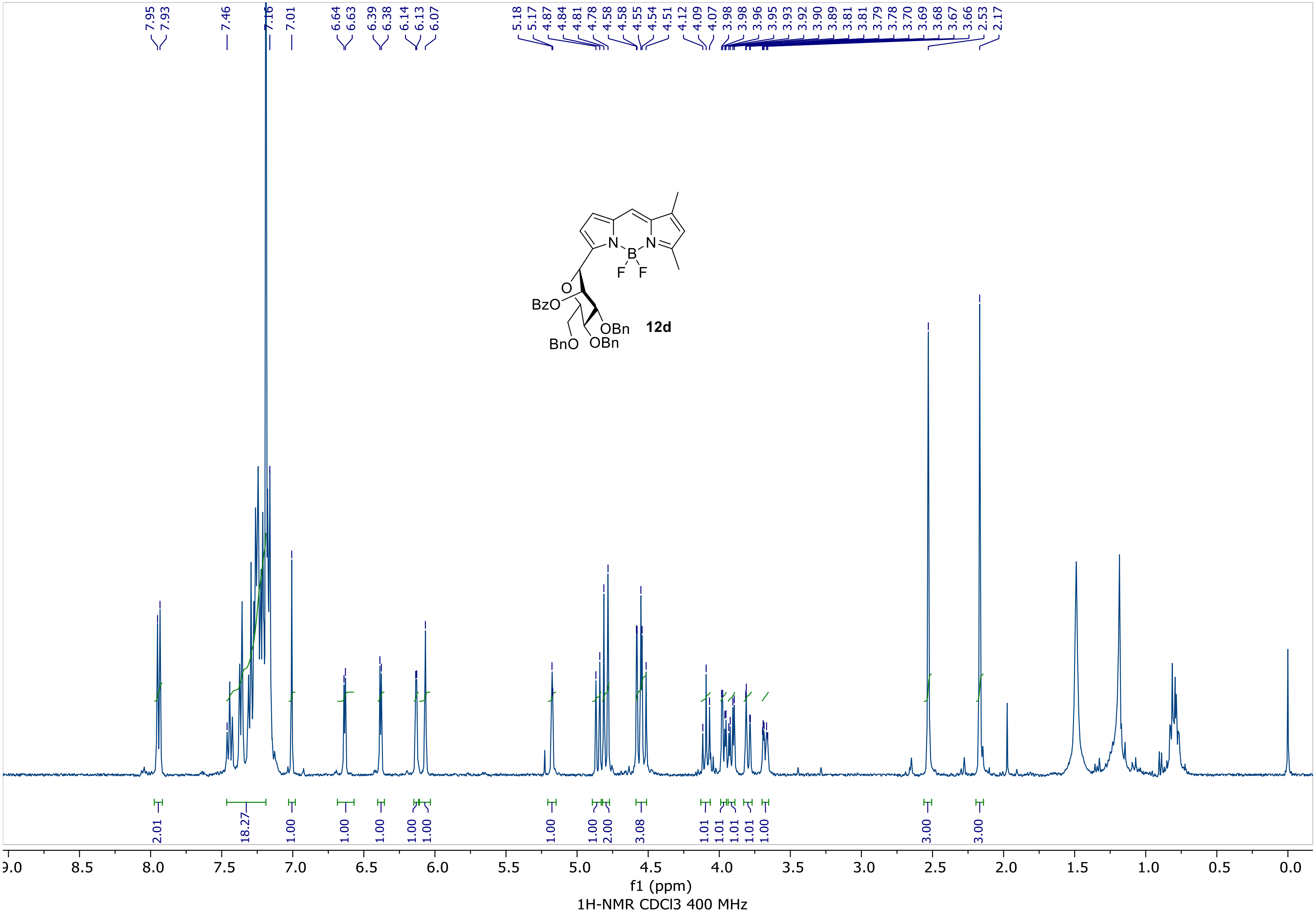
7.95 7.93 7.46 7.16 7.01 6.64 6.63 6.39 6.38 6.14 6.13 6.07 5.18 5.17 4.87 4.84 4.81 4.78 4.58 4.58 4.55 4.54 4.51 4.12 4.09 4.07 3.98 3.98 3.96 3.95 3.93 3.92 3.90 3.89 3.81 3.81 3.79 3.78 3.70 3.69 3.68 3.67 3.66 2.53 2.17
N B N F F O BzO OBn OBn BnO
12d
2.01 18.27 1.00 1.00 1.00 1.00 1.00 1.00 1.00 2.00 3.08 1.01 1.01 1.01 1.01 1.00 3.00 3.00
9.0 8.5 8.0 7.5 7.0 6.5 6.0 5.5 5.0 4.5 4.0 3.5 3.0 2.5 2.0 1.5 1.0 0.5 0.0
f1 (ppm)
1H-NMR CDCl3 400 MHz

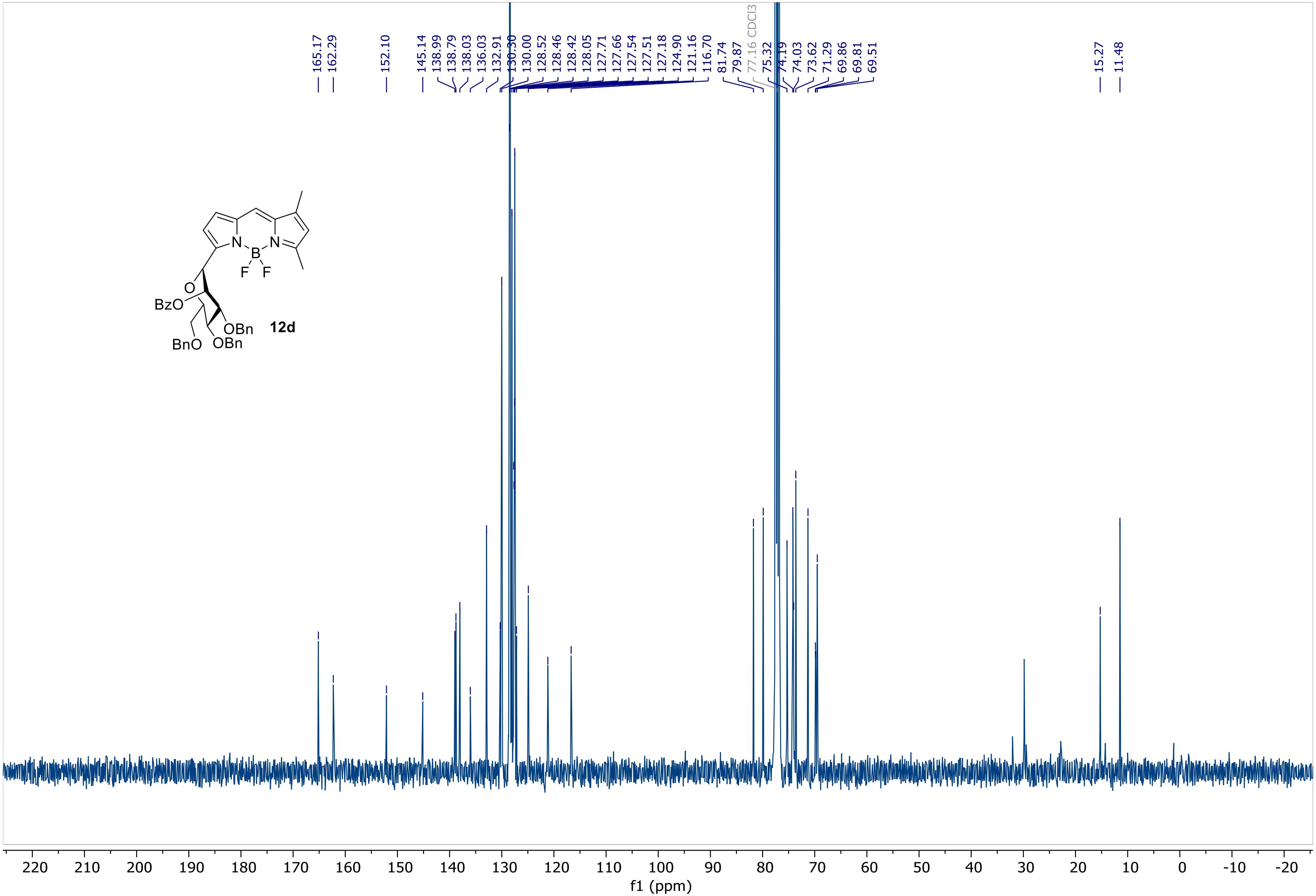

13C {1H} NMR CDCl3 125 MHz

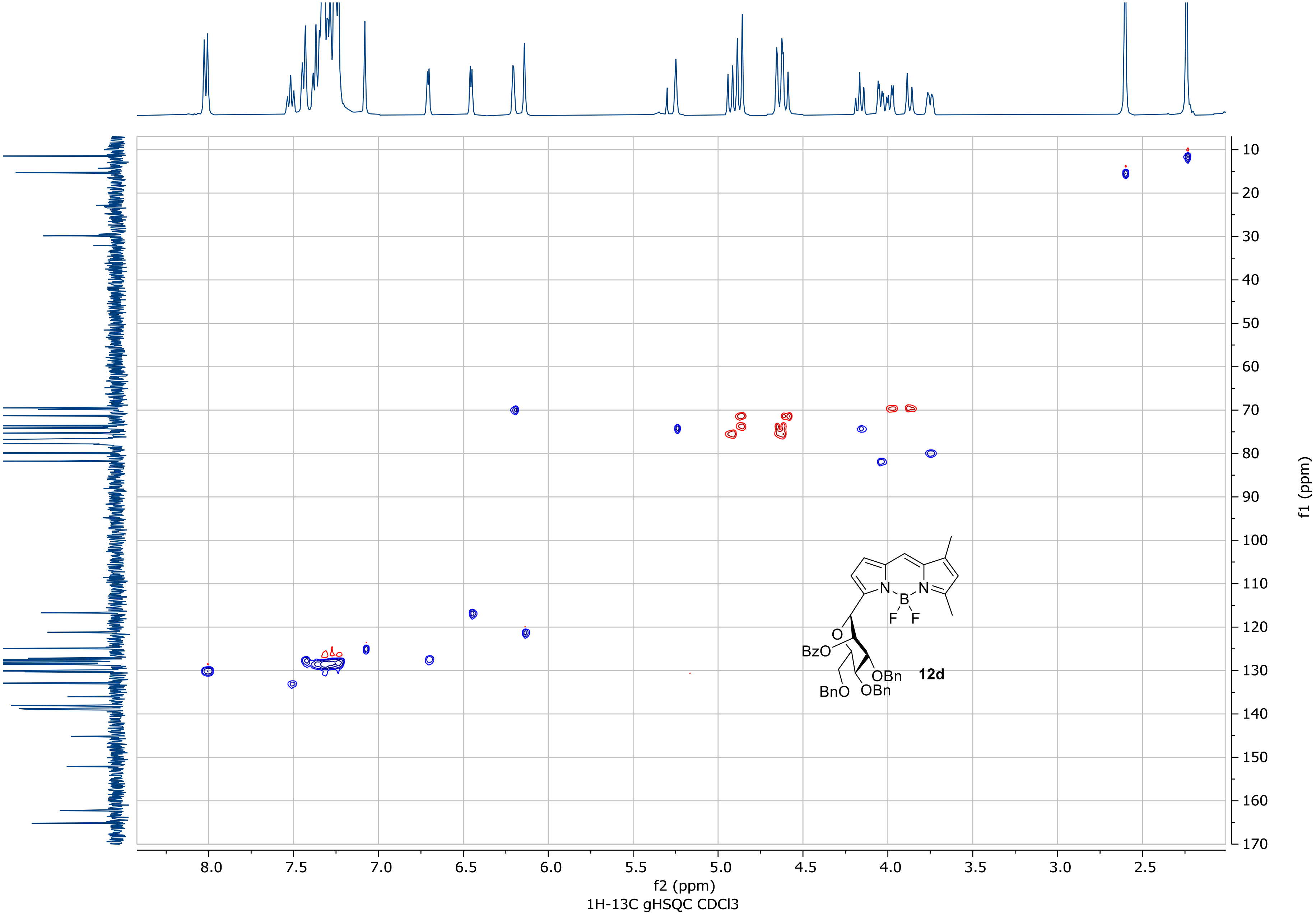

BzO
BnO
OBn
OBn
12d
f2 (ppm)
f1 (ppm)
1H-13C gHSQC CDCl3

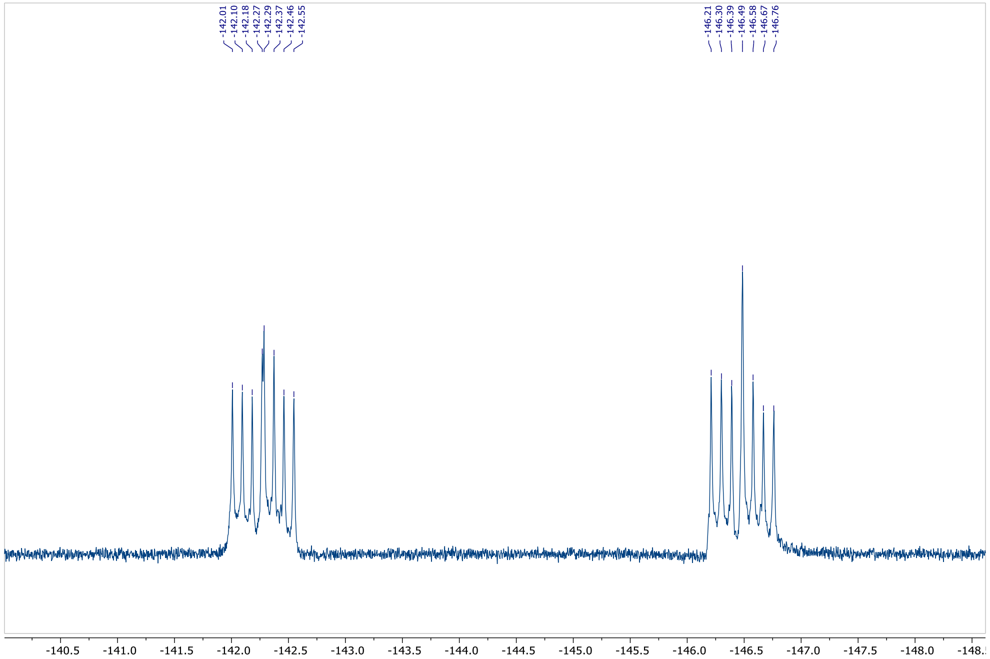

f1 (ppm)
19F NMR (CDCl3, 376 MHz)

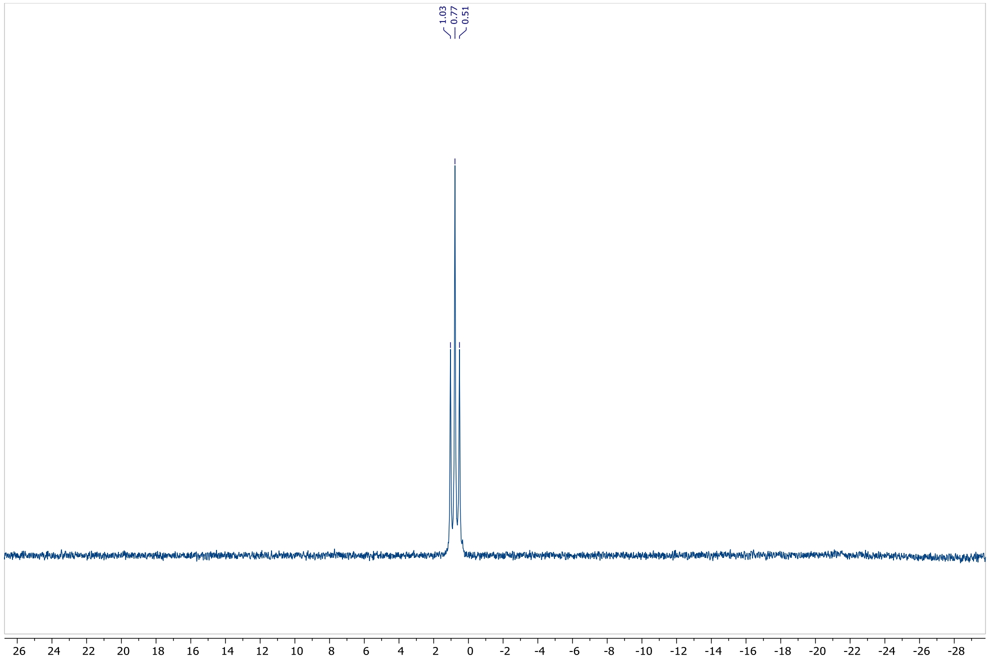

f1 (ppm)
11B NMR (128 MHz, CDCl3)

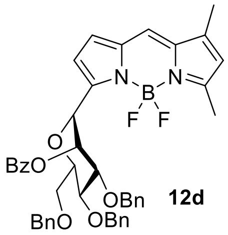

12d

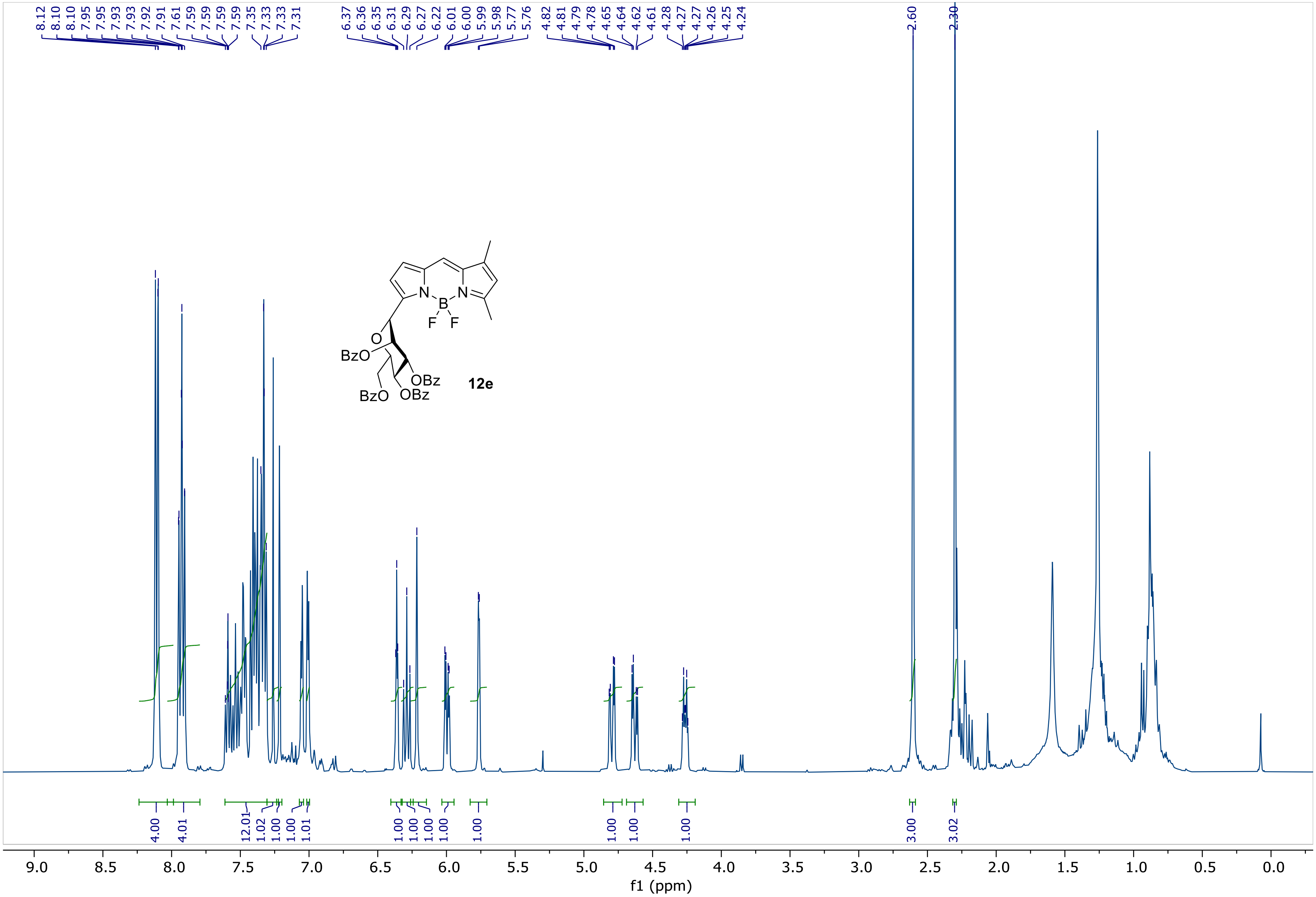

1H NMR CDCl3 400 MHz

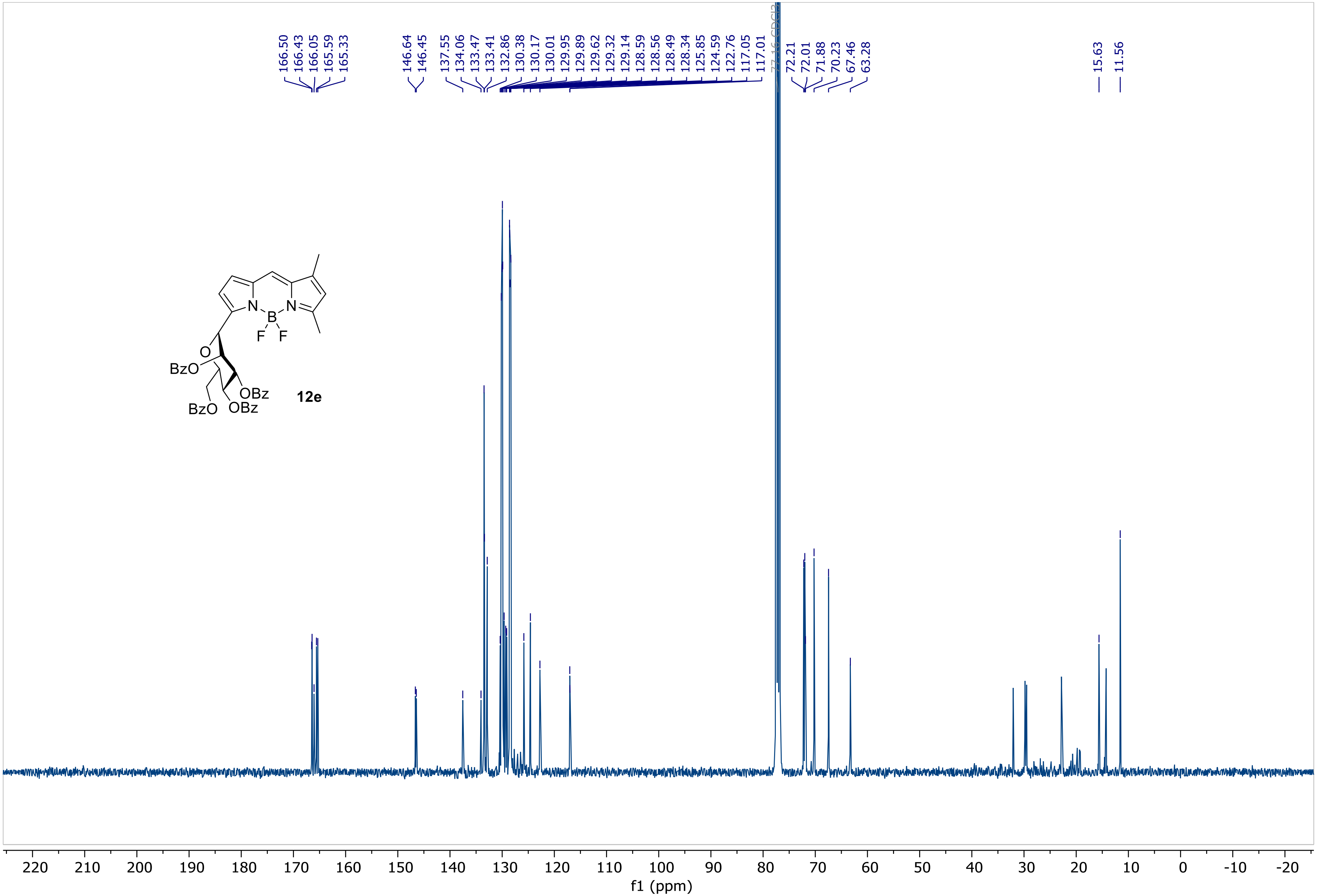

13C{1H} nmr CDCl3 125 MHz

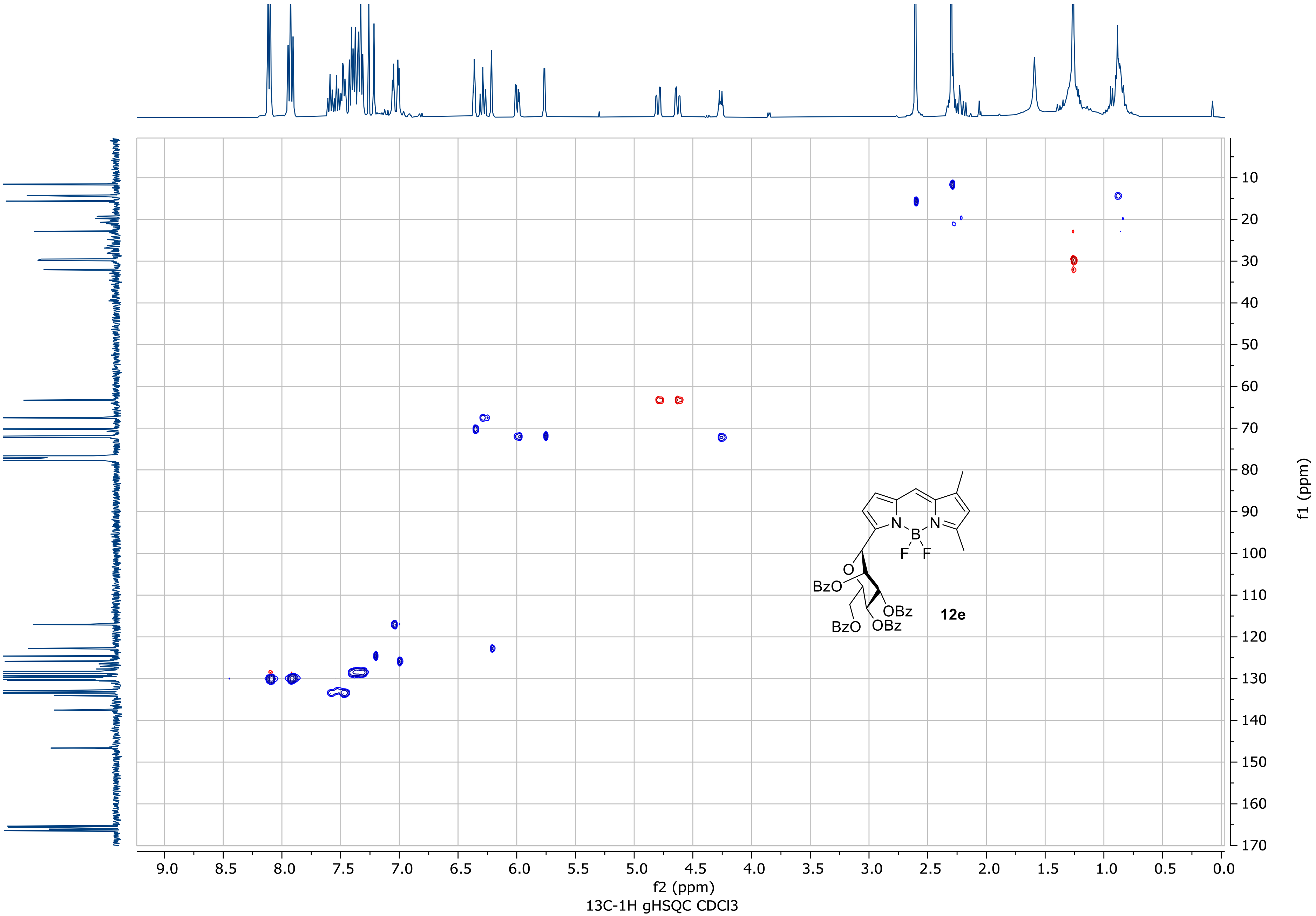

N
B
N
F
F
O
BzO
OBz
BzO
OBz
12e
f1 (ppm)
f2 (ppm)
13C-1H gHSQC CDCl3

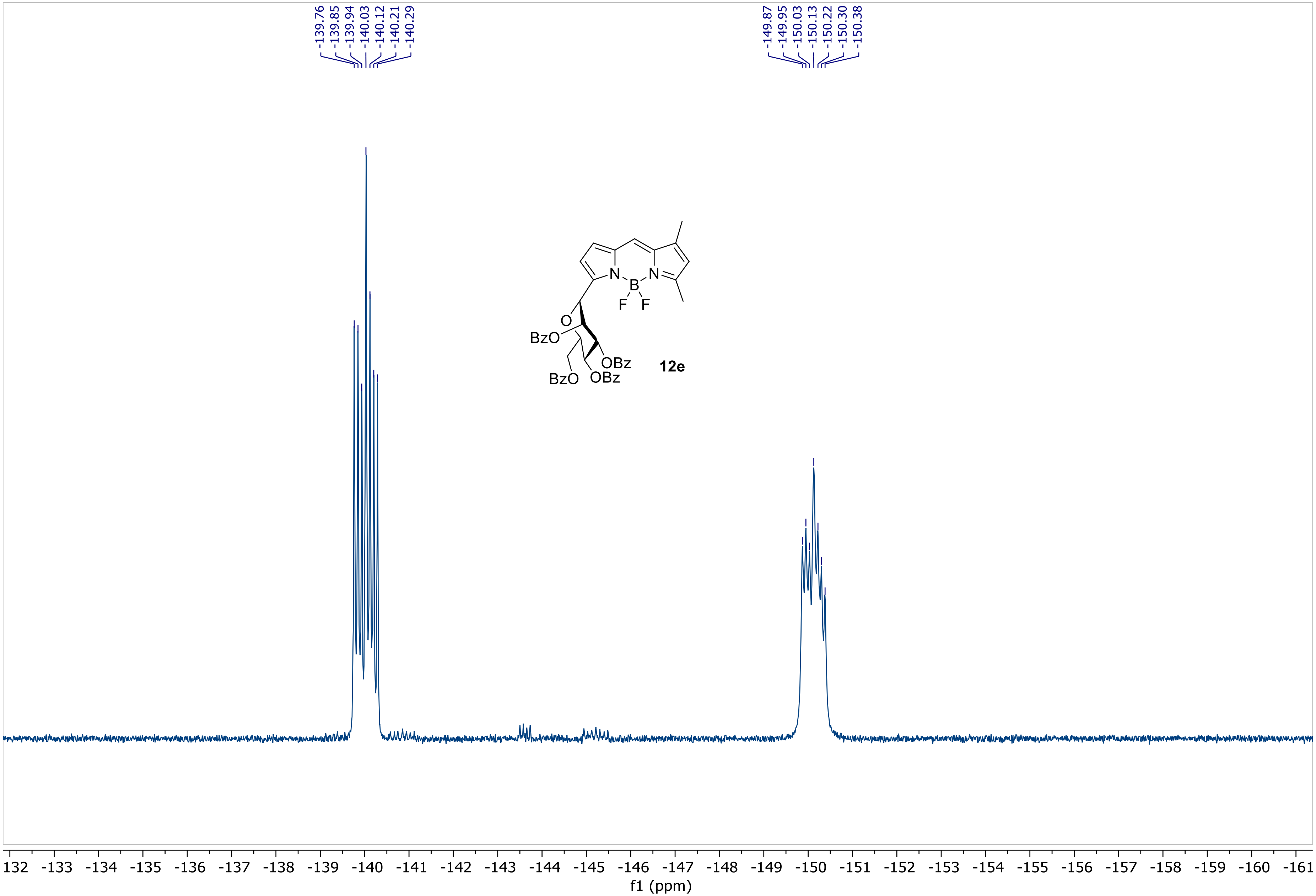

19F NMR CDCl3 376 MHz

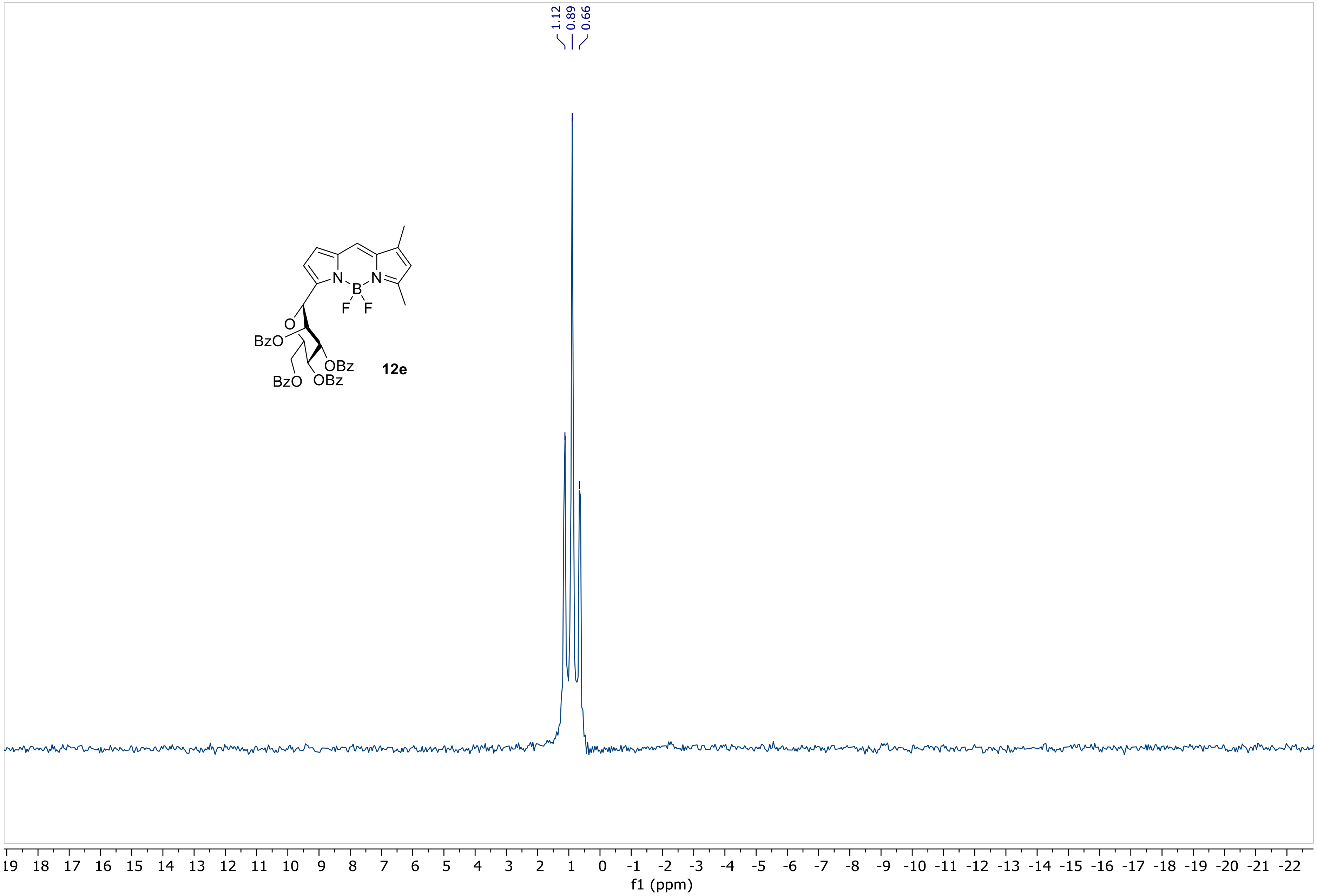

11B NMR CDCl3 128 MHz

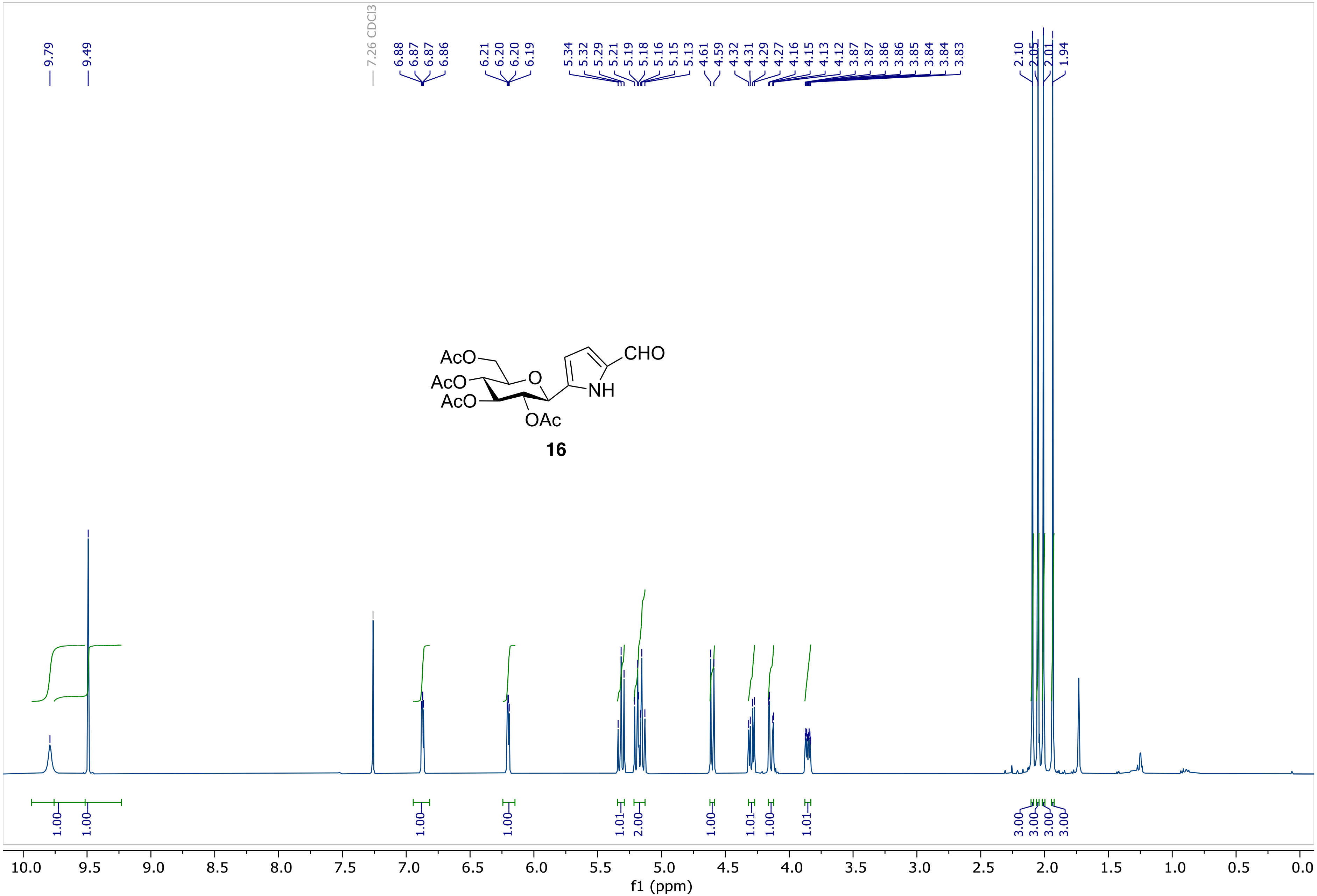

1H NMR CDCl3 400 MHz

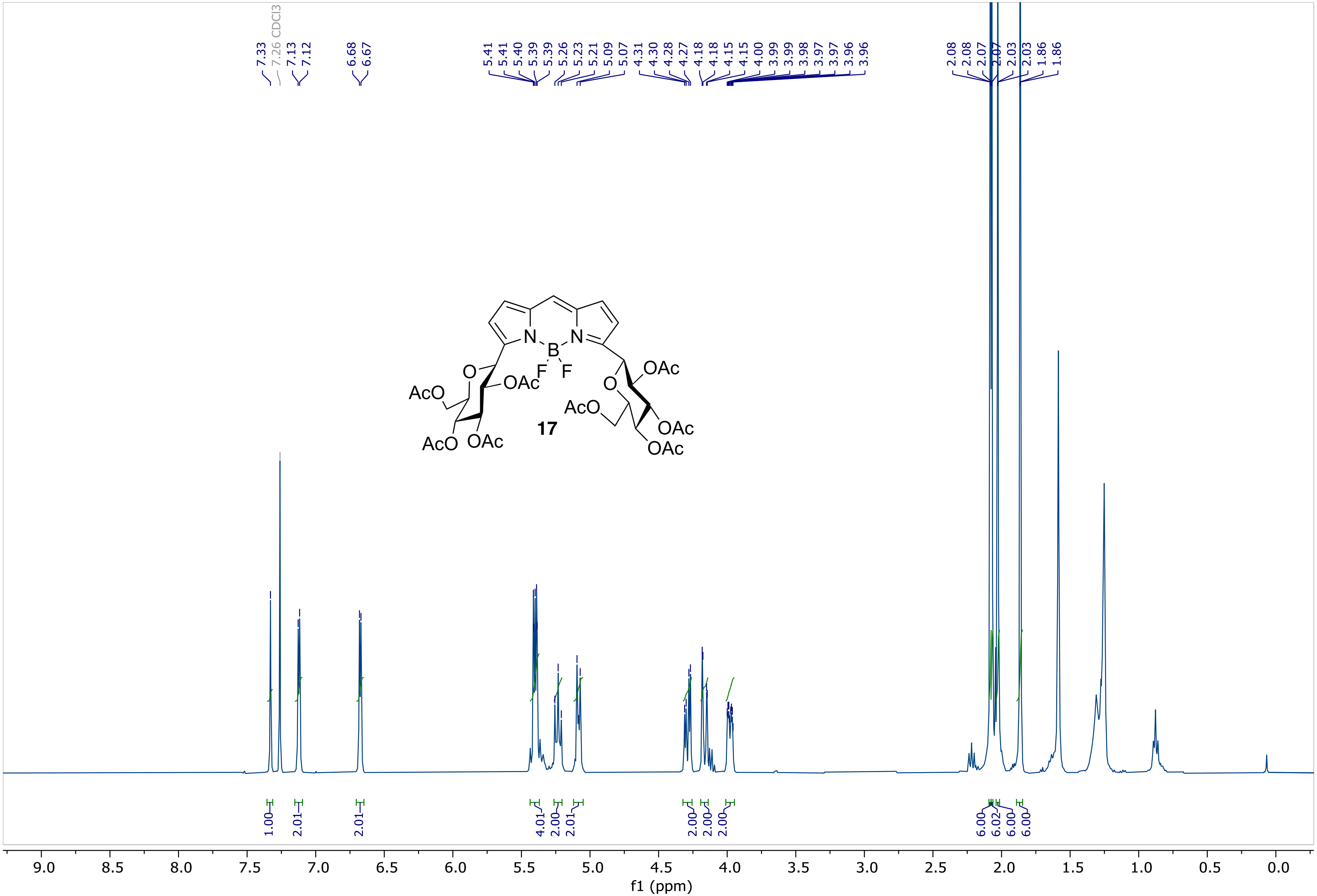

1H NMR (CDCl3, 400 MHz)

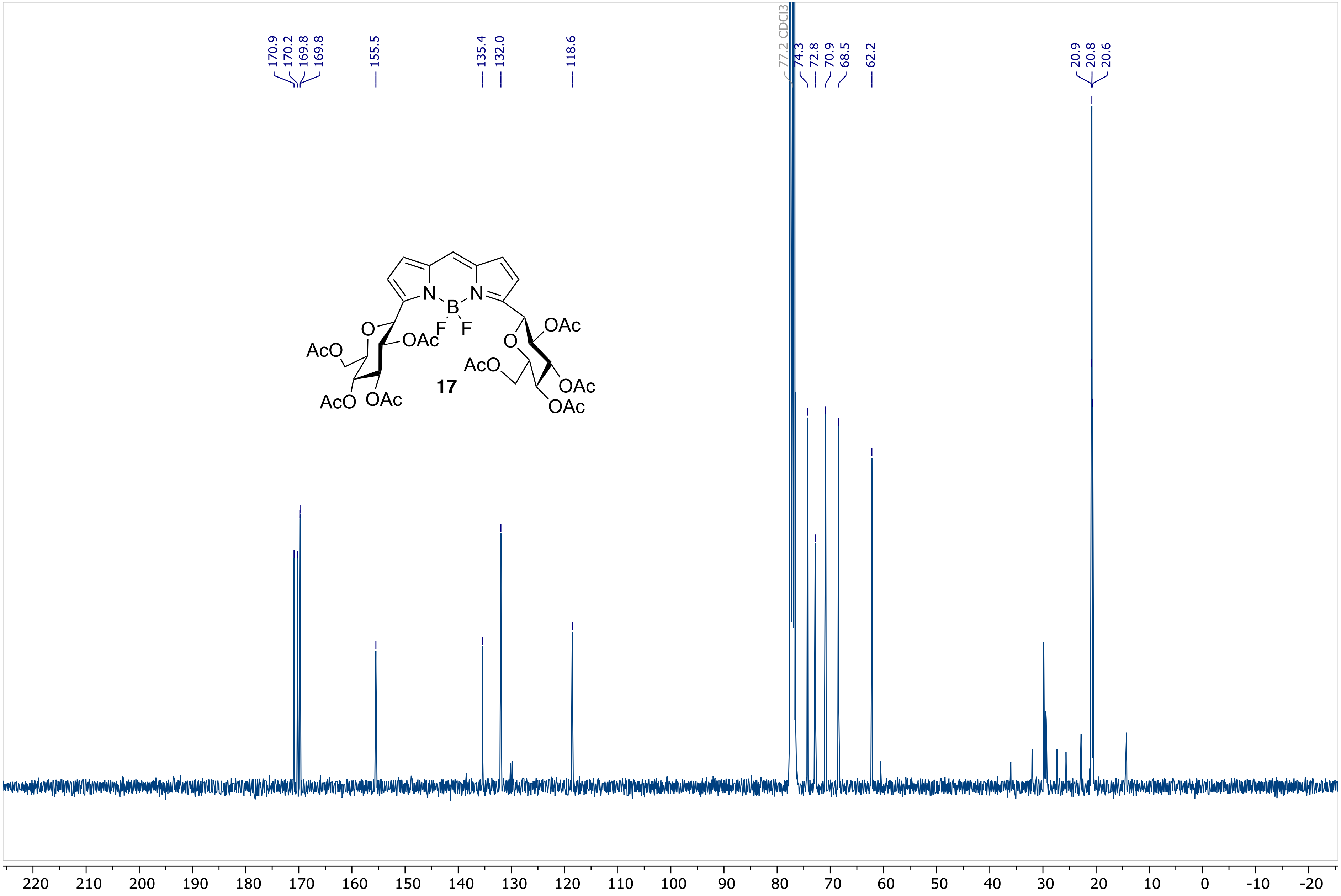

13C {1H} NMR (CDCl3, 125 MHz)

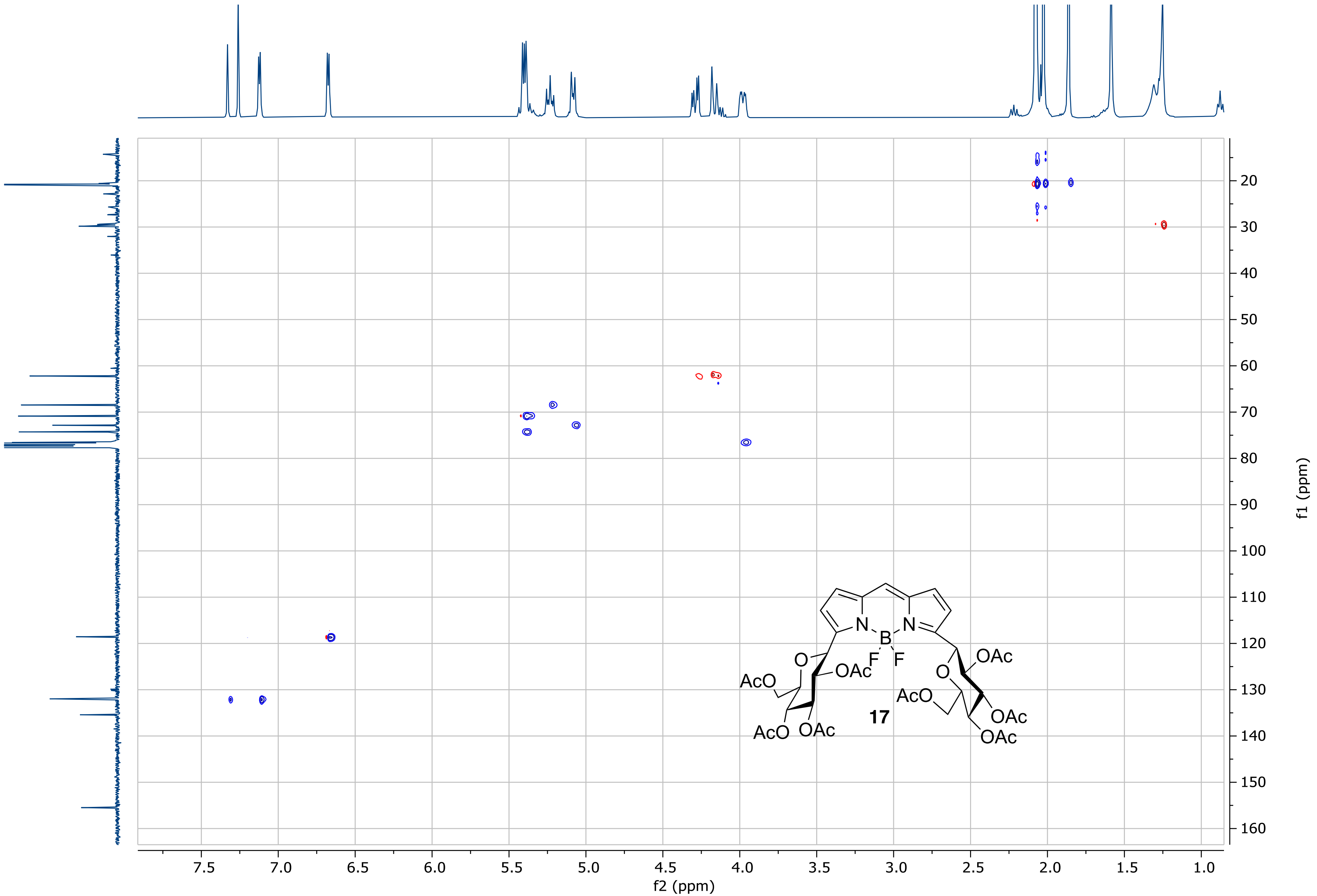

1H-13C gHSQC CDCl3

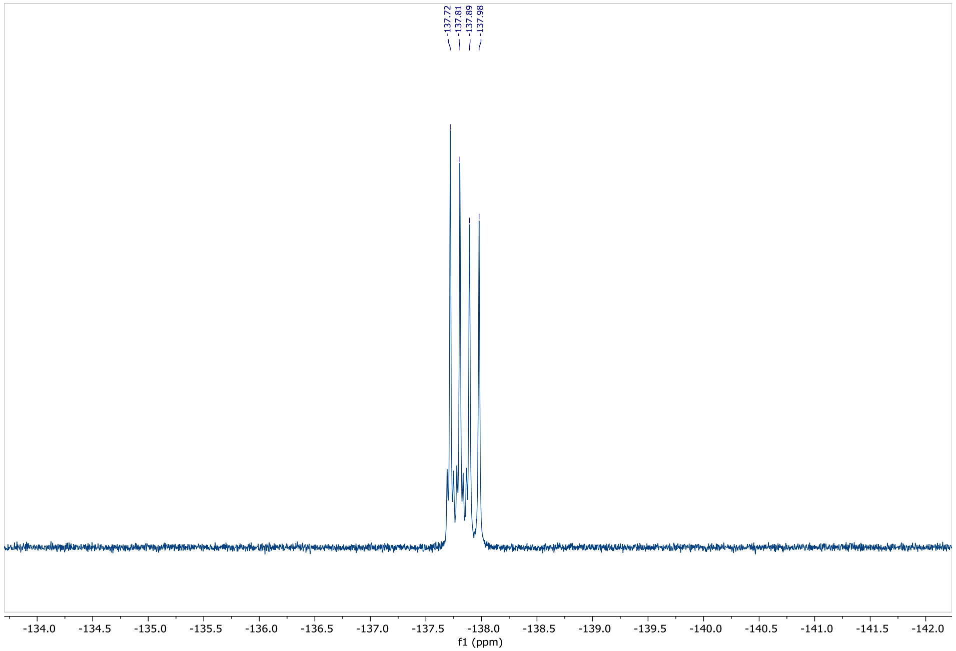

19F NMR (CDCl3, 376 MHz)

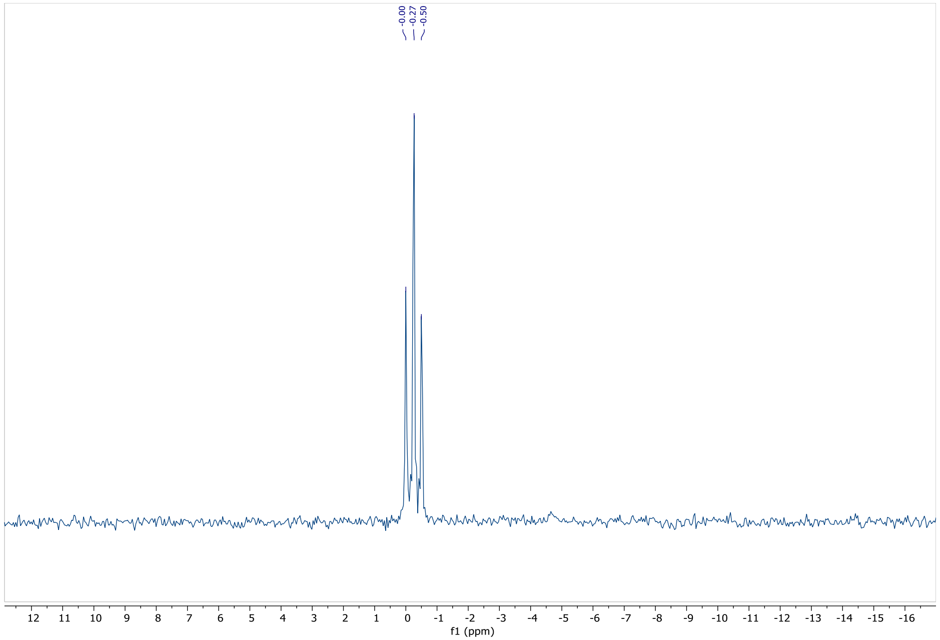

11B NMR (CDCl3, 128 MHz)

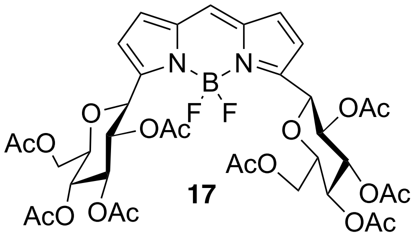

17

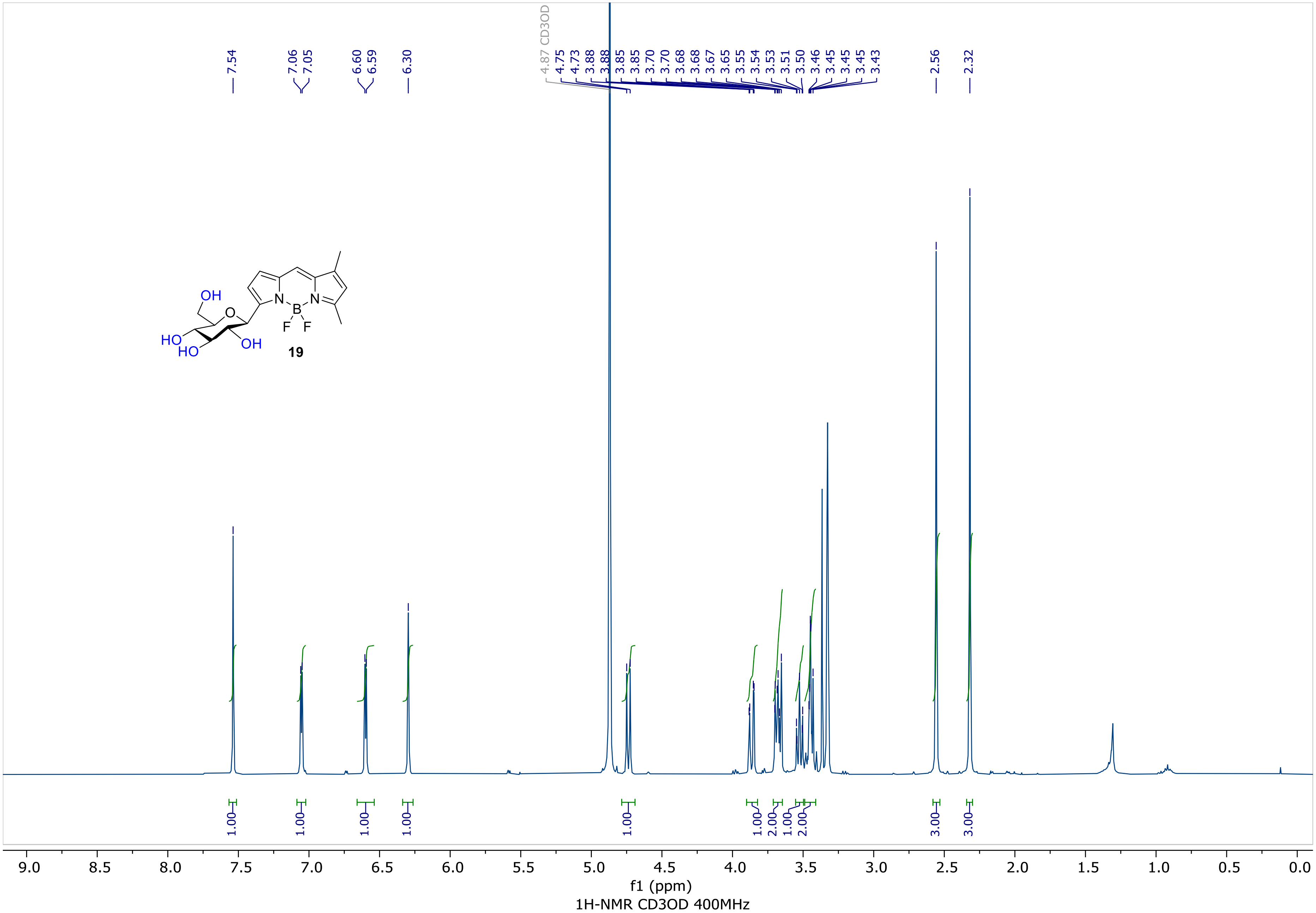

4.87 CD3OD
7.54
7.06
7.05
6.60
6.59
6.30
4.75
4.73
3.88
3.88
3.85
3.85
3.70
3.70
3.68
3.68
3.67
3.65
3.55
3.54
3.53
3.51
3.50
3.46
3.45
3.45
3.45
3.43
2.56
2.32
19
1.00
1.00
1.00
1.00
1.00
1.00
2.00
1.00
2.00
3.00
3.00
f1 (ppm)
1H-NMR CD3OD 400MHz

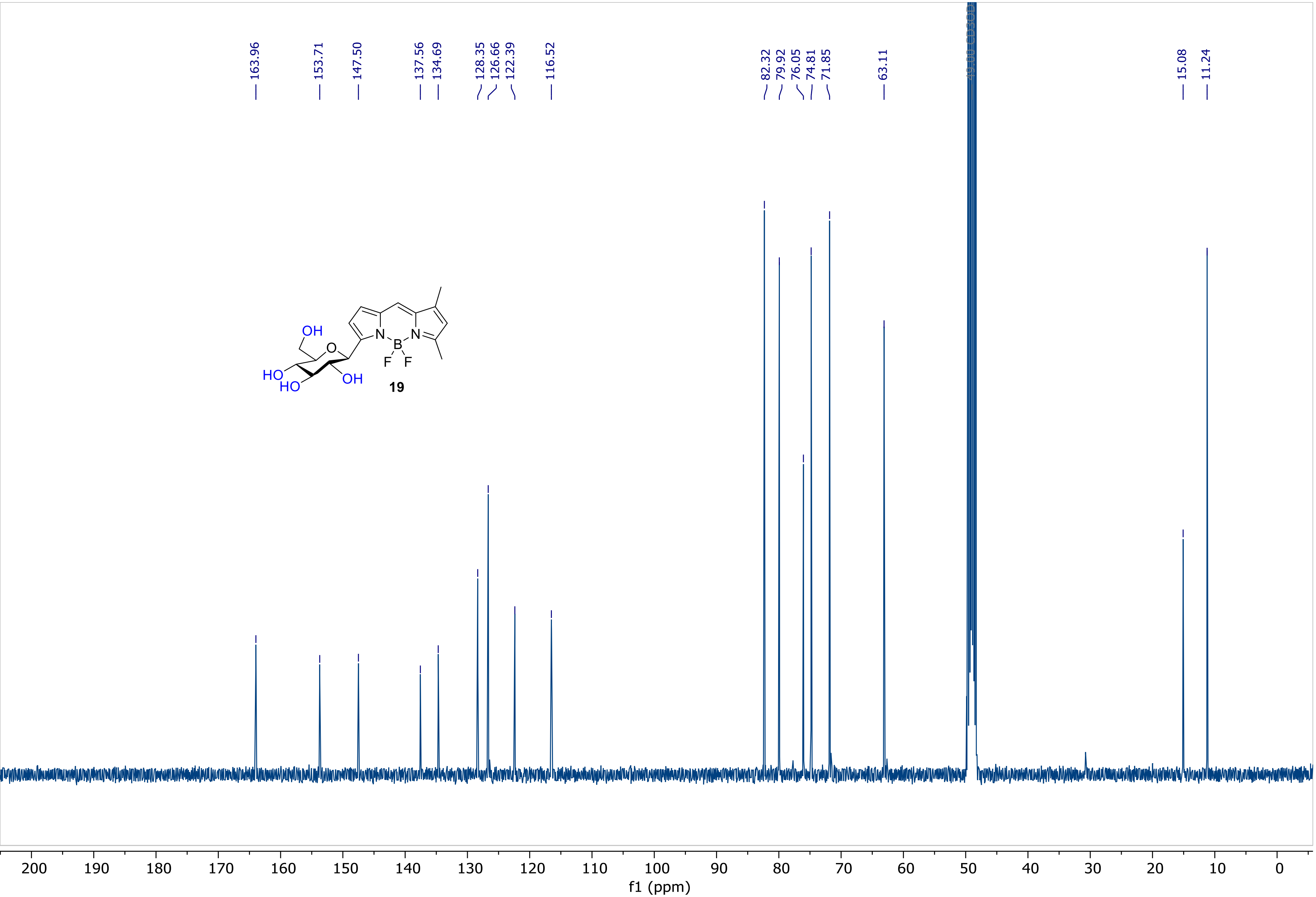

19

13C{1H}-NMR CD3OD 125 MHz

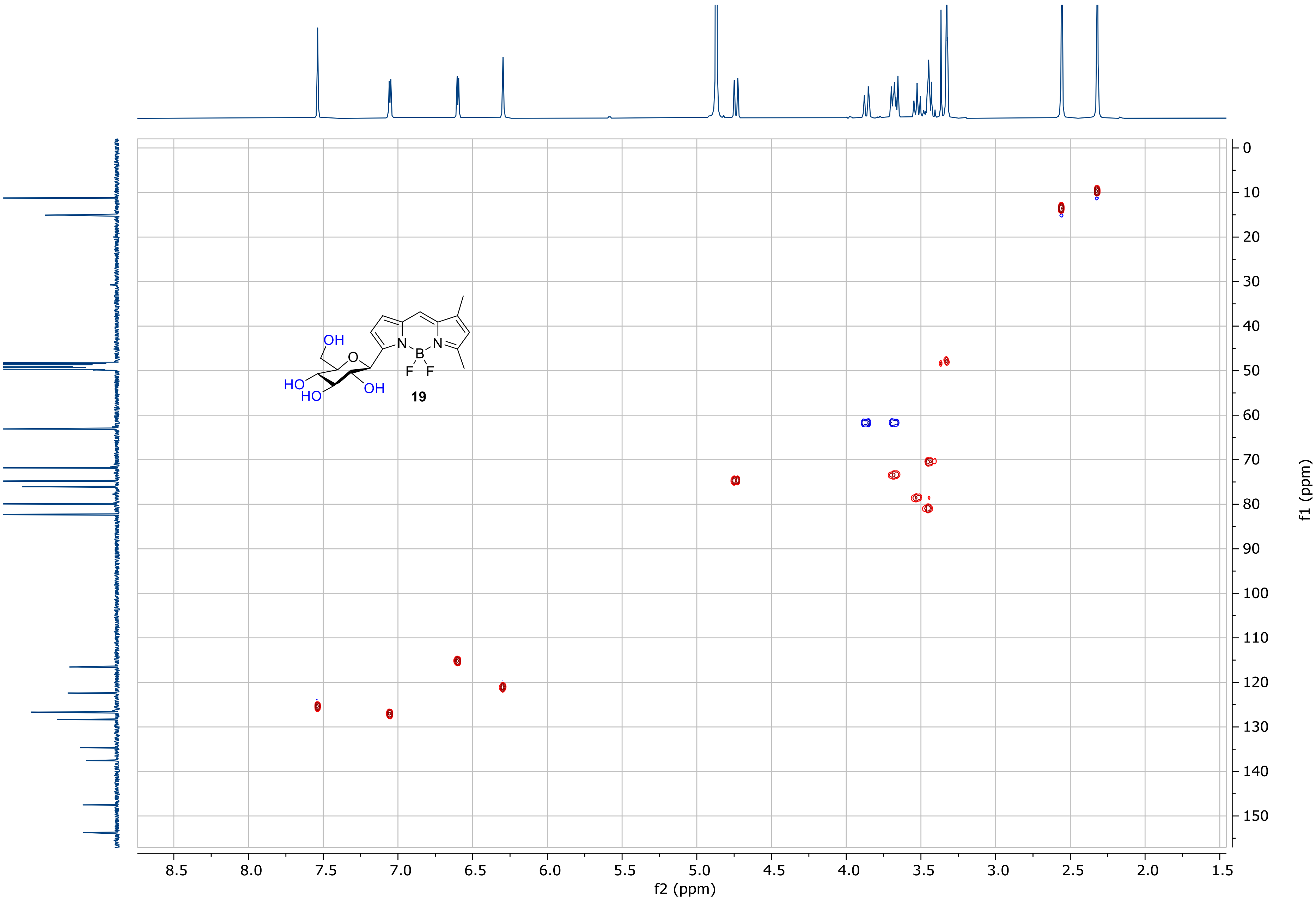

1H-13C gHSQC CD3OD

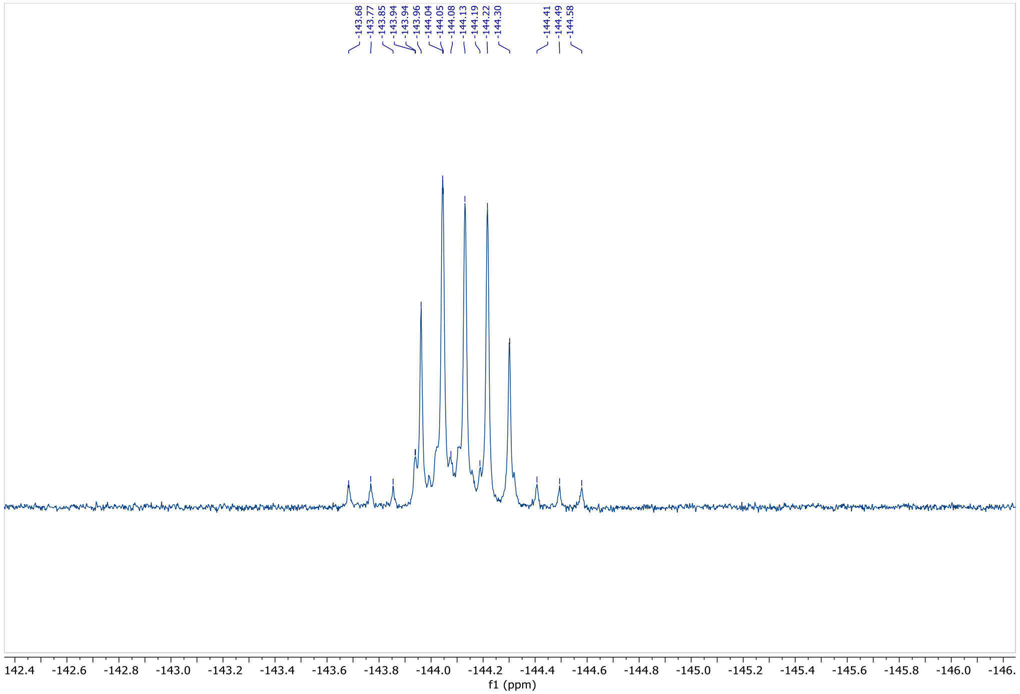

19F NMR CD3OD 376 MHz

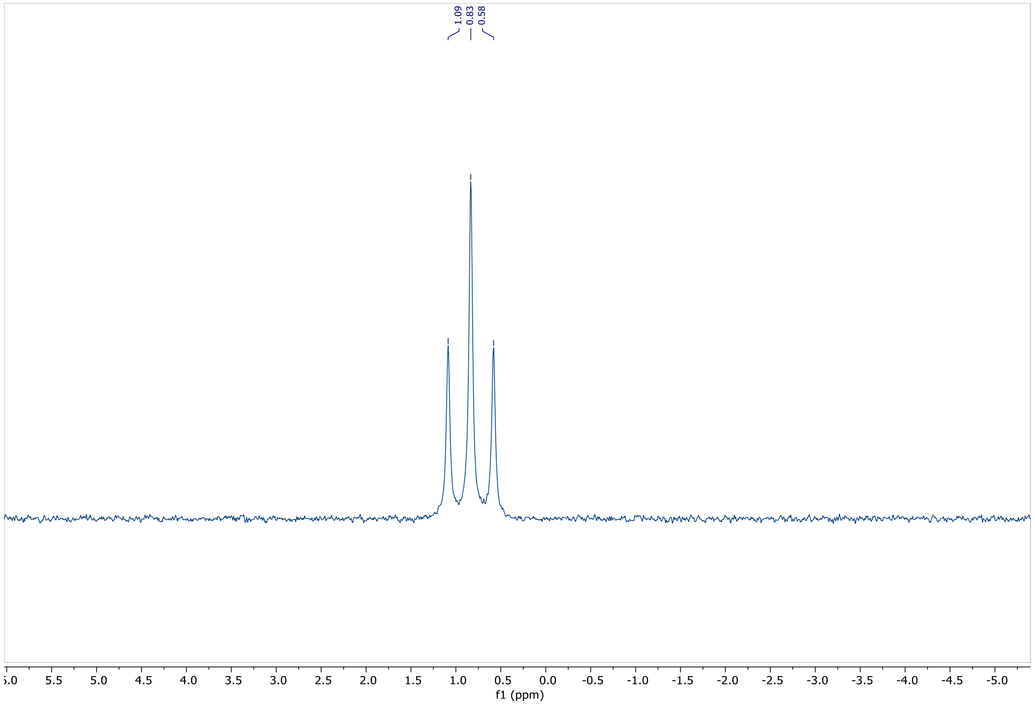

11B NMR CD3OD 128 MHz

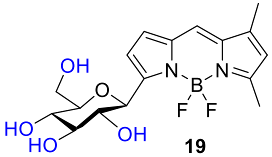

19

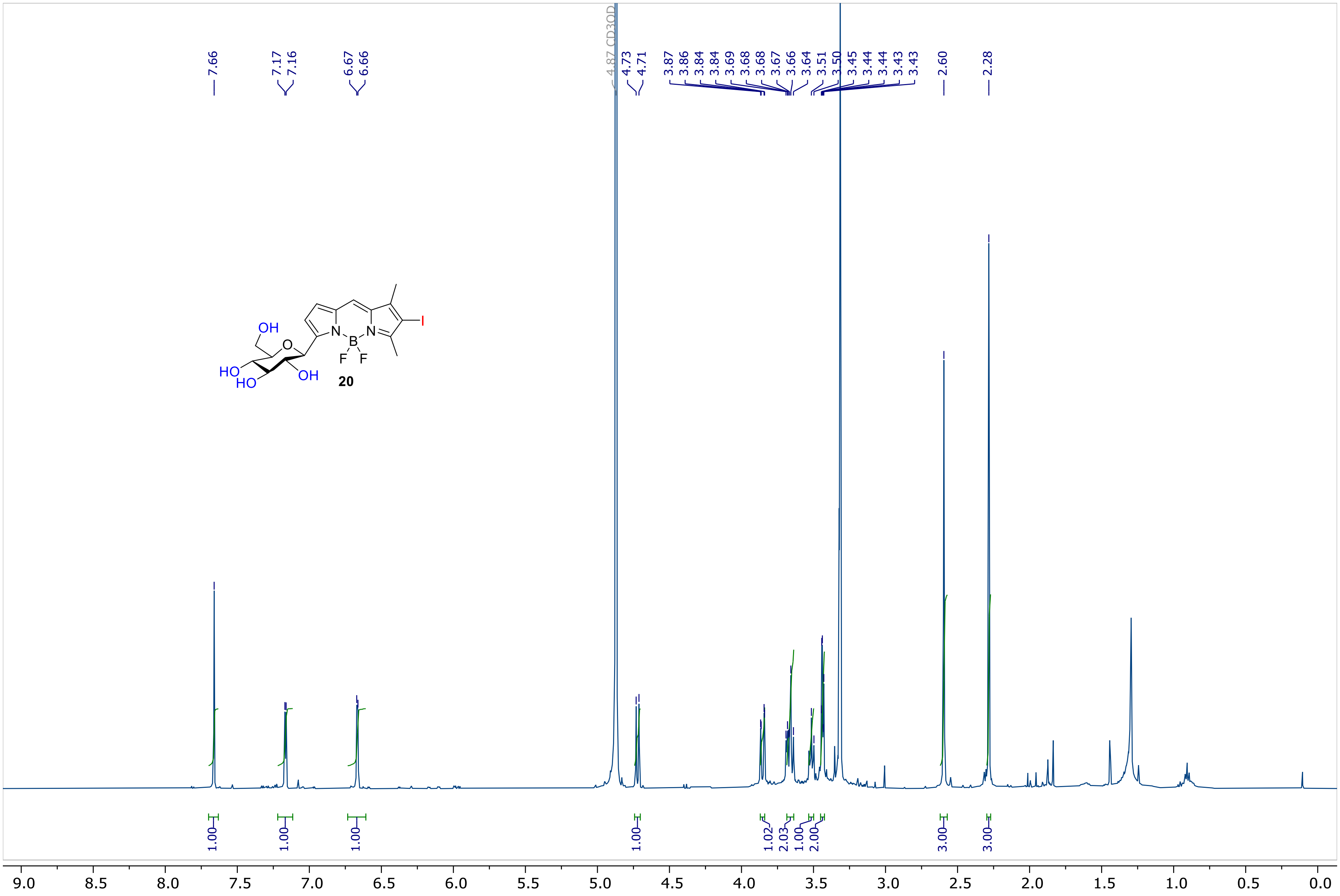

1H-NMR CD3OD 400MHz

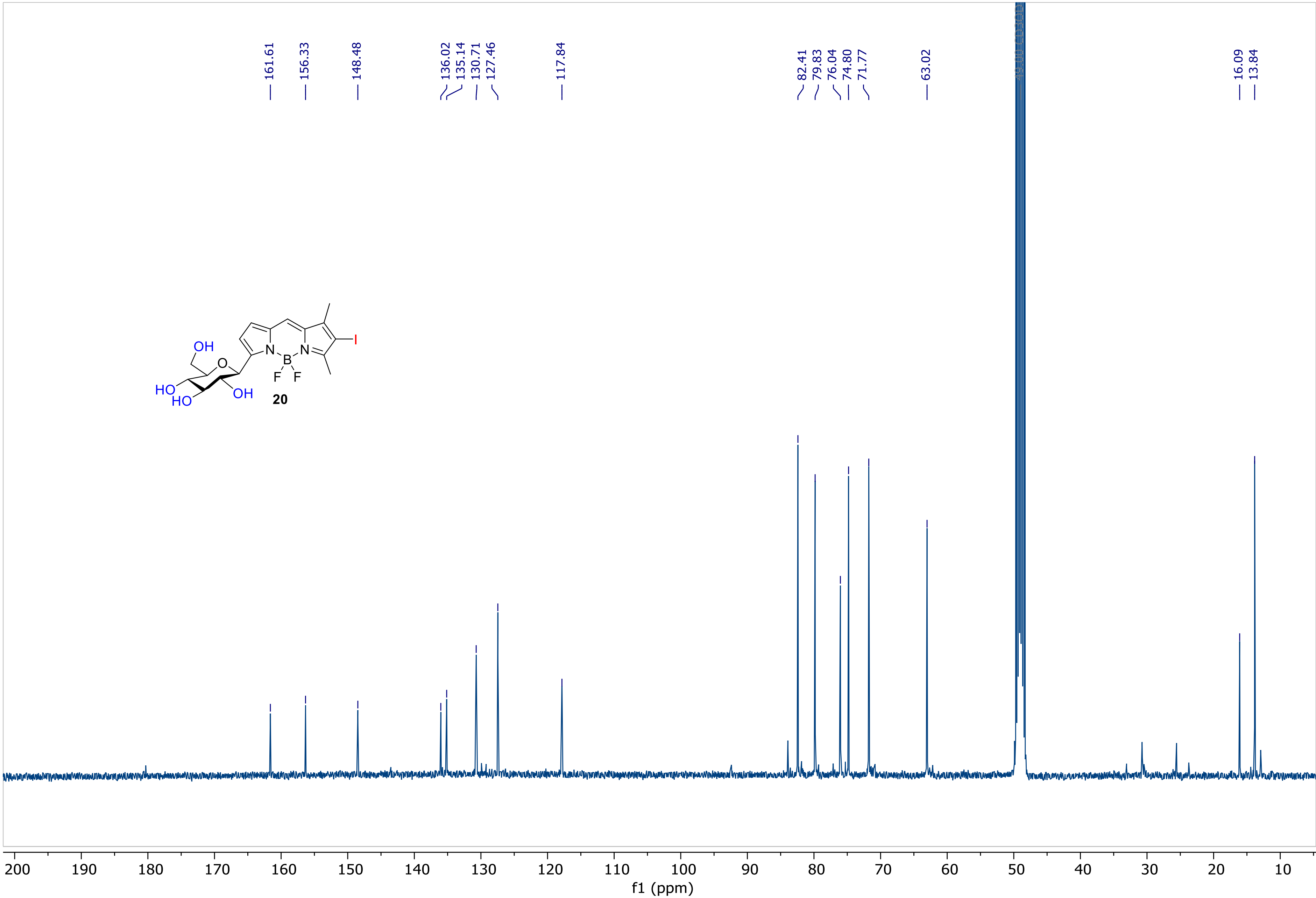

13C{1H}-NMR CD3OD 400 MHz

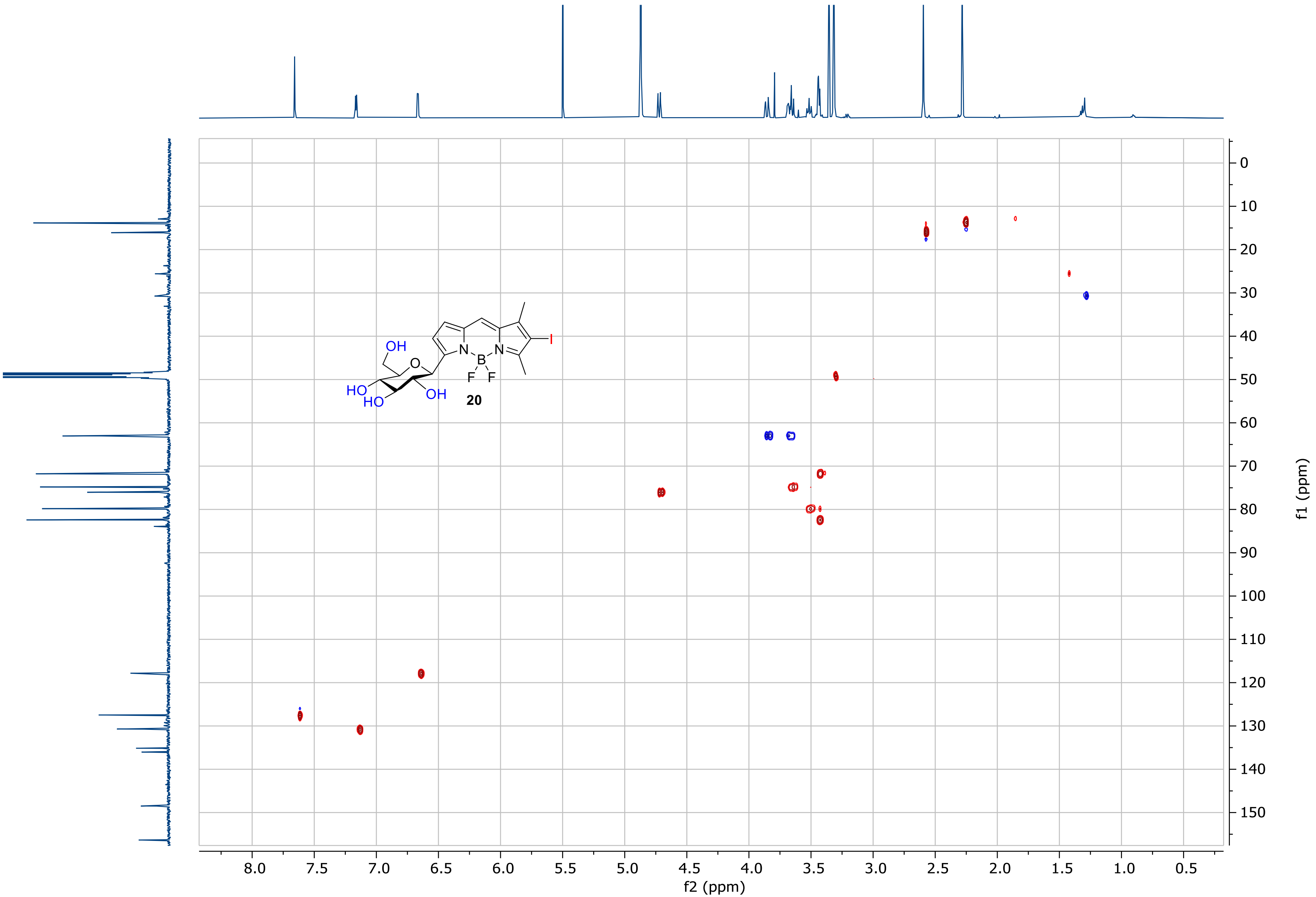

13C-1H gHSQC CD3OD

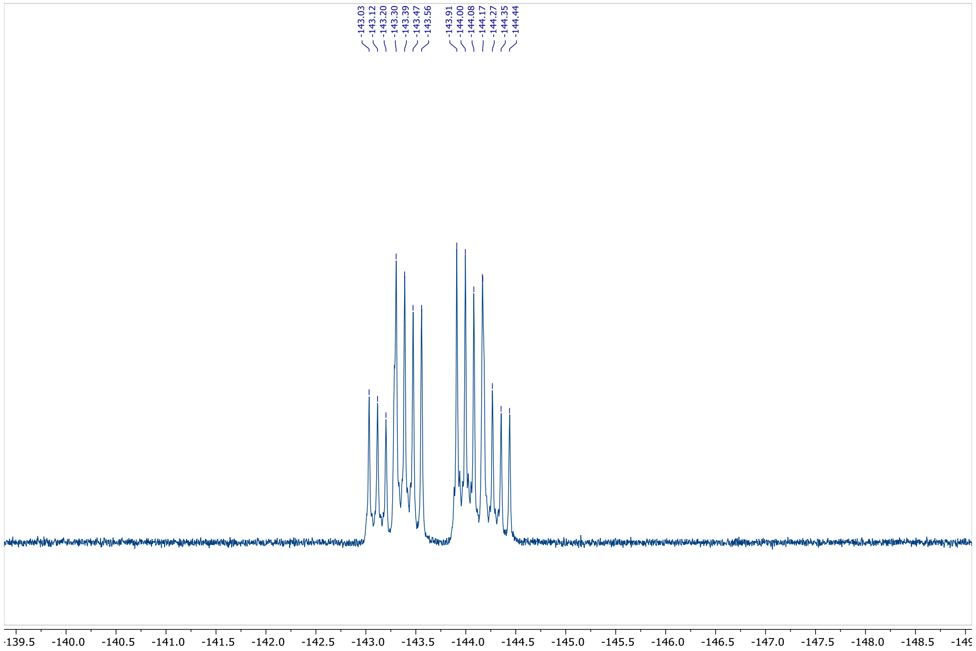

19F NMR (CD3OD, 376 MHz)

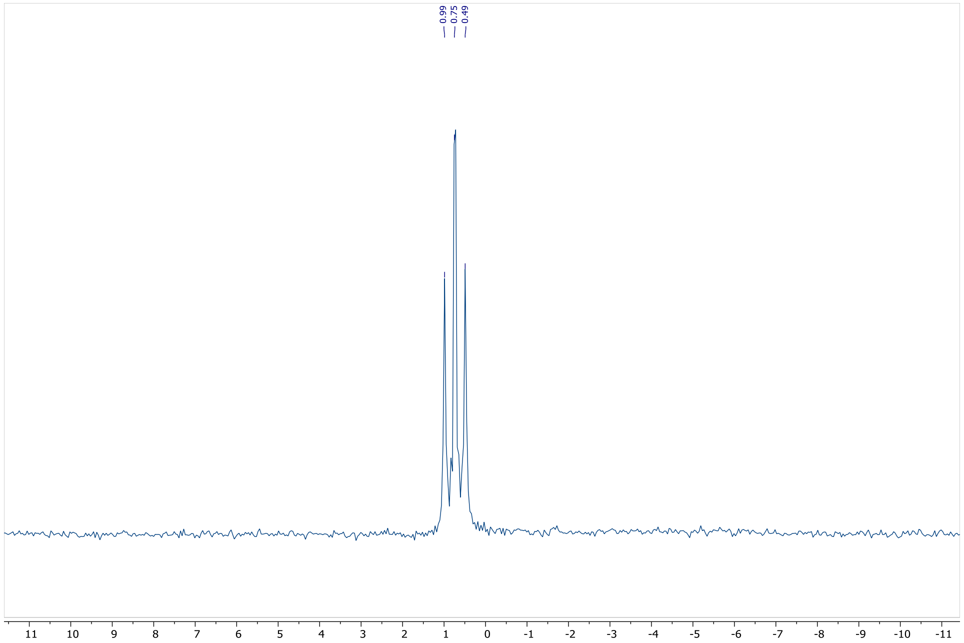

Florian/FR-3OH_11B-1-1.jdf

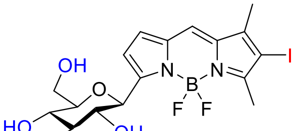

20

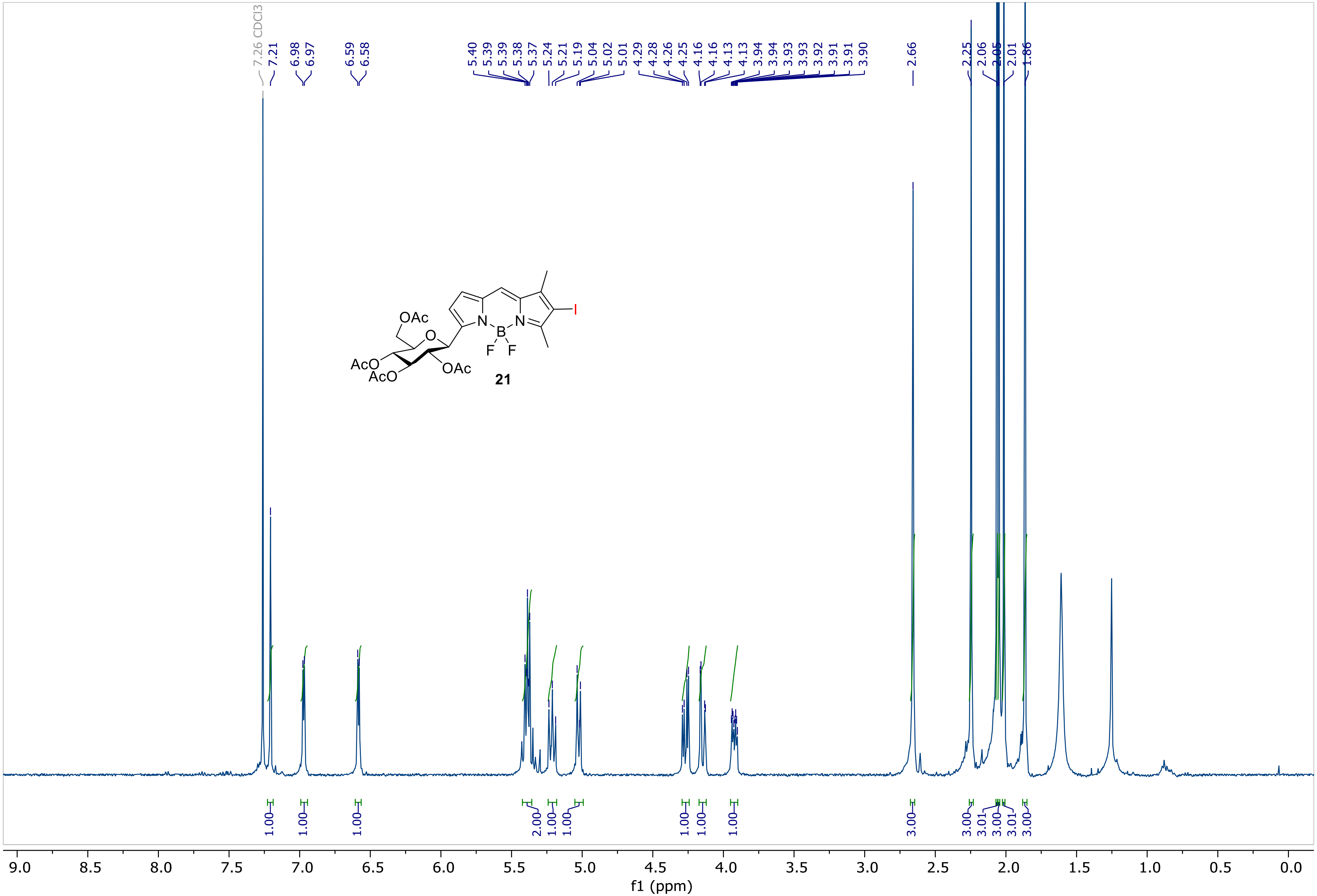

1H-NMR CDCl3 400 MHz

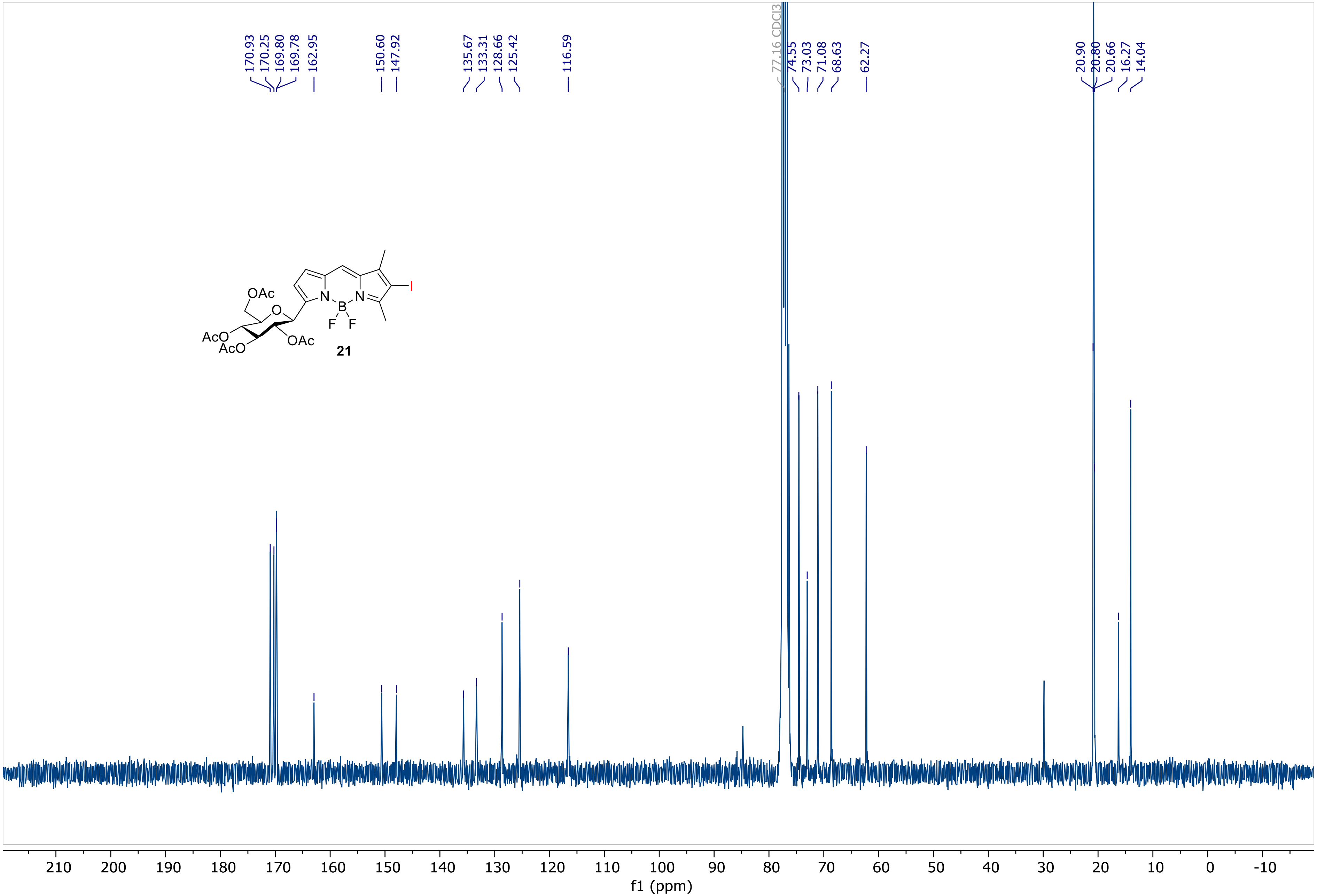

13C{1H}-NMR CDCl3 125 MHz

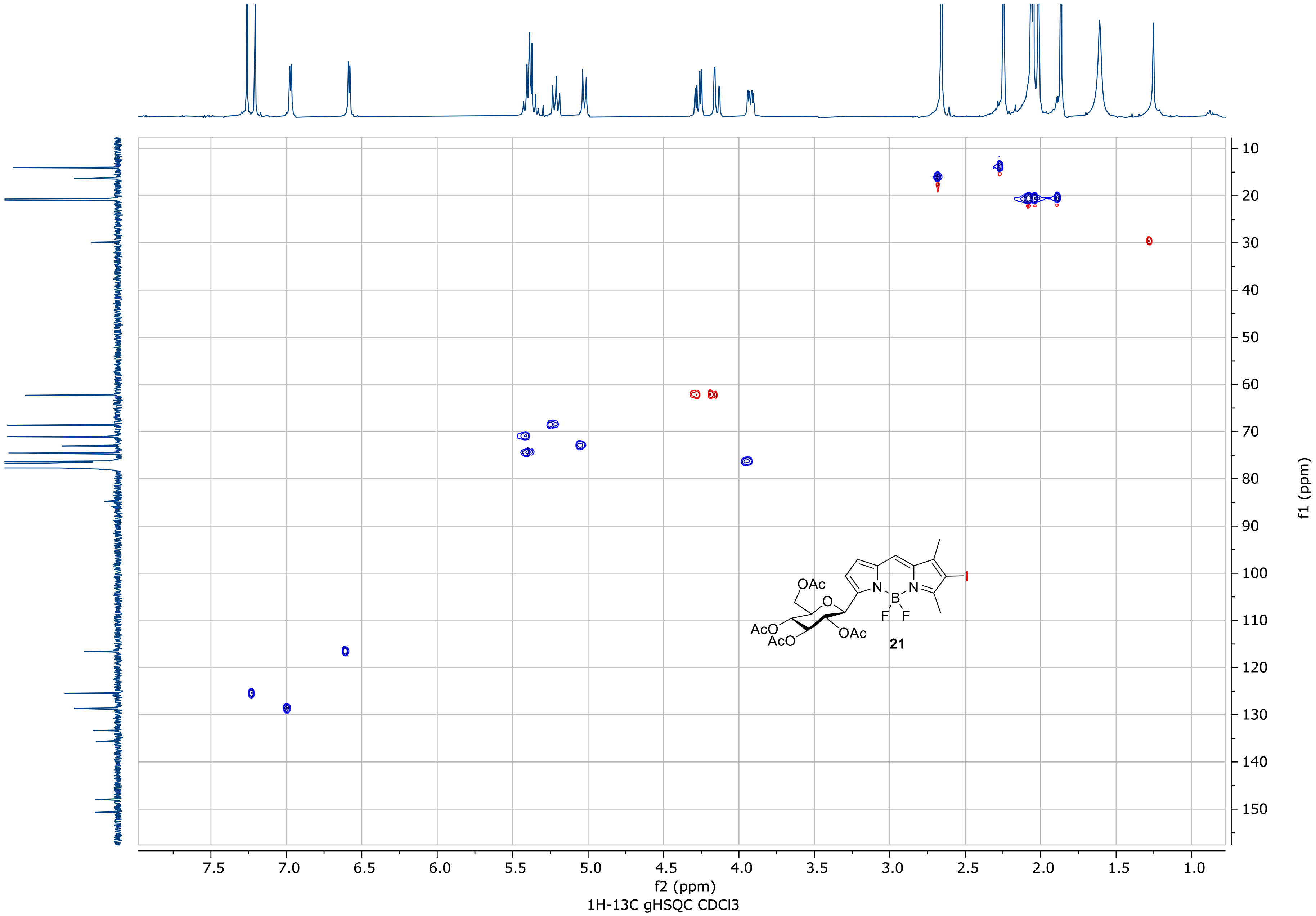

OAc
O
AcO
AcO
OAc
N
B
N
F F
I
21
f2 (ppm)
f1 (ppm)
1H-13C gHSQC CDCl3

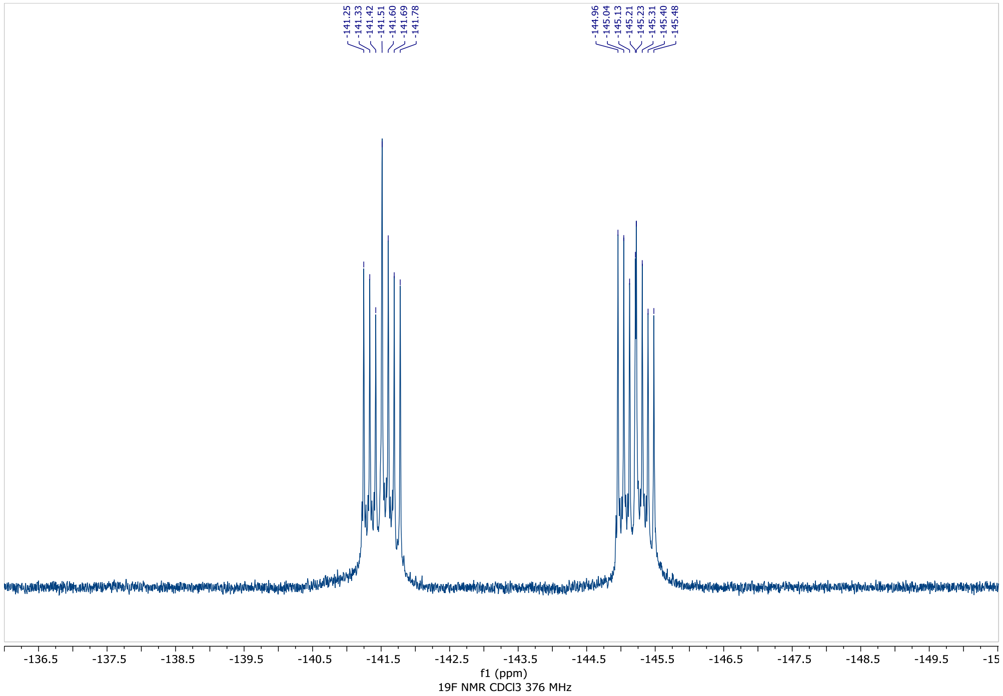
-141.25
-141.33
-141.42
-141.51
-141.60
-141.69
-141.78
-144.96
-145.04
-145.13
-145.21
-145.23
-145.31
-145.40
-145.48
-136.5 -137.5 -138.5 -139.5 -140.5 -141.5 -142.5 -143.5 -144.5 -145.5 -146.5 -147.5 -148.5 -149.5
f1 (ppm)
19F NMR CDCl3 376 MHz

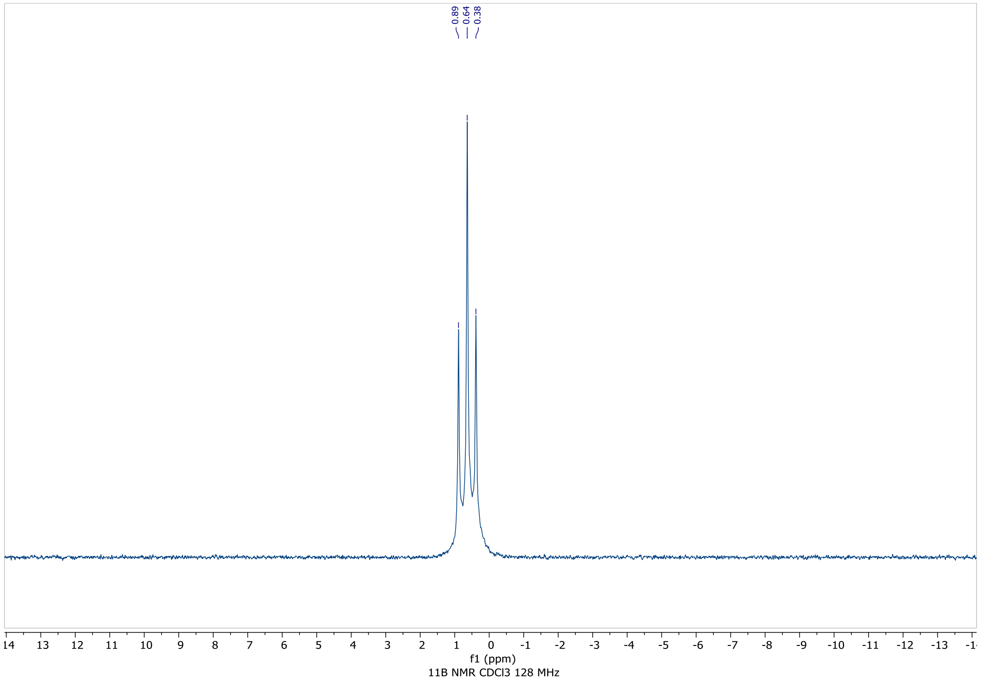
0.89
0.64
0.38
14 13 12 11 10 9 8 7 6 5 4 3 2 1 0 -1 -2 -3 -4 -5 -6 -7 -8 -9 -10 -11 -12 -13
f1 (ppm)
11B NMR CDCl3 128 MHz

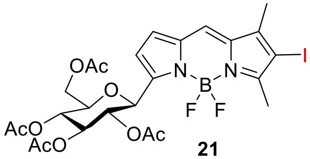
OAc
O
AcO
AcO
OAc
N
B
N
F
F
I

21

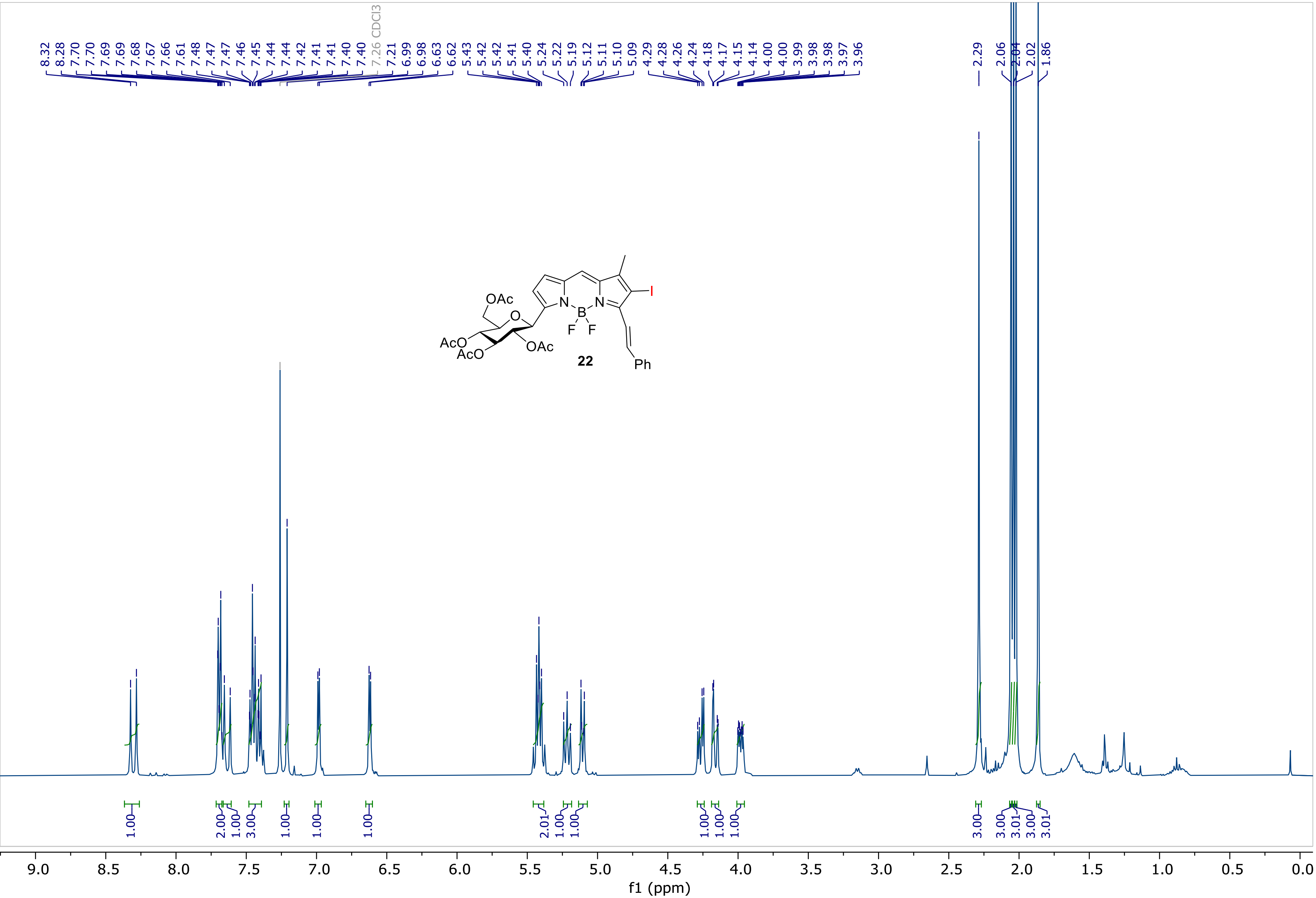

1H-NMR CDCl3 400 MHz

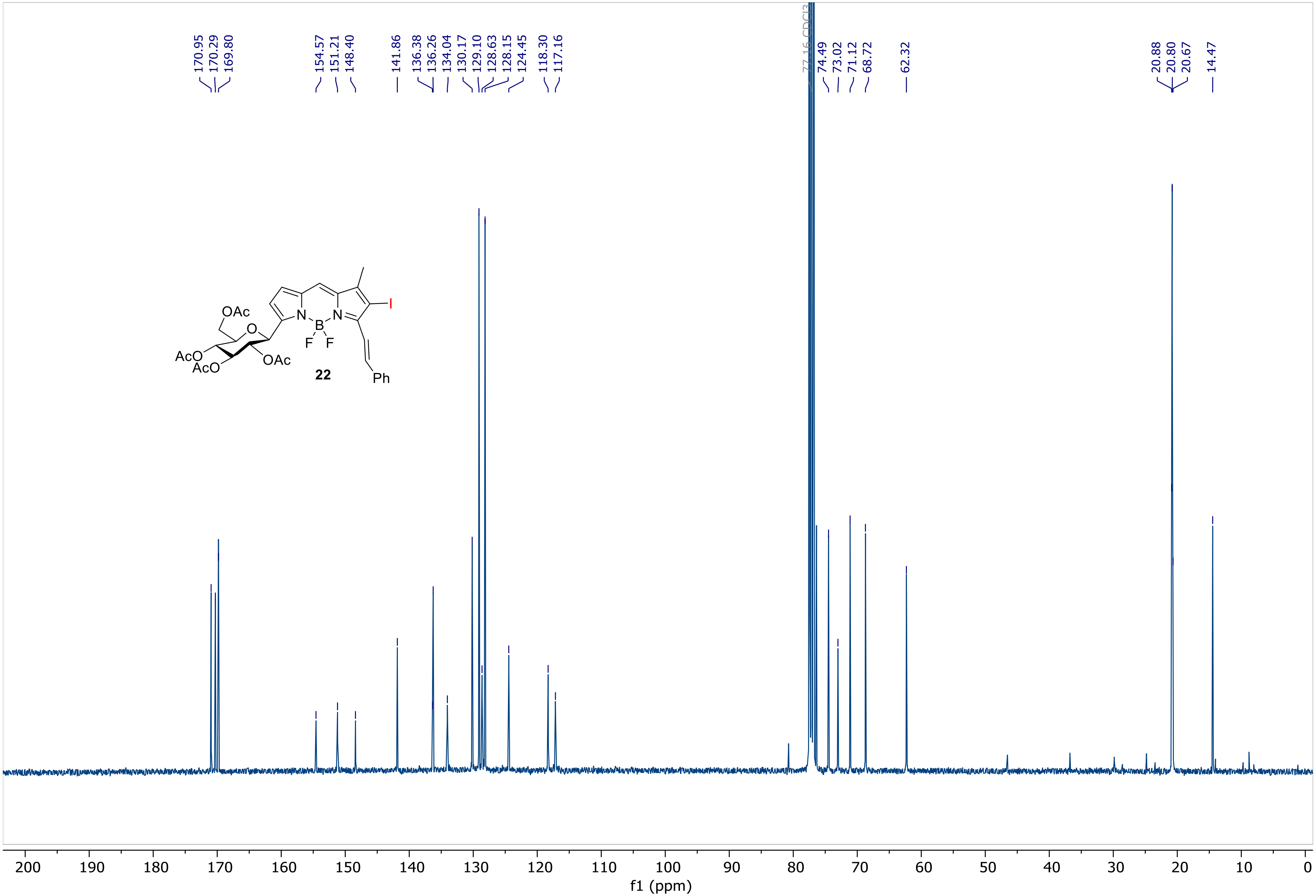

13C{1H} NMR 125MHz

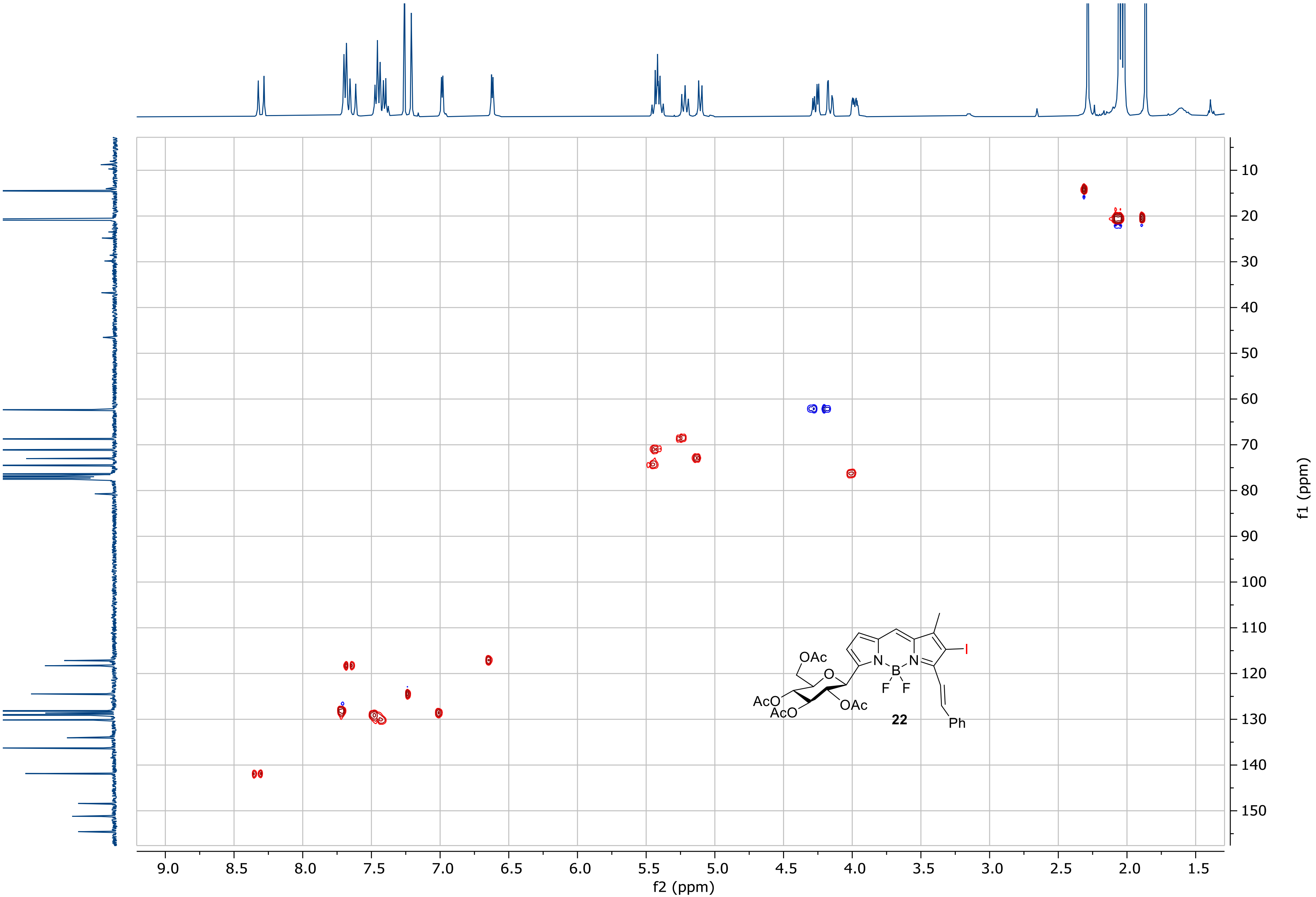

1H-13C gHSQC CDCl3

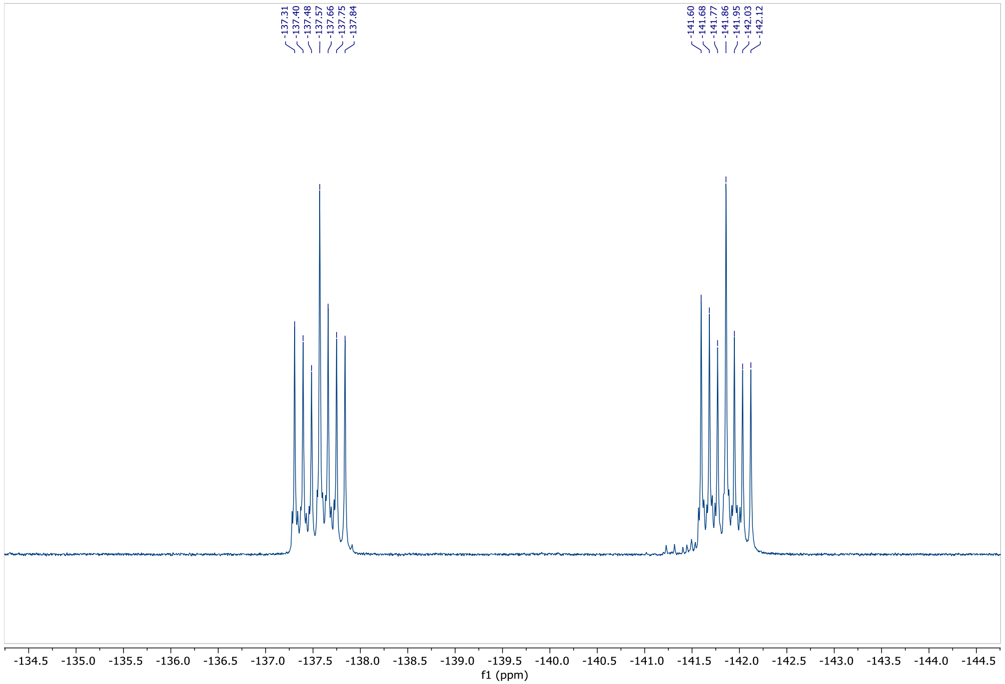

19F-NMR (CDCl3, 376 MHz)

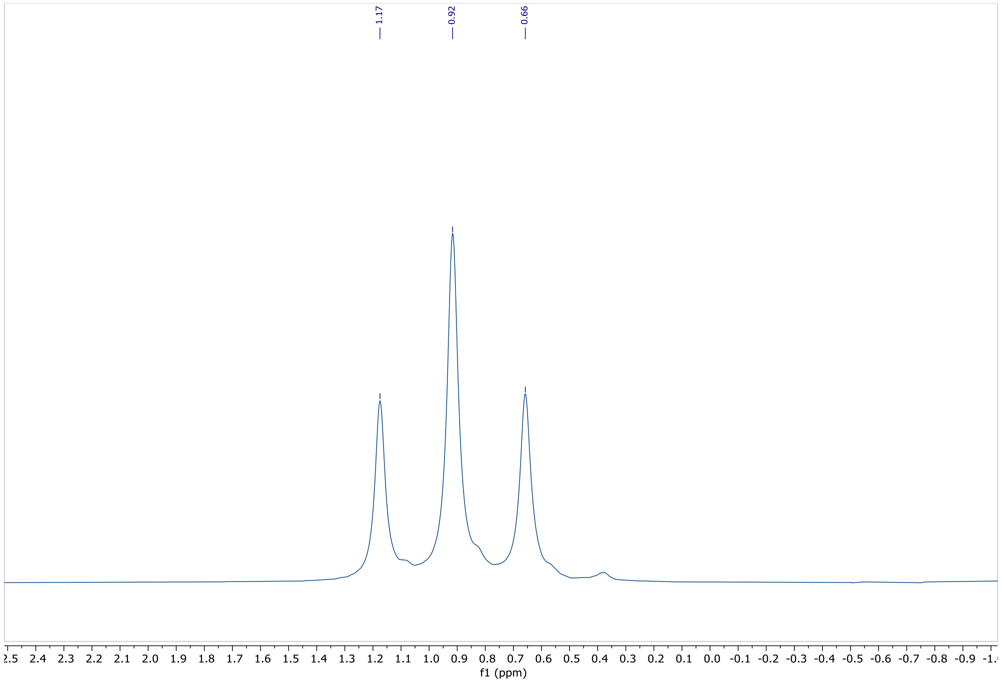

11B NMR (CDCl3, 128 MHz)

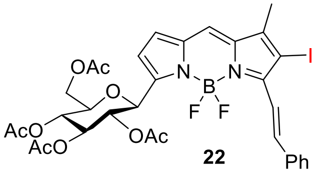

22

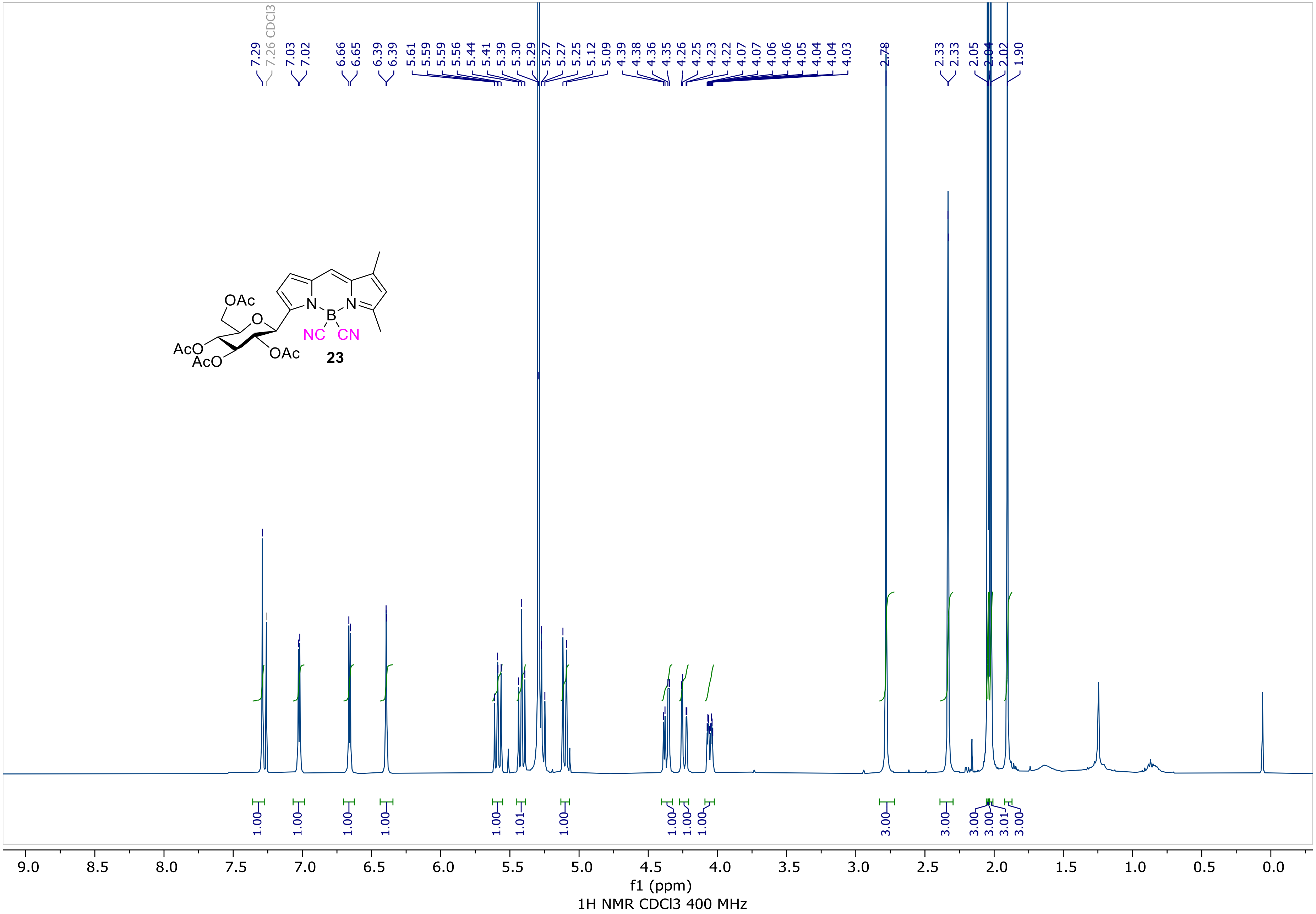
7.29
7.26 CDCl3
7.03
7.02
6.66
6.65
6.39
6.39
5.61
5.59
5.59
5.56
5.44
5.41
5.39
5.30
5.29
5.27
5.27
5.25
5.12
5.09
4.39
4.38
4.36
4.35
4.26
4.25
4.23
4.22
4.07
4.07
4.06
4.06
4.05
4.04
4.04
4.03
2.78
2.33
2.33
2.05
2.04
2.02
1.90
OAc
O
N
B
N
NC
CN
AcO
AcO
OAc
23
1.00
1.00
1.00
1.00
1.00
1.01
1.00
1.00
1.00
1.00
3.00
3.00
3.00
3.00
3.01
3.00
9.0
8.5
8.0
7.5
7.0
6.5
6.0
5.5
5.0
4.5
4.0
3.5
3.0
2.5
2.0
1.5
1.0
0.5
0.0
f1 (ppm)
1H NMR CDCl3 400 MHz

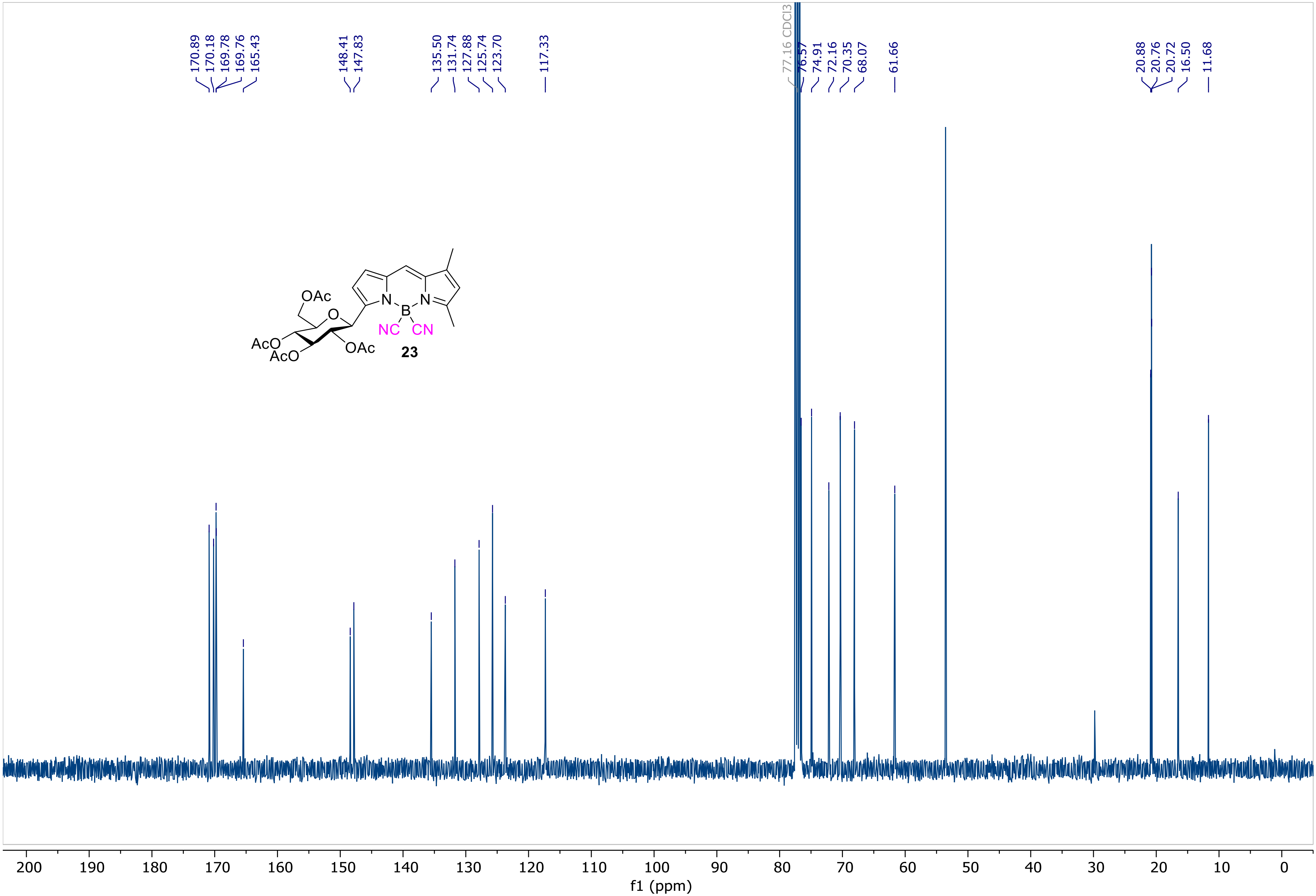

13C{1H} NMR CDCl3 125 MHz

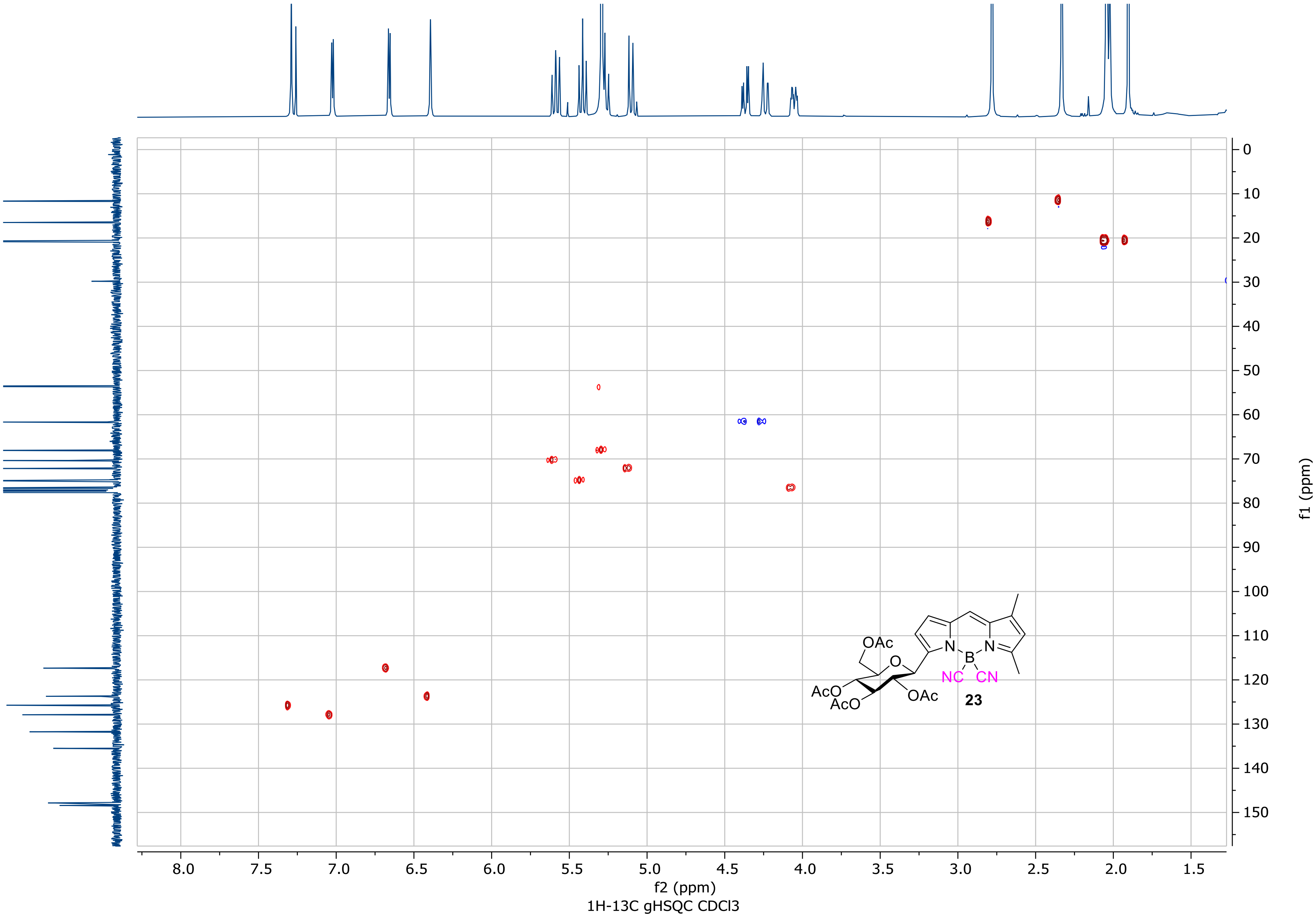
OAc
AcO
AcO
OAc
O
N
B
N
NC
CN
23
f1 (ppm)
f2 (ppm)
1H-13C gHSQC CDCl3

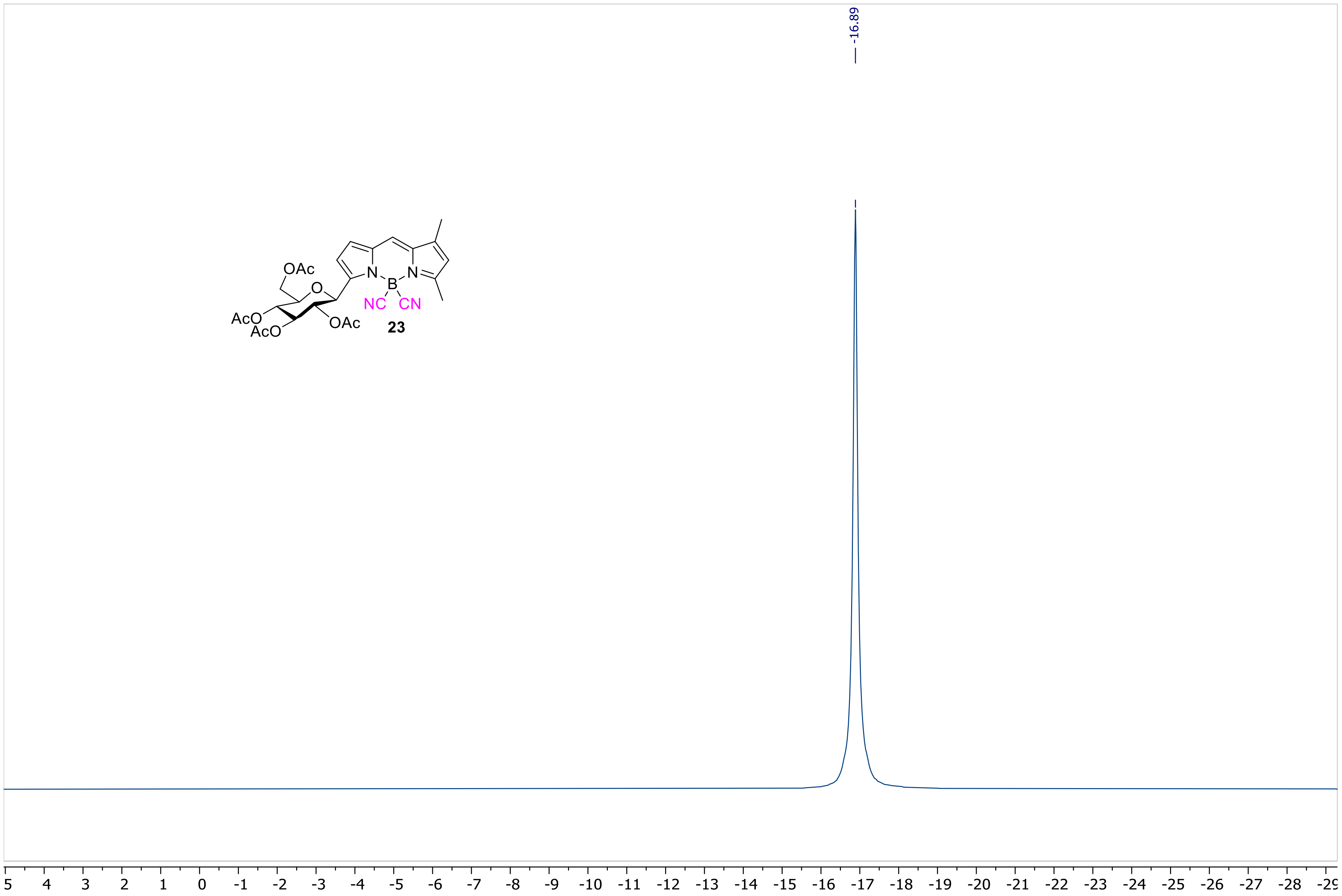

f1 (ppm)

11B NMR CDCl3 128 MHz

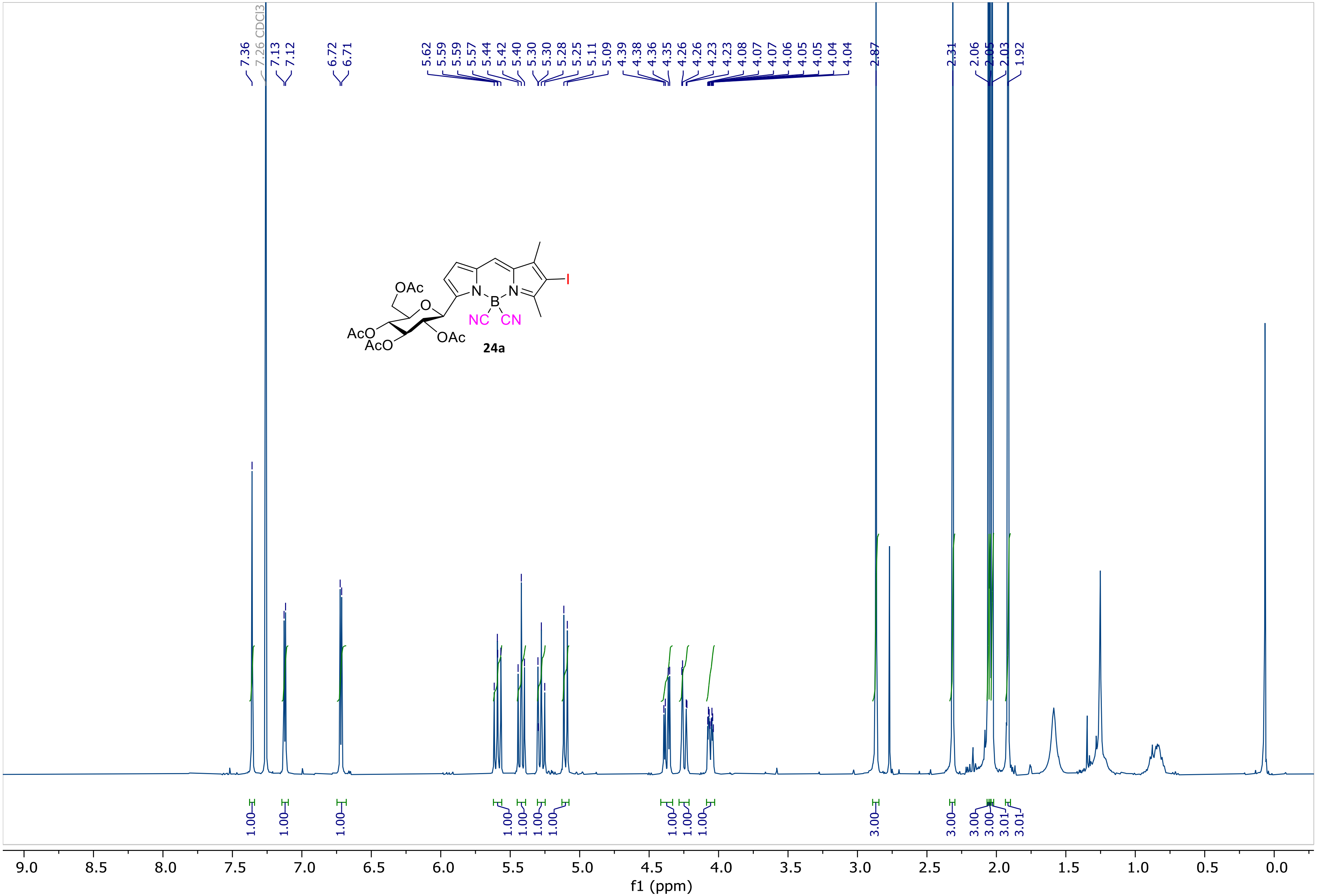

1H NMR CDCl3 400 MHz

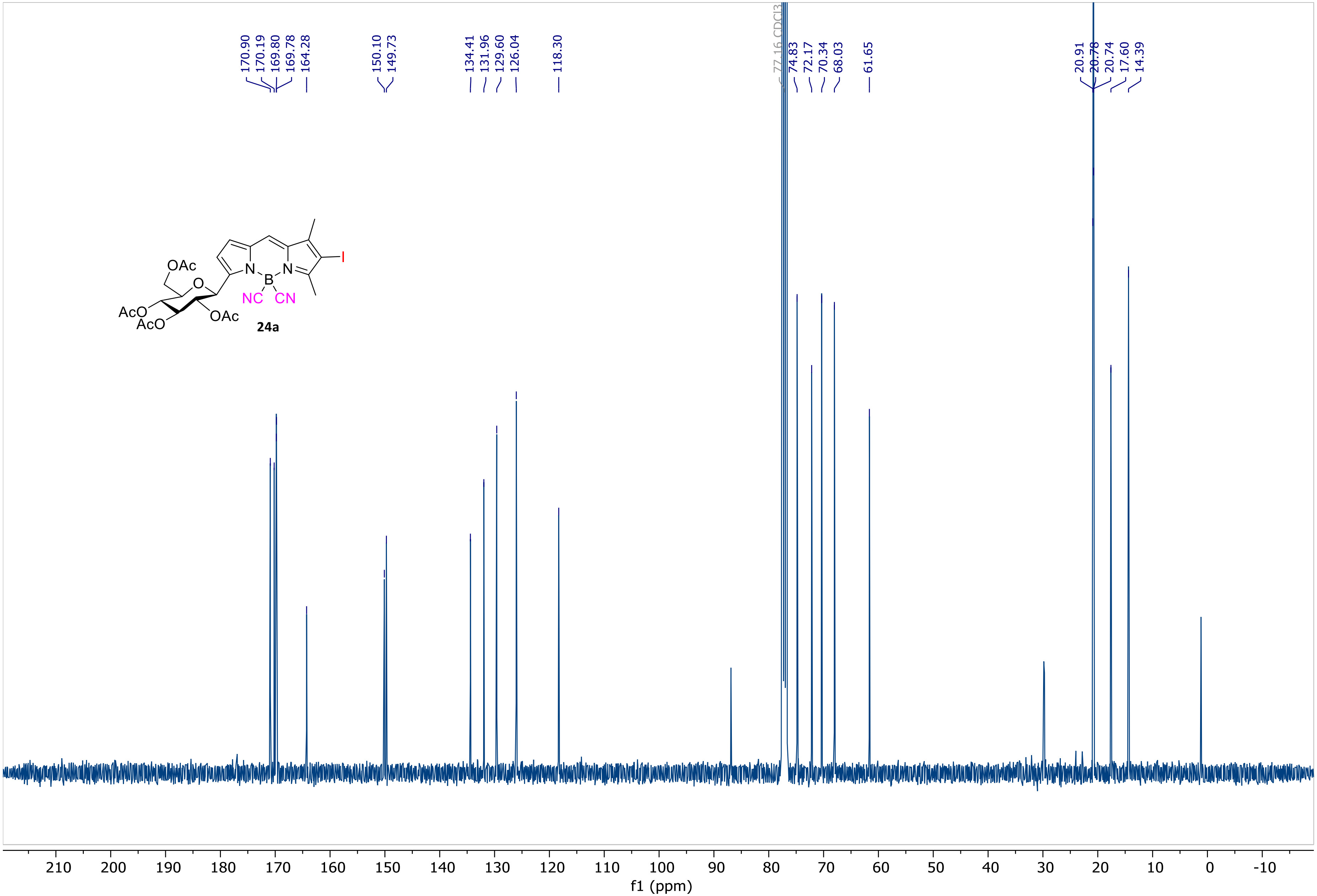

13C {1H} NMR CDCl3 125 MHz

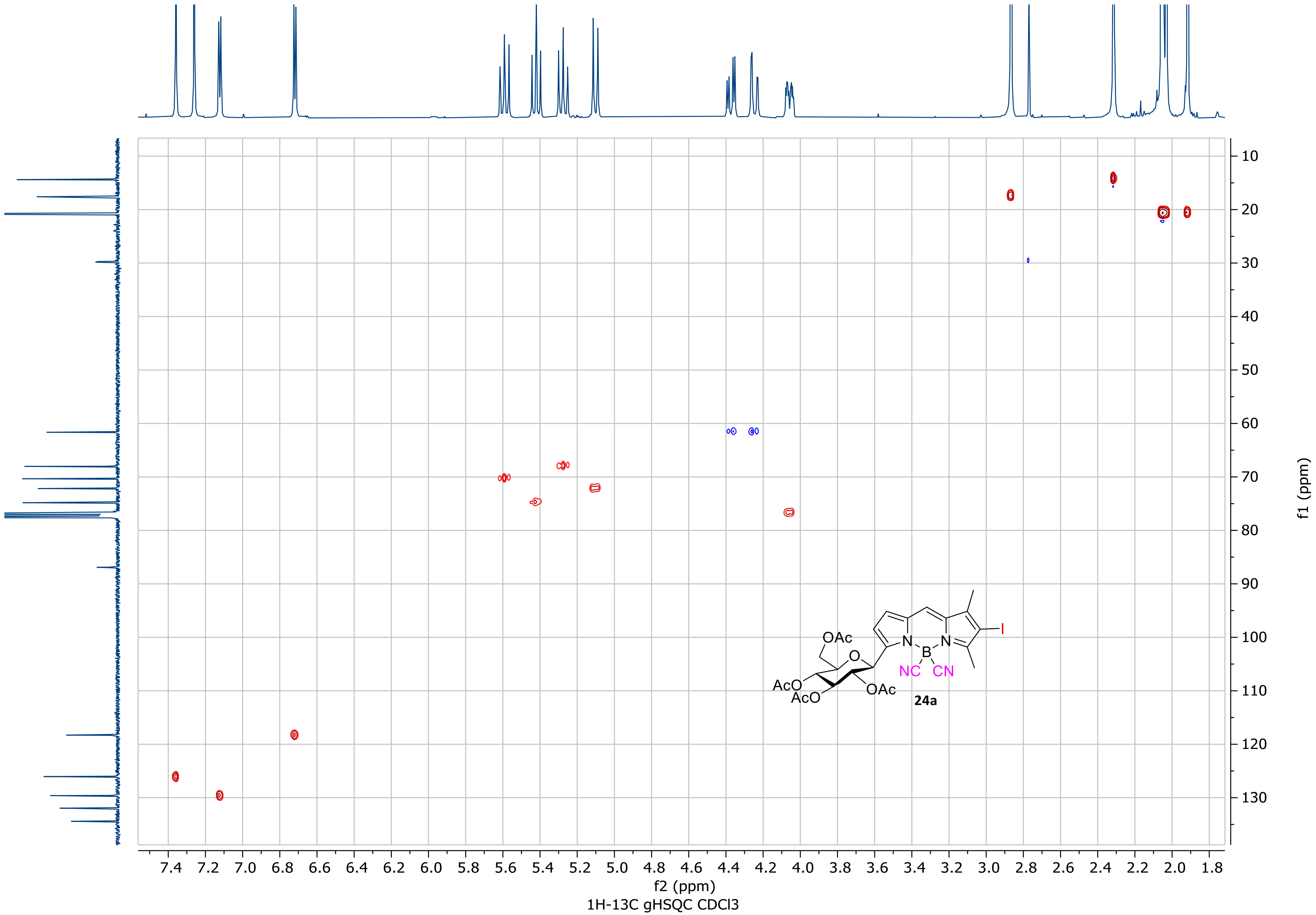
f2 (ppm)
f1 (ppm)
1H-13C gHSQC CDCl3
OAc
O
AcO
AcO
OAc
N
B
N
NC
CN
I
24a

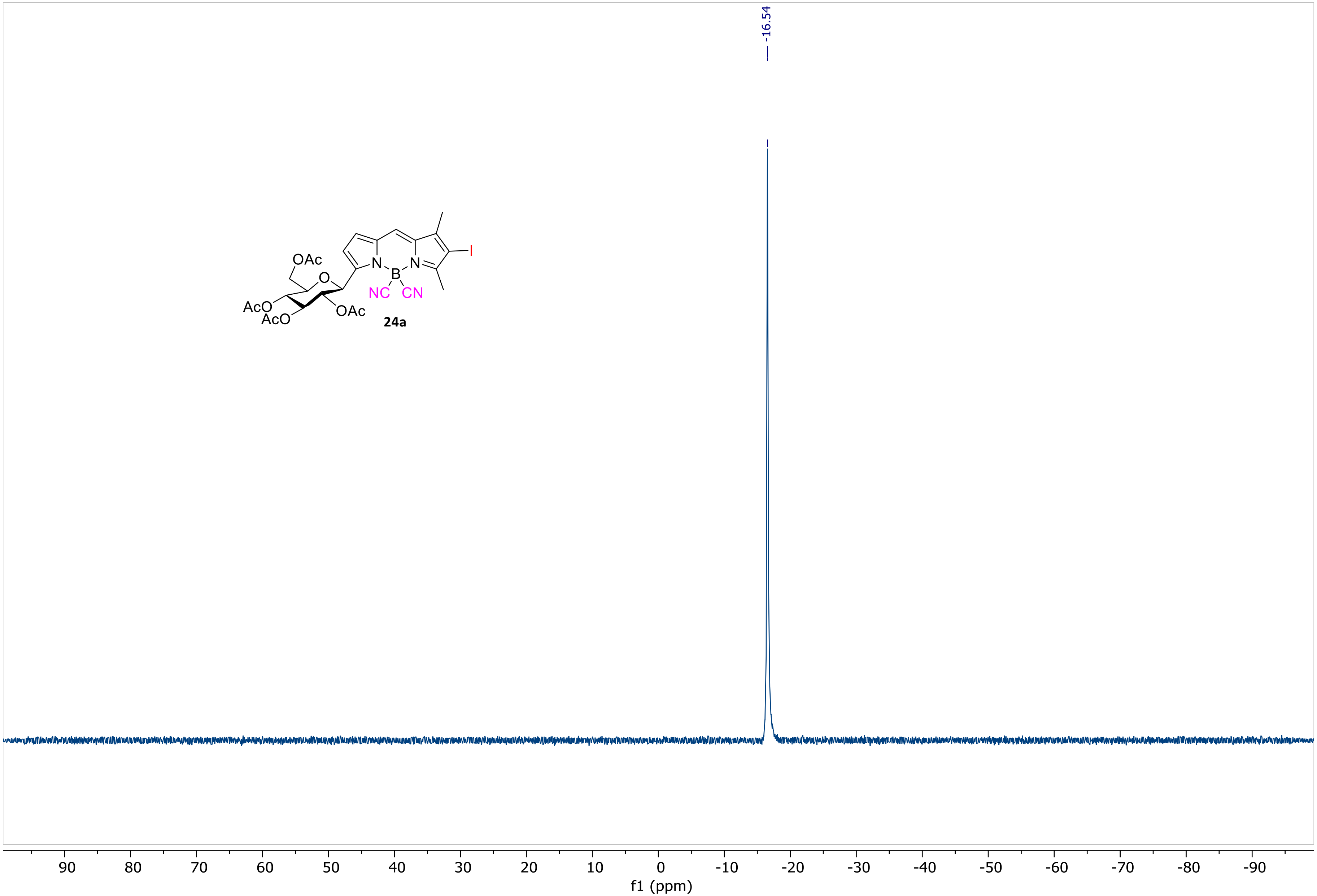

11B NMR CDCl3 128 MHz

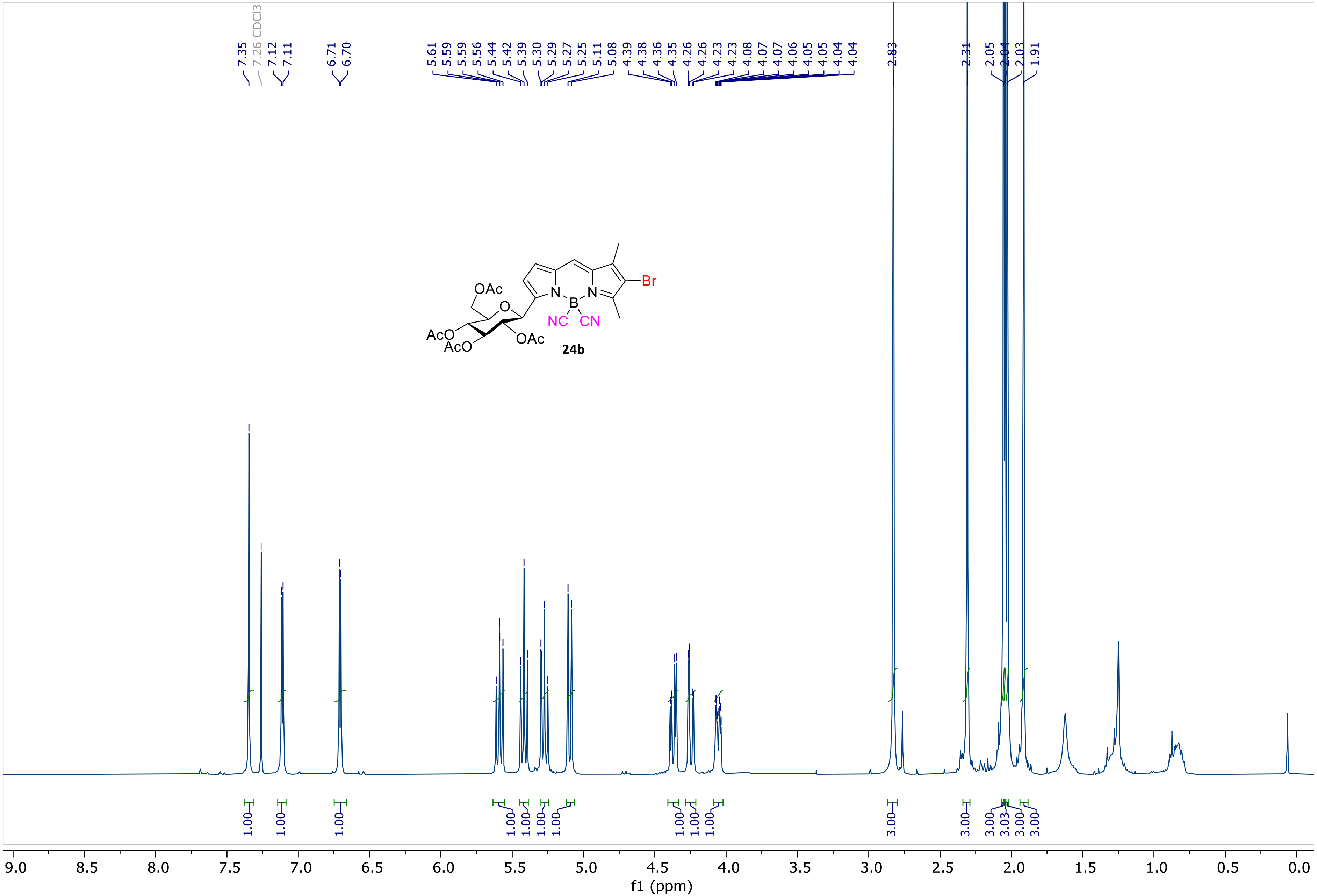

1H NMR 400 MHz CDCl3

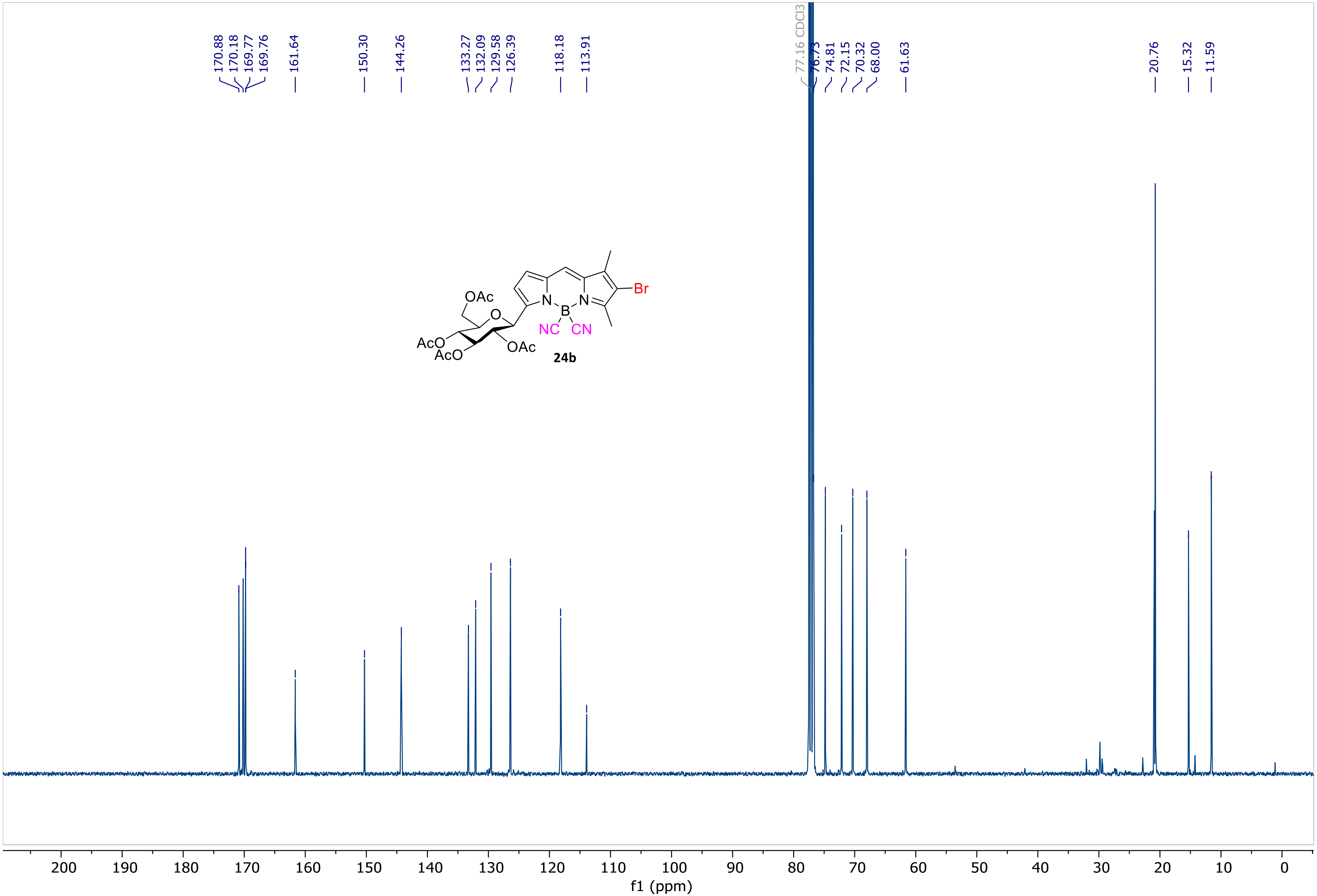

13C{1H} NMR CDCl3 125 MHz

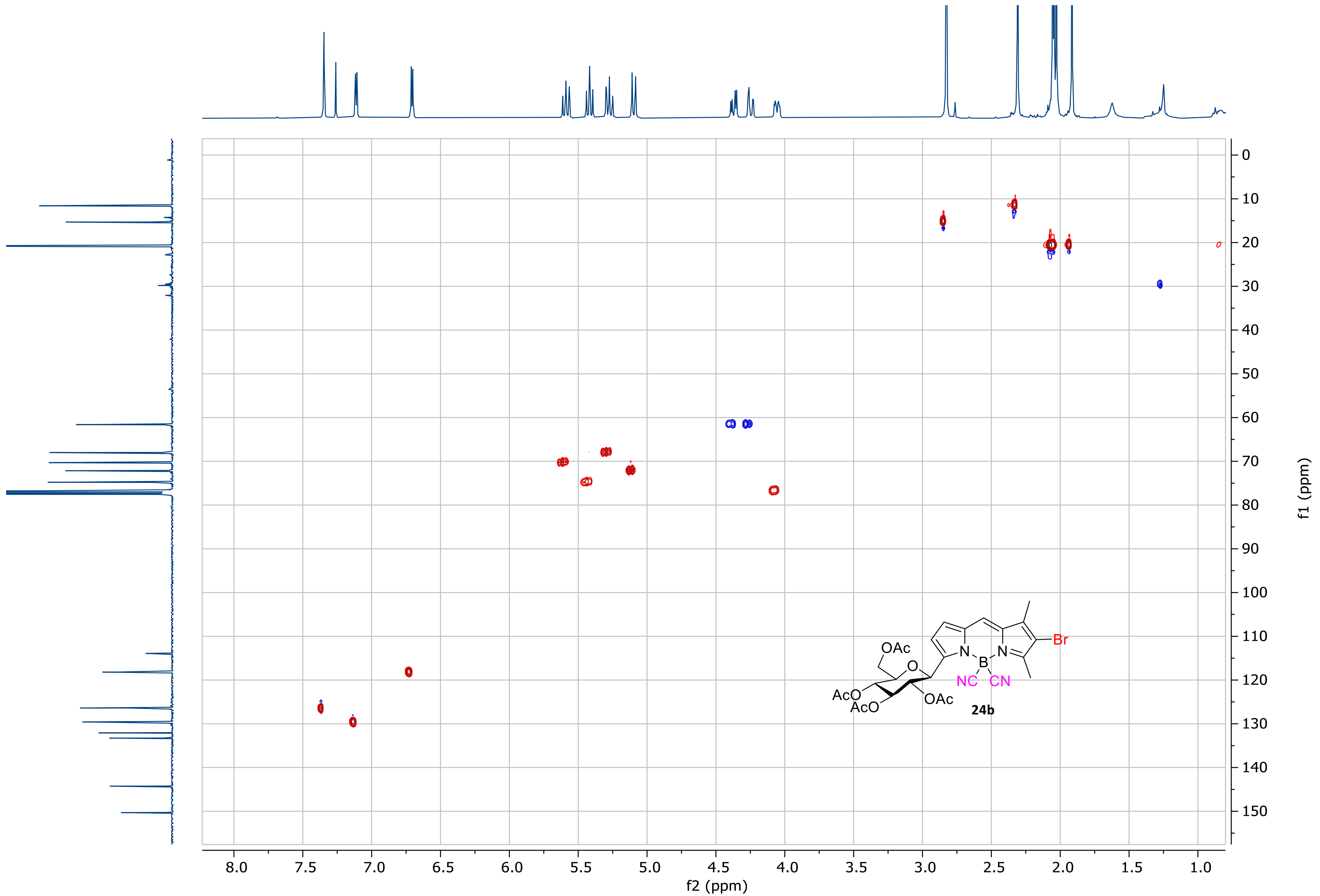

1H-13C gHSQC CDCl3

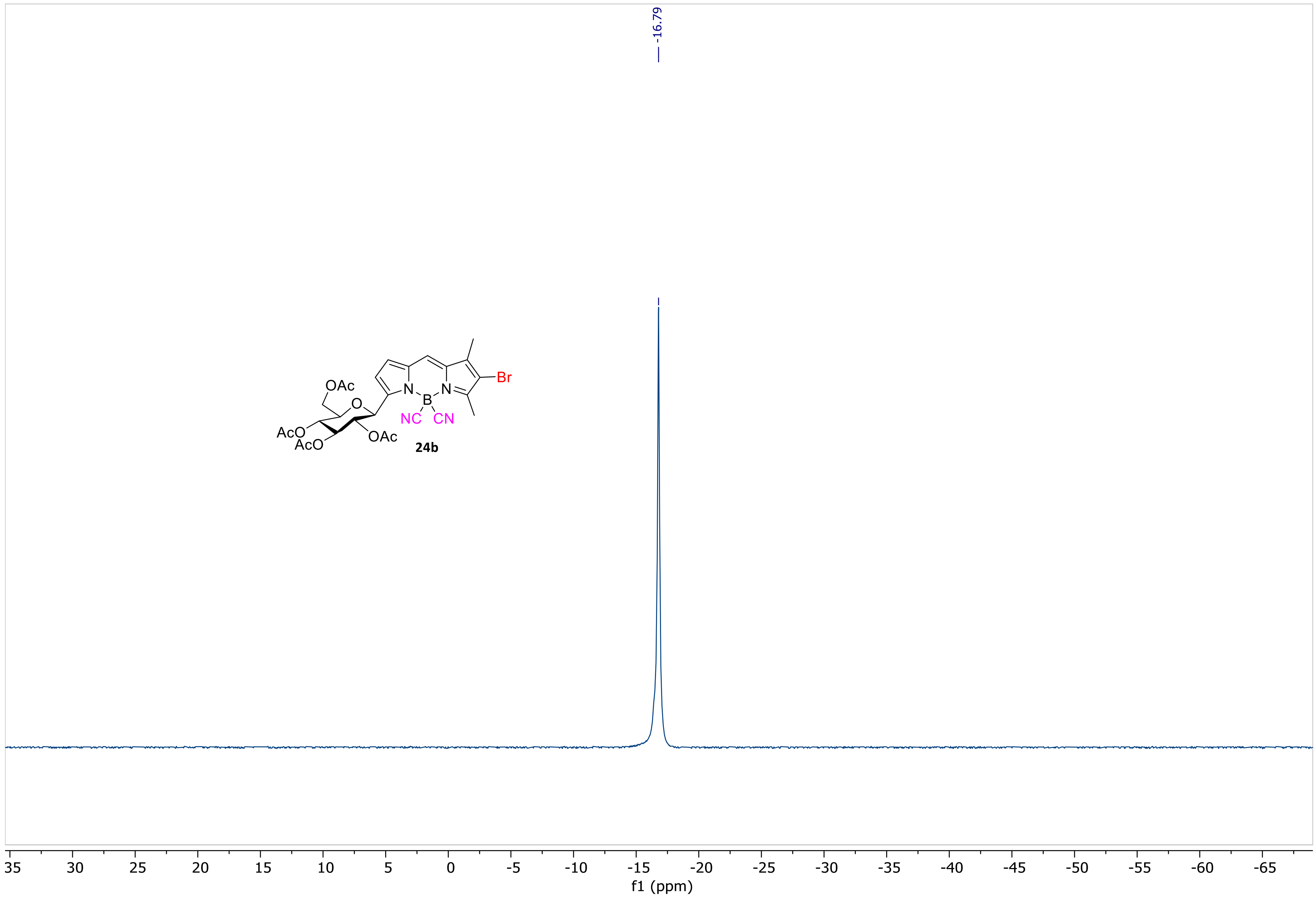

11B NMR CDCl3 128 MHz

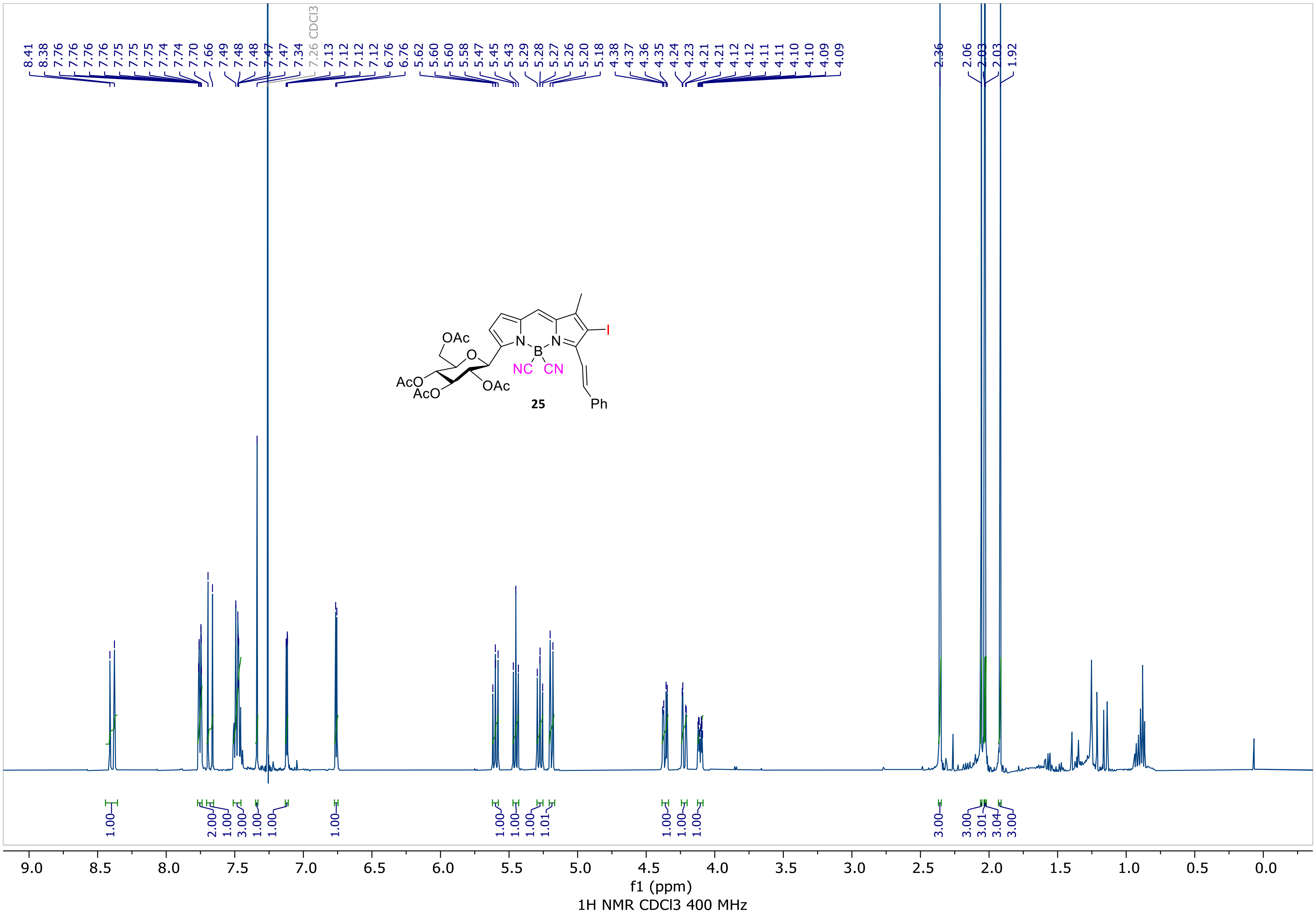
8.41 8.38 7.76 7.76 7.76 7.76 7.75 7.75 7.75 7.74 7.74 7.70 7.66 7.49 7.48 7.48 7.47 7.47 7.34 7.26 CDCl3 7.13 7.12 7.12 7.12 6.76 6.76 5.62 5.60 5.60 5.58 5.47 5.45 5.43 5.29 5.28 5.27 5.26 5.20 5.18 4.38 4.37 4.36 4.35 4.24 4.23 4.21 4.21 4.12 4.12 4.11 4.11 4.10 4.10 4.09 4.09
2.36 2.06 2.03 2.03 1.92
OAc
O
AcO
AcO
OAc
N
B
N
NC
CN
I
Ph
25
1.00 2.00 1.00 3.00 1.00 1.00 1.00 1.00 1.00 1.00 1.01 1.00 1.00 1.00 3.00 3.00 3.01 3.04 3.00
9.0 8.5 8.0 7.5 7.0 6.5 6.0 5.5 5.0 4.5 4.0 3.5 3.0 2.5 2.0 1.5 1.0 0.5 0.0
f1 (ppm)
1H NMR CDCl3 400 MHz

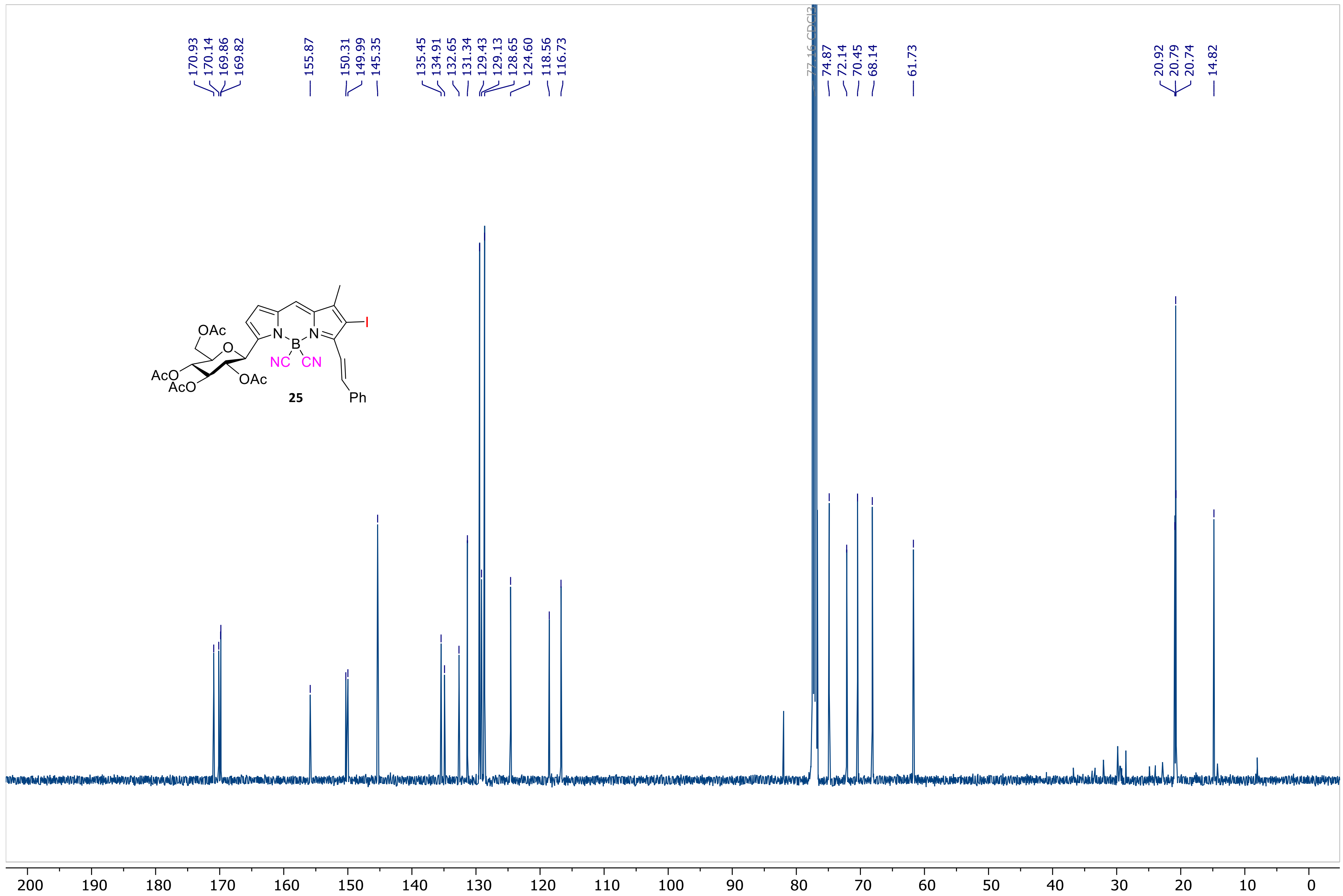

f1 (ppm)

13C{1H} NMR CDCl3 125 MHz

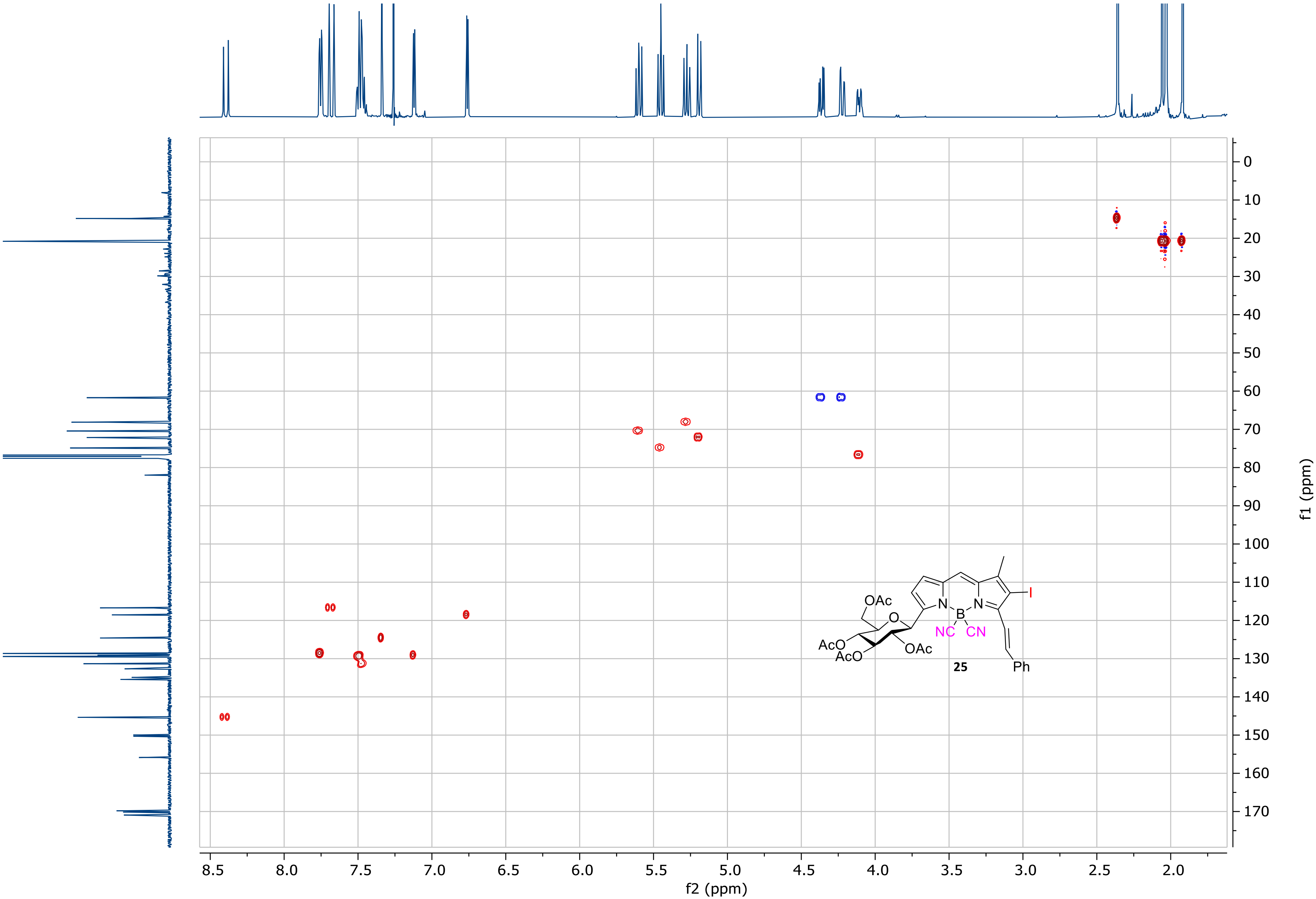

1H-13C gHSQC NMR

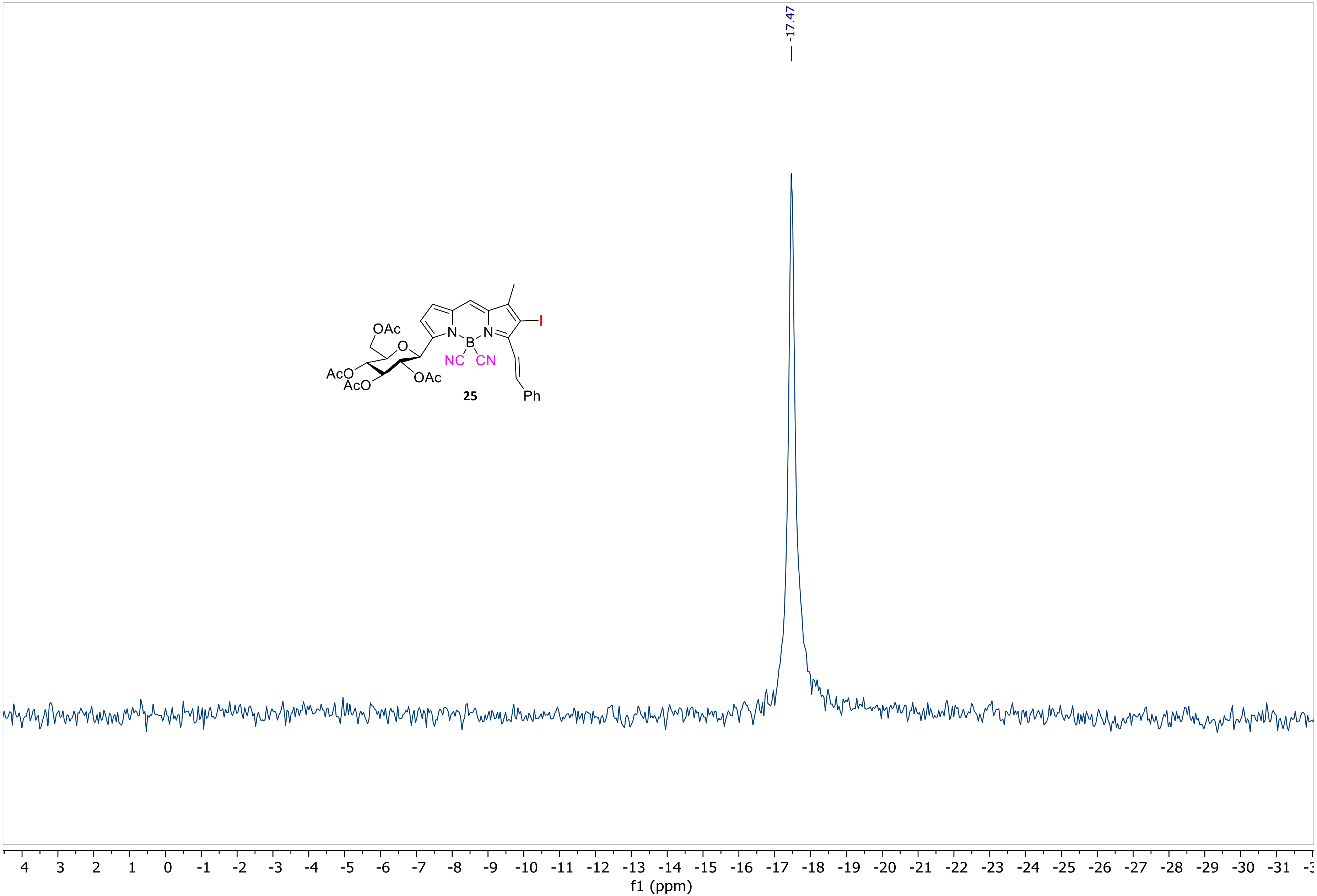

11B NMR CDCl3 128 MHz

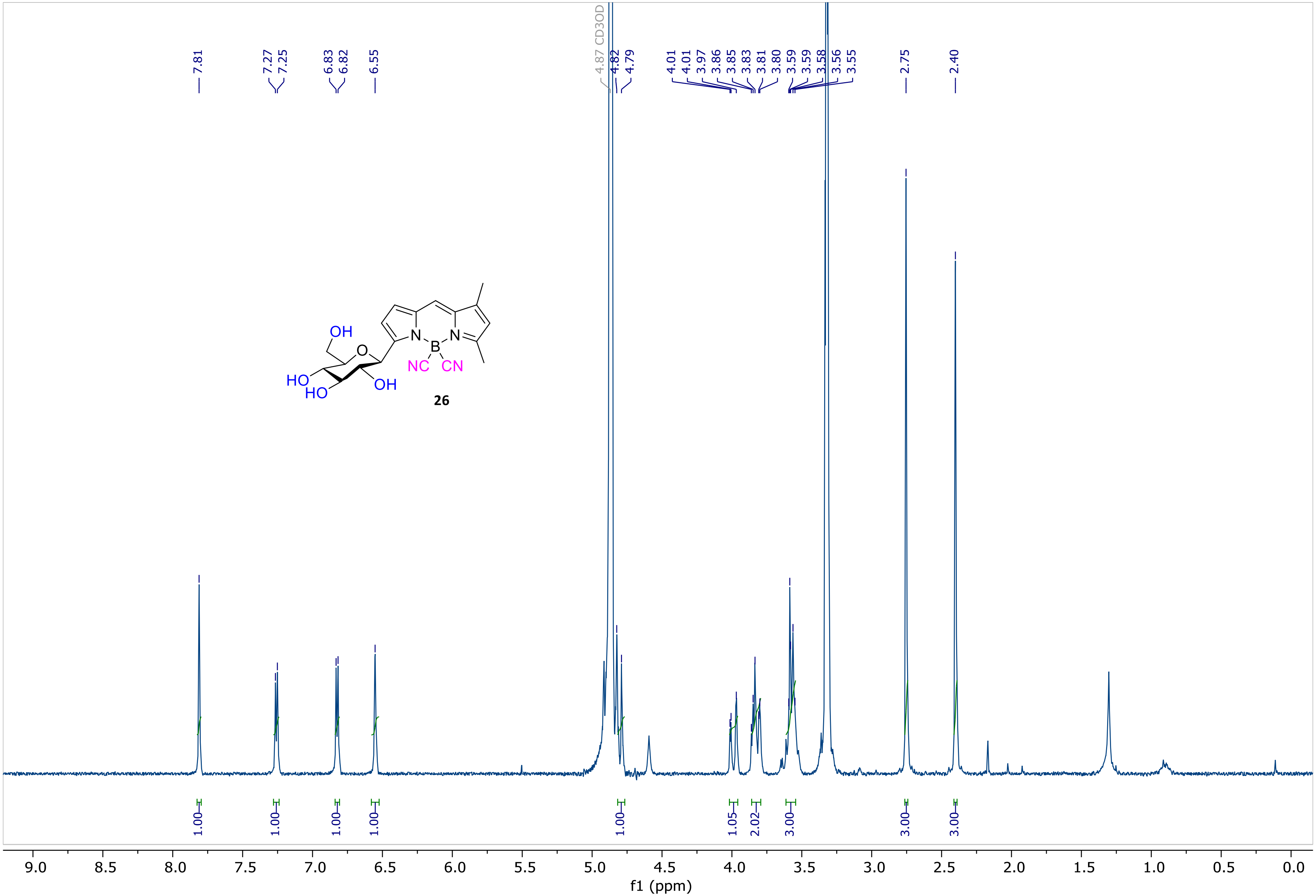

1H NMR CD3OD 400 MHz

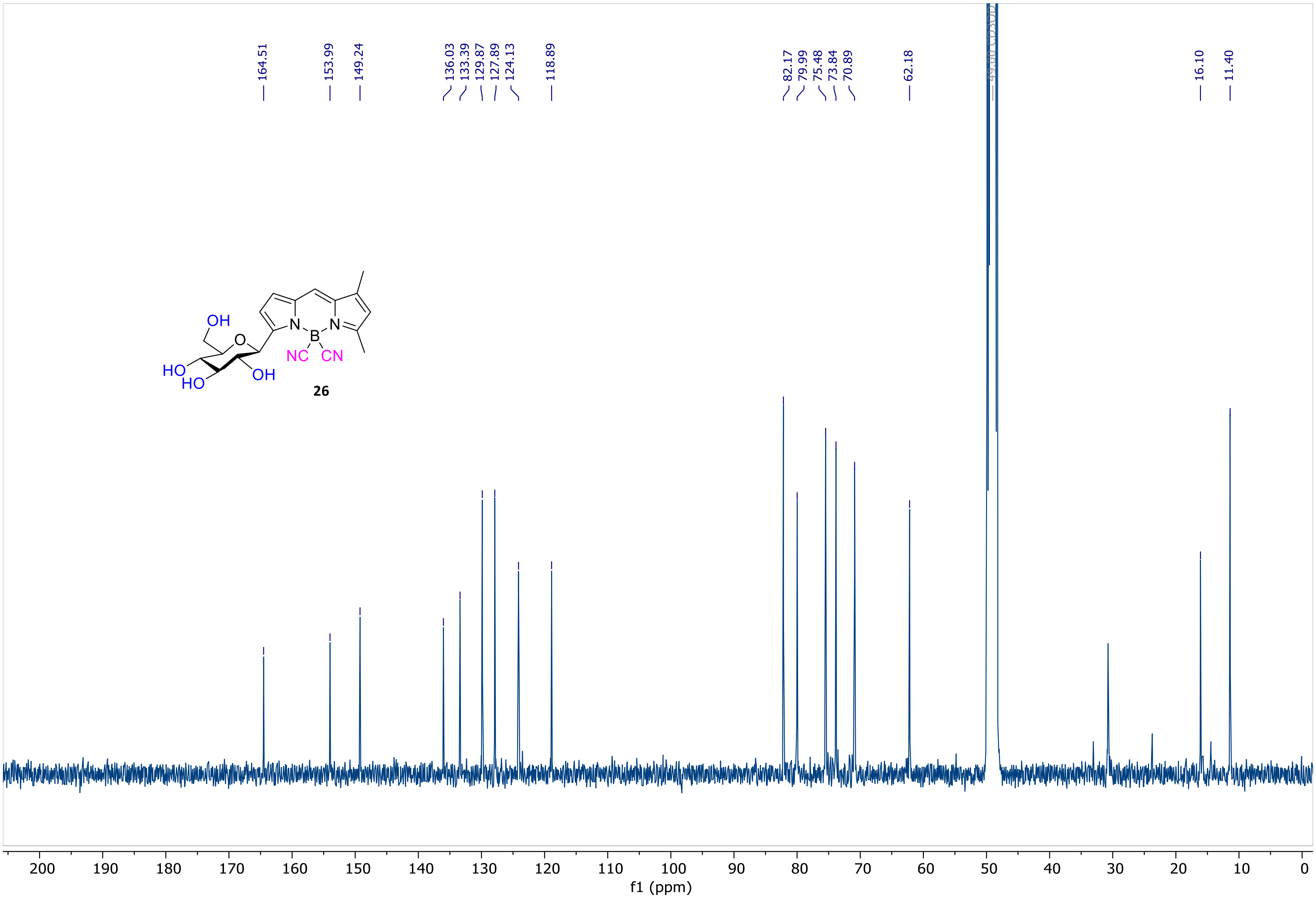

13C{1H} NMR CD3OD 125 MHz

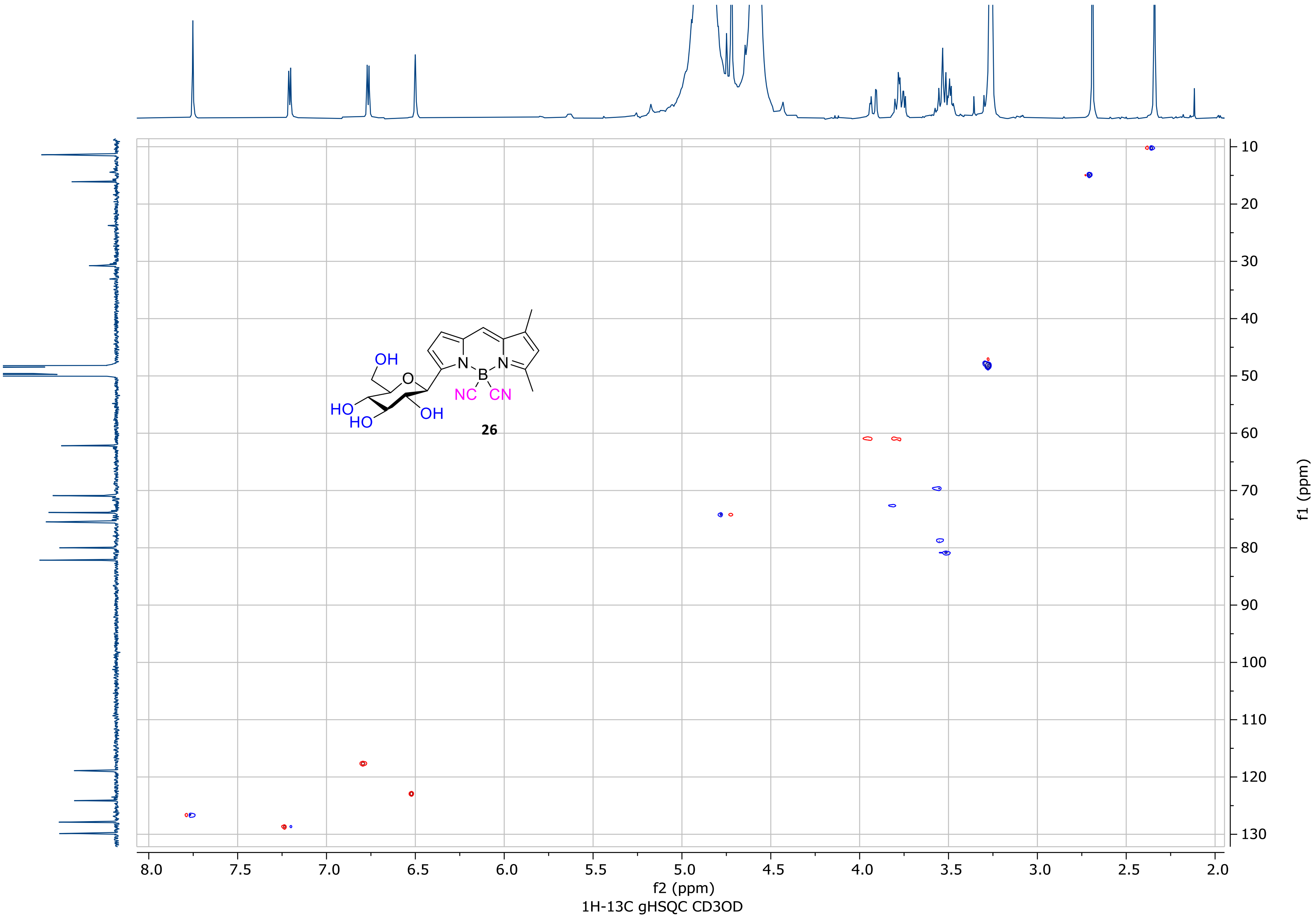

26
f1 (ppm)
f2 (ppm)
1H-13C gHSQC CD3OD

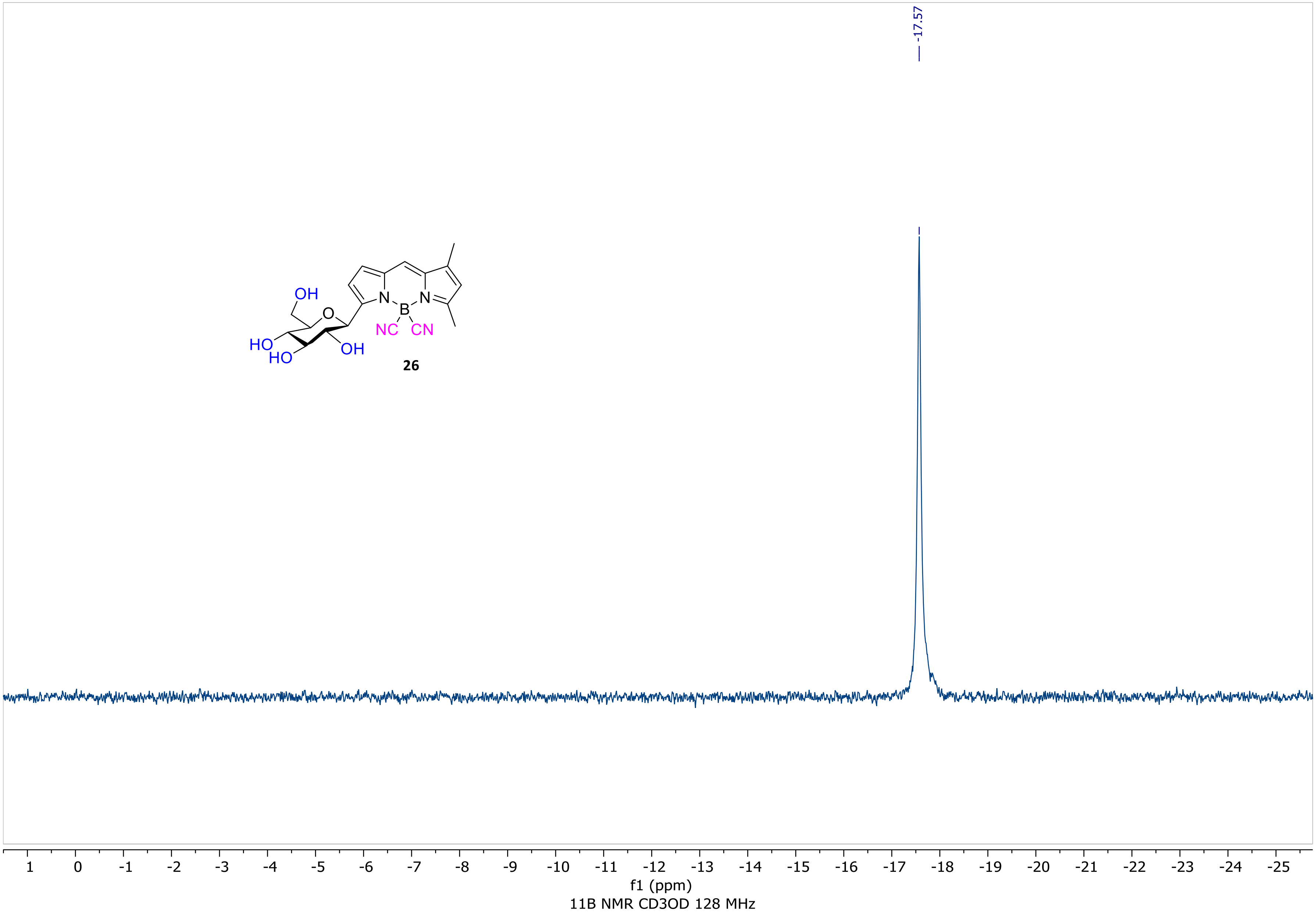
-17.57
OH
O
HO
HO
OH
N
B
N
NC
CN
26
1
0
-1
-2
-3
-4
-5
-6
-7
-8
-9
-10
-11
-12
-13
-14
-15
-16
-17
-18
-19
-20
-21
-22
-23
-24
-25
f1 (ppm)
11B NMR CD3OD 128 MHz

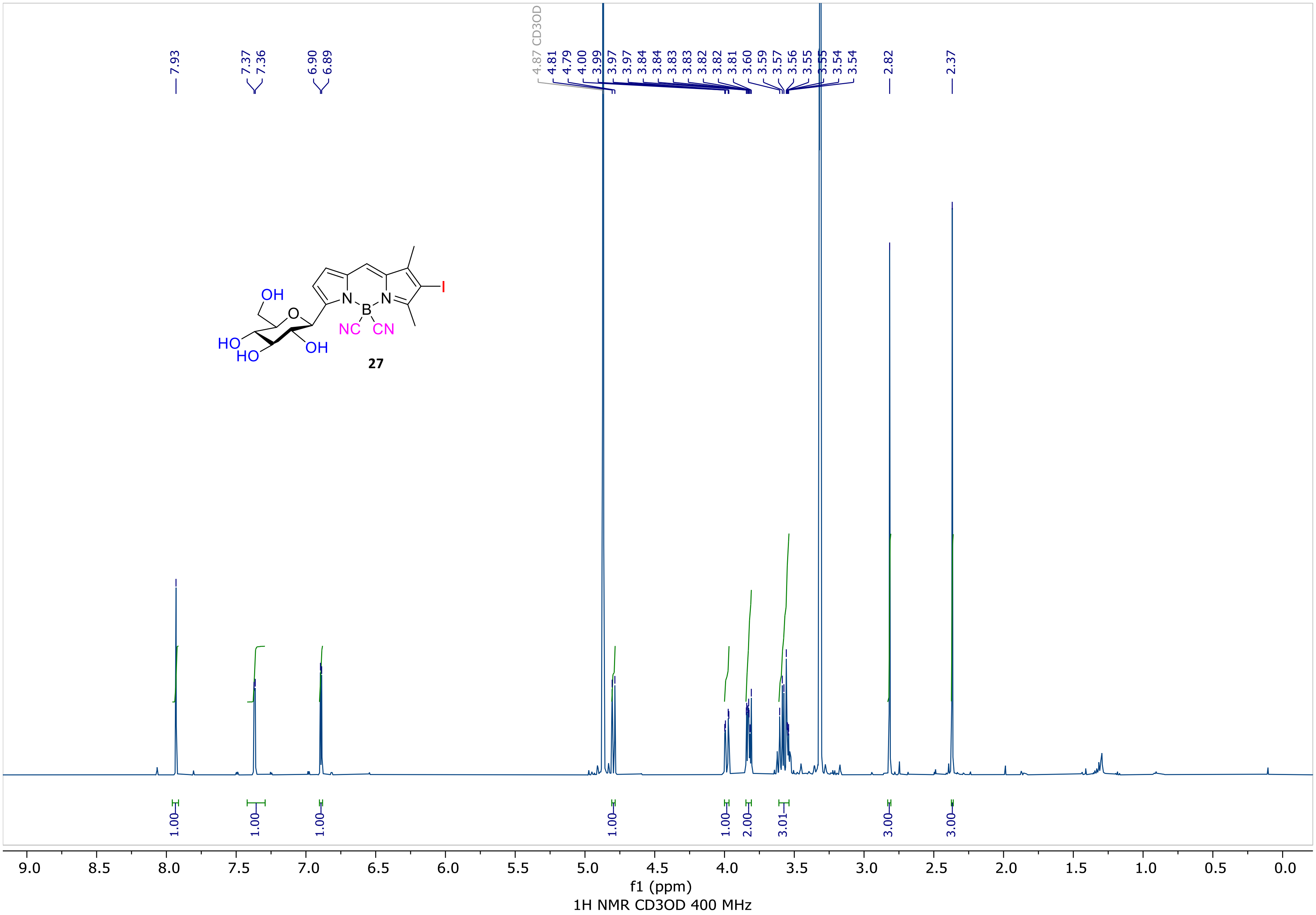
4.87 CD3OD
7.93
7.37
7.36
6.90
6.89
4.81
4.79
4.00
3.99
3.97
3.97
3.84
3.84
3.83
3.83
3.82
3.82
3.81
3.60
3.59
3.57
3.56
3.55
3.55
3.54
3.54
2.82
2.37
OH
O
HO
HO
OH
N
B
N
NC
CN
I
27
1.00
1.00
1.00
1.00
1.00
2.00
3.01
3.00
3.00
9.0
8.5
8.0
7.5
7.0
6.5
6.0
5.5
5.0
4.5
4.0
3.5
3.0
2.5
2.0
1.5
1.0
0.5
0.0
f1 (ppm)
1H NMR CD3OD 400 MHz

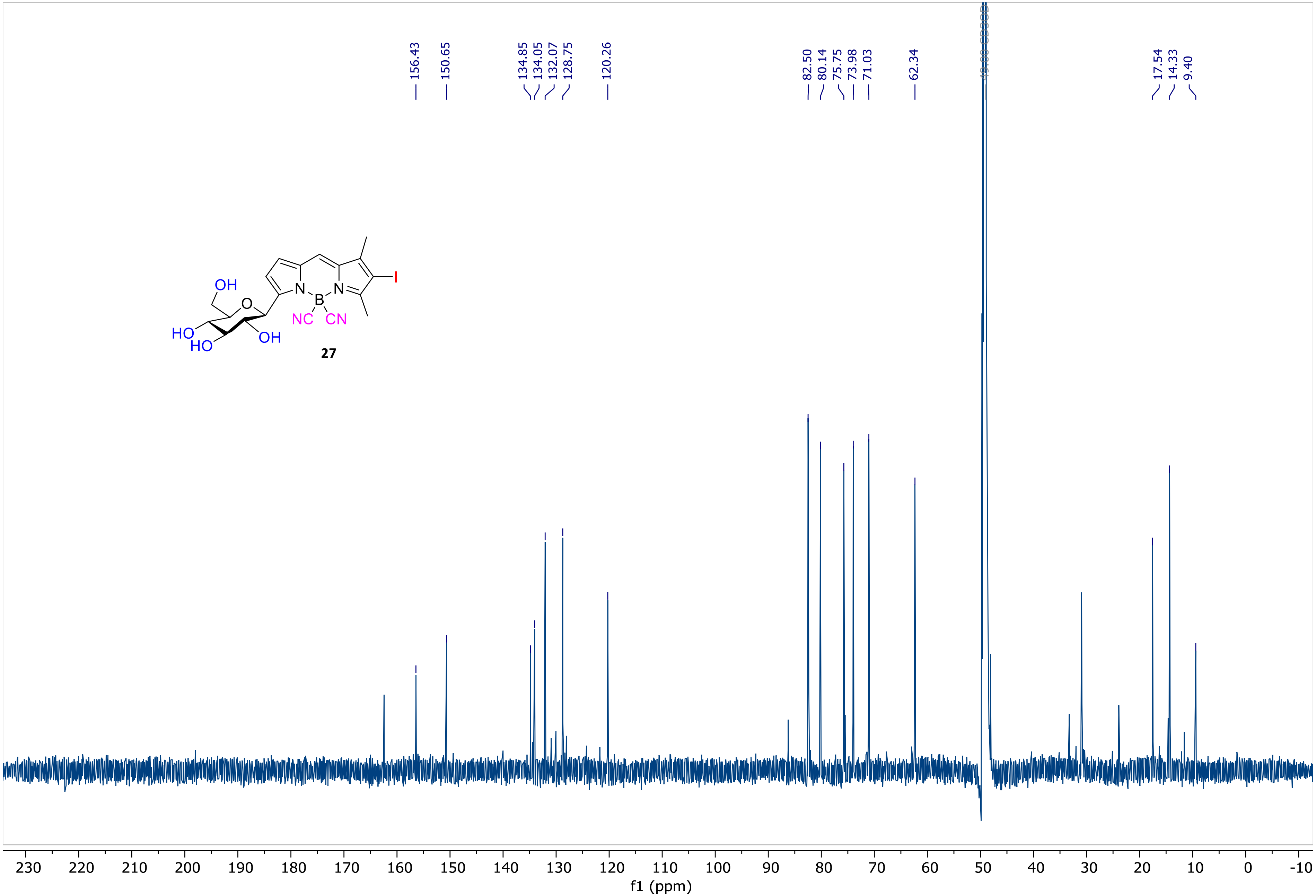

13C{1H} NMR CD3OD 125 MHz

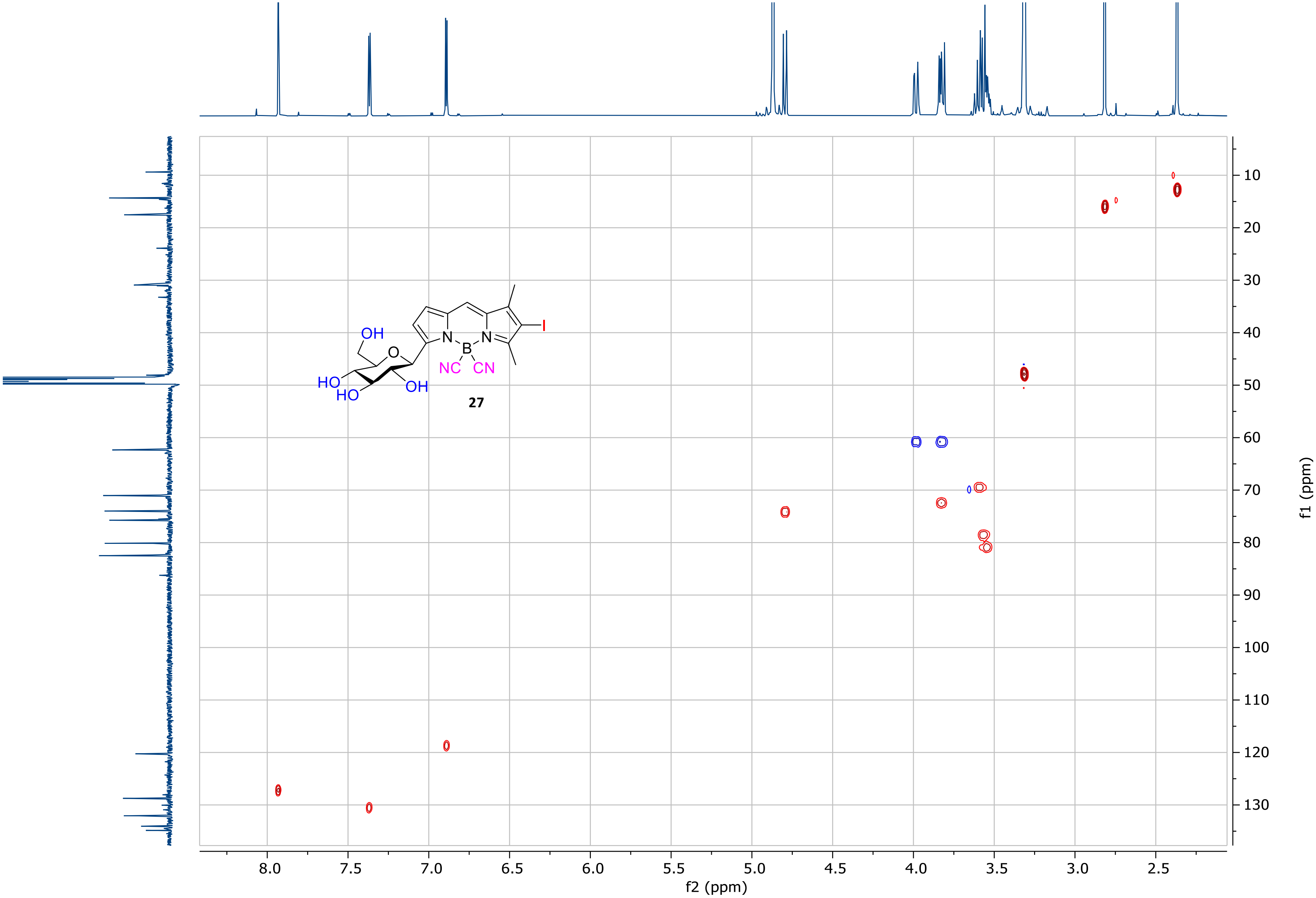

1H-13C gHSQC CD3OD

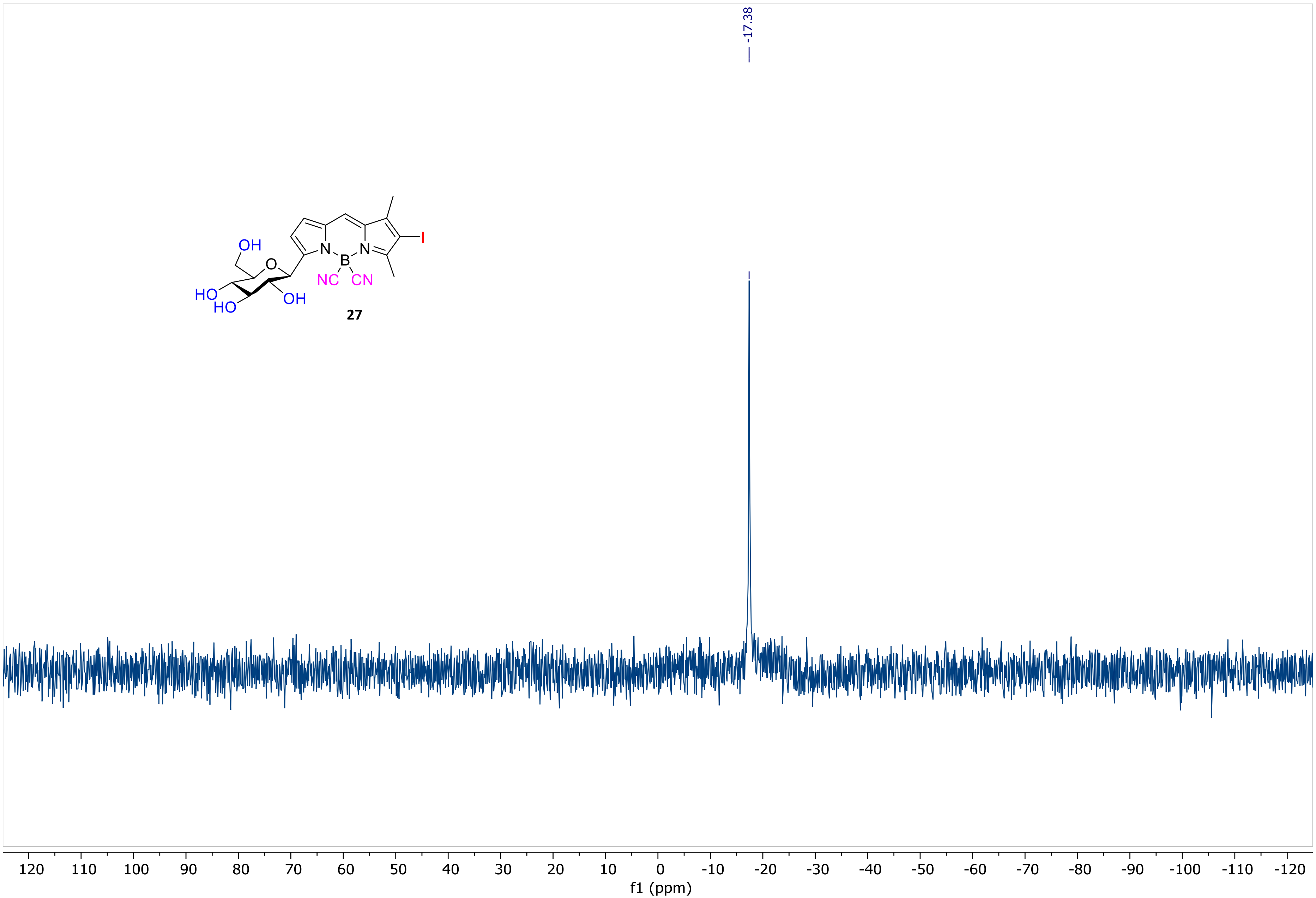

11B NMR CD3OD 128 MHz

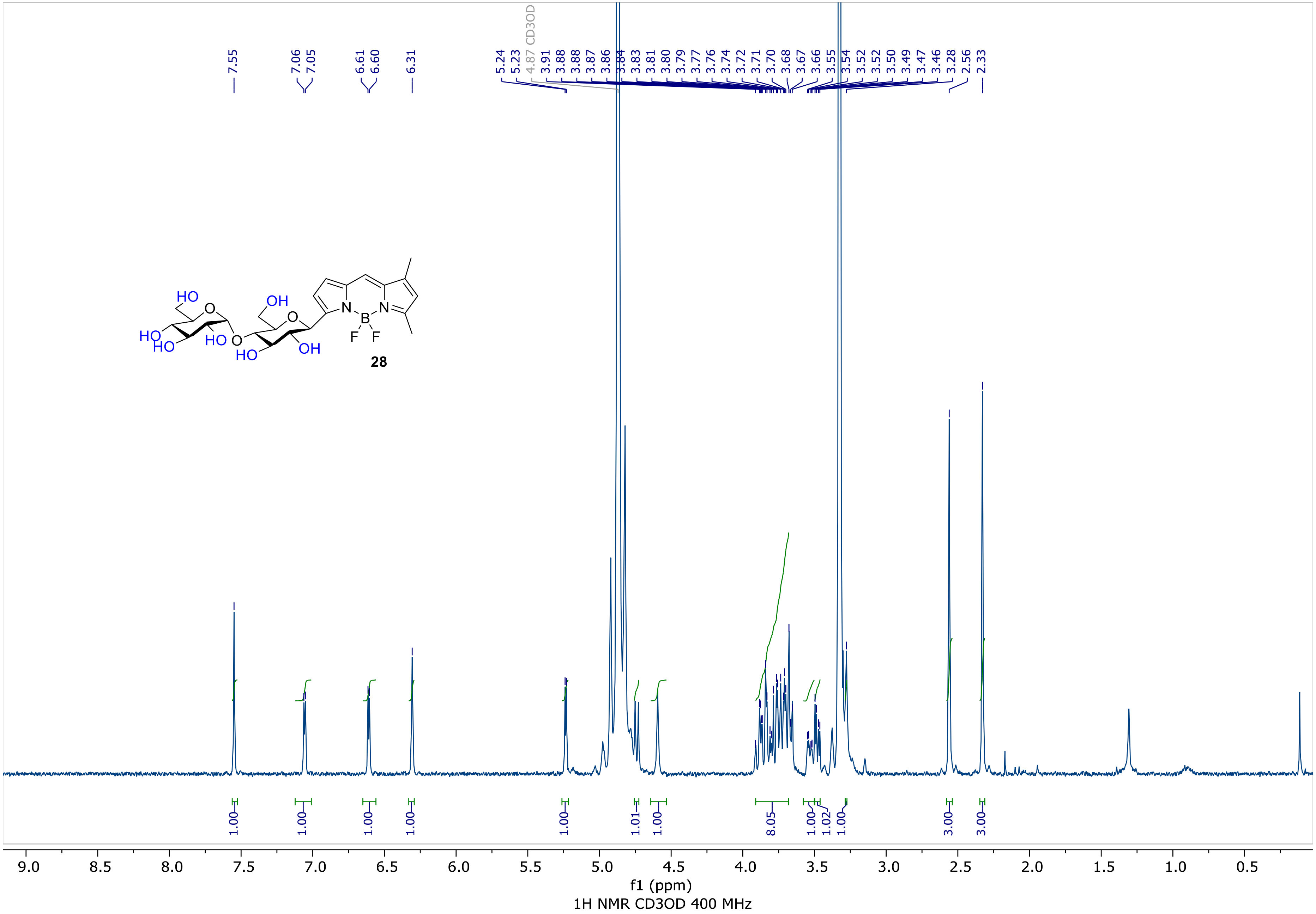

7.55
7.06
7.05
6.61
6.60
6.31
5.24
5.23
4.87 CD3OD
3.91
3.88
3.88
3.87
3.86
3.84
3.83
3.81
3.80
3.79
3.77
3.76
3.74
3.72
3.71
3.70
3.68
3.67
3.66
3.55
3.54
3.52
3.52
3.50
3.49
3.47
3.46
3.28
2.56
2.33
HO
OH
O
O
HO
HO
HO
O
HO
OH
N
B
N
F
F
28
1.00
1.00
1.00
1.00
1.00
1.01
1.00
8.05
1.00
1.02
1.00
3.00
3.00
9.0
8.5
8.0
7.5
7.0
6.5
6.0
5.5
5.0
4.5
4.0
3.5
3.0
2.5
2.0
1.5
1.0
0.5
f1 (ppm)
1H NMR CD3OD 400 MHz

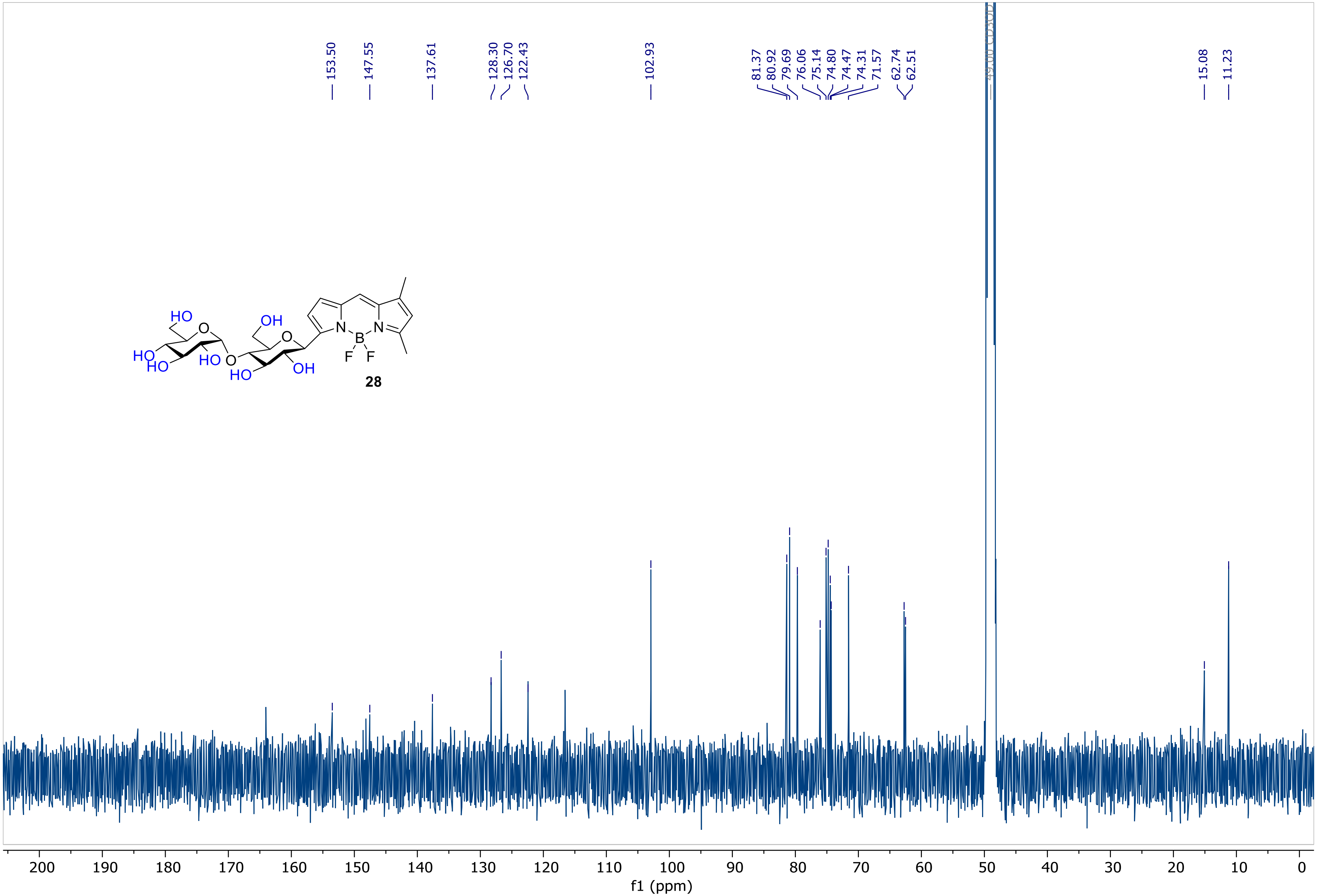

13C{1H} NMR CD3OD 125 MHz

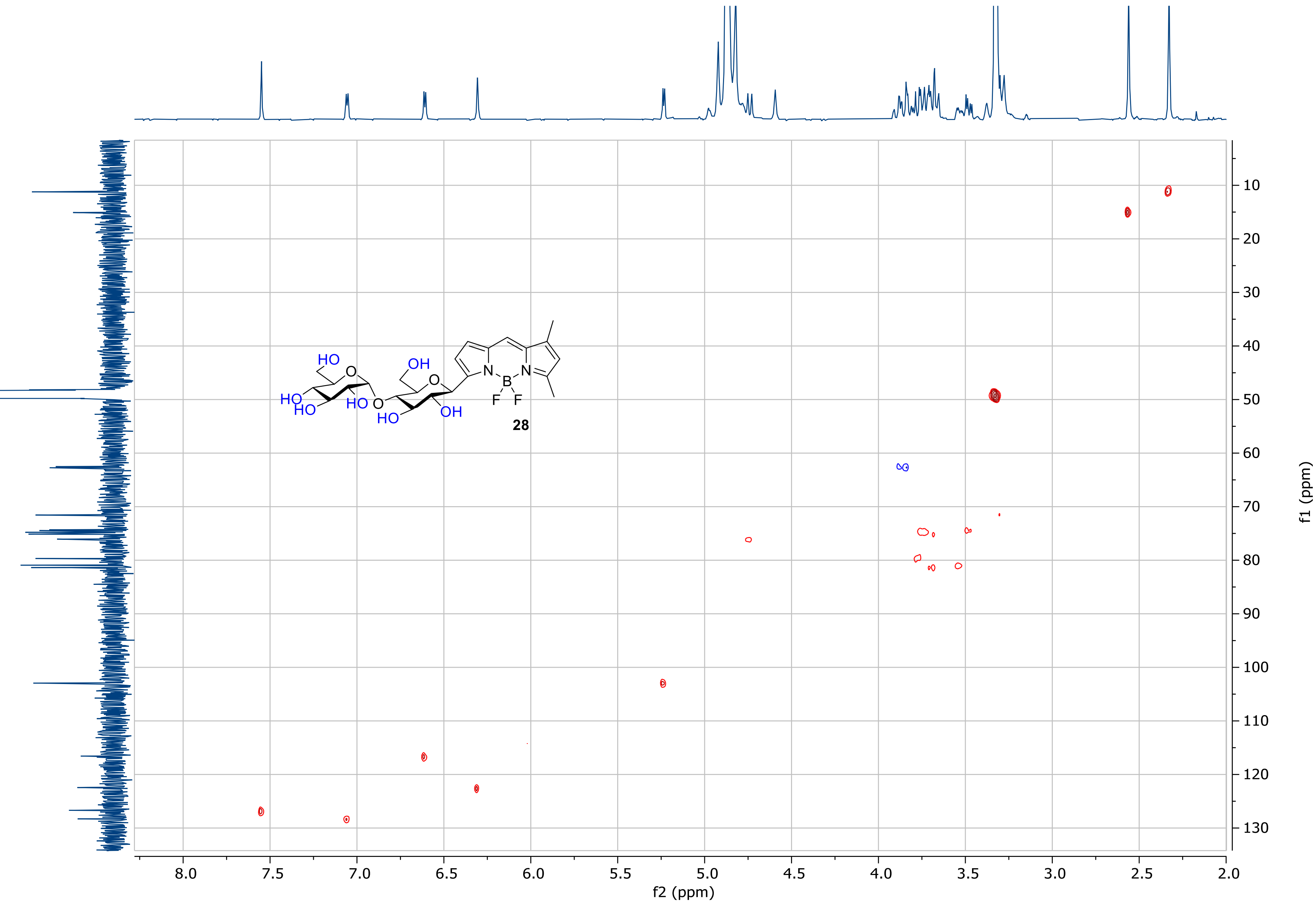

1H-13C gHSQC CD3OD

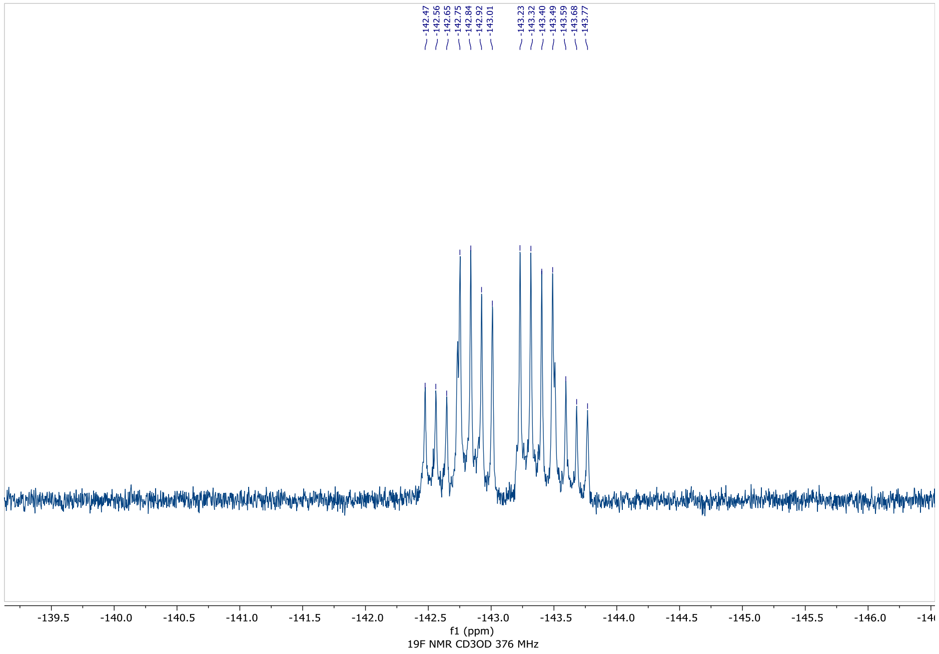
-142.47
-142.56
-142.65
-142.75
-142.84
-142.92
-143.01
-143.23
-143.32
-143.40
-143.49
-143.59
-143.68
-143.77
-139.5 -140.0 -140.5 -141.0 -141.5 -142.0 -142.5 -143.0 -143.5 -144.0 -144.5 -145.0 -145.5 -146.0
f1 (ppm)
19F NMR CD3OD 376 MHz

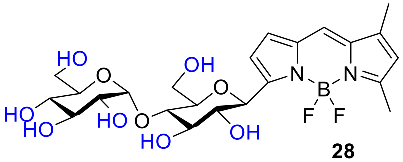
HO
HO
HO
HO
O
O
OH
O
HO
OH
N
B
N
F
F

28

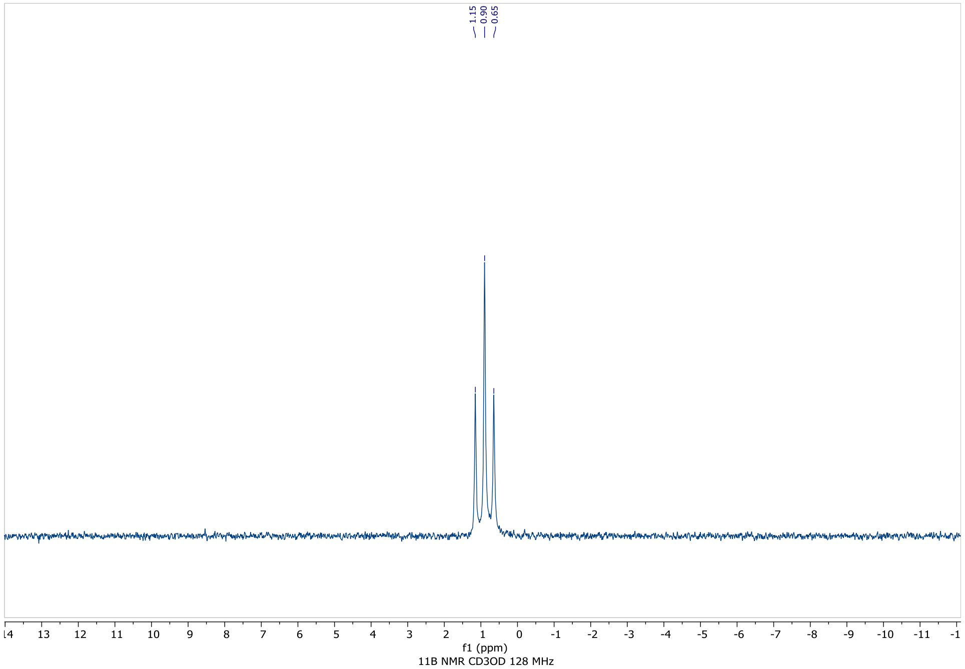
1.15
0.90
0.65
13 12 11 10 9 8 7 6 5 4 3 2 1 0 -1 -2 -3 -4 -5 -6 -7 -8 -9 -10 -11
f1 (ppm)
11B NMR CD3OD 128 MHz

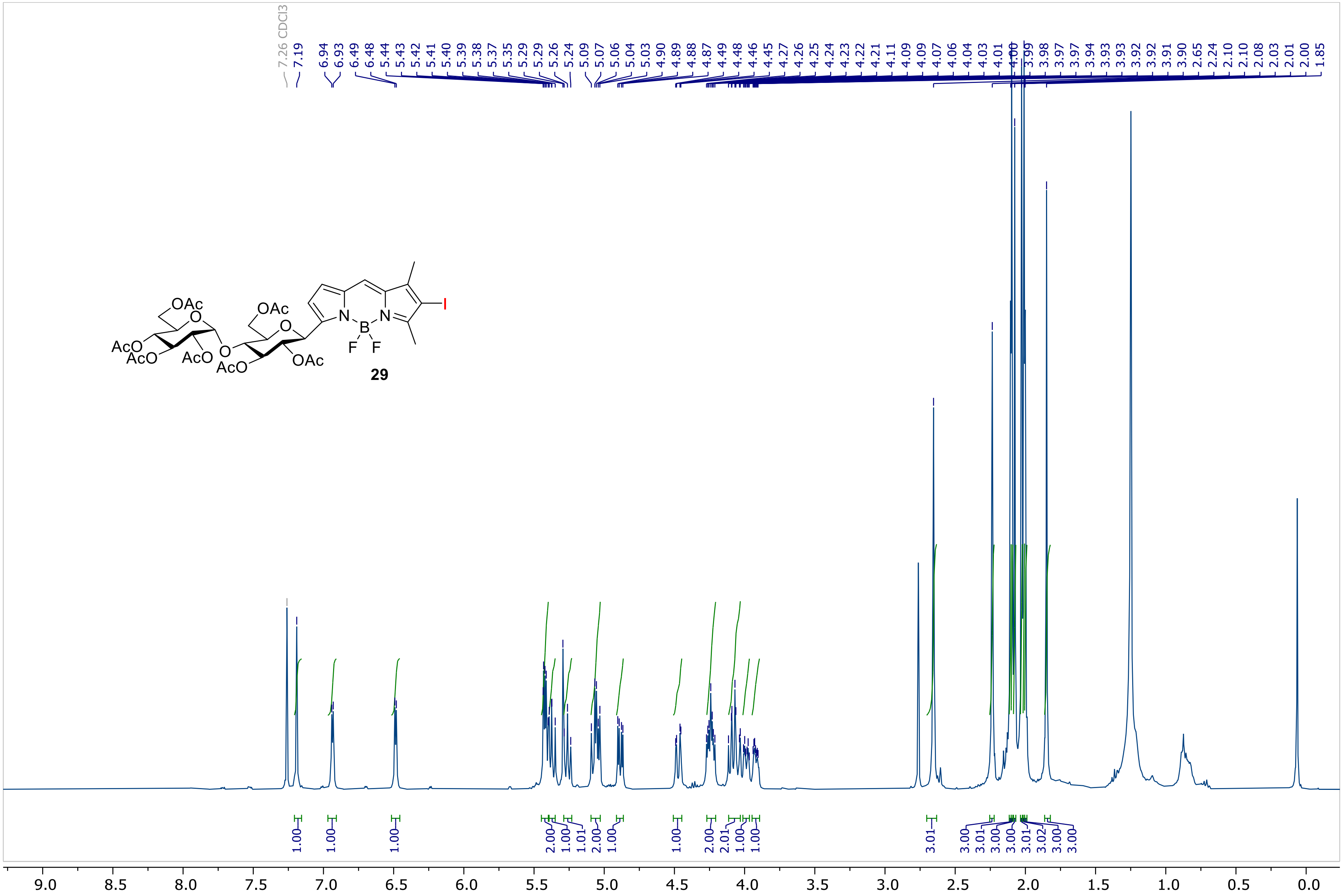

f1 (ppm)
1H NMR CDCl3 400 MHz

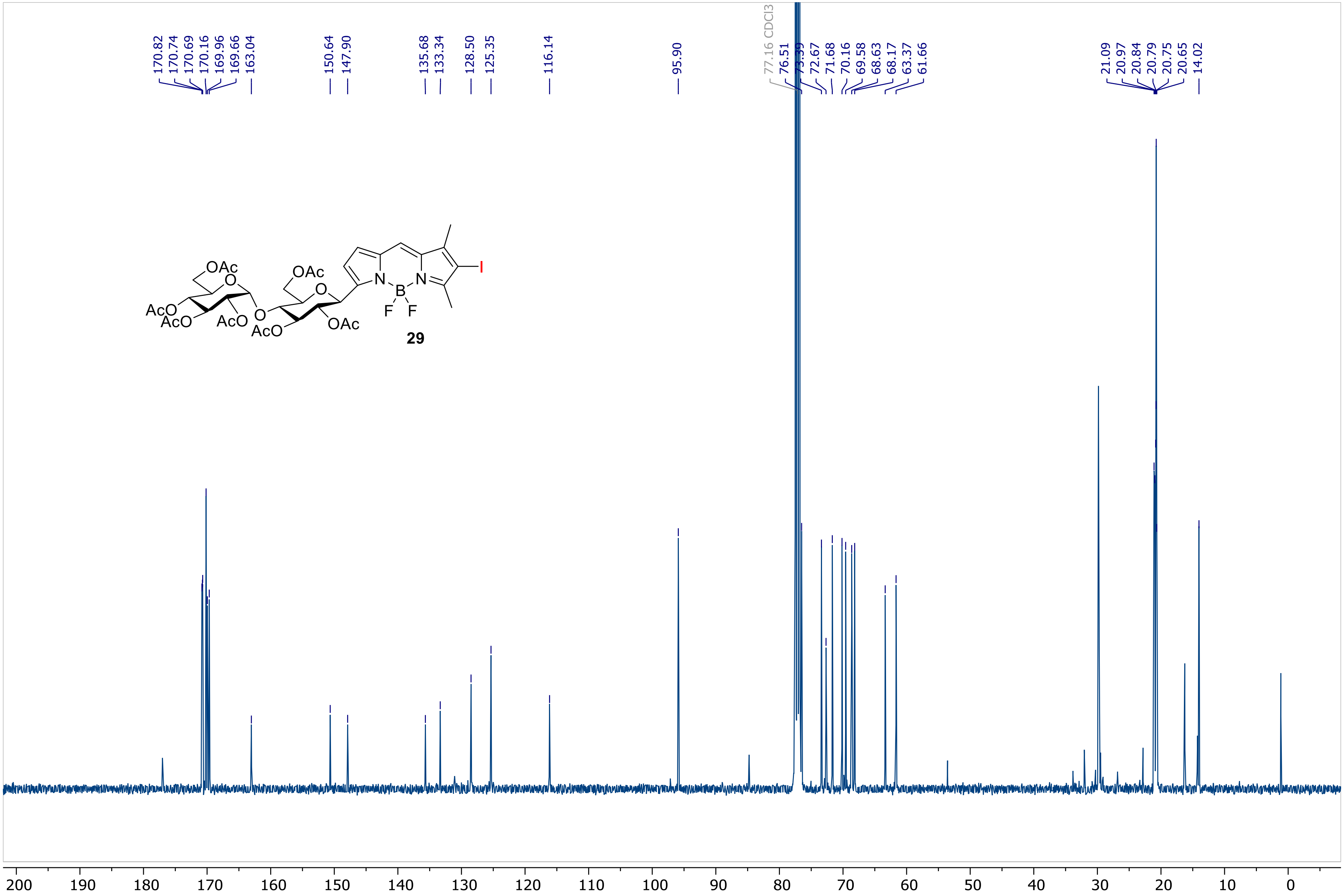

13C{1H} NMR CDCl3 125 MHz

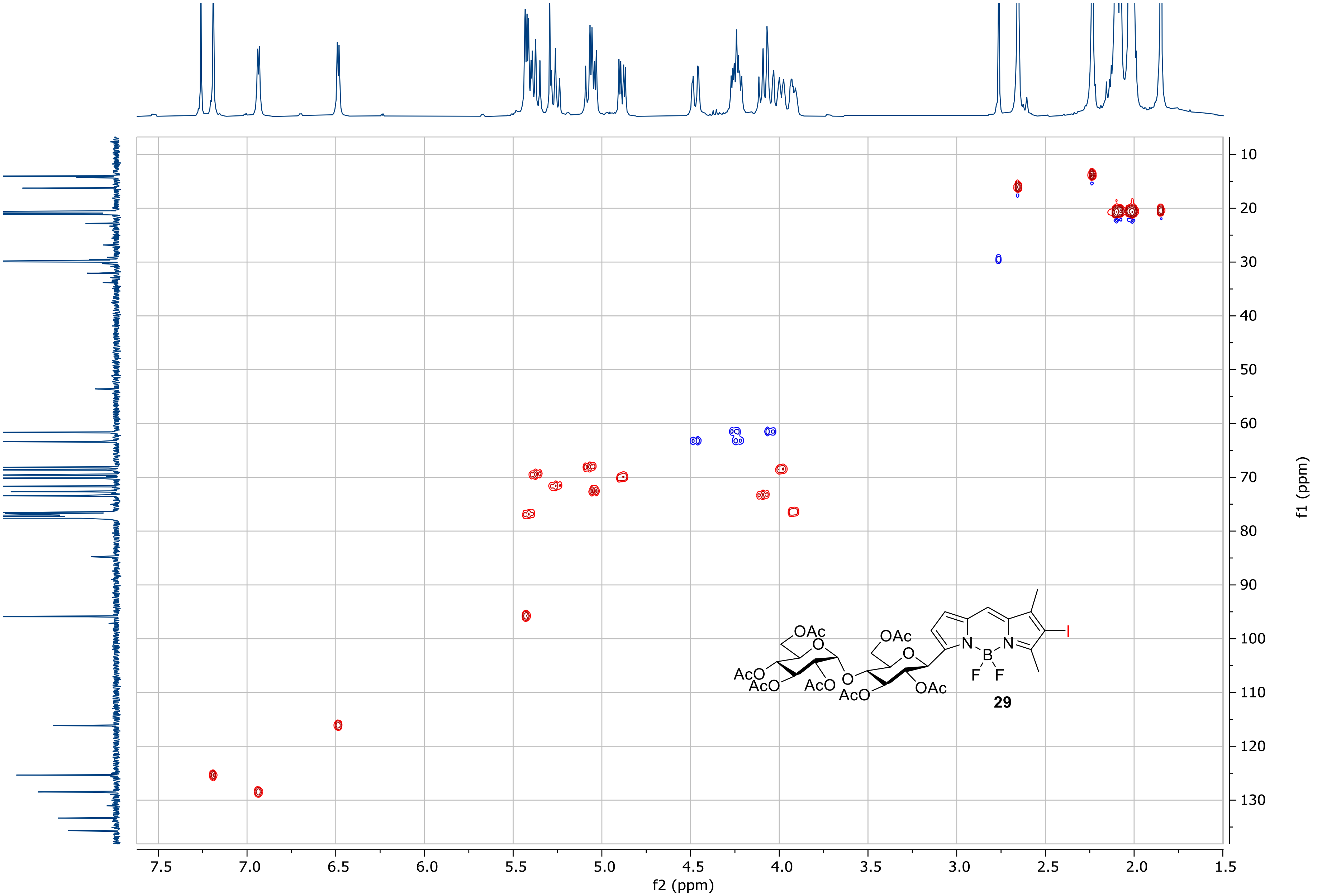

13C-1H gHSQC CDCl3

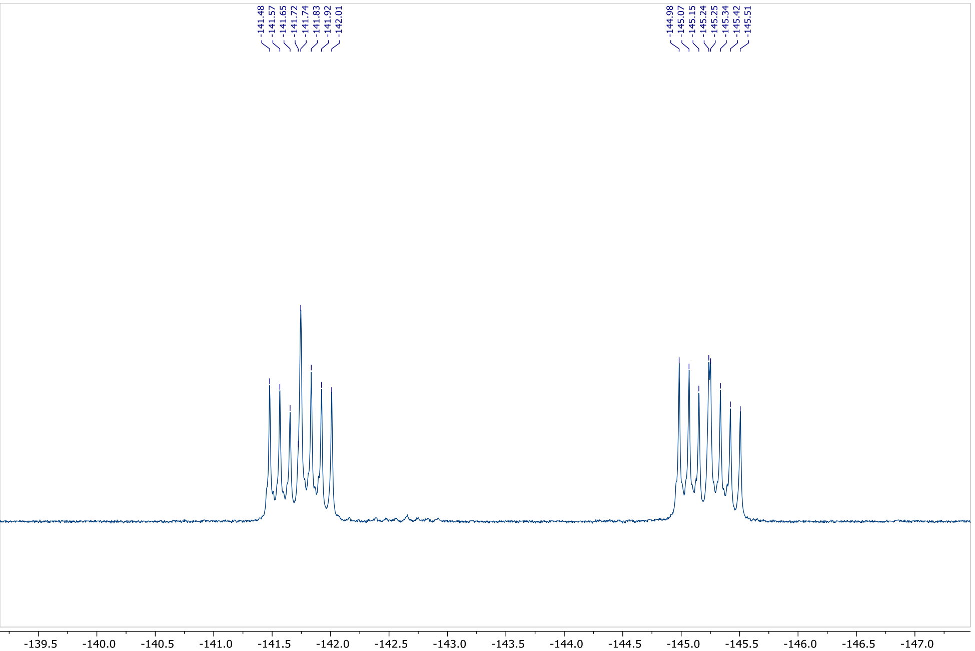

f1 (ppm)
19F NMR CDCl3 376 MHz

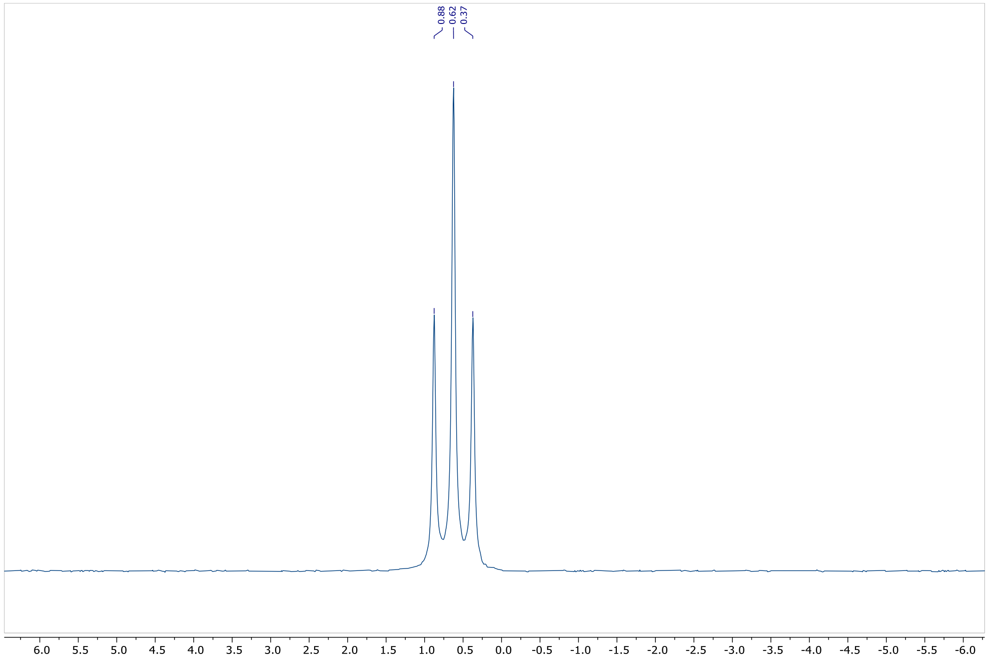

f1 (ppm)
11B NMR CDCl3 128 MHz

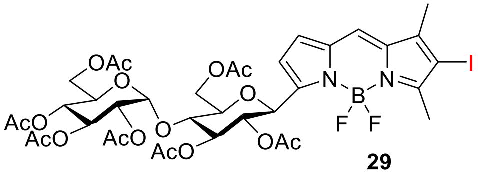

29

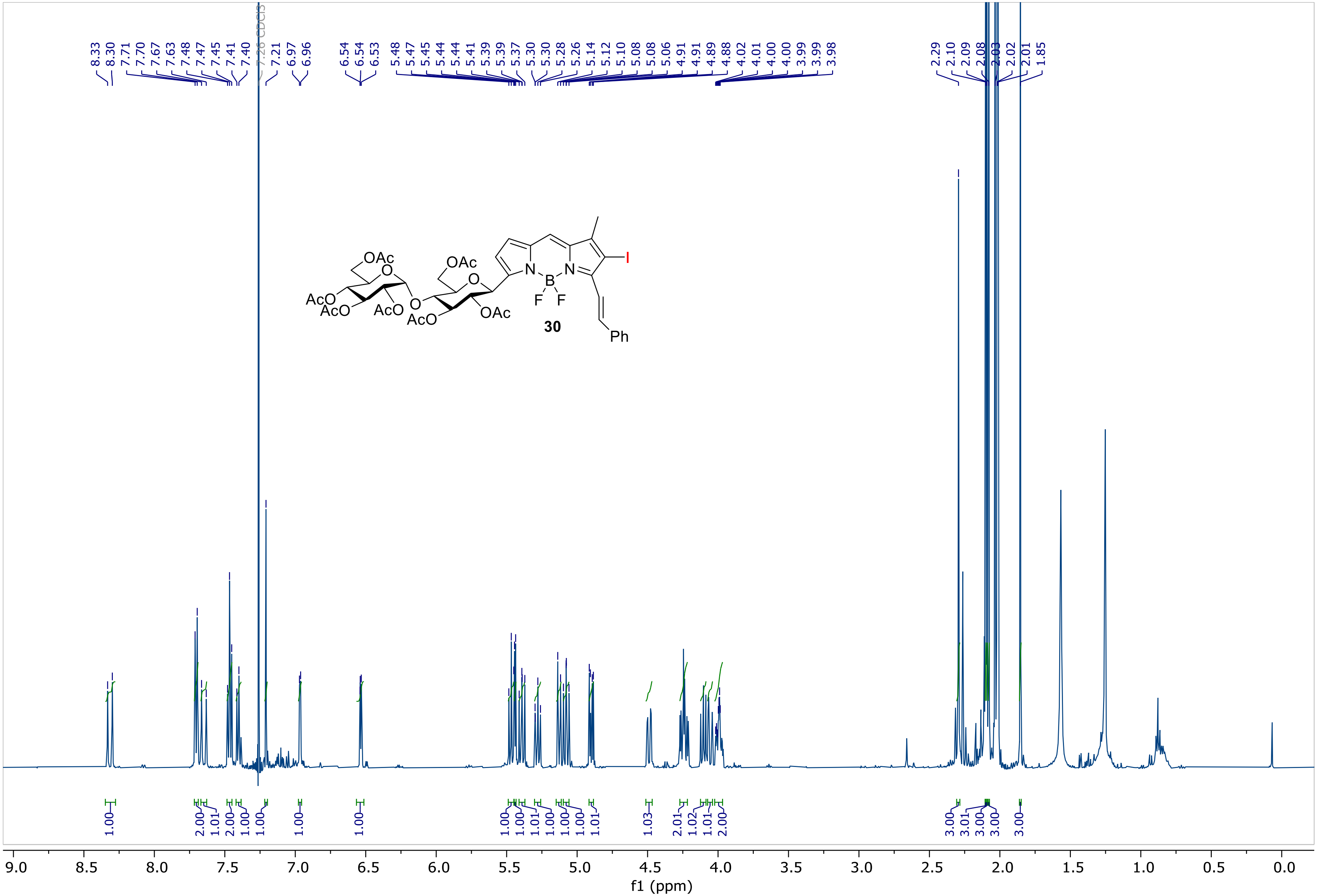

1H NMR CDCl3 400 MHz

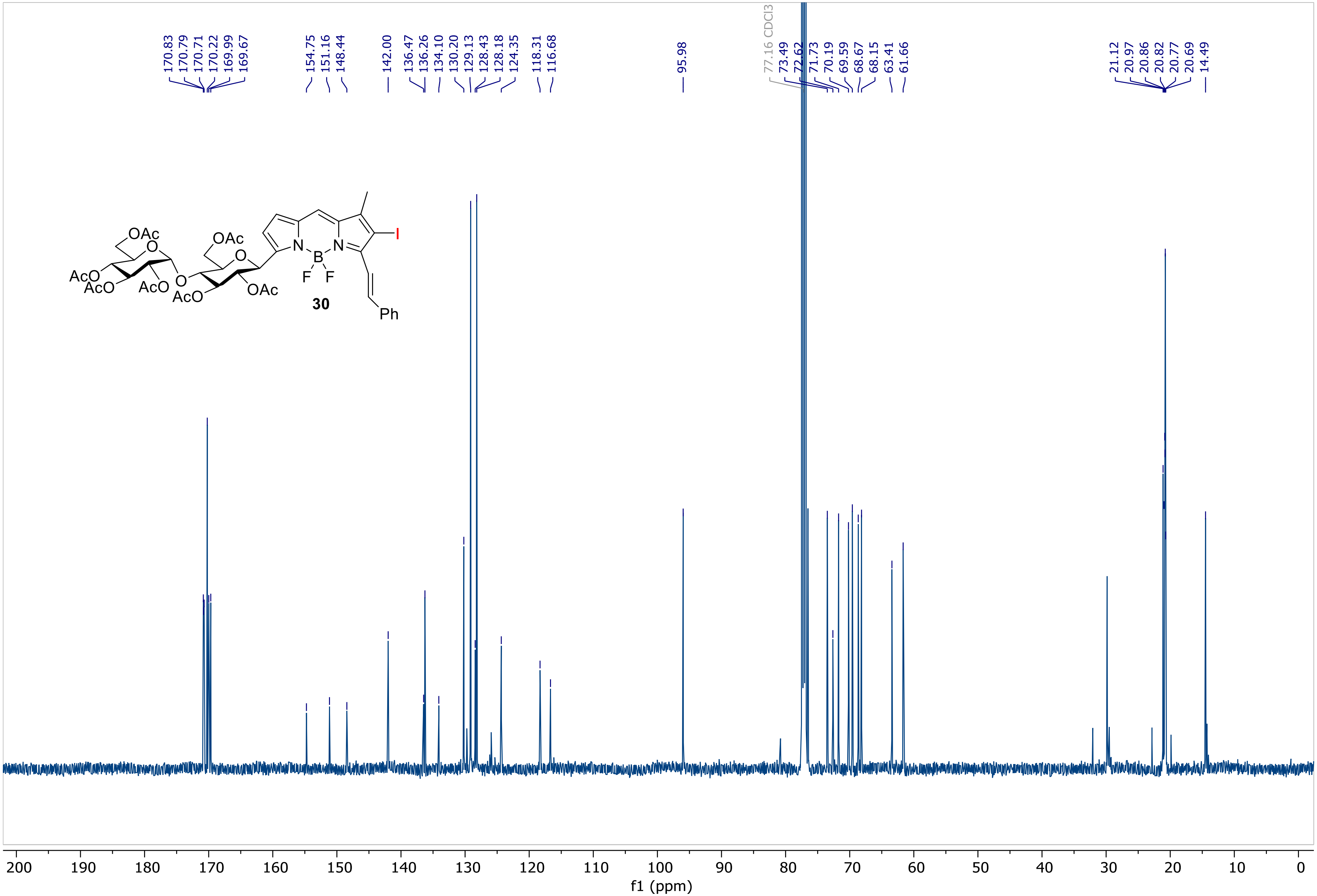

13C{1H} NMR CDCl3 125 MHz

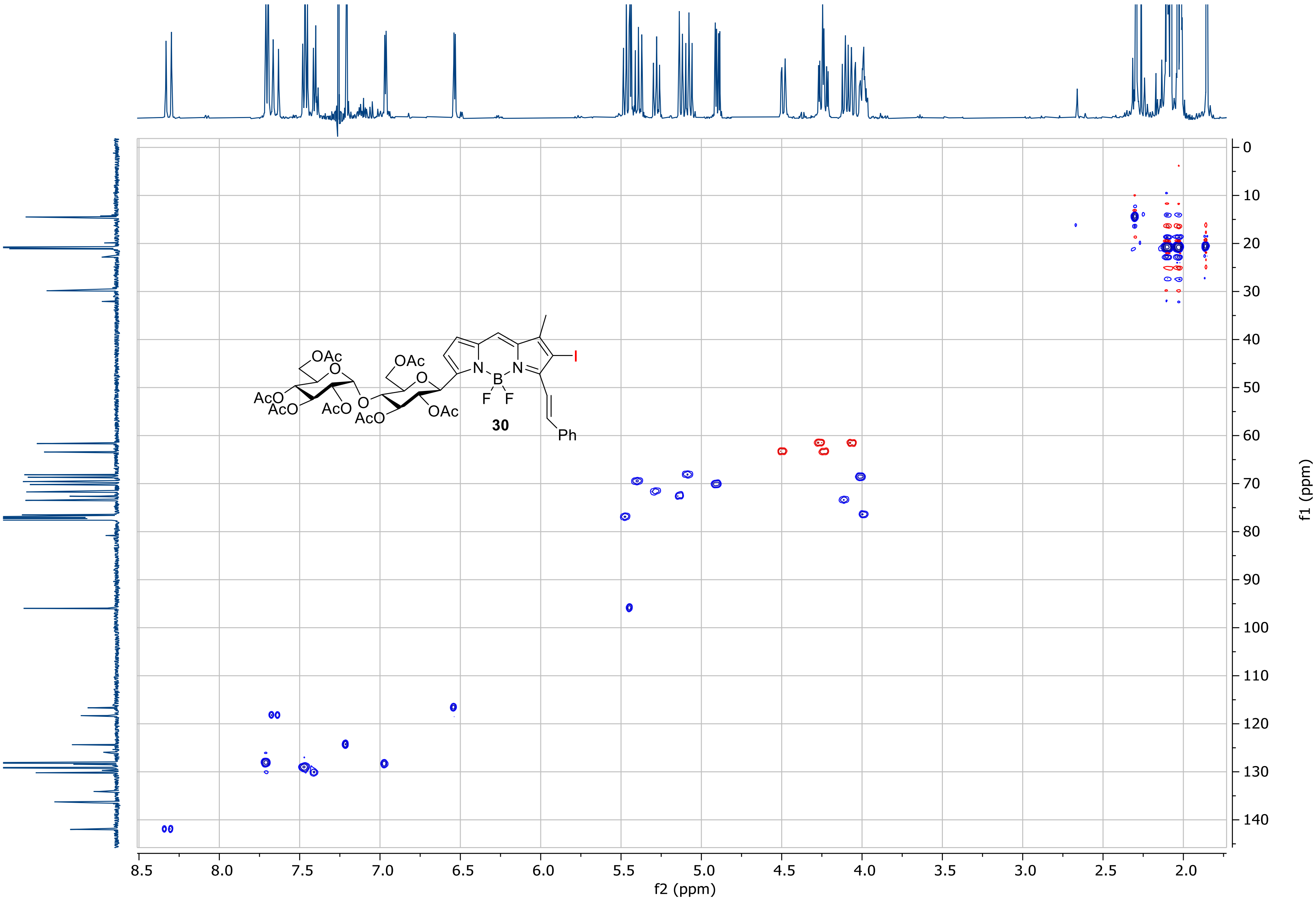

1H-13C gHSQC CDCl3

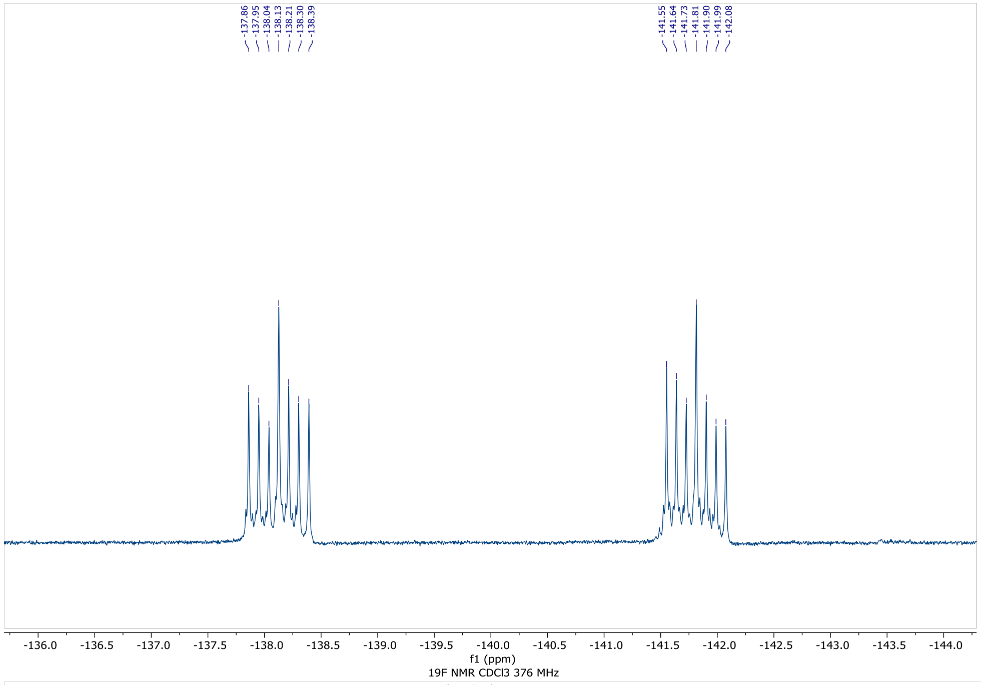
-137.86
-137.95
-138.04
-138.13
-138.21
-138.30
-138.39
-141.55
-141.64
-141.73
-141.81
-141.90
-141.99
-142.08
-136.0 -136.5 -137.0 -137.5 -138.0 -138.5 -139.0 -139.5 -140.0 -140.5 -141.0 -141.5 -142.0 -142.5 -143.0 -143.5 -144.0
f1 (ppm)
19F NMR CDCl3 376 MHz

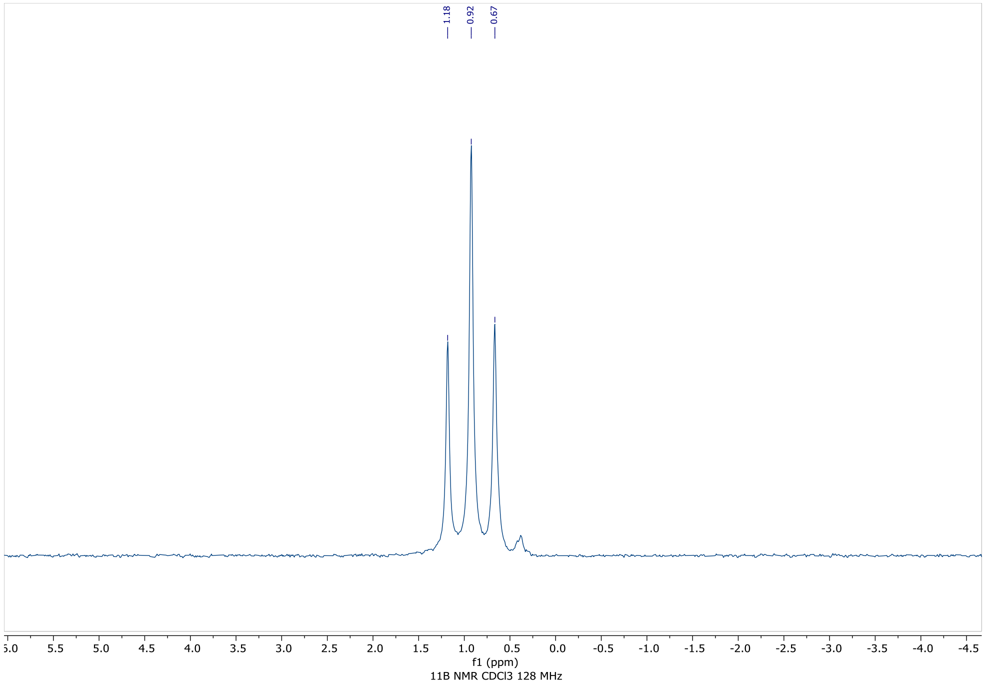
1.18
0.92
0.67
5.5 5.0 4.5 4.0 3.5 3.0 2.5 2.0 1.5 1.0 0.5 0.0 -0.5 -1.0 -1.5 -2.0 -2.5 -3.0 -3.5 -4.0 -4.5
f1 (ppm)
11B NMR CDCl3 128 MHz

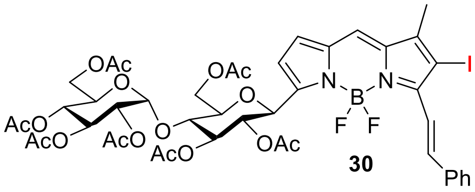
OAc
O
AcO
AcO
AcO
O
OAc
O
AcO
OAc
N
B
N
F
F
I
Ph

30

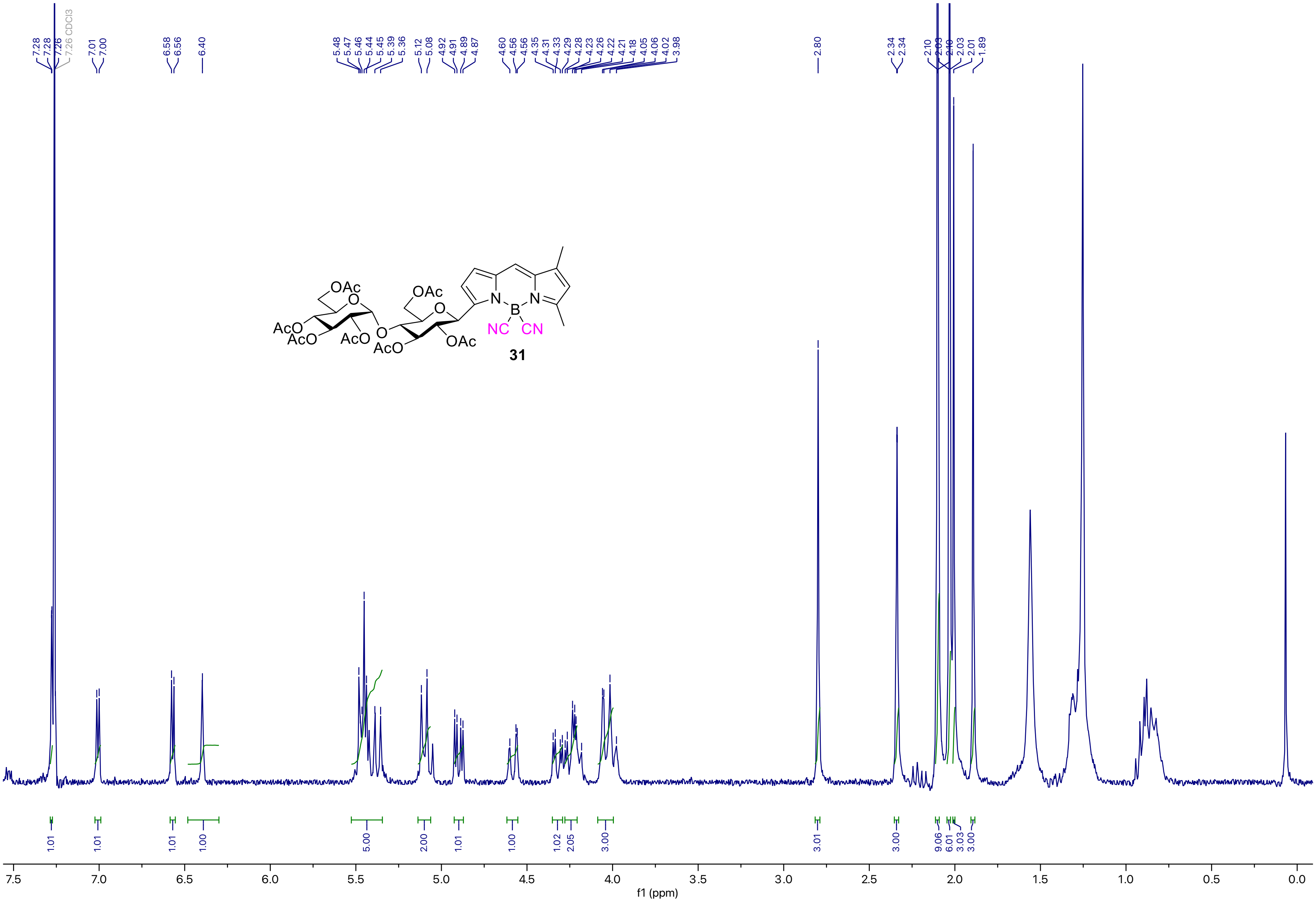

1H-NMR (CDCl3,500 MHz)

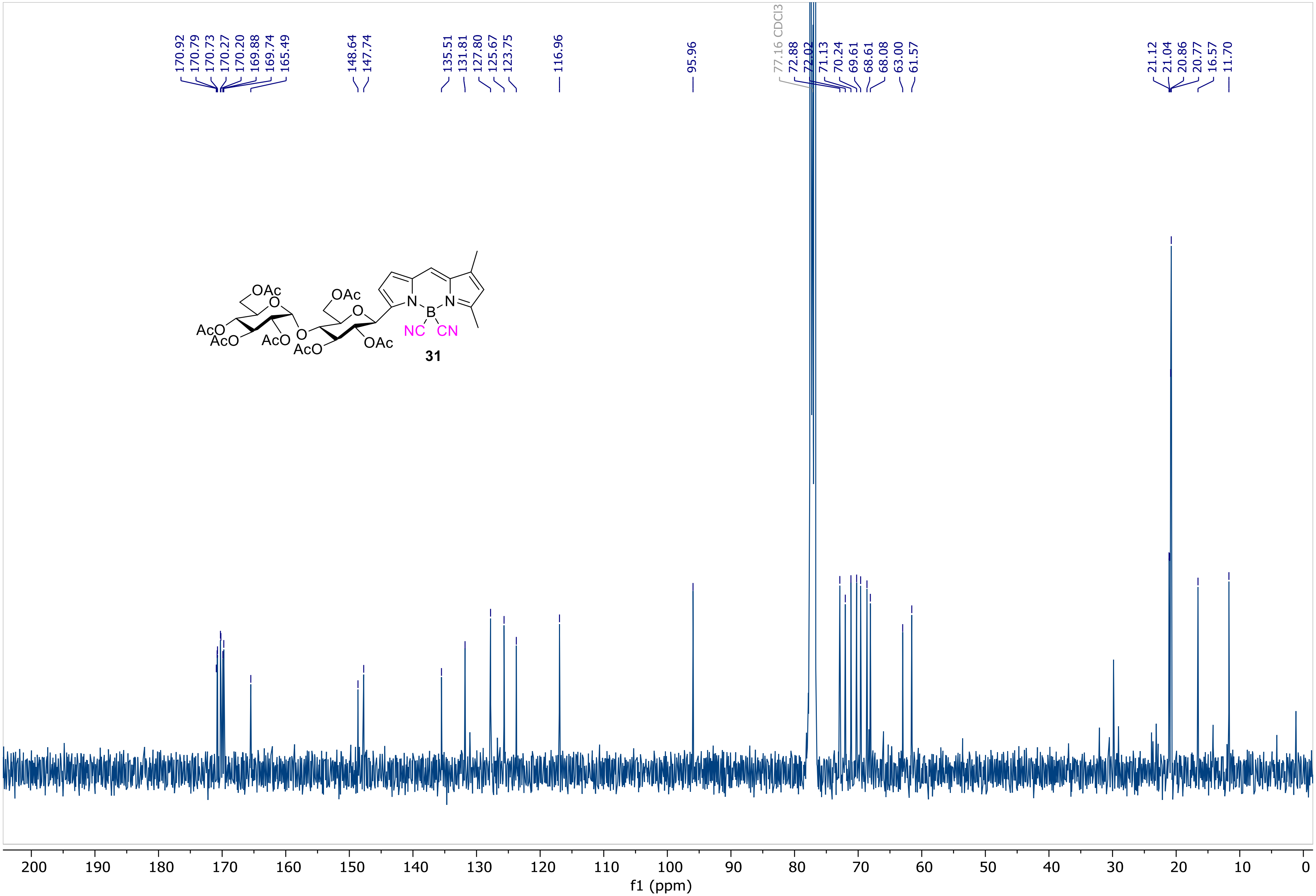

13C{1H} NMR CDCl3 125 MHz

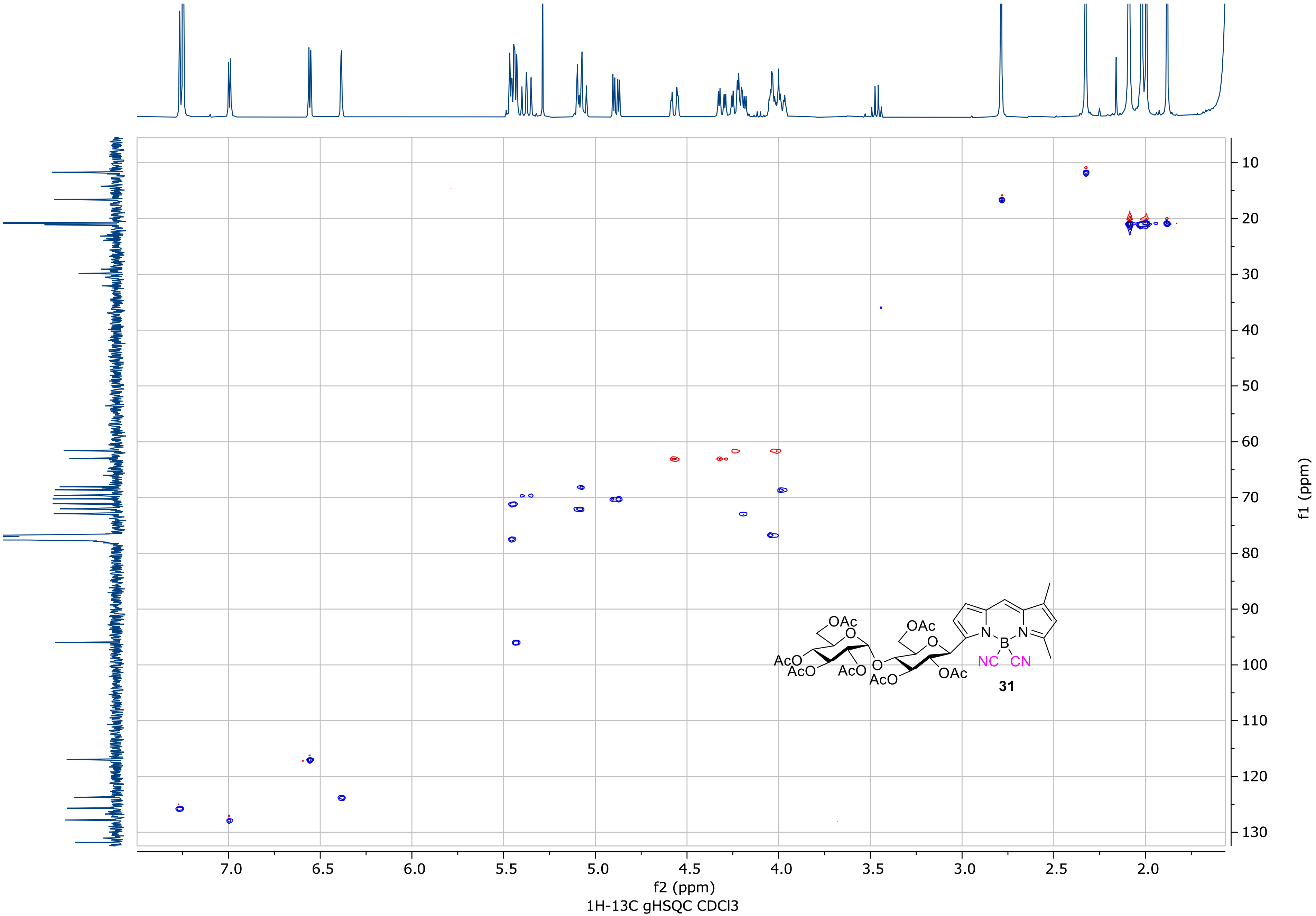

f2 (ppm)
f1 (ppm)
1H-13C gHSQC CDCl3
OAc
AcO
AcO
AcO
AcO
OAc
OAc
NC
CN
31

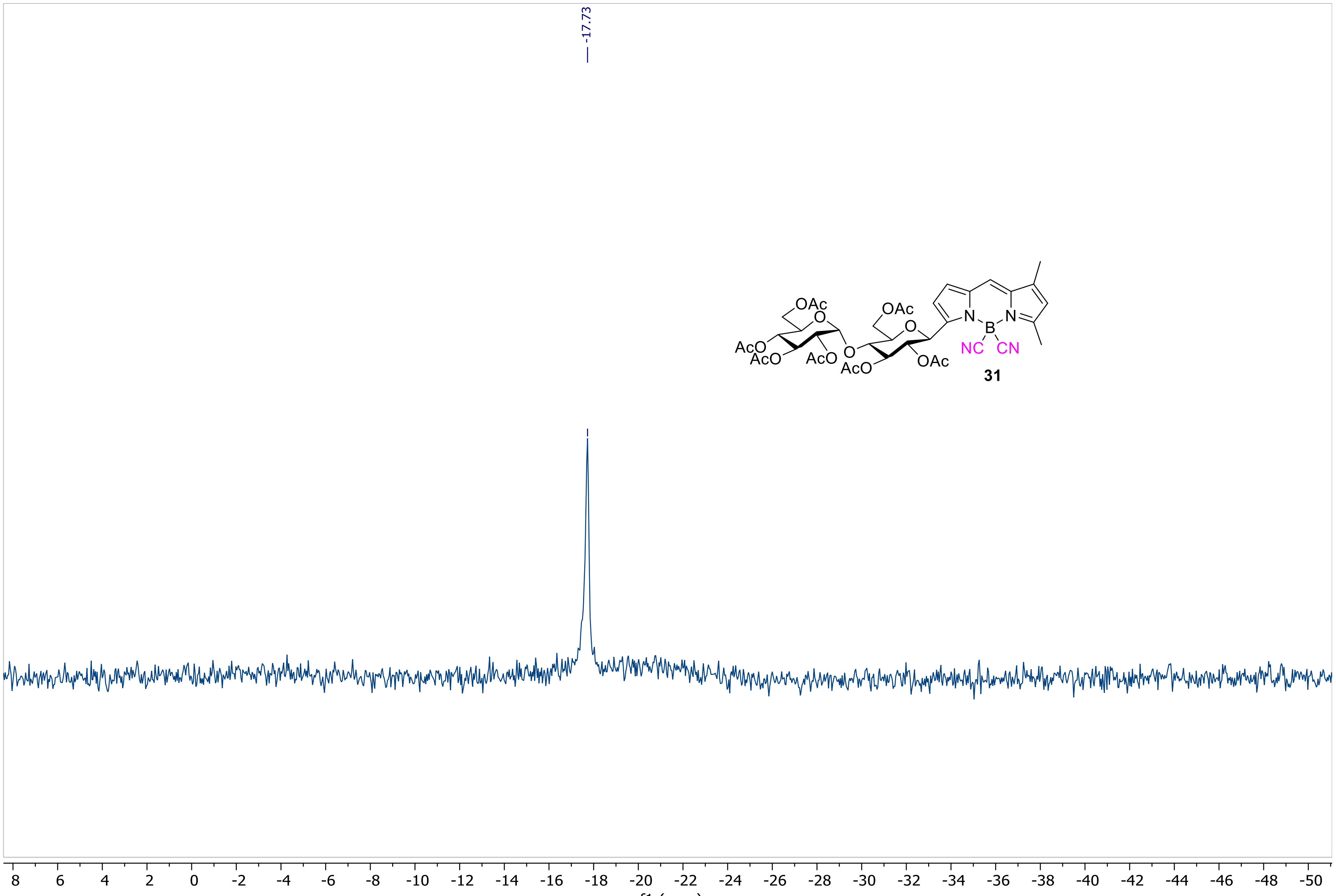

11B NMR CDCl3 128 MHz

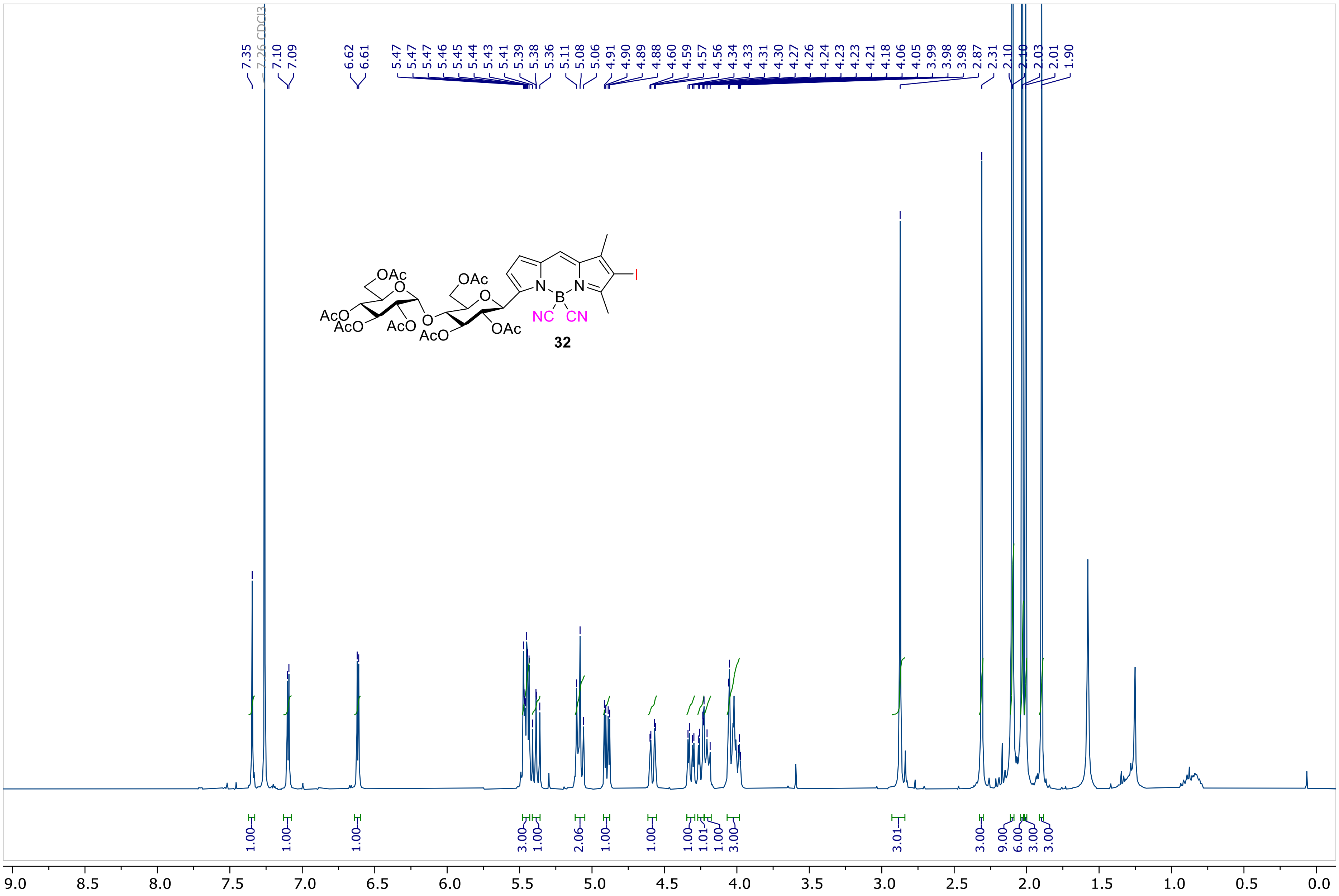

1H NMR CDCl3 400 MHz

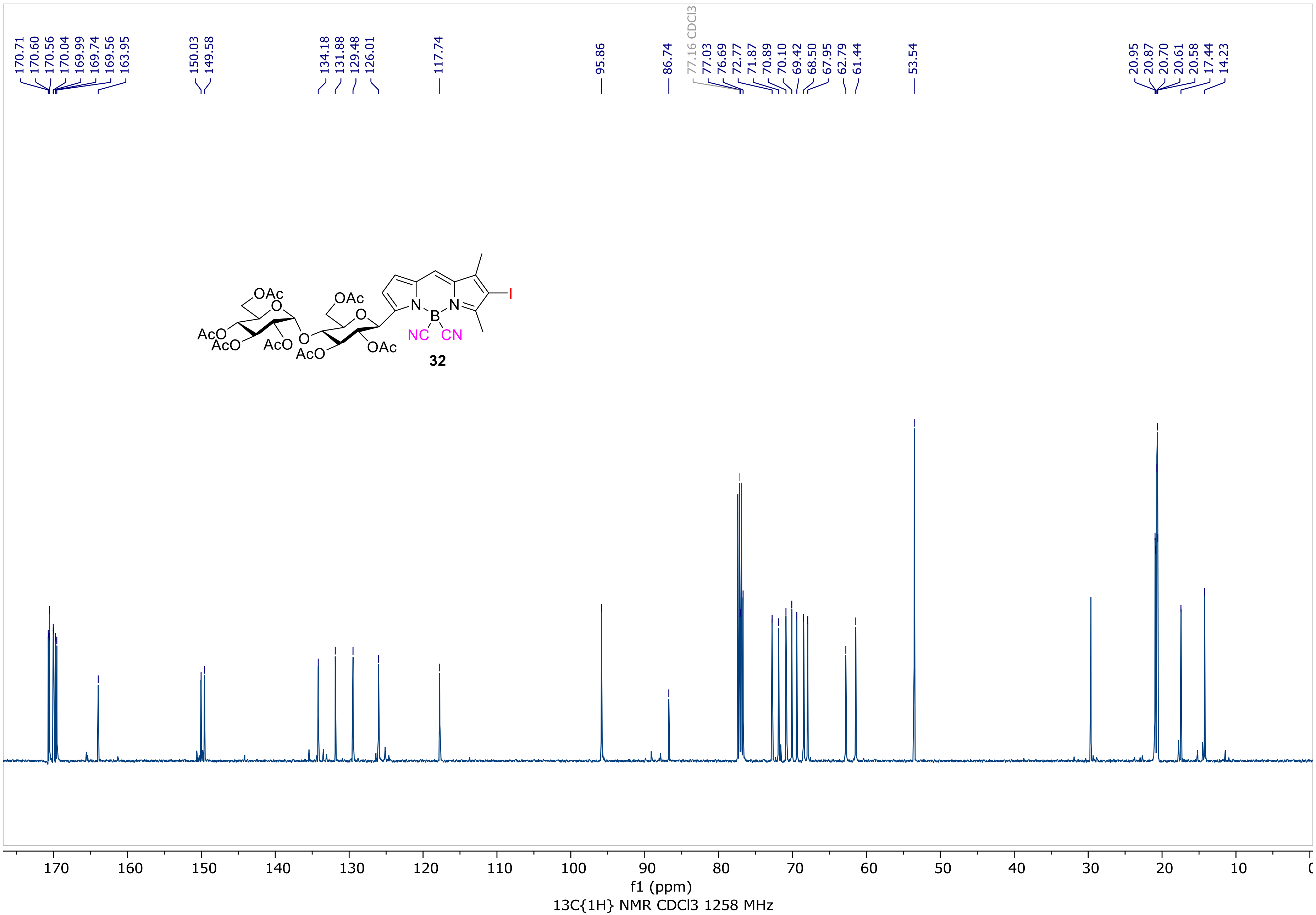
170.71
170.60
170.56
170.04
169.99
169.74
169.56
163.95
150.03
149.58
134.18
131.88
129.48
126.01
117.74
95.86
86.74
77.16 CDCl3
77.03
76.69
72.77
71.87
70.89
70.10
69.42
68.50
67.95
62.79
61.44
53.54
20.95
20.87
20.70
20.61
20.58
17.44
14.23
OAc
AcO
AcO
AcO
O
O
OAc
AcO
OAc
NC
CN
N
B
N
I
32
170 160 150 140 130 120 110 100 90 80 70 60 50 40 30 20 10
f1 (ppm)
13C{1H} NMR CDCl3 1258 MHz

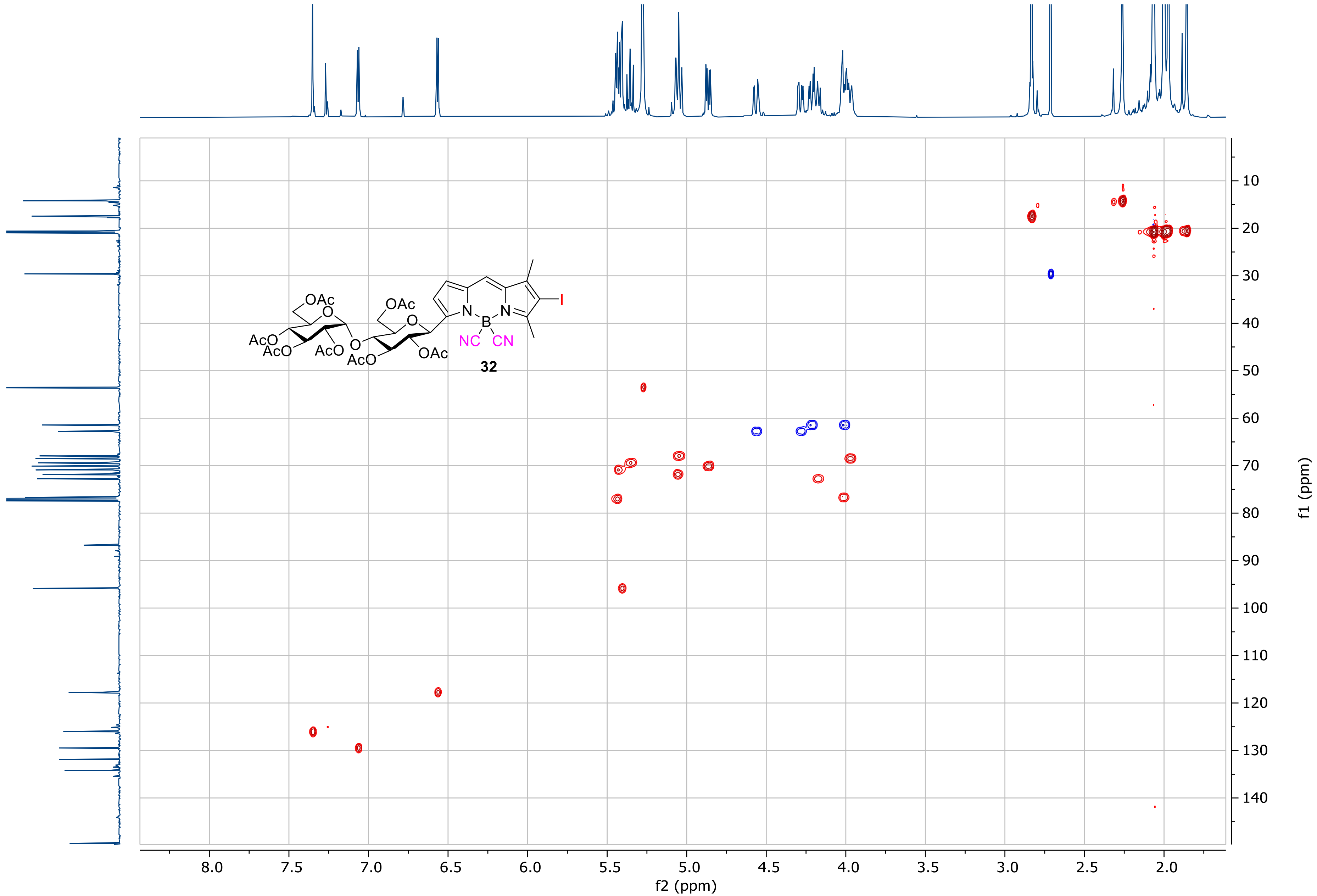

1H-13C gHSQC CDCl3

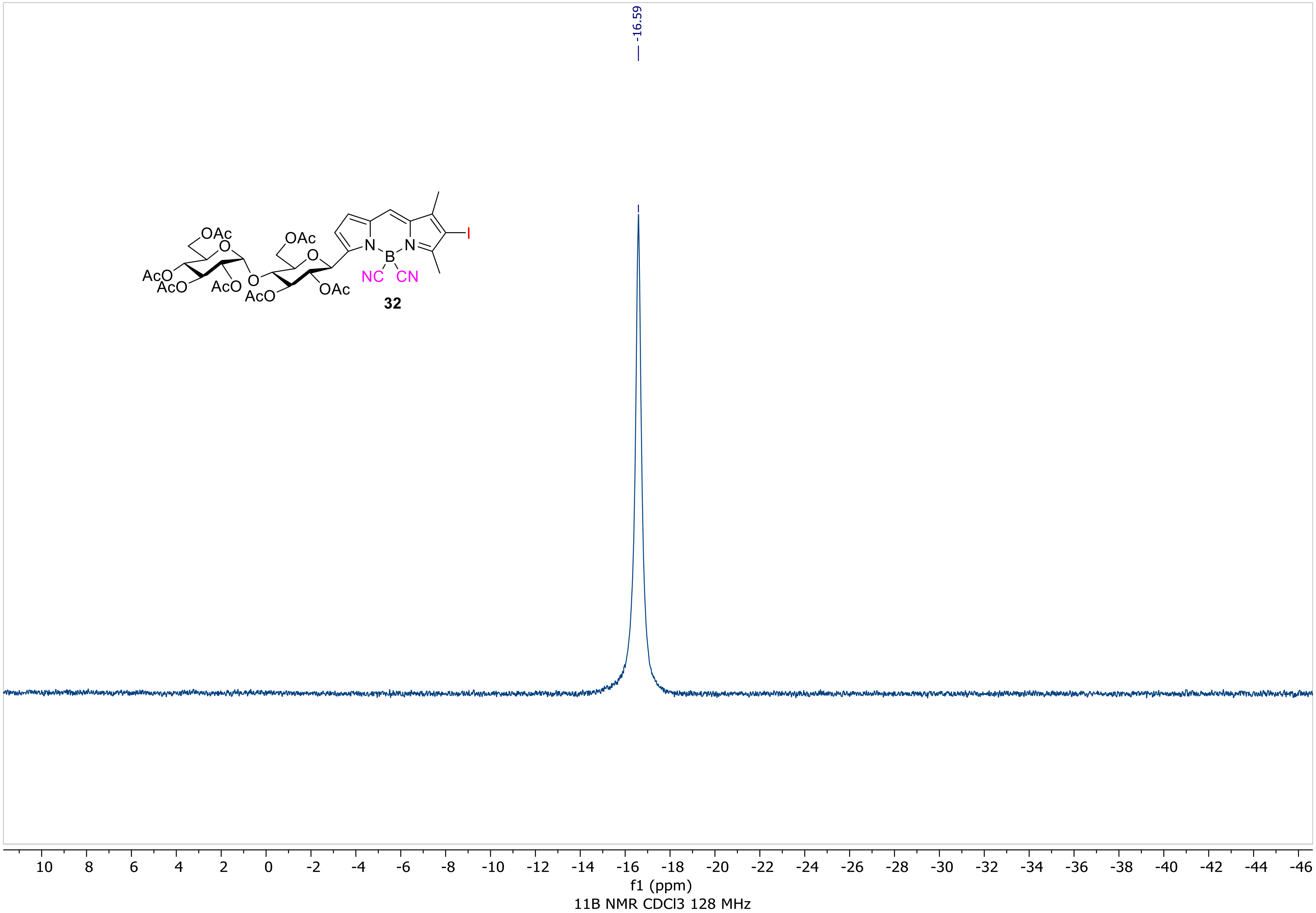
-16.59
OAc
OAc
AcO
AcO
AcO
O
AcO
OAc
N
B
N
I
NC
CN
32
10 8 6 4 2 0 -2 -4 -6 -8 -10 -12 -14 -16 -18 -20 -22 -24 -26 -28 -30 -32 -34 -36 -38 -40 -42 -44 -46
f1 (ppm)
11B NMR CDCl3 128 MHz

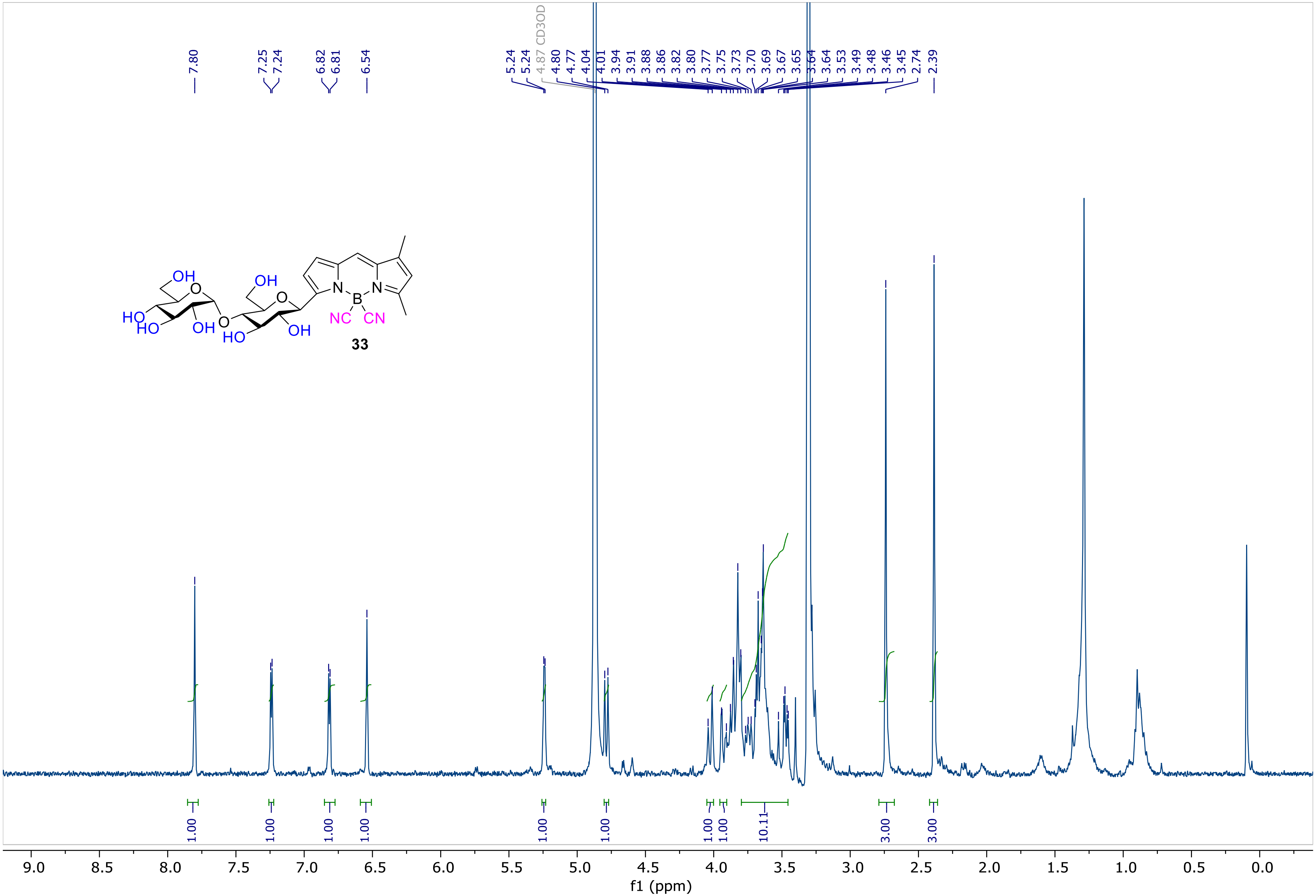

1H NMR CD3OD 400 MHz

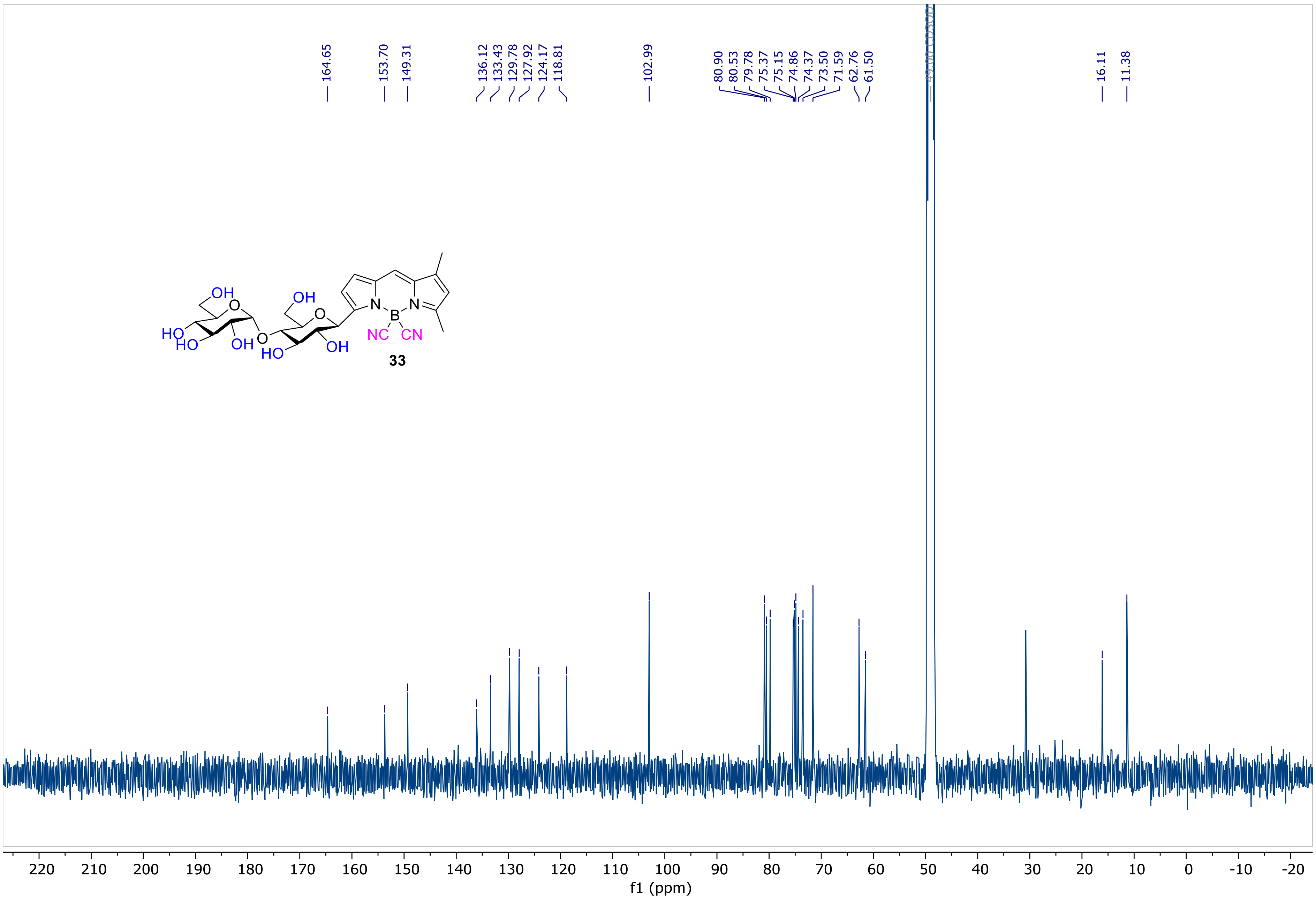

13C{1H} NMR CD3OD 125MHz

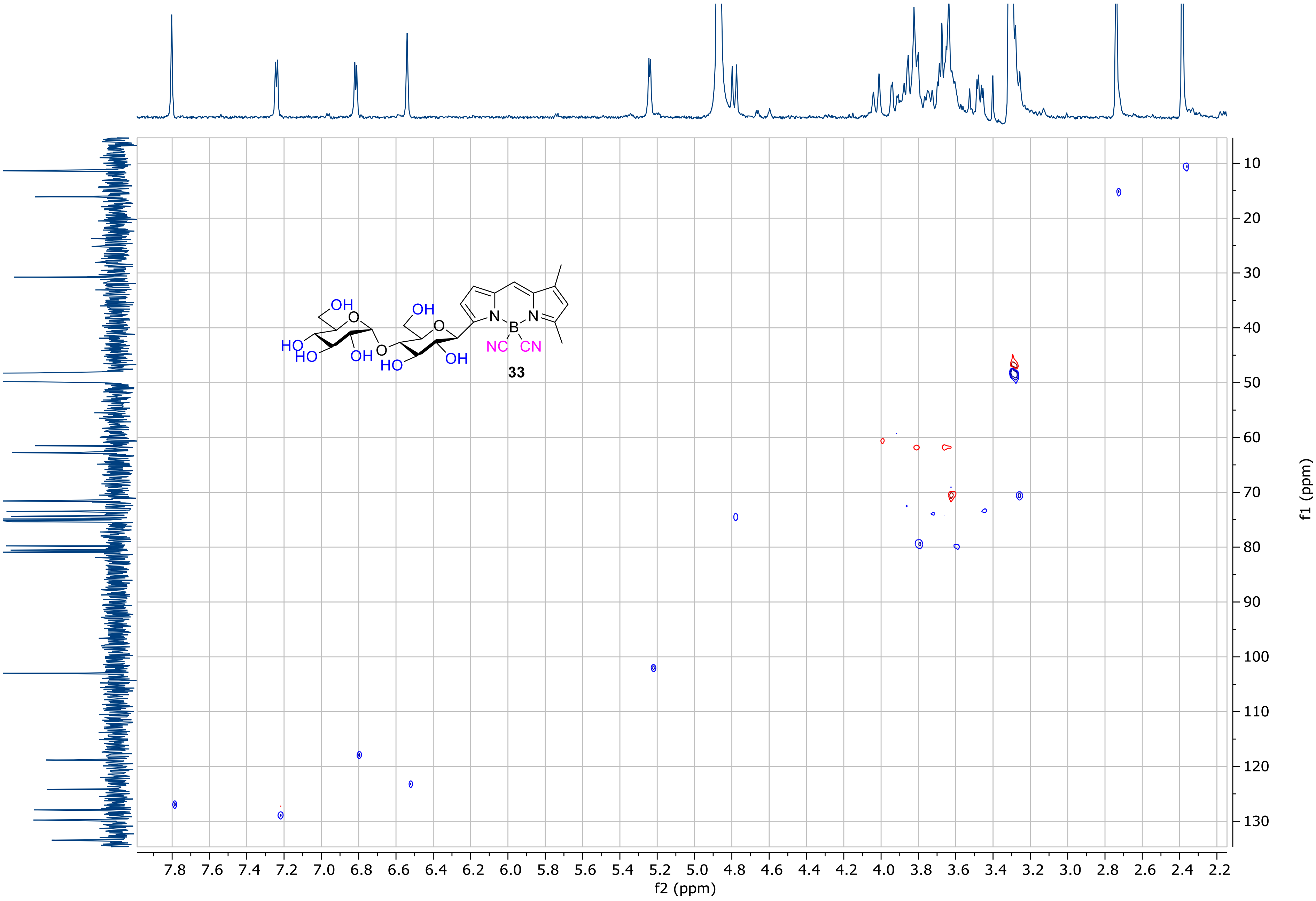

33
f1 (ppm)
f2 (ppm)
1H-13C gHSQC CD3OD

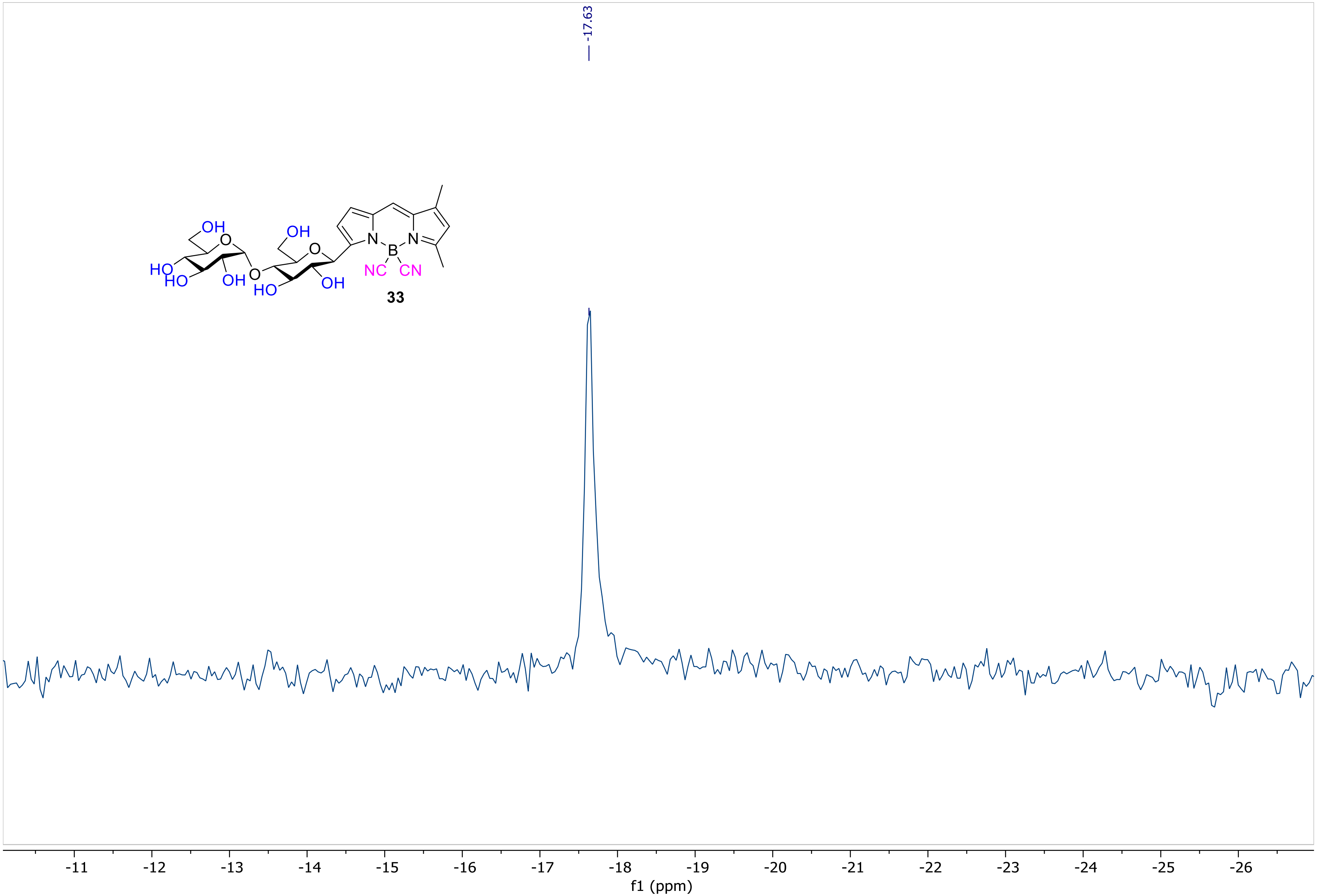

11B NMR CD3OD 128 MHz
